# Genome-wide primary transcriptome analysis of H_2_-producing archaeon *Thermococcus onnurineus* NA1

**DOI:** 10.1038/srep43044

**Published:** 2017-02-20

**Authors:** Suhyung Cho, Min-Sik Kim, Yujin Jeong, Bo-Rahm Lee, Jung-Hyun Lee, Sung Gyun Kang, Byung-Kwan Cho

**Affiliations:** 1 Department of Biological Sciences and KI for the BioCentury, Korea Advanced Institute of Science and Technology, Daejeon 305-701, Republic of Korea; 2 Korea Institute of Ocean Science and Technology, Ansan 426-744, Republic of Korea; 3 Intelligent Synthetic Biology Center, Daejeon 305-701, Republic of Korea

## Abstract

In spite of their pivotal roles in transcriptional and post-transcriptional processes, the regulatory elements of archaeal genomes are not yet fully understood. Here, we determine the primary transcriptome of the H_2_-producing archaeon *Thermococcus onnurineus* NA1. We identified 1,082 purine-rich transcription initiation sites along with well-conserved TATA box, A-rich B recognition element (BRE), and promoter proximal element (PPE) motif in promoter regions, a high pyrimidine nucleotide content (T/C) at the −1 position, and Shine-Dalgarno (SD) motifs (GGDGRD) in 5′ untranslated regions (5′ UTRs). Along with differential transcript levels, 117 leaderless genes and 86 non-coding RNAs (ncRNAs) were identified, representing diverse cellular functions and potential regulatory functions under the different growth conditions. Interestingly, we observed low GC content in ncRNAs for RNA-based regulation via unstructured forms or interaction with other cellular components. Further comparative analysis of *T. onnurineus* upstream regulatory sequences with those of closely related archaeal genomes demonstrated that transcription of orthologous genes are initiated by highly conserved promoter sequences, however their upstream sequences for transcriptional and translational regulation are largely diverse. These results provide the genetic information of *T. onnurineus* for its future application in metabolic engineering.

Archaea are unique organisms with ecological significance that have a similar genome organization and cellular structure to those of bacteria, and comparable molecular transcription and translation mechanisms to those of eukaryotes^1^,^2^,^3^. Particularly, the archaeal transcription apparatus is similar to the eukaryotic RNA polymerase (RNAP) II system, which requires a set of transcription factors to initiate transcription. In an early stage of archaeal transcription, the transcription apparatus, composed of an RNAP and associated initiation factors, is assembled at the promoter region and transcription start site (TSS), defined as the +1 position of the 5′ UTR of mRNA, to form the closed complex, commonly referred to as the pre-initiation complex^4^. Along with the pivotal role of RNAP and its related transcription factors in initiating archaeal transcription, the DNA sequence such as TATA box and BRE is a *cis*-encoded determinant for transcription initiation as a guide signal embedded in the genome^4^. Thus, determining the precise transcript architecture of transcript 5′ ends allows us to reveal diverse *cis*-encoded determinants, including promoter elements, 5′ UTRs, and TSSs. Also, the precise location of TSSs determined by experimental methods provides the sequence and structure of the mRNA 5′ end for investigating transcription regulation, mRNA stability, and translational efficiency^5^.

For genome-scale determination of TSSs, two RNA-sequencing methods have been intensively used for diverse bacterial strains, such as *Escherichia coli, Helicobacter pylori, Streptomyces coelicolor*, and *Synechocystis* sp. PCC6803^5^,^6^,^7^,^8^. Those methods are differential RNA-seq (dRNA-seq) for annotating TSS^7^,^9^ and strand-specific RNA-seq (ssRNA-seq) for measuring mRNA transcript levels^5^,^10^,^11^. In addition to these bacterial species, recent transcriptome sequencing studies on archaeal strains such as *Thermococcus kodakarensis, Methanosarcina mazei, Methanolobus psychrophilus*, and *Haloferax volcanii* identified the genome-wide location of TSS and further revealed the significance of post-transcriptional regulation and extensive ncRNA-based regulation^12^,^13^,^14^,^15^. Along with understanding the *cis*-encoded determinants of each archaeal strain, it is also important to compare upstream non-coding regions to elucidate how closely related archaeal strains respond to unique environmental conditions. Among such archaeal strains, *T. onnurineus* NA1 showed a close phylogenetic relationship to *T. kodakarensis* KOD1 with gene rearrangement at the level of chromosomal segments^16^,^17^,^18^.

Here, we exploited the dRNA-seq method to obtain an accurate map of TSSs across the *T. onnurineus* NA1 genome. *T. onnurineus* NA1 is a hyperthermophilic archaeon belonging to the order of *Thermococcales* with *Pyrococcus* species found in deep-sea hydrothermal vents^16^. This strain has a relatively small genome of approximately 1.8 Mbp, consisting of 2,026 genes, which uses monocarbon substrates such as carbon monoxide (CO) and formate as carbon and energy sources for cell growth under anaerobic conditions^19^. Extensive analyses were then performed to elucidate the *cis*-encoded determinants of transcription initiation and the upstream regulatory regions around the promoters and 5′ UTRs. Also, we generated a map of genome-scale ncRNAs and their differential utilization under formate and CO conditions, which are known to be upregulated for the production of hydrogen gas based on measuring levels of individual transcripts using ssRNA-seq^20^. This comprehensive genome-scale view of transcript architecture using upstream regulatory features provides a better understanding of transcriptional and posttranscriptional regulation in archaeal genomes.

## Results

### Determination of the primary transcriptome

**Figure 1:**
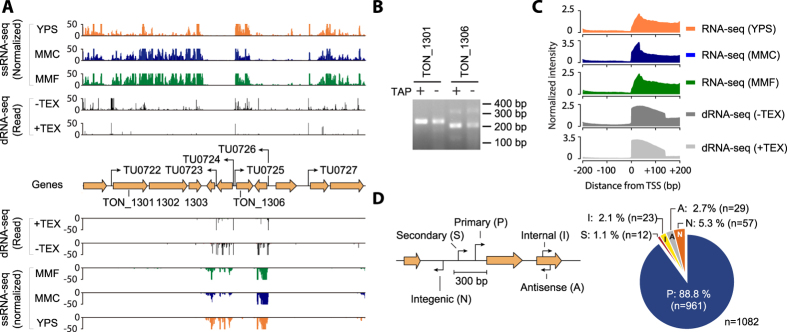
Determining transcriptional architecture of the *T. onnurineus* NA1 genome. (**A**) Example of dRNA-seq and ssRNA-seq profiles mapped onto the *T. onnurineus* NA1 genome. For TSS determination, total RNA samples were isolated from independent biological replicates of the mid-exponential growth phase cultures and two sequencing libraries were constructed, one from TEX treated (+TEX) and the other from untreated total RNA (−TEX). The expression levels of mRNA transcripts were obtained from ssRNA-seq, representing the genomic region between TON_1301 and TON_1310, in yeast peptone sulfur (YPS), MM1-CO (MMC), and MM1-Formate (MMF) media conditions. (**B**) TSS confirmation for TON_1301 and TON_1306 using the 5′ Rapid Amplification of cDNA Ends derivative method (5′tagRACE) and Sanger sequencing. Full-length gels are included in Supplementary Fig. S1. (**C**) Average normalized intensity of each position for maximum peak within ±200 nt from the identified TSSs. (**D**) A total of 1,082 TSSs were identified and classified according to their positions relative to adjacent ORFs. TSSs located from 300 bp upstream to 50 bp downstream of the start codon of the annotated ORF were classified as the primary (P) or secondary TSSs (S). The peaks found within the coding regions were assigned as the internal TSSs (I). TSSs located within the reverse strands and the annotated ORFs were classified as the antisense (A) and intergenic TSSs (N), respectively.

To determine the TSSs in the *T. onnurineus* NA1 genome, a dRNA-seq method was exploited and the abundance of RNA transcripts was quantified using ssRNA-seq method (Fig. 1A). Briefly, we isolated total RNA from independent biological replicates of mid-exponential growth phase cultures and obtained primary transcripts by removing transcripts lacking tri-phosphorylated 5′ ends (e.g., rRNA and tRNA) and any degraded transcripts by treating with terminator exonuclease (TEX)^6^,^7^,^11^. As a control, a TEX-untreated cDNA library was prepared in parallel, which represents the 5′ ends of the whole transcriptome including the intact, processed, and degraded RNAs. Consequently, two independent cDNA libraries were constructed, which were TEX treated (+TEX) and untreated (−TEX), and both were sequenced on an Illumina sequencing platform. The resulting sequence reads were trimmed and mapped to the reference genome (NC_011529), resulting in 67- and 247-fold coverage obtained for +TEX and −TEX libraries, respectively (Supplemental Table S1)^16^. Additionally, the amount of sequence reads mapped to ribosomal RNA (rRNA) were 0.03–0.04%, indicative of a high rRNA depletion efficiency for the archaeal dRNA-seq method^14^.

By integrating the mapping results with plausible criteria (see Materials and Methods for detection criteria), we newly assigned 1,082 TSSs in the *T. onnurineus* NA1 genome (Supplemental Table S2). Only TSSs present in both TEX+ and TEX− libraries within ±5 bp resolution were retained. TSSs were then curated using iterative cluster subdivision as described previously with some modifications^21^. These enriched signals enabled the determination of TSSs for TUs by the contiguous gene expression signals obtained from ssRNA-seq^6^,^11^. Currently, a total of 2,026 genes are annotated in the *T. onnurineus* NA1 genome including 1,975 protein-coding genes^16^. In addition, 1,161 operons have been predicted by the DOOR^2^ database, where 1,224 genes are organized into 410 multi-gene operons (number of genes in an operon ≥2) and other operons are transcribed from a single structural gene (751 genes in total)^22^. We identified primary TSSs from the upstream sequences of 834 operons (71.8%). Among those, 302 multi-gene operons were determined by the primary TSSs (73.7%) (Supplemental Table S2). For example, the transcription of TON_1301, TON_1302, and TON_1303 is initiated by a single TSS at the genomic position of 1,182,769 indicating that these genes are transcribed from a single TU (TU0722) (Fig. 1A). The operon harboring the largest number of genes was a ribosomal operon consisting of 23 genes (from TON_0065 to TON_0088). Although TSS information is useful to predict the TU architecture encoded in bacterial and archaeal genomes, it has limited by the absence of the length information of mRNA transcribed from the TU and the conditional use of TSS in responses to the environmental conditions. For instance, it was predicted that the longest TU is 16,819 bp in length encoding 18 genes (from TON_1563 to TON_1580) involved in H_2_ production^16^. However, it can be also speculated that the presence of additional TSSs within the long TU. Furthermore, independent verification of the identified TSSs was obtained by using the 5′ Rapid Amplification of cDNA Ends derivative method (5′tagRACE) and Sanger sequencing^23^. For instance, we obtained the targeted PCR products for several TSSs from the cDNA library (Supplemental Fig. S1), such as those for TON_1301 (237 nt) and TON_1306 (204 nt) (Fig. 1B). The 5′ end sequence of each PCR product was confirmed by Sanger sequencing (Supplemental Fig. S1).

The primary transcript profile was correlated with the normalized ssRNA-seq profiles, which showed a sharp signal increase at the TSS and covered up to 150 bp downstream from the start codons of the annotated ORFs (Fig. 1C). The ssRNA-seq profiles also indicated a sharp signal at the same TSS positions, however the maximum peak signals were observed within 25–50 bp downstream. This is consistent with the fact that most mRNAs in the ssRNA-seq libraries lack 20–30 nt from their proximal 5′ terminus^24^. This suggests that the canonical ssRNA-seq profiles reflect both intact transcripts with preserved 5′ ends and processed (or degraded) transcripts. TSSs were further categorized by their genomic locations and levels of enrichment (Fig. 1D and Supplemental Table S2), giving 961 primary TSSs (P) that were selected as a maximum peak height TSSs located within 300 bp upstream from the start codon of the annotated ORF. The secondary TSSs (S) were collected from the second highest peak in the same region as the primary TSSs, accounting for 12 ORFs, and the internal TSSs (I) were called from the peaks found within 23 coding regions. Interestingly, *T. onnurineus* NA1 showed a low preference for secondary TSSs (1.1%) under the growth conditions examined, which is similar with the finding that the closely related archaeon *T. kodakarensis* showed less use of the secondary TSSs (7.8%)^12^. The antisense (A) and intergenic TSSs (N) were collected from reverse strands of 29 annotated coding strands and 57 non-coding regions, respectively. Consequently, primary TSSs correspond to 88.8% of detected TSSs and the other TSSs were represented in the remaining 11.2% (Supplemental Table S2).

### Analysis of 5′ UTRs

**Figure 2:**
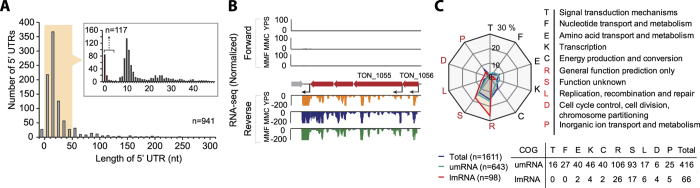
Analysis of 5′ UTRs in *T. onnurineus* NA1. (**A**) Distribution of 5′ UTR lengths is shown within 300 nt. Another distribution at 0–5 nt was found and considered to produce leaderless mRNAs (lmRNAs, n = 117). (**B**) Examples of these lmRNAs are illustrated (TON_1055 and TON_1056). (**C**) Functional enrichment analysis of proteins encoded by the umRNAs (n = 643) and lmRNAs (n = 98) using COG mapping.

We next examined the length distribution of 5′ UTRs between the defined primary TSSs and start codons of 941 annotated protein-coding genes. The 5′ UTR sequence promotes ribosomal binding through the Shine-Dalgarno sequence and frequently influences translational efficiency and post-translational regulation^25^. The primary transcript 5′ UTR lengths were mostly 0–50 nt (84.6%), with a median length of 12 nt and the longest length of 479 nt (Fig. 2A and Supplemental Fig. S2). Interestingly, we observed TSSs lacking 5′ UTRs defined as leaderless mRNAs (lmRNA), which is consistent with the fact that archaeal mRNAs typically have short 5′ UTRs^26^,^27^,^28^. In contrast, bacterial 5′ UTR lengths show a median length of 55 nt defined as 5′ UTR-associated leadered mRNA (umRNA)^12^,^27^,^29^. For instance, TON_1056 and TON_1055 encode an unknown protein and a 3-methyladenine DNA glycosylase, respectively (Fig. 2B). Based upon the fact that the length of the consensus archaeal RBS in the 5′ UTR is 6 nt, transcripts having 5′ UTRs less than 5 nt were classified as lmRNA. Previous studies showed that translation of bacterial lmRNA was highly efficient when the genomic position of start codon is identical with the transcription start position (i.e., 5′ UTR length = 0) and the limit of the 5′ UTR length for lmRNA translation was ~5 nt^27^. A total of 117 TSSs (12.4% of primary TSSs) generated a substantial subset of lmRNAs. In particular, 70% of the lmRNAs showed complete overlap with transcriptional initiation sites with the annotated start codon (i.e., the length of 5′ UTR = 0).

To determine whether lmRNAs are over- or underrepresented in certain biological functions, the umRNAs and lmRNAs were categorized into clusters of orthologous groups (COGs), respectively. The results showed that a total of 416 umRNAs (64.7%) and 66 lmRNAs (67.3%) were assigned to COG categories (Fig. 2C)^30^. Both the major portion of umRNAs (47.8%) and lmRNAs (65.2%) were highly enriched in groups R and S, which represent unknown functions, while the residual transcripts were found across diverse functions including replication, recombination, repair, inorganic ion transport, and metabolism. The number of lmRNAs in the *T. onnurineus NA1* genome is similar with the numbers in *T. kodakarensis* (~14%)^12^ and *Methanolobus psychrophilus* (~15%)^14^. However, compared to those identified in *Sulfolobus solfataricus* P2 (~69%)^31^, *Haloferax volcanii* (72%)^15^, or *Pyrococcus abyssi* (few lmRNAs)^32^, this observation suggests that the usage of leaderless translation is evolutionarily diverse among archaeal species. However, most proteins encoded by lmRNAs have not been functionally annotated yet.

### Characterization of promoters and RBSs

**Figure 3:**
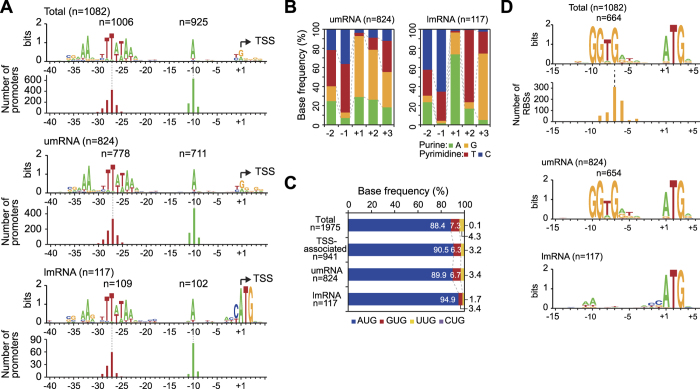
Genome-scale analysis of upstream sequences. (**A**) Determination of promoter elements. TATA boxes (5′-TTWTAW), polyA BRE motifs upstream of the TATA box, and PPE motifs “A” at −10 were identified relative to the TSS (+1). The bottom panels show the conservation of each motif of umRNAs and lmRNAs. (**B**) Proportion of each nucleotide at TSS (+1) and two nt upstream and downstream of the TSS of umRNAs and lmRNAs. The pyrimidine-purine dinucleotide motif is shown at −1 and +1 positions. (**C**) Start codon usage of all ORFs (primary TSS-associated ORFs, umRNAs, and lmRNAs. **(D)** Conserved ribosome-binding site (RBS) motif (5′-GGTG) for umRNAs (n = 796) and lmRNAs (n = 117). For lmRNAs, the absence of an RBS was confirmed.

Archaeal promoters contain two major conserved sequence elements, the A/T rich TATA box sequence of 8 bp and BRE, which is approximately centered around 27 nt and 33 nt upstream from the TSS^4^. In addition to these two major conserved sequences, the A/T-rich promoter proximal element (PPE) is located approximately −10 base pairs upstream of the TSS, and the initiator element (INR) is conserved within the initially transcribed region. It has been suggested that both PPE and INR elements are not required for initiating transcription, but they can regulate the strength of transcription output^3^. To determine the promoter elements in the *T. onnurineus NA1* genome, we searched the conserved sequences across the genome between −50 nt and +10 nt from the identified TSSs using MEME^33^. 1,006 TSSs (93.0%; *p* < 0.05) and 925 TSSs (85.5%) among the total 1,082 TSSs showed the conserved TATA box and A-rich BRE motif sequences, respectively. We found that 94.4% and 86.3% (778 and 711 out of 824, respectively, *p* < 0.05) of the umRNA and 93.2% and 87.2% (109 and 102 out of 117, respectively, *p* < 0.05) of the lmRNA represented highly conserved TATA box and BRE motif, respectively (Fig. 3A). The TATA box was identified between −30 and −20 positions and the BRE sequence appeared at 5 bp upstream of the TATA box. All primary, secondary, internal, antisense, and intergenic promoters indicated conserved TATA box and BRE motif, which is consistent with analyses from other archaeal species (Supplemental Fig. S3)^12^,^14^,^31^. An A/T rich sequence at the −10 position as a PPE motif is in agreement with the results in *P. abyssi, T. kodakarensis*, and *S. solfataricus*^12^,^31^,^32^. In addition, no difference in the promoter motif sequence was observed between highly and lowly expressed genes, indicating that upstream regulatory sequences are critical to control gene expression (Supplemental Fig. S3).

We further examined the nucleotide composition around TSSs (positions −2 to +3) (Fig. 3B). Both umRNAs and lmRNAs showed high purine nucleotide contents (A/G) of 93.1% and 97.4% at the +1 position and high pyrimidine nucleotide content (T/C) at the −1 position with 87.0% and 94.9%, respectively. These nucleotide preferences at +1 and −1 positions are found in archaea, bacteria, *S. cerevisiae*, and mammalian promoters to initiate transcription from the identical purine and pyrimidine preferences^34^,^35^. Interestingly, this dinucleotide preference reflects that lmRNA prefers a ‘C’ over other nucleotides at the −1 position. This could be related with direct recognition of the sequence by the ribosomal complex for initiating translation without interacting with the SD sequence^36^. In addition, ‘ATG’ was highly used as a translational start codon for both umRNA and lmRNA (Fig. 3C). The lmRNAs showed a high ATG codon usage of 94.9%, supporting that in addition to the pyrimidine preference at the −1 position, the conserved ATG sequence is required for leaderless translation.

Next, we searched for the SD sequence motif between −20 nt and +10 nt from the start codons (Fig. 3D). Most genes presented the highly conserved motif (*p* < 0.05), GGDGRD, which matches well with the motif found in the *T. kodakarensis* genome^12^. As expected, this motif was highly conserved in the umRNAs (79.4%, *p* < 0.05), however no such motif was found in the lmRNAs. Instead, the PPE motif was found −10 nt from the transcription initiation site. Additionally, leaderless translation in *T. onnurineus* NA1 was mainly observed for monocistronic single genes and the first gene of an operon, and was not preferred for polycistronic transcripts greater than three genes (Supplemental Table S2).

### Analysis of differential transcript levels according to growth conditions

To determine changes in transcript levels, cDNA libraries for ssRNA-seq were also constructed from the rRNA-depleted total RNAs using a ligation-free cDNA synthesis procedure based on terminal-tagging^37^. Total RNAs were isolated from mid-exponential growth phase cultures in three different media conditions: yeast extract-peptone-sulfur (YPS) media, modified minimal media containing formate (MMF), and modified minimal media containing CO (MMC). YPS media is a rich media routinely used for cell growth, and formate in MMF media is oxidized to CO_2_ and H_2_^19^,^38^. Particularly, *T. onnurineus* NA1 utilizes CO in MMC media as an energy and carbon source for H_2_ production^39^. Thus, we expected that changes in gene transcript levels under the different growth conditions facilitate the understanding of how *T. onnurineus* NA1 regulates its transcriptome for energy metabolism and H_2_ production. We used an Illumina sequencing platform to sequence the cDNA libraries, resulting in more than 1.8 × 10^7^ sequence reads (Supplemental Table S1). After removing sequence reads mapped to ribosomal genes and tRNA genes, approximately 90% of the sequence reads mapped to the reference genome with over 400-fold coverage and high strand-specificity. Pairwise correlation coefficients of the sequencing results demonstrated significant differences in gene expression between the growth conditions (Supplemental Fig. S4). Furthermore, independent qPCR analysis was conducted using selected genes representing a broad range of expression levels (Supplemental Fig. S4 and Supplemental Table 3). A comparison of the ssRNA-seq data and qPCR results showed the validity and reproducibility of ssRNA-seq based quantification (R^2^ = 0.92) (Supplemental Fig. S4). Then, the ssRNA-seq data were further quantified using DESeq package to analyze the differential gene expression under the various growth conditions (Supplemental Table S4)^40^. Comparing the expression levels of umRNAs to those of lmRNAs, umRNAs showed significantly higher expression than lmRNAs under different growth conditions (Kruskal-Wallis test, *p* < 0.0005) (Supplemental Fig. S5).

**Figure 4:**
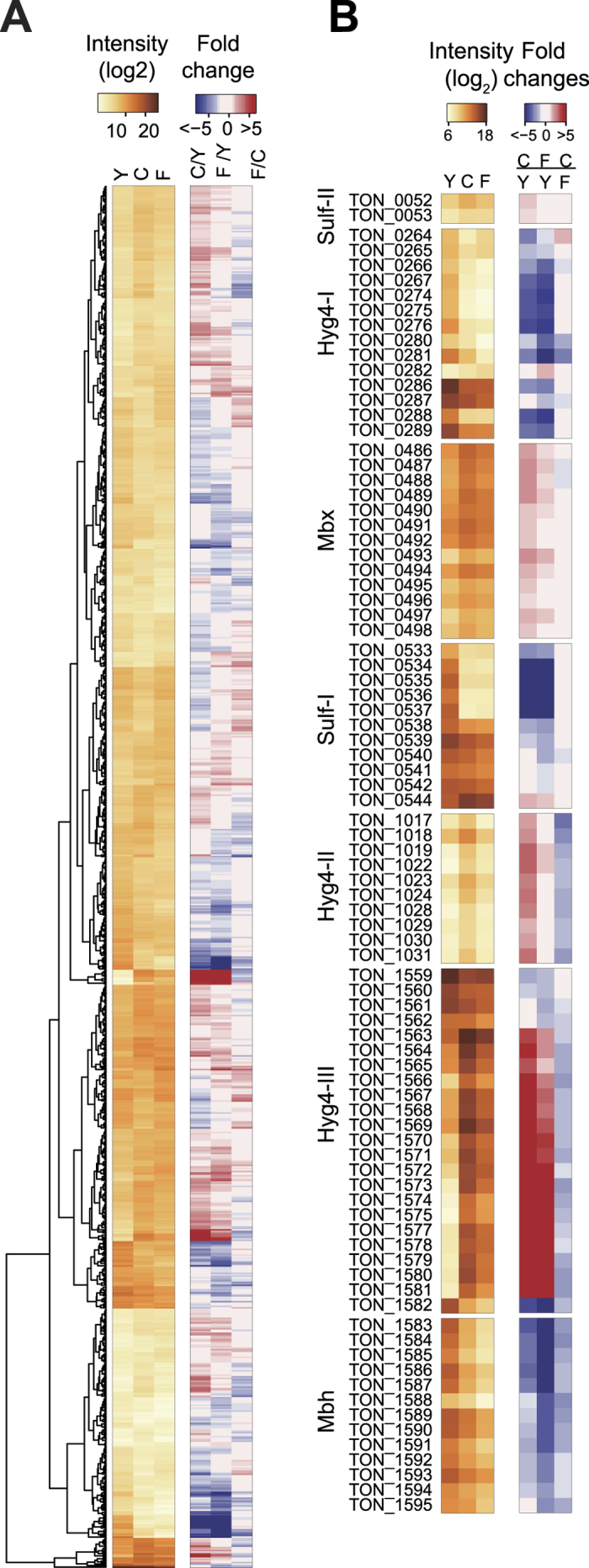
Dynamic RNA expression changes according to the gene function. (**A**) Normalized RNA expression and differential expression according to the culture media; YPS (“Y”), MMC (“C”), and MMF (“F”) as shown using a heat map. (**B**) The RNA expression and regulation of hydrogenase-related genes were shown in three hydrogenase clusters (Hyg4), sulfhydrogenase (Sulf), membrane-bound oxidoreductase (Mbx-I), and membrane bound hydrogenase (Mbh) clusters using a heat map.

It is estimated that *T. onnurineus* NA1 has at least 2,000 genes, of which 922 genes showed two-fold or greater changes in expression between MMC and YPS, and MMF and YPS conditions (*p* < 0.05) (Fig. 4A). The expression of energy production and inorganic ion transport-related proteins were significantly changed (Supplemental Fig. S6). Particularly, the *T. onnurineus* NA1 genome contains a number of hydrogenases and their related proteins such as membrane bound hydrogenase (Mbh), membrane bound oxidoreductase (Mbx), and ion transporters in Hyg4-I, Hyg4-II, Hyg4-III, and Sulf-I, Sulf-II clusters^16^. Among those, genes in Hyg4-II, Hyg4-III clusters, and Mbx showed increased transcript levels under MMF or MMC growth conditions (Fig. 4B and Supplemental Fig. S6). Whereas, the transcript levels of Hyg4-I, Mbh, and two cytoplasmic NiFe-hydrogenases (Sulf-I and Sulf-II) were downregulated. The catalytic subunit of CO dehydrogenase (TON_1018), a key CO metabolic enzyme, was significantly increased only in MMC growth media. Among the cytoplasmic NiFe-hydrogenases, the Sulf-I cluster was markedly downregulated (Normalized RNA expression <30) in the MMC and MMF growth conditions due to the absence of sulfur. However, all genes in the Sulf-I cluster were highly activated more than 2-fold by sulfur in YPS media. Taken together, hydrogen production under the presence of CO and formate seems to be largely driven by the Hyg4-II and Hyg4-III clusters, respectively.

### Determination of noncoding RNAs

**Figure 5:**
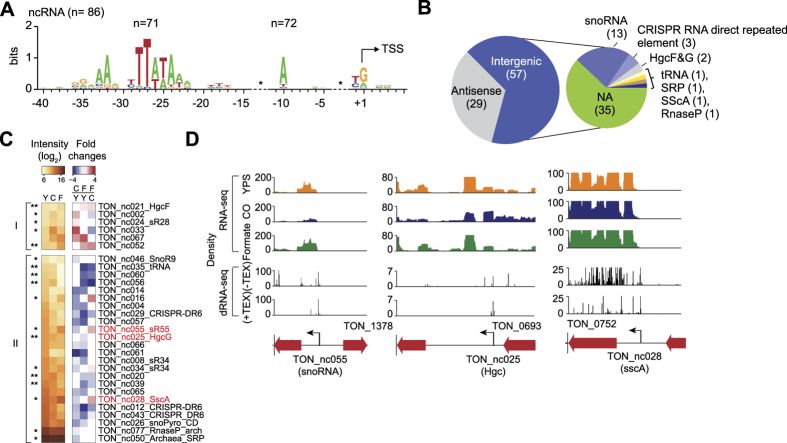
Characterization of ncRNAs. (**A**) Determining promoter elements from the region upstream of ncRNAs. Each motif (TATA boxes, BREs, and PPE motifs) was identified. (**B**) ncRNAs were classified according to their location (antisense and intergenic). The intergenic ncRNAs were further determined using functional groups based on Rfam. (**C**) The ncRNAs with expression changes over two fold in YPS, MMC, and MMF media are displayed with a heatmap. Groups I and II indicate upregulated and downregulated ncRNAs in MMC and MMF against YPS growth media, respectively. (**D**) Examples of ncRNAs expression profiles are displayed for snoRNA (TON_nc055), Hgc (TON_nc025), and sscA (TON_nc028) with the dRNA-seq profiles.

We found 86 putative ncRNAs with TATA box, BRE, and PPE motif at their upstream regions (Fig. 5A), encompassing the antisense (29 ncRNAs) and the intergenic (57 ncRNAs) regions (Fig. 5B and Supplemental Table S5). A previous genome annotation failed to identify these ncRNAs^16^, likely because of the lack of related information in the database. Furthermore, TSSs of ncRNAs were verified by using 5′tagRACE and Sanger sequencing (Supplemental Fig. S7). To analyze the functional roles of the ncRNAs, we exploited the RFAM database using sequence-based alignments, consensus secondary structures, and covariance models^41^. The functions of 22 intergenic ncRNAs were characterized with *E*-values lower than 10^−9^ (Supplemental Table S6), including 13 small nucleolar RNAs (snoRNAs) known to guide the chemical modification of rRNAs and tRNAs by promoting complex formation with essential proteins^42^. It also included three CRISPR RNA direct repeat elements, an archaeal RNase P ribozyme, an archaeal signal recognition particle RNA (SRP), and the HgcF/HgcG RNAs which were previously verified in AT-rich hyperthermophiles^43^. The ncRNAs showing expression changes >2-fold in responses to CO or formate were represented as the upregulated group (I) and downregulated group (II) (Fig. 5C). Among 22 ncRNAs, 15 ncRNAs showed the expression changes >2-fold and most ncRNAs were downregulated in the experimental growth conditions. For instance, TON_nc055, TON_nc025, and TON_nc028 encoding snoRNA, Hgc, and sscA, respectively, exhibited reduced expression levels in MMC growth media (Fig. 5D). These results suggest that ncRNAs are actively involved in regulating genes related to energy metabolism in response to CO and formate in *T. onnurineus* NA1.

### Comparative analysis of upstream regulatory regions

**Figure 6:**
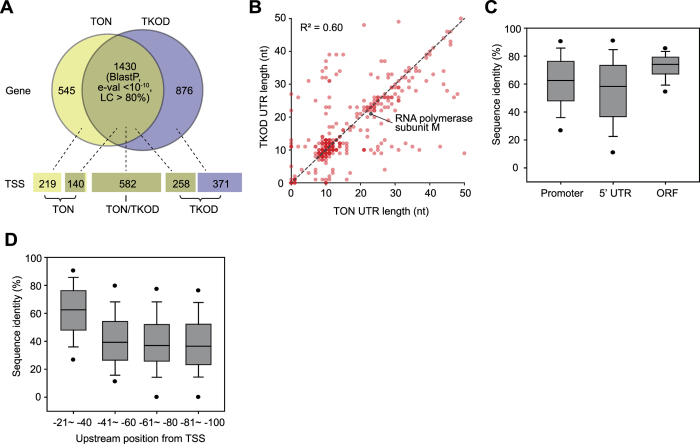
Comparative analysis of regulatory elements in closely related archaea. (**A**) Orthologous genes and species-specific genes of *T. onnurineus* NA1 and *T. kodakarensis* KOD1. (**B**) Differences in 5′ UTR length between pairs of orthologous ORFs within 50 nt. (**C**) Comparison of sequence conservation between promoters and 5′ UTR regions of orthologous ORFs and ORF sequences. (**D**) Comparison of upstream regions (−21 and −100) of the comparable pairs of orthologous ORFs.

The *T. onnurineus* NA1 genome showed high similarity to the genome of *T. kodakarensis* KOD1, another H_2_ producing archaea^16^. Although archaeal genomes show highly conserved promoter elements in general, it is unknown whether those archaea regulate the expression of orthologous genes with similar upstream regulatory sequences. To investigate whether the orthologous genes are regulated similarly or differently, we performed a comparative analysis of the upstream regulatory sequences of orthologous genes between the closely related archaea *T. onnurineus* NA1 and *T. kodakarensis* KOD1. The orthologous genes present in both archaea were selected by all-versus-all BLASTP comparison between the protein-coding genes with an *E*-value threshold of 1×10^−10^ and coverage higher than 80%, resulting in a set of 1,430 orthologs (Fig. 6A and Supplemental Table S7). Among those, 722 and 840 orthologous genes were assigned by primary TSSs in *T. onnurineus* NA1 and *T. kodakarensis* KOD1 genomes, respectively, and the remaining TSSs were assigned to unique genes. Thus, further comparative analysis of regulatory elements in upstream regions was performed with the orthologous pairs assigned by TSSs (582 pairs in total) (Supplemental Table S7).

Using the primary TSSs of the orthologous genes, we calculated the distribution of 5′ UTR lengths; they have a similar pattern with a median length of 11 nt for both archaeal genomes (Fig. 2D and Supplemental Fig. S8). This is consistent with the fact that archaeal 5′ UTRs are relatively shorter than bacterial 5′ UTRs^12^,^29^. In particular, among the lmRNAs in both archaea, a total of 50 lmRNAs encode orthologous proteins with mostly unknown functions. The comparison of 5′ UTR lengths showed a correlation (R^2^ of 0.60) between the orthologs and 166 (28.5%) of them were identical in length (Fig. 6B and Supplemental Table S8). For example, the 5′ UTR length of TON_0824 encoding RNA polymerase subunit M was 23 nt, while that of its orthologous gene (TK0533) in *T. kodakarensis* KOD1 was 21 nt (Fig. 6B). Then, the 5′ UTR conservation in the archaeal species was calculated from the sequence alignment of the 5′ UTRs from ortholog pairs. The 5′ UTR sequences were more diverse than promoter sequences and ORFs between orthologous genes in the archaea species (Fig. 6C), whereas the sequences of ORFs and promoters were 74.1% and 62.5% conserved, respectively. The 5′ UTR sequences were diverse (58.3% conservation), however the SD motifs (GGDGRD) of the RBSs were well conserved in both archaea, which were located around 6 nt from the TSS. To calculate promoter sequence conservation, we extracted the sequences between −21 and −40 relative to the TSS in both archaea. The unexpected low promoter conservation level (62.5%) might be due to sequence variations around TATA boxes and BRE motifs. In addition to the promoter and 5′ UTR sequences, the comparison of upstream sequences between −41 and −100 exhibited a gradual decrease in sequence identity with increasing distance from TSS (Fig. 6D). This might indicate the presence of different regulatory sequences for the conserved orthologous genes, such as transcription factor binding sites. Interestingly, among the hydrogenase clusters, Mbh, Mbx, SulfI, and a part of Hyg4-III clusters of *T. onnurineus* NA1 were found in *T. kodakarensis* KOD1 as orthologous pairs (Supplemental Fig. S9). Among them, the Sulf-I cluster, encoding the cytosolic Ni-Fe hydrogenase, showed a highly conserved promoter, RBS, and upstream regulatory sequence in both archaea (e-value < 2 × 10^−7^). Interestingly, ORFs (TON_0538–TON_0540) in the Sulf-I cluster encoding formate transporter, formate dehydrogenase subunit alpha (*fdhA*), and oxidoreductase iron-sulfur protein were lacking in the *T. kodakarensis* KOD1 genome^16^,^18^. Instead, ORFs (TK2073–TK2075), to orthologous TON_0541–TON_0543 encoding 4Fe-4S binding proteins and glutamate synthase, exhibited similar regulatory sequences located upstream of the omitted TON_0538. Taken together, although *T. onnurineus* NA1 and thermophilic archaea *T. kodakarensis* KOD1 have a highly conserved proteome, the regulatory regions embedded in sequences upstream of the TSS are diverse.

## Discussion

The primary transcriptome landscape with differential gene expression enables a mechanistic understanding of transcriptional and translational regulation for organisms under given conditions. In this study, we determined the primary transcriptome of the hyperthermophilic archaeon *T. onnurineus* NA1 using dRNA-seq so that the *cis*-regulatory components were cataloged on a genomic scale. In contrast to other archaea^3^,^12^,^31^,^32^, most TSSs (89.9%) in the *T. onnurineus* NA1 genome were highly embedded upstream of ORFs, however some TSSs (10.1%) were also found within ORFs, the antisense strand, and non-coding regions. In addition to the conserved promoter elements, although this is less understood, it has been suggested that archaeal 5′ UTRs contain the initiator element motif (INR), with high AT content, which affects promoter strength through interacting with transcription activators or facilitating promoter opening^3^,^44^. As reported for *Sulfolobus solfataricus* P2^44^, we found a weakly conserved motif (GAGAT) located at positions +2 to +6, indicating that its sequence variation may contribute to initiating transcription (Supplemental Fig. S10).

In spite of the critical roles of 5′ UTRs, the absence of a 5′ UTR (i.e., lmRNA) seems like a widespread regulatory strategy in archaea^12^,^14^,^31^,^45^. Along with the highly conserved promoter elements, it has been presumed that translation initiation is mediated by direct binding of the 30S ribosomal subunit or 70S ribosome and an initiator f-Met-tRNA to the AUG^36^,^46^,^47^. Consistent with the fact that the ‘AUG’ start codon is a significant determinant for leaderless translation, the identified lmRNAs highly utilize AUG as the translation start codon (94.9%) in *T. onnurineus* NA1. In addition, 5′ end phosphates are known to positively affect translation efficiency^48^,^49^. Thus, lmRNAs without leader sequences (0 nt in length) may be preferable for leaderless translation, which is consistent with the observation that over 70% of the lmRNAs lack a leader sequence (Fig. 2A). In spite of the considerable number of lmRNAs, the cellular functions of most lmRNAs are currently unknown and also show lower expression levels than umRNAs^50^. Interestingly, the different levels of lmRNAs among different archaeal species may indicate that lmRNAs are involved in regulatory mechanisms of archaeal adaptation to a wide range of environmental conditions.

To date, a wide range of ncRNAs has been identified in all the three domains of life, which are involved in various cellular functions including post-transcriptional regulation^51^. We found 15 out 22 differentially expressed ncRNAs, which were related with energy metabolism (closely related with H_2_ production) under different growth conditions. No significant difference in promoter element sequences was found, so the ncRNAs may be regulated by other transcription factors or riboswitches^52^. In contrast to the genomic GC content (51%) and due to the thermophilic growth of *T. onnurineus* NA1, most ncRNAs were AT-rich (48% GC content), and they may function as unstructured forms or be stabilized by interacting with molecules such as nucleic acids or proteins^12^,^32^,^53^. Interestingly, these unique RNA-based regulations of ncRNAs in thermophilic archaea are often observed in other archaea, such as *P. abyssi* and *T. kodakarensis*^12^,^32^.

In conclusion, an important attribute of the primary transcriptome landscape, along with comparative genomics, is that it defines the potential regulatory sequences at upstream of each ORF, where a wide range of transcriptional regulators bind to control gene expression. This complete genome analysis has provided us with much information about the genomic features of different or closely related organisms; mostly insights into genome evolution, pathogenicity, and the core genome^54^,^55^,^56^. Similar to the core genome concept, comparing regulatory sequences of orthologous ORFs in closely related archaea provides information regarding whether the genes are regulated by similar regulatory mechanisms. Thus, comparative genome analysis coupled with primary transcriptome analysis can aid in annotating ORFs and determining regulatory features of lesser-studied archaeal species.

## Methods

### Cell culture and media

*T. onnurineus* NA1 cells were cultured anaerobically at 80 °C to mid exponential phase with growth rate of 0.32 h^−1^, 0.21 h^−1^ and 0.31 h^−1^ in yeast extract-peptone-sulfur (YPS), modified minimal-CO (MMC), and modified minimal-formate (MMF) medium, respectively^20^. YPS medium was prepared as previously described^57^. MMF or MMC media were prepared by adding 1% sodium formate or 1 bar CO gas into the modified minimal media (MM1) containing 0.1% yeast extract, 3.5% NaCl, 0.07% KCl, 0.39% MgSO_4_, 0.04% CaCl_2_·2H_2_O, 0.03% NH_4_Cl, 0.015% Na_2_HPO_4_, 0.003% NaSiO_3_, 0.05% NaHCO_3_, and 0.05% cysteine-HCl. The medium was supplemented with 0.1% (v/v) each of Holden’s trace element and Balch’s vitamin solution^57^,^58^.

### RNA purification

Duplicate cell cultures in the exponential growth phase were harvested and stored at −80 °C prior to use. After thawing, the resulting cell pellet was used to obtain total RNA by extraction with TRIzol reagent (Invitrogen, Carlsbad, CA). DNase I (Thermo Scientific Inc., Waltham, MA) treatment was performed at 37 °C for 30 min to remove genomic DNA from the isolated total RNA. Total RNA was further purified by chloroform extraction and ethanol precipitation. Purified RNA concentration was determined using a NanoDrop 1000 spectrophotometer (Thermo Scientific Inc.) and quality was checked by visualization using the Experion system (Bio-Rad, Hercules, CA) and by measuring the A260/A280 ratio.

### Strand-specific RNA-seq (ssRNA-seq)

A Ribo-Zero rRNA Removal Kit (Epicentre, Madison, WI) was used to deplete rRNAs in accordance with the manufacturer’s protocol and rRNA removal was confirmed using the Experion system. The rRNA-depleted RNA samples were converted into sequencing libraries using a ScriptSeq RNA-seq library preparation kit (Epicentre). After confirming the quality of the sequencing libraries using TOPO cloning (Invitrogen), they were subsequently quantified using a Qubit 2.0 fluorometer (Invitrogen) and the Experion system. The samples were then sequenced on a Genome Analyzer IIx platform (Illumina Inc., San Diego, CA) with read length of 36 nucleotides.

### Differential RNA-seq (dRNA-seq)

Two libraries were prepared using the modified dRNA-seq method as described previously^7^,^10^. Total mRNA was split into two samples for preparing two different libraries: the primary transcriptome library and the whole transcriptome library. First, we treated the rRNA-depleted RNA samples with 1 U of Terminator 5′-Phosphate-Dependent Exonuclease (TEX, Epicentre), 2 μL of 10 × Terminator Reaction Buffer A (Epicentre), and 20 U of RNaseOUT (Invitrogen) at 30 °C for 1 hr. This reaction enriched intact 5′ tri-phosphorylated mRNAs by removing 5′ mono-phosphorylated rRNA and any degraded mRNA. The reaction was terminated by adding 1 μL of 100 mM EDTA (pH 8.0), and purified by phenol-chloroform extraction and ethanol precipitation. Second, we treated the rRNA-depleted RNA samples with 20 U of RNA 5′ polyphosphatase (TAP, Epicentre) without TEX treatment. To ligate a 5′ adapter (5′-GUUCAGAGUUCUACAGUCCGACGAUC), the tri-phosphates at the mRNA 5′ ends were converted to 5′ mono-phosphates with 20 U of TAP in a 20 μL reaction containing 2 μL of 10 × TAP reaction buffer (Epicentre) and 20 U of RNaseOUT at 37 °C for 1 hr, followed by phenol-chloroform extraction and ethanol precipitation. Ligation was performed in a reaction containing 20 U of T4 RNA ligase (Epicentre), 2 μL of 10 × T4 RNA Ligase buffer (Epicentre), 2 μL of 10 mM ATP, and 20 U of RNaseOUT at 37 °C for 3 hr. Then, the ligated products were incubated with the random 3′ overhanging primer (N9; 5′-GTGACTGGAGTTCAGACGTGTGCTCTTCCGATCTNNNNNNNNN) at 70 °C for 10 min followed by incubation at 25 °C for 10 min. The first-strand cDNA samples were then prepared by incubation with 600 U of SuperScript III reverse transcriptase, 6 μL of 100 mM DTT, 3 μL of 10 mM dNTP mix, 1 μL of actinomycin D (1 mg/mL), and 30 U of RNaseOUT at 25 °C for 10 min, 37 °C for 1 h, 42 °C for 1 h, and 70 °C for 15 min, sequentially. The reaction was then chilled to 4 °C. To remove residual RNAs, the reverse transcribed products were incubated with 20 μL of 1 N NaOH at 65 °C for 30 min, followed by neutralization with 20 μL of 1 N HCl. The synthesized cDNA was purified using a QIAquick PCR Purification Kit (Qiagen, Hilden, Germany) according to the manufacturer’s instructions and then purified again by ethanol precipitation. The cDNA fragments ranging from 100 to 350 bp were then fractionated from a 2% agarose gel by Pippin Prep (Sage Science, Beverly, MA). The cDNA samples were amplified with a forward primer (5′-AATGATACGGCGACCACCGAGATCTACACGTTCAGAGTTCTACAGTCCGA) and indexed primer (5′-AGATCGGAAGAGCACACGTCTGAACTCCAGTCACNNNNNNATCTCGTATGCCGTCTTCTGCTTG). Amplification was monitored on a Thermocycler (Bio-Rad) and stopped at the beginning of the saturation point to avoid over-amplification. The amplified sequencing libraries ranging from 150 to 400 bp were fractionated from a 2% agarose gel by Pippin Prep. The final amplified library was purified with a MinElute Gel Extraction Kit (Qiagen) and quantified using a Qubit 2.0 fluorometer. After validation with TOPO cloning, the sequencing libraries were sequenced using a MiSeq Reagent kit v2 in single-read mode with 150 cycles on the MiSeq platform with read lengths of 150 nucleotides.

### 5′ Rapid amplification of cDNA ends (5′ RACE)

One microgram of rRNA-depleted mRNA was ligated with 50 pmol of a short RNA adapter (5′-ACGGACUAGAAGAAA) by T4 RNA ligase (Thermo) and SUPERase-In (Thermo) at 37 °C for 90 min and then at 70 °C for 10 min. To remove excess adapters after the reaction, the sample was incubated with 40 μL of AMPureXP beads for 5 min and the supernatant was removed. The beads were washed with 70% ethanol twice and then resuspended in 22 μL of RNase-free water. The supernatants were transferred to new 1.5 mL tubes and divided into two samples for TAP treatment (+TAP) and non-treatment (−TAP). The samples were incubated with and without TAP in the presence of SUPERase-In at 37 °C for 60 min. Each sample was then ethanol precipitated and further resuspended in 13 μL of RNase-free water. The RNA sample was ligated with 10 pmol of an RNA adapter (5′-AUAUGCGCGAAUUCCUGUAGAACGAACACUAGAAGAAA) using 10 U of T4 RNA ligase and 20 U of SUPERase-In at 37 °C for 90 min and then at 70 °C for 10 min. The excess adapter removal step was performed as described above and the sample was then resuspended in 10 μL of RNase-free water. Eight microliters of the adapter-ligated RNA sample was reverse transcribed with Superscript III RT (Invitrogen) with random hexamers according to the manufacturer’s instructions. Then cDNA samples were further purified by AMPureXP beads and ethanol precipitation, and resuspended in 20 μL of RNase-free water. For amplification of specific cDNAs, targeted cDNAs were amplified with a 5′ primer (5′-GCGCGAATTCCTGTAGAACG) (25 pmol) and 3′ primer (10 pmol) specific to each gene. The amplified cDNAs were resolved with a 2.5% agarose gel and identities were further confirmed by Sanger sequencing.

### Quantitative real-time PCR

First-strand cDNAs were synthesized from total RNA using the SuperScript III First-Strand Synthesis System (Invitrogen) in accordance with the manufacturer’s instructions. Amplification of cDNAs was monitored on a CFX96 Real-Time PCR Detection System with SYBR Green I Nucleic Acid Gel Stain (Invitrogen) under the following conditions: 98 °C for 10 s; 62 °C for 30 s; and 72 °C for 10 s for 35 cycles.

### Data processing and analysis

For ssRNA-seq analysis, adapter sequences were trimmed and sequence reads shorter than 25 nucleotides were discarded. The retained sequence reads were mapped to the *T. onnurineus* NA1 (NC_011529) genome sequence using CLC genomics workbench (CLC bio, Aarhus, Denmark) with the following parameters: mismatch cost = 2, insertion cost = 3, deletion cost = 3, length fraction = 0.9, and similarity = 0.9. Only sequence reads that aligned to unique genomic locations were retained. To normalize the RNA-seq profile, read depths at every genomic position were adjusted to the value proportional to one million mapped reads. To avoid bias caused by large numbers of rRNA and tRNA genes, we omitted the reads that were mapped to rRNA and tRNA genes as described previously^59^. Differentially expressed genes were identified using the DESeq package in R^40^. For dRNA-seq analysis, the reads from two experiments were summed, whose TSS adapter sequence (5′-CAGAGTTCTACAGTCCGACGATC) and N9 sequences were trimmed and sequence reads shorter than 25 nucleotides were also removed. Genomic positions of the 5′ ends of uniquely aligned dRNA-seq reads were considered to be potential TSSs. Only TSSs present in both TEX+ and TEX− libraries (within ±5 bp) were retained. TSSs were then curated as described previously with some modifications^21^. Briefly, potential TSSs within 150 bp were clustered together; they were then sub-clustered with the standard deviation <15. Clusters and sub-clusters with less than three reads were removed. The potential TSS with the most reads in a sub-cluster was selected as the TSS. If a cluster had several TSSs, the standard deviation of two adjacent TSSs was calculated. If the calculated standard deviation was less than 15, the one with lower reads was removed. The TSSs were then manually curated and classified according to their positions. Among the TSSs located from 300 bp upstream of the respective annotated start codon of each ORF, the TSS with the maximum number of reads was classified as the primary (P) TSS and the others were termed secondary (S). In the case of the TSSs located within an annotated ORF, the TSS on the same strand as the coding sequence was classified as internal (I), while a TSS on the opposite strand was antisense (A). The TSSs not falling into any of the categories mentioned above were classified as intergenic (N). Only secondary and internal TSSs with at least 50% of the reads of the primary TSS were retained. COG functional categories were extracted from NCBI RefSeq (NC_011529.ptt) and gene associations to KEGG pathways were determined using the KEGG website (http://www.genome.jp/kegg/tool/map_pathway2.html) with NCBI GeneID. Essential genes in *T. onnurineus* were identified by the KEGG orthologs^60^ of essential genes in *E. coli*^61^. RNA-seq depth profiles and TSS profiles were visualized using SignalMap software from NimbleGen (http://www.nimblegen.com/products/software/). In-house scripts used for data processing are available on request.

### Motif discovery

The 60 bp sequences upstream of each TSS were extracted to identify the promoter motifs. The conserved sequence (AANNNTTATAA) was obtained using MEME software. We extracted sequences 7 bp upstream and 21 bp downstream sequences from the first and last nucleotides of the conserved sequence, respectively. The extracted sequences (N7-AANNNTTATAA-N21) were then used to draw the conserved motif sequences using Weblogo^62^.

### Comparative genome analysis

An all-versus-all BLASTP search was performed using the protein sets in genomes to identify pairwise matches and the E-value threshold was 1×10^−10^ with over 80% coverage. The phylogenetic tree was constructed by the neighbor-joining method using PHYLIP version 3.65, and was visualized using TREEVIEW software^63^. The distance method for the neighbor-joining algorithm was generated by a Dayhoff PAM matrix. The robustness of phylogenetic tree topologies was evaluated by bootstrap analyses of the neighbor-joining algorithms based on 1,000 resamples.

## Additional Information

**Accession codes:** RNA sequencing data were deposited in the Gene Expression Omnibus database under accession number GSE85760 and also available at http://cholab.or.kr/data/.

**How to cite this article**: Cho, S. *et al*. Genome-wide primary transcriptome analysis of H_2_-producing archaeon *Thermococcus onnurineus* NA1. *Sci. Rep.* **7**, 43044; doi: 10.1038/srep43044 (2017).

**Publisher's note:** Springer Nature remains neutral with regard to jurisdictional claims in published maps and institutional affiliations.

## Supplementary Material

Supplementary Information
