# Supplementary material for: Genome-wide primary transcriptome analysis of H2-producing archaeon Thermococcus onnurineus NA1
Source: Sci Rep. 2017 Feb 20;7:43044. doi: 10.1038/srep43044 (PMC5316973; doi:10.1038/srep43044)
Supplement: Supplementary Information [file srep43044-s1.pdf]

**Supplementary information**

# Genome-wide primary transcriptome analysis of $H_2$-producing archaeon *Thermococcus onnurineus* NA1

Suhyung Cho[1,¶,*], Min-Sik Kim[2,¶], Yujin Jeong[1,¶], Bo-Rahm Lee[3], Jung-Hyun Lee[2], Sung Gyun Kang[2], and Byung-Kwan Cho[1,3,*]

[1]Department of Biological Sciences and KI for the BioCentury, Korea Advanced Institute of Science and Technology, Daejeon 305-701, Republic of Korea

[2]Korea Institute of Ocean Science and Technology, Ansan 426-744, Republic of Korea

[3]Intelligent Synthetic Biology Center, Korea Advanced Institute of Science and Technology, Daejeon 305-701, Republic of Korea

*Correspondence and requests for materials should be addressed to S.C. (shcho95@kaist.ac.kr) or B.-K.C. (bcho@kaist.ac.kr)

[¶] These authors contributed equally to this work.

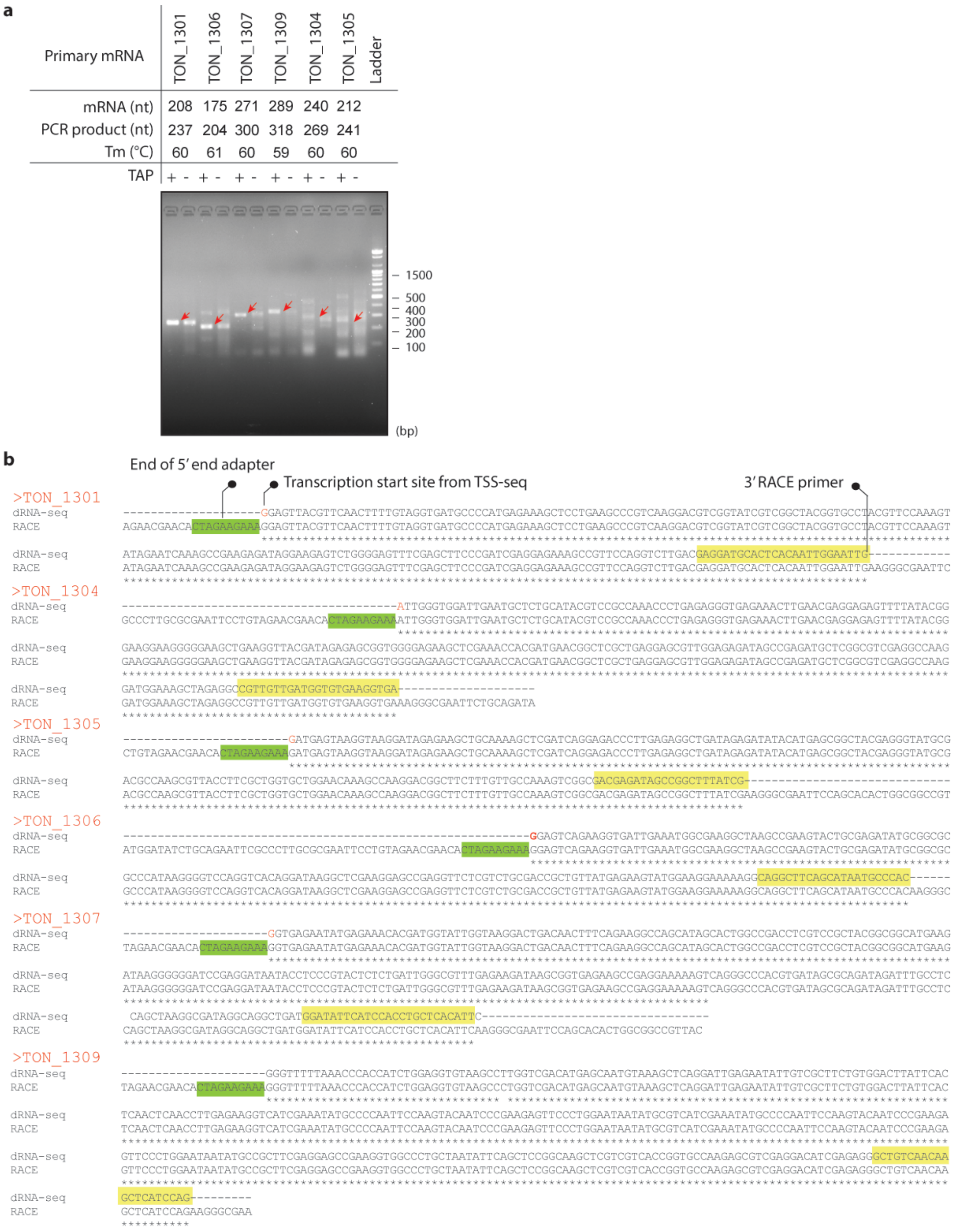


**Supplementary Fig. S1.** 5' Rapid Amplification of cDNA Ends (5'tagRACE). (**a**) The TSSs of 6 mRNAs found by dRNA-seq were confirmed using 5'tagRACE and targeted genes were separated by 2.5% agarose gel electrophoresis. (**b**) The 5' end of amplified DNA fragments was confirmed using pyrosequencing.

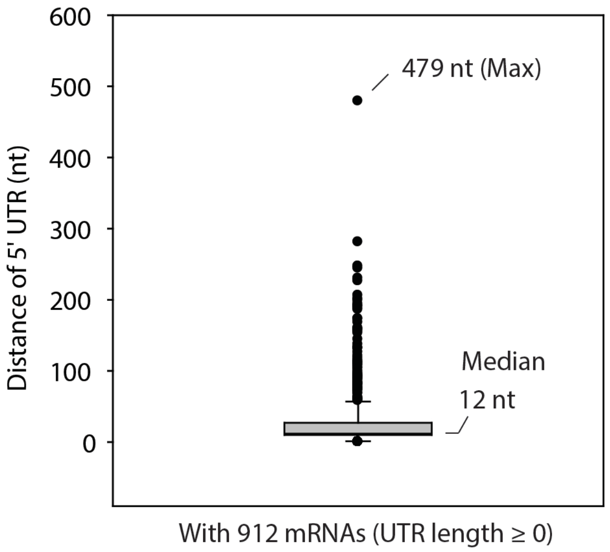


**Supplementary Fig. S2. Distribution of 5' UTR length.** The maximal and median length of 5' UTRs in *T. onnurineus* NA1.

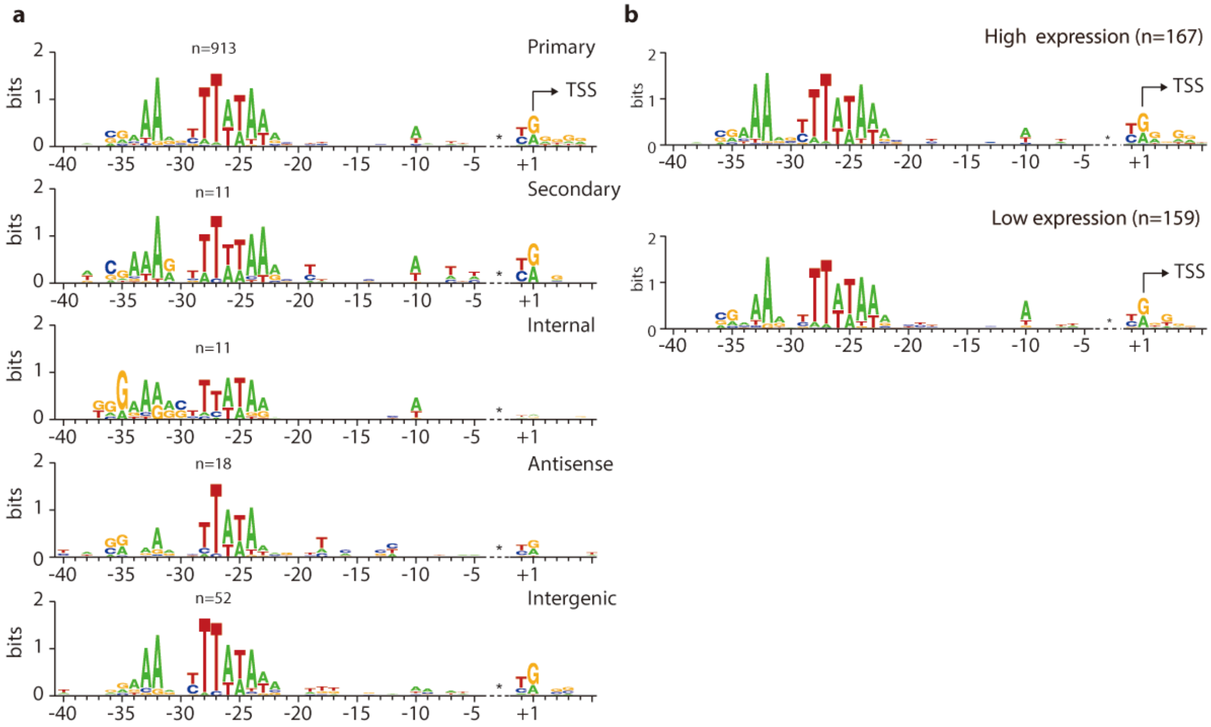


**Supplementary Fig. S3. Promoter motifs upstream of transcription start sites.** (**a**) The TATA boxes, BREs, and PPE motifs are shown between the TSS (+1) and 40 nt upstream according to primary, secondary, internal, antisense, and intergenic TSS location using MEME. (**b**) The promoter motifs according to promoter strength. The promoters and BRE motifs were compared for genes corresponding to high (high-ranked 167 genes) and low transcription levels (low-ranked 159 genes) using MEME.

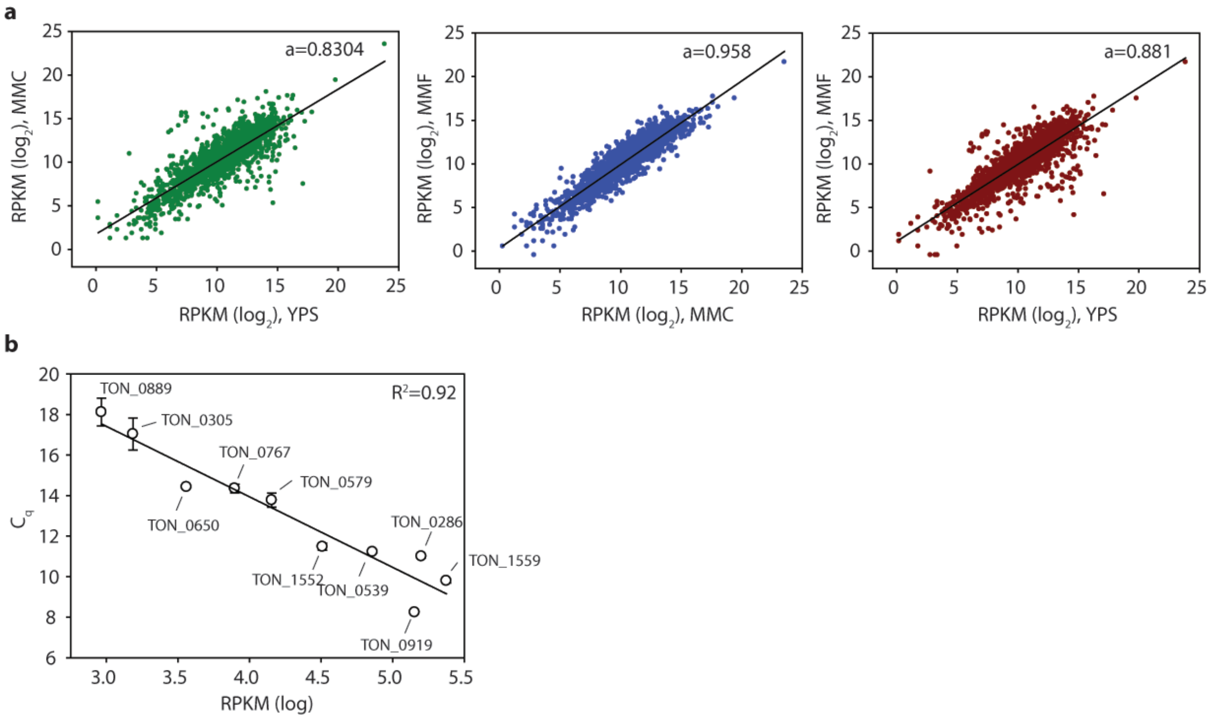


**Supplementary Fig. S4. Differential RNA expression. (a)** Pairwise correlation of RNA expression between the growth conditions of YPS/MMC, MMC/MMF, and YPS/MMF were examined and illustrated with a scatter plot. (**b**) Quantitative analysis of cellular mRNAs by qRT-PCR. The correlation between $C_q$ value from qPCR with RPKM from ssRNA-seq was examined for 10 specific mRNAs.

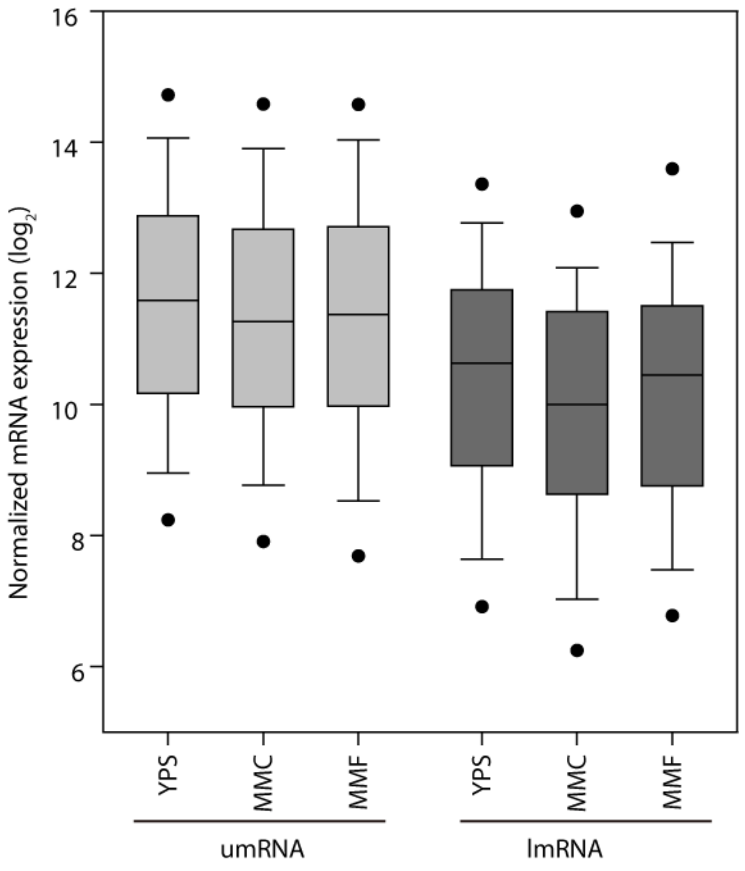


**Supplementary Fig. S5. The box plot of normalized mRNA expression according to the culture media, YPS, MMC, and MMF in leadered mRNA and leaderless mRNA.**

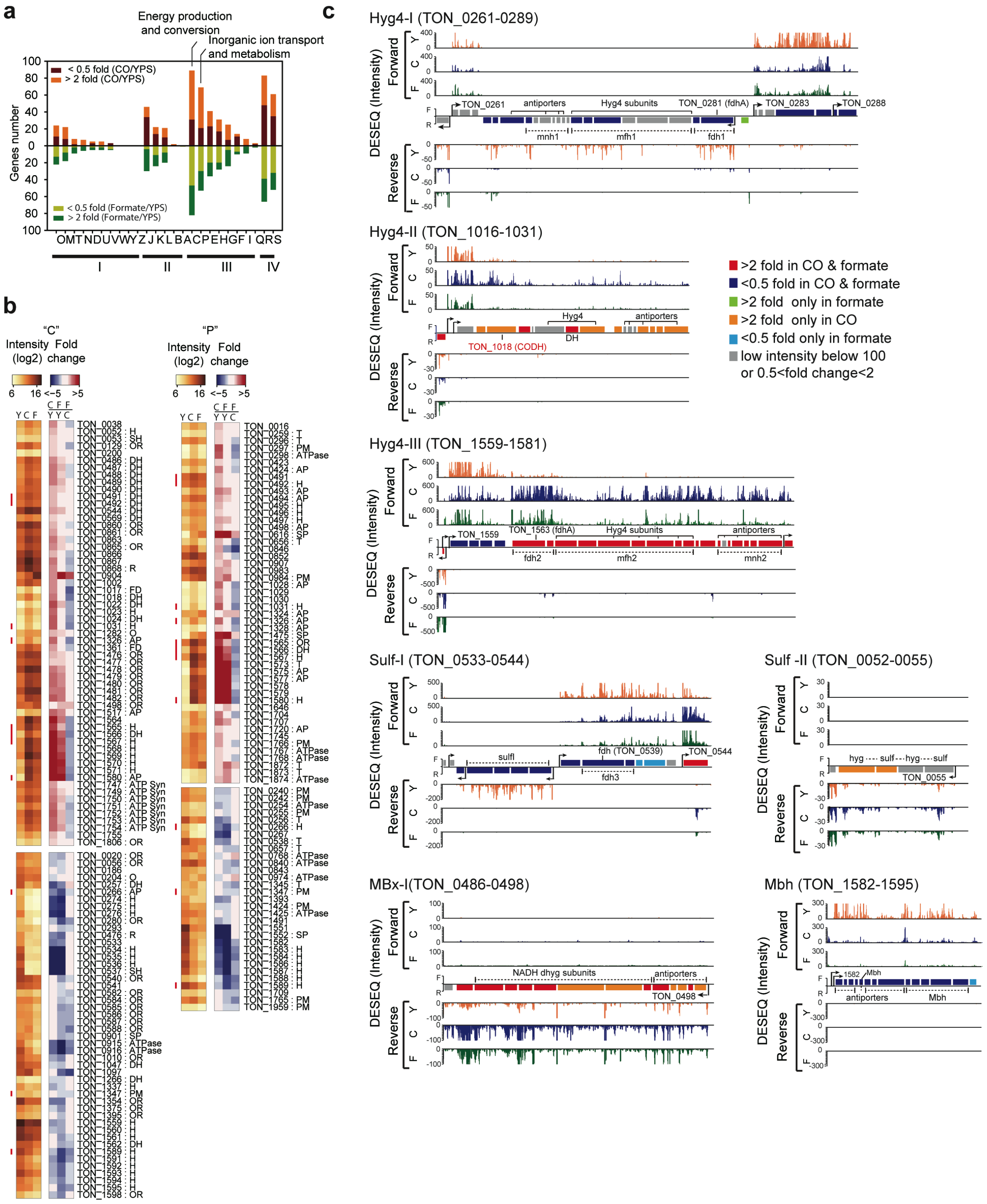

a
Energy production and conversion
Inorganic ion transport and metabolism
< 0.5 fold (CO/YPS)
> 2 fold (CO/YPS)
< 0.5 fold (Formate/YPS)
> 2 fold (Formate/YPS)
Genes number
OMTNDUVWYZJKLBACPEHGFIQRS
I
II
III
IV
b
"C"
Intensity (log2)
Fold change
"P"
6 16 <-5 >5
c
Hyg4-I (TON_0261-0289)
Hyg4-II (TON_1016-1031)
Hyg4-III (TON_1559-1581)
Sulf-I (TON_0533-0544)
Sulf -II (TON_0052-0055)
MBx-I(TON_0486-0498)
Mbh (TON_1582-1595)
DESEQ (Intensity)
Forward
Reverse
TON_0261
antiporters
Hyg4 subunits
TON_0281 (fdhA)
TON_0283
TON_0288
mnh1
mfh1
fdh1
TON_1018 (CODH)
DH
TON_1559
TON_1563 (fdhA)
fdh2
mfh2
mnh2
sulfI
fdh (TON_0539)
TON_0544
fdh3
hyg ---- sulf---hyg----sulf
TON_0055
NADH dhyg subunits
TON_0498
1582
Mbh
>2 fold in CO & formate
<0.5 fold in CO & formate
>2 fold only in formate
>2 fold only in CO
<0.5 fold only in formate
low intensity below 100 or 0.5<fold change<2

**Supplementary Fig. S6. Differential gene expression.** (**a**) The functional groups showing differential expression over 2-fold in MMC and MMF against YPS were categorized as I, II, III, and IV (cellular processes and signaling, information storage and processing, metabolism, and poorly characterized) by COG mapping, and further divided into subgroups of each functional group. Significant changes in gene expression were observed in diverse functions including energy production and inorganic ion transport/metabolism as shown in the COG mapping. (**b**) The changes in expression profiles of genes in various growth media of group C and group P, which were highly activated in (b) were displayed using a heat map with protein function. The left red bar in the heat map indicates the common genes involved in both groups C (energy production and conversion) and P (inorganic ion transport and metabolism). Abbreviations: H, hydrogenase; OR, oxidoreductase; DH, dehydrogenase; R, reductase; FD, ferredoxin; SH, Sulfhydrogenase; O, oxidase; AP, antiporter; PM, permease; T, transporter; SP, symporter. (**c**) the profile feature by ssRNA-seq. The hydrogenase 4 in three Hyg4 clusters showed differential gene expression in different growth conditions. In Hyg4-I, mfh1 with Hyg4 subunits, fdh1, and mnh1 with antiporters clusters were downregulated in CO and formate conditions. In contrast, all fdh2, mfh2, and mnh2 in Hgy4-III exhibited expression > 2-fold greater in CO and formate conditions but were scarcely expressed in YPS media. The Hyg4-II cluster was expressed only in the presence of CO, while not expressed in formate and YPS conditions. Especially, CO-responsive hydrogenase was substantially activated, and the catalytic subunit of CO dehydrogenase (TON_1018), a key CO metabolic enzyme, was significantly increased only in CO media. In addition, there are two clusters with sulfhydrogenase and cytosol hydrogenase, Sulf-I and Sulf-II . Their RNA expression was not occurred in the CO and formate media without sulfur but all genes in a Sulf-I cluster were fully activated > 2-fold by sulfur in YPS rich media. Especially, Sulf-I cluster was together with fdh cluster in different operon but at just near Sulf-I cluster. Moreover, fdh3 part was also highly expressed in all conditions but sulfI part activated by sulfur was totally deactivated in all conditions. Sulf-II consists of simple repeated Hyg and Sulf subunits, but entire expression level in all media was very low (RPKM < 30). Membrane bound oxidoreductase (MBx-I) subunits composing of NADH dependent dehydrogenase and antiporters were mostly activated > 2-fold in CO and formate against YPS. In contrast, membrane bound hydrogenase (Mbh) clusters with antiporters were poorly activated in CO and formate media for YPS. Taken together, high hydrogen production in CO and formate media seems to be driven by high expression of Hyg4-II and Hyg4-III clusters with the reduction of transcription of the other hydrogenase clusters.

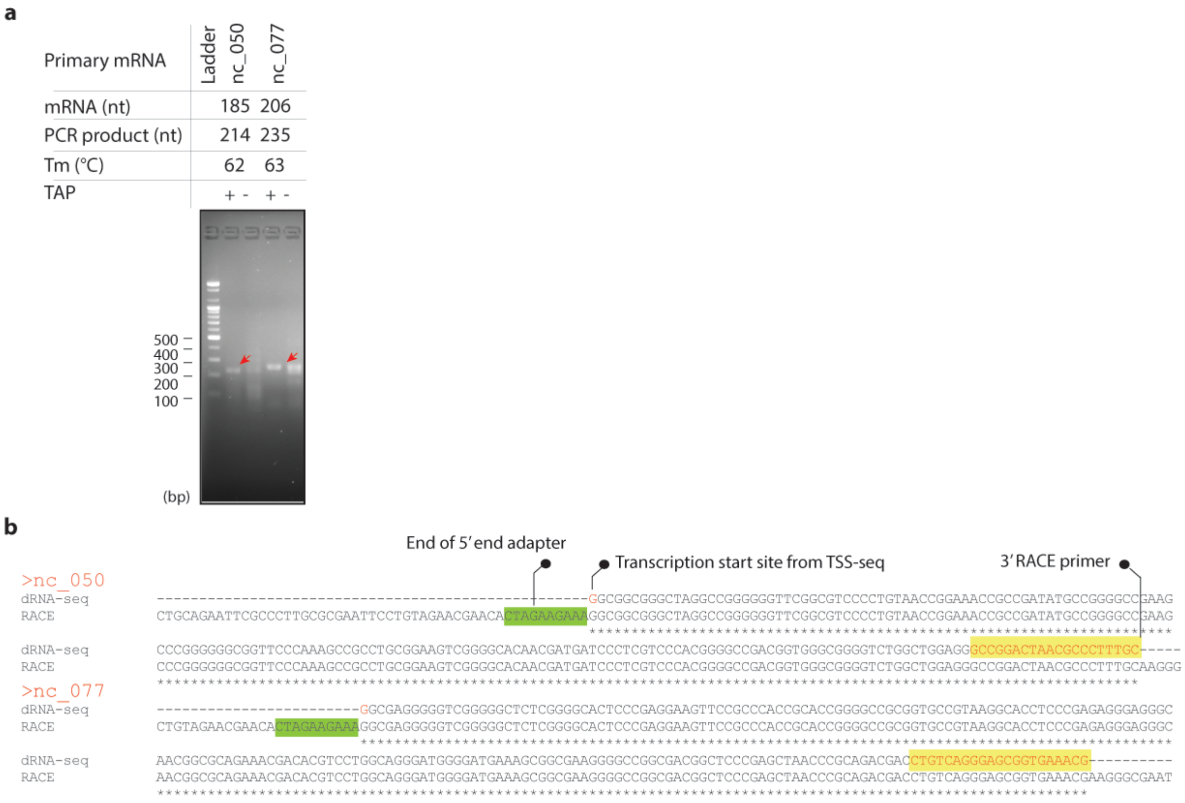


**Supplementary Fig. S7. TSS determination of ncRNAs by 5' Rapid Amplification of cDNA Ends (5'RACE).** (**a**) The TSSs of nc_050 and nc_077 corresponding to archaeal signal recognition particle RNA and Archaeal RNAse P, respectively, were newly found by dRNA-seq and their DNA sizes were confirmed using agarose gel electrophoresis (2.5%). (**b**) The DNA sequence was determined by Sanger sequencing.

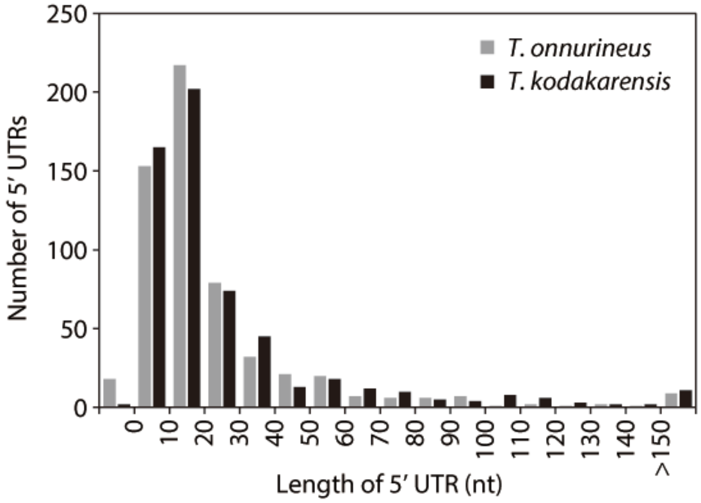


**Supplementary Fig. S8**. The distribution of 5' UTR length of *T. onnurineus* NA1 and *T. kodakarensis* orthologous genes.

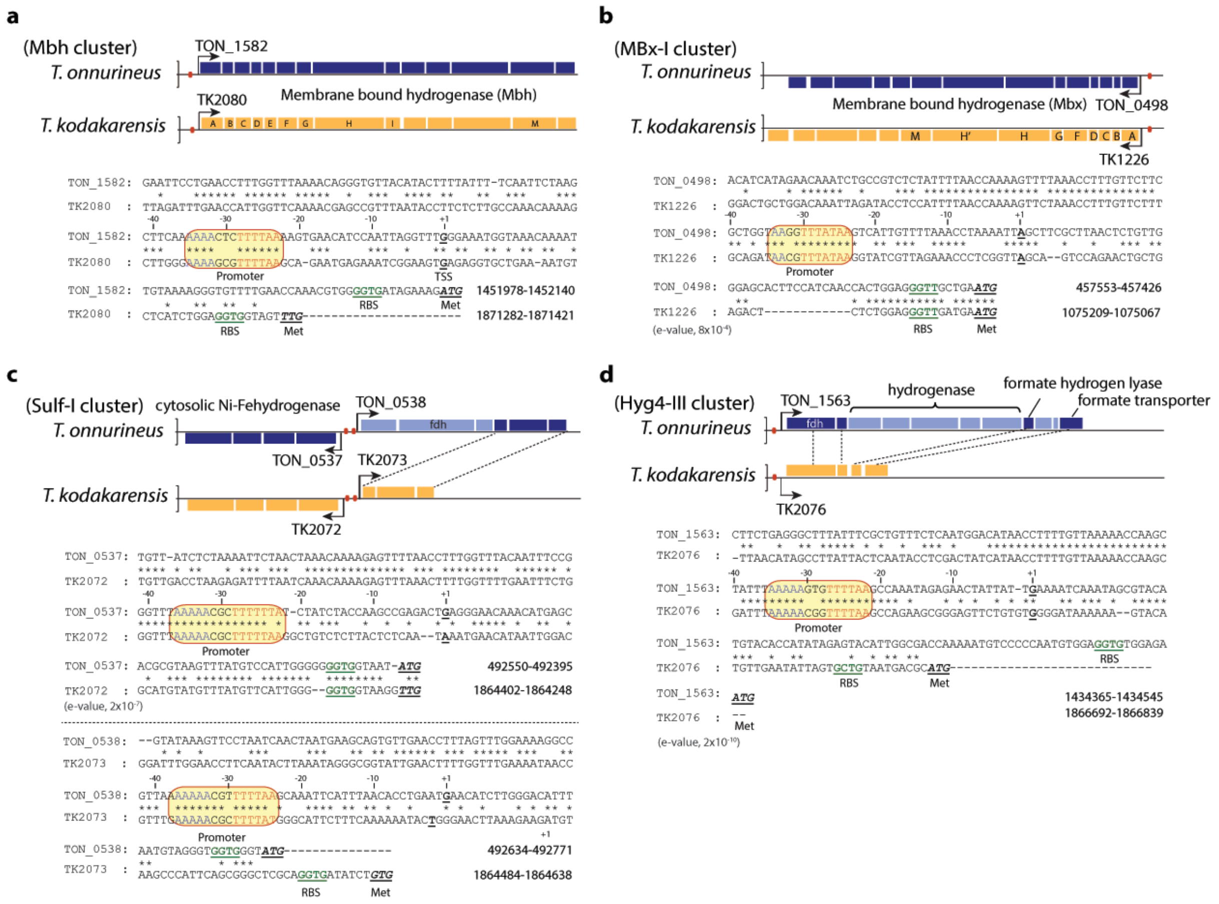


**Supplementary Fig. S9. The promoter, its regulatory upstream DNA sequence, and 5' UTR between orthologous genes in *T. onnurineus* and *T. kodakarensis*.** Among Mbh, Mbx-I, and Sulf-I, and Hyg4-III clusters, the upstream sequences of orthologous genes were each aligned and compared. Their promoters, RBSs, and start codons were analyzed together.

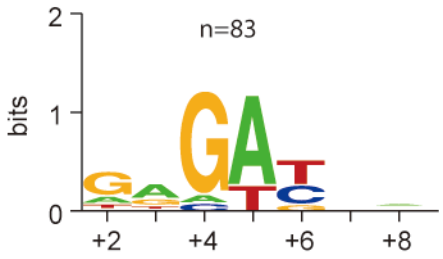


**Supplementary Fig. S10. The INR motif in 5' UTRs of *T. onnurineus* NA1.** The initiator element (Inr) motif was searched from positions +2–+8 downstream of TSSs in 328 leadered RNAs which have a 5' UTR length > 19 nt. In result, 83 RNAs showed a conserved GAGAT motif (p-val < 0.05; MEME).

**Supplementary Table 1**. NGS Sequencing results

| | dRNA-seq | | ssRNA-seq | | |
|---|---|---|---|---|---|
| | **TEX+** | **TEX-** | **YPS** | **CO** | **Formate** |
| Total reads | 4,150,751 | 7,754,462 | 24,032,700 | 21,475,352 | 18,657,279 |
| Total mapped reads | 1,449,816 | 4,860,704 | 23,186,484 | 19,664,249 | 17,750,691 |
| Uniquely mapped reads | 1,448,204 | 4,857,961 | 23,127,009 | 19,634,732 | 17,703,832 |
| % of mapped reads | 34.89% | 62.65% | 96.23% | 91.43% | 94.89% |
| Average length | 103.62 | 112.12 | 35.75 | 35.93 | 35.9 |
| Number of bases | 150,062,898 | 544,674,587 | 826,846,321 | 705,405,046 | 635,582,990 |

**Supplementary Table 2.** Determination of transcription units architecture

| TU | TSS | Strand | Abundance | Category | Current Annotation Assembly | operon start | operon end | Gene start | Gene end | 5'-UTR-length |
|---|---|---|---|---|---|---|---|---|---|---|
| TU0001 | 4783 | - | -3 | P | TON_0002 | TON_0002 | TON_0002 | 4774 | 4079 | 9 |
| TU0002 | 6374 | - | -5558 | P | TON_0004; TON_0003 | TON_0004 | TON_0003 | 6473 | 4815 | -99 |
| TU0003 | 6426 | + | 69 | P | TON_0005 | TON_0005 | TON_0005 | 6437 | 7906 | 11 |
| TU0004 | 8069 | + | 25 | P | TON_0006 | TON_0006 | TON_0006 | 8069 | 9247 | 0 |
| TU0005 | 9257 | + | 44 | P | TON_0007 | TON_0007 | TON_0007 | 9286 | 11511 | 29 |
| TU0006 | 11555 | + | 122 | P | TON_0008 | TON_0008 | TON_0008 | 11580 | 11834 | 25 |
| TU0007 | 12474 | + | 5 | P | TON_0009 | TON_0009 | TON_0009 | 12484 | 12699 | 10 |
| TU0008 | 14671 | - | -24 | P | TON_0012 | TON_0012 | TON_0012 | 14661 | 14275 | 10 |
| TU0009 | 14744 | + | 241 | P | TON_0013 | TON_0013 | TON_0013 | 14754 | 15152 | 10 |
| TU0010 | 15233 | + | 1 | P | TON_0014 | TON_0014 | TON_0014 | 15273 | 16313 | 40 |
| TU0011 | 16345 | + | 1 | P | TON_0015; TON_0016 | TON_0015 | TON_0016 | 16353 | 17779 | 8 |
| TU0012 | 19367 | - | -28 | P | TON_0017 | TON_0017 | TON_0017 | 19359 | 18313 | 8 |
| TU0013 | 19403 | + | 24 | P | TON_0018 | TON_0018 | TON_0018 | 19415 | 19738 | 12 |
| TU0014 | 23264 | - | -1 | P | TON_0020 | TON_0020 | TON_0020 | 23269 | 21353 | -5 |
| TU0015 | 23885 | - | -8 | P | TON_0022 | TON_0022 | TON_0022 | 23875 | 23624 | 10 |
| TU0016 | 23960 | + | 496 | P | TON_0023 | TON_0023 | TON_0023 | 23969 | 24256 | 9 |
| TU0017 | 25032 | - | -990 | P | TON_0025 | TON_0025 | TON_0025 | 25022 | 24573 | 10 |
| TU0018 | 25333 | + | 3 | P | TON_0027; TON_0028; TON_0029; TON_0030; TON_0031 | TON_0027 | TON_0031 | 25378 | 29206 | 45 |
| TU0019 | 25830 | + | 10 | I | TON_0027; TON_0028; TON_0029; TON_0030; TON_0031 | TON_0027 | TON_0031 | 25378 | 29206 | - |
| TU0020 | 29564 | + | 277 | P | TON_0033 | TON_0033 | TON_0033 | 29596 | 29994 | 32 |
| TU0021 | 31229 | - | -83 | P | TON_0035 | TON_0035 | TON_0035 | 31220 | 30696 | 9 |
| TU0022 | 33167 | - | -23 | P | TON_0037 | TON_0037 | TON_0037 | 33151 | 32309 | 16 |
| TU0023 | 36831 | - | -1139 | P | TON_0041; TON_0040; TON_0039; TON_0038 | TON_0041 | TON_0038 | 36801 | 33235 | 30 |
| TU0024 | 36882 | + | 200 | N | TON_nc001 | TON_nc001 | TON_nc001 | - | - | - |
| TU0025 | 37394 | - | -801 | P | TON_0042 | TON_0042 | TON_0042 | 37382 | 36951 | 12 |
| TU0026 | 41339 | - | -6 | I | TON_0044; TON_0043 | TON_0044 | TON_0043 | 41418 | 37451 | - |
| TU0027 | 41434 | - | -7 | P | TON_0044; TON_0043 | TON_0044 | TON_0043 | 41418 | 37451 | 16 |
| TU0028 | 41518 | + | 153 | P | TON_0045 | TON_0045 | TON_0045 | 41527 | 42039 | 9 |
| TU0029 | 43552 | - | -203 | P | TON_0047 | TON_0047 | TON_0047 | 43543 | 42953 | 9 |

| | | | | | | | | | | |
|---|---|---|---|---|---|---|---|---|---|---|
| TU0030 | 44713 | + | 2309 | P | TON_0049 | TON_0049 | TON_0049 | 44714 | 45553 | 1 |
| TU0031 | 46757 | - | -1 | P | TON_0051 | TON_0051 | TON_0051 | 46742 | 46419 | 15 |
| TU0032 | 50678 | - | -11 | P | TON_0055; TON_0054; TON_0053; TON_0052 | TON_0055 | TON_0052 | 50648 | 46791 | 30 |
| TU0033 | 50789 | + | 3 | P | TON_0056; TON_0057 | TON_0056 | TON_0057 | 50799 | 53059 | 10 |
| TU0034 | 54045 | - | -15 | P | TON_0058 | TON_0058 | TON_0058 | 54034 | 53132 | 11 |
| TU0035 | 54224 | + | 67 | P | TON_0059 | TON_0059 | TON_0059 | 54235 | 54828 | 11 |
| TU0036 | 55604 | - | -166 | P | TON_0060 | TON_0060 | TON_0060 | 55592 | 54831 | 12 |
| TU0037 | 56789 | - | -584 | P | TON_0061 | TON_0061 | TON_0061 | 56777 | 55623 | 12 |
| TU0038 | 58117 | + | 955 | P | TON_0063 | TON_0063 | TON_0063 | 58197 | 58745 | 80 |
| TU0039 | 58954 | + | 5 | P | TON_0064 | TON_0064 | TON_0064 | 58963 | 60105 | 9 |
| TU0040 | 60514 | + | 32 | P | TON_0065; TON_0066; TON_0067; TON_0068; TON_0069; TON_0070; TON_0071; TON_0072; TON_0073; TON_0074; TON_0075; TON_0076; TON_0077; TON_0078; TON_0079; TON_0080; TON_0081; TON_0082; TON_0083; TON_0084; TON_0085; | TON_0065 | TON_0089 | 60403 | 74583 | -111 |
| TU0041 | 74636 | + | 5262 | P | TON_0090; TON_0091 | TON_0090 | TON_0091 | 74659 | 75769 | 23 |
| TU0042 | 75181 | + | 8 | P | TON_0091 | TON_0091 | TON_0091 | 75242 | 75769 | 61 |
| TU0043 | 75800 | + | 326 | P | TON_0092 | TON_0092 | TON_0092 | 75812 | 76084 | 12 |
| TU0044 | 77011 | + | 21 | P | TON_0095 | TON_0095 | TON_0095 | 77020 | 77727 | 9 |
| TU0045 | 77128 | + | 28 | I | TON_0095 | TON_0095 | TON_0095 | 77020 | 77727 | - |
| TU0046 | 77764 | + | 48 | P | TON_0096 | TON_0096 | TON_0096 | 77788 | 78792 | 24 |
| TU0047 | 78016 | + | 12 | I | TON_0096 | TON_0096 | TON_0096 | 77788 | 78792 | - |
| TU0048 | 79015 | + | 1 | P | TON_0097; TON_0098 | TON_0097 | TON_0098 | 79038 | 80839 | 23 |
| TU0049 | 81023 | + | 62 | N | TON_nc002 | TON_nc002 | TON_nc002 | - | - | - |
| TU0050 | 81382 | + | 1 | P | TON_0099 | TON_0099 | TON_0099 | 81381 | 81683 | -1 |
| TU0051 | 82522 | + | 27 | P | TON_0101; TON_1984 | TON_0101 | TON_1984 | 82480 | 83181 | -42 |
| TU0052 | 83221 | + | 5 | P | TON_0102; TON_0103; TON_0104 | TON_0102 | TON_0104 | 83221 | 84639 | 0 |
| TU0053 | 85449 | + | 201 | P | TON_1985 | TON_1985 | TON_1985 | 85452 | 85539 | - |
| TU0054 | 86364 | + | 5 | P | TON_0108 | TON_0108 | TON_0108 | 86402 | 86809 | 38 |
| TU0055 | 86538 | + | 10 | I | TON_0108 | TON_0108 | TON_0108 | 86402 | 86809 | - |
| TU0056 | 87141 | + | 4 | P | TON_0110 | TON_0110 | TON_0110 | 87156 | 87329 | 15 |

| | | | | | | | | | | |
|---|---|---|---|---|---|---|---|---|---|---|
| TU0057 | 89143 | + | 66 | P | TON_0113 | TON_0113 | TON_0113 | 89168 | 89326 | 25 |
| TU0058 | 89778 | - | -3 | P | TON_0114 | TON_0114 | TON_0114 | 89751 | 89425 | 27 |
| TU0059 | 89866 | + | 60 | P | TON_0115 | TON_0115 | TON_0115 | 89876 | 90175 | 10 |
| TU0060 | 90200 | + | 285 | P | TON_0116 | TON_0116 | TON_0116 | 90200 | 91522 | 0 |
| TU0061 | 92279 | - | -1422 | P | TON_0117 | TON_0117 | TON_0117 | 92255 | 91494 | 24 |
| TU0062 | 92750 | - | -8 | P | TON_1982 | TON_1982 | TON_1982 | 92743 | 92666 | - |
| TU0063 | 92801 | + | 488 | P | TON_0118 | TON_0118 | TON_0118 | 92811 | 93263 | 10 |
| TU0064 | 94312 | - | -561 | P | TON_0120; TON_0119 | TON_0120 | TON_0119 | 94297 | 93249 | 15 |
| TU0065 | 96450 | - | -123 | P | TON_0123; TON_0122; TON_0121 | TON_0123 | TON_0121 | 96439 | 94352 | 11 |
| TU0066 | 96684 | + | 36 | P | TON_0124 | TON_0124 | TON_0124 | 96710 | 96817 | 26 |
| TU0067 | 97786 | + | 3210 | P | TON_0126 | TON_0126 | TON_0126 | 97796 | 98005 | 10 |
| TU0068 | 98088 | + | 3 | P | TON_0127 | TON_0127 | TON_0127 | 98098 | 99399 | 10 |
| TU0069 | 99430 | + | 393 | P | TON_0128 | TON_0128 | TON_0128 | 99440 | 100243 | 10 |
| TU0070 | 100344 | + | 6 | P | TON_0129 | TON_0129 | TON_0129 | 100367 | 101704 | 23 |
| TU0071 | 102034 | + | 6015 | P | TON_1988 | TON_1988 | TON_1988 | 102041 | 102118 | - |
| TU0072 | 102892 | - | -19434 | P | TON_0130 | TON_0130 | TON_0130 | 102877 | 102152 | 15 |
| TU0073 | 103961 | - | -20 | P | TON_0131 | TON_0131 | TON_0131 | 103939 | 103106 | 22 |
| TU0074 | 104039 | + | 46 | P | TON_0132 | TON_0132 | TON_0132 | 104050 | 104925 | 11 |
| TU0075 | 104974 | + | 32 | P | TON_0133; TON_0134 | TON_0133 | TON_0134 | 104986 | 106763 | 12 |
| TU0076 | 106811 | + | 164 | P | TON_0135 | TON_0135 | TON_0135 | 106821 | 107945 | 10 |
| TU0077 | 108044 | + | 7 | P | TON_0136 | TON_0136 | TON_0136 | 108093 | 109415 | 49 |
| TU0078 | 112251 | + | 6 | N | TON_nc003 | TON_nc003 | TON_nc003 | - | - | - |
| TU0079 | 112601 | + | 102 | N | TON_nc004 | TON_nc004 | TON_nc004 | - | - | - |
| TU0080 | 116703 | - | -74 | P | TON_0141 | TON_0141 | TON_0141 | 116577 | 113674 | 126 |
| TU0081 | 120042 | - | -28 | P | TON_0143 | TON_0143 | TON_0143 | 120032 | 118962 | 10 |
| TU0082 | 120091 | + | 4545 | P | TON_0144 | TON_0144 | TON_0144 | 120160 | 120744 | 69 |
| TU0083 | 120827 | + | 4 | P | TON_0145; TON_0146 | TON_0145 | TON_0146 | 120838 | 121961 | 11 |
| TU0084 | 123536 | - | -320 | P | TON_0147 | TON_0147 | TON_0147 | 123289 | 121958 | 247 |
| TU0085 | 123853 | + | 33 | P | TON_0148 | TON_0148 | TON_0148 | 123861 | 124868 | 8 |
| TU0086 | 125311 | - | -281 | P | TON_0149 | TON_0149 | TON_0149 | 125311 | 124865 | 0 |
| TU0087 | 125384 | + | 61 | P | TON_0150; TON_0151 | TON_0150 | TON_0151 | 125393 | 126245 | 9 |
| TU0088 | 128201 | - | -12 | P | TON_0152 | TON_0152 | TON_0152 | 128191 | 126242 | 10 |

| | | | | | | | | | | |
|---|---|---|---|---|---|---|---|---|---|---|
| TU0089 | 130359 | + | 159 | P | TON_0154 | TON_0154 | TON_0154 | 130368 | 130685 | 9 |
| TU0090 | 133209 | - | -73 | P | TON_0156 | TON_0156 | TON_0156 | 133150 | 131567 | 59 |
| TU0091 | 133450 | + | 737 | N | TON_nc005 | TON_nc005 | TON_nc005 | - | - | - |
| TU0092 | 133733 | + | 269 | P | TON_0157 | TON_0157 | TON_0157 | 133762 | 135021 | 29 |
| TU0093 | 141395 | - | -224 | P | TON_0163 | TON_0163 | TON_0163 | 141382 | 140219 | 13 |
| TU0094 | 141446 | + | 415 | P | TON_0164; TON_0165; TON_0166 | TON_0164 | TON_0166 | 141492 | 145400 | 46 |
| TU0095 | 145438 | + | 83 | P | TON_0167; TON_0168 | TON_0167 | TON_0168 | 145449 | 146481 | 11 |
| TU0096 | 147190 | - | -8 | I | TON_0169 | TON_0169 | TON_0169 | 147436 | 146837 | - |
| TU0097 | 147445 | - | -14 | P | TON_0169 | TON_0169 | TON_0169 | 147436 | 146837 | 9 |
| TU0098 | 147479 | + | 116 | P | TON_0170 | TON_0170 | TON_0170 | 147490 | 148698 | 11 |
| TU0099 | 148783 | + | 1553 | P | TON_0171 | TON_0171 | TON_0171 | 148806 | 148976 | 23 |
| TU0100 | 152846 | - | -1 | P | TON_0174; TON_0173; TON_0172 | TON_0174 | TON_0172 | 152758 | 148982 | 88 |
| TU0101 | 152972 | + | 5 | P | TON_0175 | TON_0175 | TON_0175 | 152983 | 153801 | 11 |
| TU0102 | 153843 | + | 252 | P | TON_0176 | TON_0176 | TON_0176 | 153895 | 155043 | 52 |
| TU0103 | 154876 | + | 15 | P | TON_0177 | TON_0177 | TON_0177 | 155082 | 155267 | 206 |
| TU0104 | 156305 | + | 400 | P | TON_0180; TON_0181 | TON_0180 | TON_0181 | 156347 | 158022 | 42 |
| TU0105 | 158897 | - | -44 | P | TON_0183 | TON_0183 | TON_0183 | 158914 | 158447 | -17 |
| TU0106 | 159404 | - | -95 | N | TON_nc006 | TON_nc006 | TON_nc006 | - | - | - |
| TU0107 | 160465 | + | 40 | P | TON_0185 | TON_0185 | TON_0185 | 160492 | 160695 | 27 |
| TU0108 | 160762 | + | 26 | P | TON_0186 | TON_0186 | TON_0186 | 160762 | 162036 | 0 |
| TU0109 | 162102 | + | 301 | P | TON_0187; TON_0188 | TON_0187 | TON_0188 | 162111 | 163903 | 9 |
| TU0110 | 164178 | + | 111 | P | TON_0189 | TON_0189 | TON_0189 | 164188 | 165909 | 10 |
| TU0111 | 167269 | - | -13 | P | TON_0190 | TON_0190 | TON_0190 | 167247 | 165961 | 22 |
| TU0112 | 169912 | - | -18 | P | TON_0191 | TON_0191 | TON_0191 | 169885 | 167384 | 27 |
| TU0113 | 171388 | + | 17 | A | TON_nc007 | TON_nc007 | TON_nc007 | - | - | - |
| TU0114 | 171885 | - | -45 | P | TON_0192 | TON_0192 | TON_0192 | 171873 | 169996 | 12 |
| TU0115 | 172560 | + | 1099 | P | TON_0194 | TON_0194 | TON_0194 | 172558 | 172758 | -2 |
| TU0116 | 172769 | + | 55 | P | TON_0195 | TON_0195 | TON_0195 | 172769 | 173476 | 0 |
| TU0117 | 173557 | + | 5 | P | TON_0196 | TON_0196 | TON_0196 | 173585 | 174202 | 28 |
| TU0118 | 174325 | + | 374 | N | TON_nc008 | TON_nc008 | TON_nc008 | - | - | - |
| TU0119 | 176634 | - | -2 | P | TON_0198; TON_0197 | TON_0198 | TON_0197 | 176613 | 174424 | 21 |
| TU0120 | 178110 | + | 3 | P | TON_0200; TON_0201 | TON_0200 | TON_0201 | 178120 | 179590 | 10 |

| | | | | | | | | | | |
|---|---|---|---|---|---|---|---|---|---|---|
| TU0121 | 181316 | + | 1046 | P | TON_0203; TON_0204; TON_0205 | TON_0203 | TON_0205 | 181334 | 184424 | 18 |
| TU0122 | 185851 | - | -11 | P | TON_0208; TON_0207 | TON_0208 | TON_0207 | 185838 | 184932 | 13 |
| TU0123 | 186637 | - | -25 | P | TON_0209 | TON_0209 | TON_0209 | 186637 | 185957 | 0 |
| TU0124 | 186718 | + | 66 | P | TON_0210 | TON_0210 | TON_0210 | 186729 | 187007 | 11 |
| TU0125 | 189274 | + | 10 | P | TON_0213; TON_0214 | TON_0213 | TON_0214 | 189304 | 192153 | 30 |
| TU0126 | 192256 | + | 4 | P | TON_0215 | TON_0215 | TON_0215 | 192267 | 192575 | 11 |
| TU0127 | 205107 | - | -42 | P | TON_0224 | TON_0224 | TON_0224 | 205084 | 203960 | 23 |
| TU0128 | 205720 | - | -1341 | P | TON_0226; TON_0225 | TON_0226 | TON_0225 | 205622 | 205090 | 98 |
| TU0129 | 206955 | - | -18 | P | TON_0227 | TON_0227 | TON_0227 | 206954 | 205689 | 1 |
| TU0130 | 207172 | + | 16 | P | TON_1993 | TON_1993 | TON_1993 | 207172 | 207246 | - |
| TU0131 | 207505 | + | 41 | P | TON_0228 | TON_0228 | TON_0228 | 207515 | 207718 | 10 |
| TU0132 | 209468 | - | -9 | A | TON_nc009 | TON_nc009 | TON_nc009 | - | - | - |
| TU0133 | 211496 | - | -82 | P | TON_0231 | TON_0231 | TON_0231 | 211486 | 210287 | 10 |
| TU0134 | 211611 | + | 247 | N | TON_nc010 | TON_nc010 | TON_nc010 | 211715 | 212541 | - |
| TU0135 | 212580 | + | 6 | P | TON_0234 | TON_0234 | TON_0234 | 212603 | 212782 | 23 |
| TU0136 | 212920 | + | 1490 | P | TON_0235; TON_0236 | TON_0235 | TON_0236 | 213001 | 213486 | 81 |
| TU0137 | 213555 | + | 151 | P | TON_0237; TON_0238; TON_0239 | TON_0237 | TON_0239 | 213583 | 215403 | 28 |
| TU0138 | 215503 | + | 34 | P | TON_0240; TON_0241; TON_0242; TON_0243 | TON_0240 | TON_0243 | 215538 | 217855 | 35 |
| TU0139 | 220620 | - | -87 | P | TON_0245; TON_0244 | TON_0245 | TON_0244 | 220596 | 217845 | 24 |
| TU0140 | 222046 | - | -1 | P | TON_0246 | TON_0246 | TON_0246 | 222033 | 220672 | 13 |
| TU0141 | 223526 | - | -214 | P | TON_0249; TON_0248 | TON_0249 | TON_0248 | 223515 | 222743 | 11 |
| TU0142 | 223751 | - | -2229 | P | TON_0250 | TON_0250 | TON_0250 | 223750 | 223565 | 1 |
| TU0143 | 225380 | - | -11 | P | TON_0252; TON_0251; TON_0250 | TON_0252 | TON_0250 | 225380 | 223565 | 0 |
| TU0144 | 225456 | + | 4027 | P | TON_0253 | TON_0253 | TON_0253 | 225469 | 226428 | 13 |
| TU0145 | 230054 | - | -11 | P | TON_0257; TON_0256; TON_0255; TON_0254 | TON_0257 | TON_0254 | 230045 | 226396 | 9 |
| TU0146 | 230156 | + | 944 | P | TON_0258 | TON_0258 | TON_0258 | 230165 | 230539 | 9 |
| TU0147 | 232854 | - | -8 | P | TON_0260 | TON_0260 | TON_0260 | 232845 | 231958 | 9 |
| TU0148 | 232988 | + | 176 | P | TON_0261; TON_0262 | TON_0261 | TON_0262 | 232997 | 234159 | 9 |
| TU0149 | 250426 | - | -38 | P | TON_0281; TON_0280; TON_0279; TON_0278; TON_0277; TON_0276; TON_0275; TON_0274; TON_0273; TON_0272; TON_0271; TON_0270; TON_0269; TON_0268; TON_0267; | TON_0281 | TON_0264 | 250391 | 234875 | 35 |

| | | | | | | | | | | |
|---|---|---|---|---|---|---|---|---|---|---|
| TU0150 | 251610 | + | 76 | P | TON_0283; TON_0284; TON_0285; TON_0286; TON_0287 | TON_0283 | TON_0287 | 251621 | 256432 | 11 |
| TU0151 | 256502 | + | 62 | P | TON_0288; TON_0289 | TON_0288 | TON_0289 | 256511 | 258000 | 9 |
| TU0152 | 262074 | - | -37 | P | TON_0295; TON_0294; TON_0293 | TON_0295 | TON_0293 | 261880 | 260693 | 194 |
| TU0153 | 266909 | + | 2 | P | TON_0301; TON_0302; TON_0303 | TON_0301 | TON_0303 | 266920 | 269317 | 11 |
| TU0154 | 267051 | + | 5 | I | TON_0301; TON_0302; TON_0303 | TON_0301 | TON_0303 | 266920 | 269317 | - |
| TU0155 | 272031 | - | -18 | P | TON_0305 | TON_0305 | TON_0305 | 272021 | 270696 | 10 |
| TU0156 | 273258 | - | -39 | P | TON_0306 | TON_0306 | TON_0306 | 273247 | 272081 | 11 |
| TU0157 | 273610 | - | -76 | P | TON_0307 | TON_0307 | TON_0307 | 273600 | 273301 | 10 |
| TU0158 | 277948 | - | -6 | P | TON_0308 | TON_0308 | TON_0308 | 277922 | 273678 | 26 |
| TU0159 | 278589 | + | 21 | A | TON_nc011 | TON_nc011 | TON_nc011 | - | - | - |
| TU0160 | 279211 | - | -113 | P | TON_0309 | TON_0309 | TON_0309 | 279202 | 278165 | 9 |
| TU0161 | 279215 | + | 2 | P | TON_0310 | TON_0310 | TON_0310 | 279260 | 281725 | 45 |
| TU0162 | 281989 | + | 428 | P | TON_0311 | TON_0311 | TON_0311 | 282016 | 284364 | 27 |
| TU0163 | 284515 | + | 1 | P | TON_0312 | TON_0312 | TON_0312 | 284522 | 285907 | 7 |
| TU0164 | 285939 | + | 1 | P | TON_0313 | TON_0313 | TON_0313 | 285947 | 287371 | 8 |
| TU0165 | 287499 | + | 8 | S | TON_0314 | TON_0314 | TON_0314 | 287594 | 288262 | - |
| TU0166 | 287585 | + | 155 | P | TON_0314 | TON_0314 | TON_0314 | 287594 | 288262 | 9 |
| TU0167 | 288900 | + | 2 | P | TON_0315 | TON_0315 | TON_0315 | 288910 | 291063 | 10 |
| TU0168 | 293209 | + | 917 | P | TON_0319 | TON_0319 | TON_0319 | 293284 | 293964 | 75 |
| TU0169 | 293230 | - | -1192 | P | TON_0318; TON_0317; TON_0316 | TON_0318 | TON_0316 | 293148 | 291064 | 82 |
| TU0170 | 295789 | - | -83 | N | TON_nc012 | TON_nc012 | TON_nc012 | - | - | - |
| TU0171 | 298133 | - | -30 | P | TON_0322 | TON_0322 | TON_0322 | 297965 | 297243 | 168 |
| TU0172 | 302937 | - | -2734 | P | TON_0327 | TON_0327 | TON_0327 | 302914 | 302216 | 23 |
| TU0173 | 303051 | + | 13 | P | TON_0328 | TON_0328 | TON_0328 | 303061 | 304359 | 10 |
| TU0174 | 304599 | - | -266 | P | TON_0329 | TON_0329 | TON_0329 | 304589 | 304356 | 10 |
| TU0175 | 304674 | + | 3 | P | TON_0330 | TON_0330 | TON_0330 | 304686 | 305216 | 12 |
| TU0176 | 305255 | + | 148 | P | TON_0331 | TON_0331 | TON_0331 | 305268 | 308933 | 13 |
| TU0177 | 309192 | + | 125 | P | TON_0332 | TON_0332 | TON_0332 | 309336 | 310130 | 144 |
| TU0178 | 310451 | - | -4254 | P | TON_0333 | TON_0333 | TON_0333 | 310418 | 310206 | 33 |
| TU0179 | 311484 | - | -6 | P | TON_0335; TON_0334; TON_0333 | TON_0335 | TON_0333 | 311474 | 310206 | 10 |
| TU0180 | 311590 | + | 47 | P | TON_0336; TON_0337; TON_0338; TON_0339; TON_0340; TON_0341 | TON_0336 | TON_0341 | 311613 | 315859 | 23 |

| | | | | | | | | | | |
|---|---|---|---|---|---|---|---|---|---|---|
| TU0181 | 312832 | + | 87 | P | TON_0337; TON_0338; TON_0339; TON_0340; TON_0341 | TON_0337 | TON_0341 | 312843 | 315859 | 11 |
| TU0182 | 315921 | + | 5 | P | TON_0342; TON_0343 | TON_0342 | TON_0343 | 315962 | 317820 | 41 |
| TU0183 | 317853 | + | 24 | P | TON_0344 | TON_0344 | TON_0344 | 317881 | 319929 | 28 |
| TU0184 | 319979 | + | 1 | P | TON_0345; TON_0346 | TON_0345 | TON_0346 | 319989 | 321599 | 10 |
| TU0185 | 321653 | + | 22 | P | TON_0347 | TON_0347 | TON_0347 | 321664 | 322371 | 11 |
| TU0186 | 323711 | - | -97 | P | TON_0348 | TON_0348 | TON_0348 | 323702 | 322554 | 9 |
| TU0187 | 324665 | + | 99 | P | TON_0349 | TON_0349 | TON_0349 | 324677 | 324964 | 12 |
| TU0188 | 324895 | + | 8 | P | TON_0350 | TON_0350 | TON_0350 | 324977 | 325357 | 82 |
| TU0189 | 326273 | - | -2 | P | TON_0351 | TON_0351 | TON_0351 | 326263 | 325364 | 10 |
| TU0190 | 326370 | + | 5 | P | TON_0352 | TON_0352 | TON_0352 | 326389 | 327990 | 19 |
| TU0191 | 328887 | - | -9 | P | TON_0353 | TON_0353 | TON_0353 | 328877 | 327987 | 10 |
| TU0192 | 328963 | + | 215 | P | TON_0354 | TON_0354 | TON_0354 | 328995 | 329387 | 32 |
| TU0193 | 329509 | + | 42 | P | TON_0355 | TON_0355 | TON_0355 | 329520 | 330446 | 11 |
| TU0194 | 332399 | - | -54 | P | TON_0357 | TON_0357 | TON_0357 | 332388 | 331582 | 11 |
| TU0195 | 332436 | + | 3 | P | TON_0358; TON_0359; TON_0360 | TON_0358 | TON_0360 | 332446 | 335623 | 10 |
| TU0196 | 334794 | - | -18 | A | TON_nc013 | TON_nc013 | TON_nc013 | - | - | - |
| TU0197 | 334908 | - | -4 | A | TON_nc014 | TON_nc014 | TON_nc014 | - | - | - |
| TU0198 | 336823 | - | -256 | P | TON_0361 | TON_0361 | TON_0361 | 336807 | 335632 | 16 |
| TU0199 | 336946 | + | 22 | P | TON_1997 | TON_1997 | TON_1997 | 336950 | 337037 | - |
| TU0200 | 338027 | - | -61 | P | TON_0362 | TON_0362 | TON_0362 | 338016 | 337129 | 11 |
| TU0201 | 338106 | + | 7 | P | TON_0363; TON_0364 | TON_0363 | TON_0364 | 338115 | 340447 | 9 |
| TU0202 | 341825 | - | -4 | P | TON_0366; TON_0365 | TON_0366 | TON_0365 | 341775 | 340407 | 50 |
| TU0203 | 342723 | - | -589 | P | TON_0368; TON_0367 | TON_0368 | TON_0367 | 342712 | 341772 | 11 |
| TU0204 | 343914 | - | -77 | P | TON_0369 | TON_0369 | TON_0369 | 343889 | 342852 | 25 |
| TU0205 | 345893 | - | -43 | P | TON_0371; TON_0370 | TON_0371 | TON_0370 | 345882 | 343965 | 11 |
| TU0206 | 345944 | + | 1045 | P | TON_0372; TON_0373 | TON_0372 | TON_0373 | 345954 | 347279 | 10 |
| TU0207 | 347369 | + | 9945 | P | TON_0374 | TON_0374 | TON_0374 | 347380 | 348060 | 11 |
| TU0208 | 350753 | - | -14 | P | TON_0376 | TON_0376 | TON_0376 | 350742 | 348859 | 11 |
| TU0209 | 350852 | + | 207 | P | TON_0377; TON_0378; TON_0379 | TON_0377 | TON_0379 | 350863 | 352362 | 11 |
| TU0210 | 353771 | + | 44 | P | TON_0381 | TON_0381 | TON_0381 | 353781 | 354167 | 10 |
| TU0211 | 354861 | - | -4 | I | TON_0382 | TON_0382 | TON_0382 | 354968 | 354162 | - |

| | | | | | | | | | | |
|---|---|---|---|---|---|---|---|---|---|---|
| TU0212 | 354978 | - | -17 | P | TON_0382 | TON_0382 | TON_0382 | 354968 | 354162 | 10 |
| TU0213 | 355053 | + | 55 | P | TON_0383; TON_0384; TON_0385 | TON_0383 | TON_0385 | 355063 | 357556 | 10 |
| TU0214 | 358363 | - | -80 | P | TON_0386 | TON_0386 | TON_0386 | 358352 | 357534 | 11 |
| TU0215 | 360597 | + | 1 | P | TON_0390; TON_0391 | TON_0390 | TON_0391 | 360644 | 362433 | 47 |
| TU0216 | 363724 | - | -3 | S | TON_0392 | TON_0392 | TON_0392 | 363754 | 362489 | - |
| TU0217 | 363764 | - | -8 | P | TON_0392 | TON_0392 | TON_0392 | 363754 | 362489 | 10 |
| TU0218 | 363814 | + | 8 | P | TON_0393 | TON_0393 | TON_0393 | 363822 | 365198 | 8 |
| TU0219 | 365972 | - | -163 | P | TON_0394 | TON_0394 | TON_0394 | 365972 | 365169 | 0 |
| TU0220 | 366125 | + | 24 | N | TON_nc015 | TON_nc015 | TON_nc015 | - | - | - |
| TU0221 | 366207 | + | 4 | N | TON_nc016 | TON_nc016 | TON_nc016 | - | - | - |
| TU0222 | 369574 | - | -202 | P | TON_0396; TON_0395 | TON_0396 | TON_0395 | 369560 | 366326 | 14 |
| TU0223 | 370708 | - | -312 | P | TON_0397 | TON_0397 | TON_0397 | 370684 | 369632 | 24 |
| TU0224 | 370826 | + | 3 | P | TON_0398; TON_0399; TON_0400 | TON_0398 | TON_0400 | 370845 | 372938 | 19 |
| TU0225 | 373067 | + | 9 | P | TON_0401 | TON_0401 | TON_0401 | 373079 | 373765 | 12 |
| TU0226 | 377032 | - | -33 | P | TON_0403; TON_0402 | TON_0403 | TON_0402 | 377020 | 373803 | 12 |
| TU0227 | 377421 | - | -103 | P | TON_0404 | TON_0404 | TON_0404 | 377504 | 377127 | -83 |
| TU0228 | 377422 | + | 831 | P | TON_0405 | TON_0405 | TON_0405 | 377466 | 378626 | 44 |
| TU0229 | 378661 | + | 68 | P | TON_0406; TON_0407 | TON_0406 | TON_0407 | 378720 | 379435 | 59 |
| TU0230 | 379493 | + | 83 | P | TON_0408; TON_0409 | TON_0408 | TON_0409 | 379505 | 380940 | 12 |
| TU0231 | 381538 | - | -38 | P | TON_0410 | TON_0410 | TON_0410 | 381528 | 380974 | 10 |
| TU0232 | 381622 | + | 2 | P | TON_0411 | TON_0411 | TON_0411 | 381622 | 383367 | 0 |
| TU0233 | 384143 | - | -21 | P | TON_0412 | TON_0412 | TON_0412 | 384136 | 383357 | 7 |
| TU0234 | 386096 | - | -26 | P | TON_0413 | TON_0413 | TON_0413 | 386038 | 384182 | 58 |
| TU0235 | 387090 | - | -9 | P | TON_0414 | TON_0414 | TON_0414 | 387062 | 386238 | 28 |
| TU0236 | 388122 | - | -78 | P | TON_0415; TON_0414 | TON_0415 | TON_0414 | 388111 | 386238 | 11 |
| TU0237 | 390945 | - | -41 | P | TON_0418; TON_0417; TON_0416 | TON_0418 | TON_0416 | 390936 | 388137 | 9 |
| TU0238 | 391969 | - | -1 | P | TON_0419 | TON_0419 | TON_0419 | 391962 | 391027 | 7 |
| TU0239 | 394048 | - | -1 | P | TON_0421 | TON_0421 | TON_0421 | 394030 | 392765 | 18 |
| TU0240 | 394086 | + | 873 | P | TON_0422 | TON_0422 | TON_0422 | 394096 | 394566 | 10 |
| TU0241 | 394587 | + | 16 | P | TON_0423 | TON_0423 | TON_0423 | 394587 | 396308 | 0 |
| TU0242 | 396068 | - | -5 | A | TON_nc017 | TON_nc017 | TON_nc017 | - | - | - |
| TU0243 | 396263 | + | 45 | P | TON_0424 | TON_0424 | TON_0424 | 396395 | 397735 | 132 |

| | | | | | | | | | | |
|---|---|---|---|---|---|---|---|---|---|---|
| TU0244 | 397415 | - | -15 | A | TON_nc018 | TON_nc018 | TON_nc018 | - | - | - |
| TU0245 | 398613 | - | -1 | P | TON_0425 | TON_0425 | TON_0425 | 398601 | 397738 | 12 |
| TU0246 | 399116 | - | -9 | P | TON_0426 | TON_0426 | TON_0426 | 399087 | 398677 | 29 |
| TU0247 | 400588 | - | -2 | P | TON_0427 | TON_0427 | TON_0427 | 400542 | 399163 | 46 |
| TU0248 | 402378 | - | -57 | P | TON_0429; TON_0428 | TON_0429 | TON_0428 | 402369 | 400644 | 9 |
| TU0249 | 402428 | + | 1 | P | TON_0430; TON_0431 | TON_0430 | TON_0431 | 402429 | 403969 | 1 |
| TU0250 | 402973 | + | 11 | P | TON_0431 | TON_0431 | TON_0431 | 403007 | 403969 | 34 |
| TU0251 | 405258 | - | -270 | P | TON_0433; TON_0432 | TON_0433 | TON_0432 | 405235 | 403938 | 23 |
| TU0252 | 407250 | - | -4 | P | TON_0435; TON_0434 | TON_0435 | TON_0434 | 407240 | 405296 | 10 |
| TU0253 | 407463 | + | 31 | P | TON_0436 | TON_0436 | TON_0436 | 407475 | 407981 | 12 |
| TU0254 | 408057 | + | 21 | P | TON_0437 | TON_0437 | TON_0437 | 408067 | 408804 | 10 |
| TU0255 | 411394 | + | 148 | P | TON_0440 | TON_0440 | TON_0440 | 411370 | 412101 | -24 |
| TU0256 | 412613 | - | -334 | P | TON_0441 | TON_0441 | TON_0441 | 412601 | 412098 | 12 |
| TU0257 | 412723 | + | 327 | P | TON_0442 | TON_0442 | TON_0442 | 412733 | 413965 | 10 |
| TU0258 | 420659 | - | -5 | P | TON_0447; TON_0446 | TON_0447 | TON_0446 | 420645 | 418875 | 14 |
| TU0259 | 421043 | - | -23 | P | TON_0448 | TON_0448 | TON_0448 | 421022 | 420696 | 21 |
| TU0260 | 422728 | - | -17 | P | TON_0451 | TON_0451 | TON_0451 | 422701 | 422351 | 27 |
| TU0261 | 423634 | - | -100 | P | TON_0453; TON_0452 | TON_0453 | TON_0452 | 423627 | 422765 | 7 |
| TU0262 | 424162 | - | -198 | P | TON_0455 | TON_0455 | TON_0455 | 424126 | 423866 | 36 |
| TU0263 | 424692 | - | -25 | N | TON_nc019 | TON_nc019 | TON_nc019 | - | - | - |
| TU0264 | 424741 | + | 122 | P | TON_0456 | TON_0456 | TON_0456 | 424751 | 425251 | 10 |
| TU0265 | 426800 | + | 912 | P | TON_0460; TON_0461 | TON_0460 | TON_0461 | 426893 | 428868 | 93 |
| TU0266 | 432674 | - | -57 | P | TON_0466 | TON_0466 | TON_0466 | 432666 | 432205 | 8 |
| TU0267 | 432789 | + | 146 | P | TON_1970 | TON_1970 | TON_1970 | 432720 | 433067 | -69 |
| TU0268 | 433524 | - | -5 | P | TON_0468 | TON_0468 | TON_0468 | 433513 | 433262 | 11 |
| TU0269 | 434088 | - | -2 | P | TON_0469 | TON_0469 | TON_0469 | 434078 | 433614 | 10 |
| TU0270 | 434162 | + | 123 | P | TON_0470 | TON_0470 | TON_0470 | 434172 | 434789 | 10 |
| TU0271 | 434852 | + | 450 | P | TON_0471 | TON_0471 | TON_0471 | 434852 | 435580 | 0 |
| TU0272 | 435934 | - | -16 | P | TON_0472 | TON_0472 | TON_0472 | 435922 | 435572 | 12 |
| TU0273 | 437394 | - | -96 | P | TON_0474 | TON_0474 | TON_0474 | 437383 | 436568 | 11 |
| TU0274 | 437840 | - | -183 | P | TON_0475 | TON_0475 | TON_0475 | 437840 | 437454 | 0 |
| TU0275 | 438147 | + | 20 | P | TON_0476 | TON_0476 | TON_0476 | 438181 | 439545 | 34 |

| | | | | | | | | | | |
|---|---|---|---|---|---|---|---|---|---|---|
| TU0276 | 440143 | + | 894 | P | TON_0479; TON_0480; TON_0481 | TON_0479 | TON_0481 | 440156 | 442889 | 13 |
| TU0277 | 441478 | + | 506 | P | TON_0480; TON_0481 | TON_0480 | TON_0481 | 441498 | 442889 | 20 |
| TU0278 | 444869 | + | 24 | P | TON_0484 | TON_0484 | TON_0484 | 444877 | 446178 | 8 |
| TU0279 | 446804 | - | -16 | P | TON_0485 | TON_0485 | TON_0485 | 446793 | 446209 | 11 |
| TU0280 | 456707 | - | -97 | P | TON_0498; TON_0497; TON_0496; TON_0495; TON_0494; TON_0493; TON_0492; TON_0491; TON_0490; TON_0489; TON_0488; TON_0487; | TON_0498 | TON_0486 | 456653 | 446875 | 54 |
| TU0281 | 457453 | - | -841 | P | TON_0499 | TON_0499 | TON_0499 | 457428 | 456928 | 25 |
| TU0282 | 457498 | - | -12 | S | TON_0499 | TON_0499 | TON_0499 | 457428 | 456928 | - |
| TU0283 | 459096 | - | -21 | N | TON_nc020 | TON_nc020 | TON_nc020 | - | - | - |
| TU0284 | 461617 | - | -169 | P | TON_0502; TON_0501 | TON_0502 | TON_0501 | 461607 | 459188 | 10 |
| TU0285 | 461882 | + | 95 | P | TON_0503 | TON_0503 | TON_0503 | 461893 | 463020 | 11 |
| TU0286 | 463827 | - | -444 | P | TON_0504; TON_1971 | TON_0504 | TON_1971 | 463817 | 462955 | 10 |
| TU0287 | 463877 | + | 128 | P | TON_0505 | TON_0505 | TON_0505 | 463887 | 465815 | 10 |
| TU0288 | 468013 | - | -97 | P | TON_0507 | TON_0507 | TON_0507 | 467982 | 466735 | 31 |
| TU0289 | 468809 | - | -4 | P | TON_0509 | TON_0509 | TON_0509 | 468805 | 468326 | 4 |
| TU0290 | 469464 | - | -220 | P | TON_0510 | TON_0510 | TON_0510 | 469438 | 469262 | 26 |
| TU0291 | 469516 | + | 7 | P | TON_0511 | TON_0511 | TON_0511 | 469556 | 470119 | 40 |
| TU0292 | 471373 | - | -21 | P | TON_0512 | TON_0512 | TON_0512 | 471359 | 470109 | 14 |
| TU0293 | 472974 | - | -7 | P | TON_0514; TON_0513 | TON_0514 | TON_0513 | 472965 | 471403 | 9 |
| TU0294 | 473065 | + | 1 | P | TON_0515 | TON_0515 | TON_0515 | 473544 | 474248 | 479 |
| TU0295 | 474326 | + | 5 | P | TON_1998 | TON_1998 | TON_1998 | 474335 | 474412 | - |
| TU0296 | 474682 | + | 4 | P | TON_0516 | TON_0516 | TON_0516 | 474692 | 475237 | 10 |
| TU0297 | 475275 | + | 474 | P | TON_0517 | TON_0517 | TON_0517 | 475321 | 475776 | 46 |
| TU0298 | 478178 | + | 12 | P | TON_0521 | TON_0521 | TON_0521 | 478189 | 479244 | 11 |
| TU0299 | 480092 | - | -14 | P | TON_0522 | TON_0522 | TON_0522 | 480085 | 479372 | 7 |
| TU0300 | 480634 | - | -1061 | P | TON_0523 | TON_0523 | TON_0523 | 480612 | 480163 | 22 |
| TU0301 | 481130 | - | -296 | P | TON_0524 | TON_0524 | TON_0524 | 481086 | 480784 | 44 |
| TU0302 | 481480 | - | -156 | P | TON_0525; TON_0524 | TON_0525 | TON_0524 | 481469 | 480784 | 11 |
| TU0303 | 483202 | - | -80 | P | TON_0528; TON_0527 | TON_0528 | TON_0527 | 483192 | 481929 | 10 |
| TU0304 | 483287 | + | 64 | P | TON_0529 | TON_0529 | TON_0529 | 483311 | 485218 | 24 |
| TU0305 | 485322 | + | 26 | P | TON_0530; TON_0531 | TON_0530 | TON_0531 | 485334 | 487410 | 12 |

| | | | | | | | | | | |
|---|---|---|---|---|---|---|---|---|---|---|
| TU0306 | 487505 | + | 15 | P | TON_0532 | TON_0532 | TON_0532 | 487523 | 487816 | 18 |
| TU0307 | 488292 | - | -36 | P | TON_0533 | TON_0533 | TON_0533 | 488280 | 487831 | 12 |
| TU0308 | 492450 | - | -62 | P | TON_0537; TON_0536; TON_0535; TON_0534 | TON_0537 | TON_0534 | 492397 | 488328 | 53 |
| TU0309 | 492734 | + | 62 | P | TON_0538; TON_0539; TON_0540; TON_0541; TON_0542; TON_0543 | TON_0538 | TON_0543 | 492769 | 498286 | 35 |
| TU0310 | 498551 | + | 203 | P | TON_0544 | TON_0544 | TON_0544 | 498574 | 499794 | 23 |
| TU0311 | 502213 | - | -9 | P | TON_0546 | TON_0546 | TON_0546 | 502189 | 500315 | 24 |
| TU0312 | 503530 | - | -40 | P | TON_0547 | TON_0547 | TON_0547 | 503480 | 502293 | 50 |
| TU0313 | 504419 | - | -1555 | N | TON_nc021 | TON_nc021 | TON_nc021 | 504170 | 503598 | - |
| TU0314 | 505327 | - | -24 | P | TON_0549 | TON_0549 | TON_0549 | 505327 | 504458 | 0 |
| TU0315 | 507140 | - | -19 | P | TON_0551 | TON_0551 | TON_0551 | 507127 | 506333 | 13 |
| TU0316 | 507260 | + | 15 | P | TON_0552 | TON_0552 | TON_0552 | 507269 | 508009 | 9 |
| TU0317 | 508051 | + | 402 | N | TON_nc022 | TON_nc022 | TON_nc022 | - | - | - |
| TU0318 | 509870 | + | 3 | P | TON_0554 | TON_0554 | TON_0554 | 509938 | 511401 | 68 |
| TU0319 | 509881 | - | -23 | P | TON_0553 | TON_0553 | TON_0553 | 509824 | 508118 | 57 |
| TU0320 | 511623 | - | -622 | P | TON_0555 | TON_0555 | TON_0555 | 511613 | 511404 | 10 |
| TU0321 | 514034 | - | -379 | P | TON_0559 | TON_0559 | TON_0559 | 514025 | 513408 | 9 |
| TU0322 | 514335 | + | 881 | P | TON_0560 | TON_0560 | TON_0560 | 514360 | 515577 | 25 |
| TU0323 | 515633 | + | 15 | A | TON_nc023 | TON_nc023 | TON_nc023 | - | - | - |
| TU0324 | 515850 | - | -899 | P | TON_0561 | TON_0561 | TON_0561 | 515898 | 515623 | -48 |
| TU0325 | 515958 | + | 4 | P | TON_0562; TON_0563; TON_0564 | TON_0562 | TON_0564 | 515978 | 518045 | 20 |
| TU0326 | 519221 | - | -29 | P | TON_0565 | TON_0565 | TON_0565 | 519211 | 518042 | 10 |
| TU0327 | 520972 | + | 2091 | P | TON_0567 | TON_0567 | TON_0567 | 520987 | 521373 | 15 |
| TU0328 | 522910 | - | -18 | P | TON_0568 | TON_0568 | TON_0568 | 522899 | 521451 | 11 |
| TU0329 | 523014 | + | 13 | P | TON_0569; TON_0570 | TON_0569 | TON_0570 | 523025 | 525032 | 11 |
| TU0330 | 526374 | + | 5 | P | TON_0573 | TON_0573 | TON_0573 | 526374 | 527105 | 0 |
| TU0331 | 527142 | + | 19 | P | TON_0574 | TON_0574 | TON_0574 | 527152 | 527373 | 10 |
| TU0332 | 529728 | + | 24218 | P | TON_0577 | TON_0577 | TON_0577 | 529740 | 530981 | 12 |
| TU0333 | 534214 | - | -165 | P | TON_0579 | TON_0579 | TON_0579 | 534204 | 532003 | 10 |
| TU0334 | 534355 | + | 4 | P | TON_0580 | TON_0580 | TON_0580 | 534384 | 534731 | 29 |
| TU0335 | 535198 | - | -17 | P | TON_0581 | TON_0581 | TON_0581 | 535198 | 534728 | 0 |
| TU0336 | 540666 | - | -556 | P | TON_0588; TON_0587; TON_0586; TON_0585; TON_0584; TON_0583; | TON_0588 | TON_0582 | 540645 | 535259 | 21 |

| | | | | | | | | | | |
|---|---|---|---|---|---|---|---|---|---|---|
| TU0337 | 541778 | - | -10 | P | TON_0589 | TON_0589 | TON_0589 | 541767 | 540796 | 11 |
| TU0338 | 541829 | + | 496 | P | TON_0590 | TON_0590 | TON_0590 | 541829 | 542611 | 0 |
| TU0339 | 544115 | - | -36 | P | TON_0592 | TON_0592 | TON_0592 | 544104 | 543199 | 11 |
| TU0340 | 544214 | - | -309 | N | TON_nc024 | TON_nc024 | TON_nc024 | - | - | - |
| TU0341 | 544701 | - | -4 | P | TON_0593 | TON_0593 | TON_0593 | 544694 | 544245 | 7 |
| TU0342 | 544797 | + | 28 | P | TON_0594; TON_0595; TON_0596; TON_0597 | TON_0594 | TON_0597 | 544818 | 549534 | 21 |
| TU0343 | 550580 | - | -89 | P | TON_0598 | TON_0598 | TON_0598 | 550567 | 549584 | 13 |
| TU0344 | 552785 | - | -142 | P | TON_0600 | TON_0600 | TON_0600 | 552763 | 552428 | 22 |
| TU0345 | 553435 | - | -435 | P | TON_0601 | TON_0601 | TON_0601 | 553423 | 552776 | 12 |
| TU0346 | 554961 | - | -4 | P | TON_0602 | TON_0602 | TON_0602 | 554945 | 553515 | 16 |
| TU0347 | 555041 | + | 10 | P | TON_0603 | TON_0603 | TON_0603 | 555062 | 556111 | 21 |
| TU0348 | 556149 | + | 11 | P | TON_0604 | TON_0604 | TON_0604 | 556149 | 556751 | 0 |
| TU0349 | 556787 | + | 19 | P | TON_0605 | TON_0605 | TON_0605 | 556787 | 557539 | 0 |
| TU0350 | 559435 | - | -49 | P | TON_0606 | TON_0606 | TON_0606 | 559424 | 557499 | 11 |
| TU0351 | 560425 | - | -11 | P | TON_0607 | TON_0607 | TON_0607 | 560386 | 559928 | 39 |
| TU0352 | 563238 | - | -14 | P | TON_0610; TON_0609; TON_0608 | TON_0610 | TON_0608 | 563238 | 560452 | 0 |
| TU0353 | 563328 | + | 2 | P | TON_0611 | TON_0611 | TON_0611 | 563339 | 565189 | 11 |
| TU0354 | 565299 | + | 29 | P | TON_0612 | TON_0612 | TON_0612 | 565310 | 566014 | 11 |
| TU0355 | 570176 | - | -124 | P | TON_0613 | TON_0613 | TON_0613 | 570175 | 566006 | 1 |
| TU0356 | 570257 | + | 19 | P | TON_0614 | TON_0614 | TON_0614 | 570267 | 570542 | 10 |
| TU0357 | 570567 | + | 7 | P | TON_0615 | TON_0615 | TON_0615 | 570567 | 571616 | 0 |
| TU0358 | 572673 | + | 44 | P | TON_0617 | TON_0617 | TON_0617 | 572684 | 574426 | 11 |
| TU0359 | 574466 | + | 108 | P | TON_1999 | TON_1999 | TON_1999 | 574470 | 574546 | - |
| TU0360 | 578314 | - | -109 | P | TON_0619 | TON_0619 | TON_0619 | 578313 | 577168 | 1 |
| TU0361 | 578454 | + | 50 | P | TON_0620 | TON_0620 | TON_0620 | 578484 | 579212 | 30 |
| TU0362 | 579244 | + | 3224 | P | TON_0621 | TON_0621 | TON_0621 | 579317 | 579970 | 73 |
| TU0363 | 586728 | - | -196 | P | TON_0628 | TON_0628 | TON_0628 | 586728 | 585844 | 0 |
| TU0364 | 586814 | + | 2 | P | TON_0629; TON_0630 | TON_0629 | TON_0630 | 586852 | 588584 | 38 |
| TU0365 | 591969 | + | 136 | P | TON_0636 | TON_0636 | TON_0636 | 591980 | 594619 | 11 |
| TU0366 | 596355 | - | -23 | P | TON_0638; TON_0637 | TON_0638 | TON_0637 | 596346 | 594677 | 9 |
| TU0367 | 596492 | + | 5 | P | TON_0639 | TON_0639 | TON_0639 | 596501 | 597505 | 9 |
| TU0368 | 601325 | - | -68 | P | TON_0643 | TON_0643 | TON_0643 | 601314 | 599611 | 11 |

| | | | | | | | | | | |
|---|---|---|---|---|---|---|---|---|---|---|
| TU0369 | 601434 | + | 1675 | P | TON_0644 | TON_0644 | TON_0644 | 601464 | 601700 | 30 |
| TU0370 | 604533 | - | -351 | P | TON_0647 | TON_0647 | TON_0647 | 604521 | 603427 | 12 |
| TU0371 | 606054 | - | -32 | P | TON_0649 | TON_0649 | TON_0649 | 606038 | 605772 | 16 |
| TU0372 | 606743 | - | -24 | P | TON_0650 | TON_0650 | TON_0650 | 606743 | 606087 | 0 |
| TU0373 | 606821 | + | 8 | P | TON_0651 | TON_0651 | TON_0651 | 606831 | 607877 | 10 |
| TU0374 | 607002 | + | 12 | I | TON_0651 | TON_0651 | TON_0651 | 606831 | 607877 | - |
| TU0375 | 610808 | - | -48 | P | TON_0654; TON_0653 | TON_0654 | TON_0653 | 610808 | 609019 | 0 |
| TU0376 | 611270 | - | -37 | P | TON_0655 | TON_0655 | TON_0655 | 611244 | 610846 | 26 |
| TU0377 | 614026 | + | 4 | P | TON_0659 | TON_0659 | TON_0659 | 614035 | 615276 | 9 |
| TU0378 | 616242 | - | -128 | P | TON_0660 | TON_0660 | TON_0660 | 616231 | 615428 | 11 |
| TU0379 | 616322 | + | 50 | P | TON_0661 | TON_0661 | TON_0661 | 616332 | 616817 | 10 |
| TU0380 | 616826 | + | 150 | P | TON_0662; TON_0663 | TON_0662 | TON_0663 | 616866 | 617870 | 40 |
| TU0381 | 617898 | + | 9 | P | TON_0664; TON_0665 | TON_0664 | TON_0665 | 617950 | 619295 | 52 |
| TU0382 | 620485 | - | -27 | P | TON_0666 | TON_0666 | TON_0666 | 620475 | 619282 | 10 |
| TU0383 | 620572 | + | 18 | S | TON_0667 | TON_0667 | TON_0667 | 620716 | 620910 | - |
| TU0384 | 620595 | + | 62 | P | TON_0667 | TON_0667 | TON_0667 | 620716 | 620910 | 121 |
| TU0385 | 622344 | - | -14 | P | TON_0670 | TON_0670 | TON_0670 | 622114 | 621770 | 230 |
| TU0386 | 622438 | + | 11 | P | TON_0671; TON_0672 | TON_0671 | TON_0672 | 622462 | 625165 | 24 |
| TU0387 | 626254 | - | -20 | P | TON_0673 | TON_0673 | TON_0673 | 626244 | 625249 | 10 |
| TU0388 | 626359 | + | 4 | P | TON_2000 | TON_2000 | TON_2000 | 626359 | 626436 | - |
| TU0389 | 626597 | + | 2294 | P | TON_0674 | TON_0674 | TON_0674 | 626783 | 627535 | 186 |
| TU0390 | 626775 | + | 12 | S | TON_0674 | TON_0674 | TON_0674 | 626783 | 627535 | - |
| TU0391 | 627638 | + | 5 | P | TON_0675 | TON_0675 | TON_0675 | 627600 | 628757 | -38 |
| TU0392 | 629366 | - | -63 | P | TON_0676 | TON_0676 | TON_0676 | 629355 | 628774 | 11 |
| TU0393 | 632197 | - | -245 | P | TON_0677 | TON_0677 | TON_0677 | 632166 | 629512 | 31 |
| TU0394 | 635715 | - | -68 | P | TON_0680; TON_0679; TON_0678 | TON_0680 | TON_0678 | 635715 | 633150 | 0 |
| TU0395 | 635749 | + | 96 | P | TON_0681; TON_0682; TON_0683; TON_0684 | TON_0681 | TON_0684 | 635758 | 637756 | 9 |
| TU0396 | 638885 | - | -1 | P | TON_0685 | TON_0685 | TON_0685 | 638885 | 637770 | 0 |
| TU0397 | 640935 | - | -1 | P | TON_0687 | TON_0687 | TON_0687 | 640919 | 639465 | 16 |
| TU0398 | 641032 | + | 19 | P | TON_0688 | TON_0688 | TON_0688 | 640988 | 641995 | -44 |
| TU0399 | 642579 | - | -4 | P | TON_0689 | TON_0689 | TON_0689 | 642579 | 641992 | 0 |
| TU0400 | 642682 | + | 42 | P | TON_0690; TON_0691 | TON_0690 | TON_0691 | 642692 | 644400 | 10 |

| | | | | | | | | | | |
|---|---|---|---|---|---|---|---|---|---|---|
| TU0401 | 644706 | - | -5940 | P | TON_0692 | TON_0692 | TON_0692 | 644697 | 644476 | 9 |
| TU0402 | 645021 | - | -8 | N | TON_nc025 | TON_nc025 | TON_nc025 | - | - | - |
| TU0403 | 647218 | - | -189 | P | TON_0694; TON_0693 | TON_0694 | TON_0693 | 647218 | 645060 | 0 |
| TU0404 | 647340 | + | 45 | P | TON_0695 | TON_0695 | TON_0695 | 647359 | 648642 | 19 |
| TU0405 | 648307 | + | 29 | I | TON_0695 | TON_0695 | TON_0695 | 647359 | 648642 | - |
| TU0406 | 650928 | - | -18 | P | TON_0697 | TON_0697 | TON_0697 | 650927 | 649968 | 1 |
| TU0407 | 650959 | + | 621 | P | TON_0698 | TON_0698 | TON_0698 | 650970 | 651503 | 11 |
| TU0408 | 653988 | - | -248 | P | TON_0700 | TON_0700 | TON_0700 | 653988 | 652816 | 0 |
| TU0409 | 655083 | - | -116 | P | TON_0701 | TON_0701 | TON_0701 | 655083 | 654076 | 0 |
| TU0410 | 656336 | + | 701 | P | TON_0703 | TON_0703 | TON_0703 | 656336 | 657142 | 0 |
| TU0411 | 659693 | - | -16 | P | TON_0705 | TON_0705 | TON_0705 | 659664 | 658792 | 29 |
| TU0412 | 662018 | - | -77 | P | TON_0707 | TON_0707 | TON_0707 | 661988 | 660336 | 30 |
| TU0413 | 662154 | + | 67 | P | TON_0708 | TON_0708 | TON_0708 | 662165 | 662476 | 11 |
| TU0414 | 668921 | - | -5 | P | TON_0714 | TON_0714 | TON_0714 | 668910 | 667471 | 11 |
| TU0415 | 670836 | + | 4 | P | TON_0718; TON_0719 | TON_0718 | TON_0719 | 670847 | 672370 | 11 |
| TU0416 | 673202 | - | -13 | P | TON_0720 | TON_0720 | TON_0720 | 673202 | 672360 | 0 |
| TU0417 | 673423 | + | 10 | P | TON_0721 | TON_0721 | TON_0721 | 673454 | 673879 | 31 |
| TU0418 | 675156 | + | 71 | P | TON_0723; TON_0724 | TON_0723 | TON_0724 | 675166 | 676233 | 10 |
| TU0419 | 679055 | + | 54 | P | TON_0726 | TON_0726 | TON_0726 | 679111 | 679326 | 56 |
| TU0420 | 679411 | + | 50 | P | TON_0727 | TON_0727 | TON_0727 | 679421 | 681196 | 10 |
| TU0421 | 681314 | + | 4 | P | TON_0728; TON_0729 | TON_0728 | TON_0729 | 681403 | 682334 | 89 |
| TU0422 | 682547 | + | 23 | P | TON_0730 | TON_0730 | TON_0730 | 682560 | 683744 | 13 |
| TU0423 | 683946 | + | 26 | P | TON_0731 | TON_0731 | TON_0731 | 683974 | 684804 | 28 |
| TU0424 | 685389 | + | 28 | P | TON_0734; TON_0735; TON_0736 | TON_0734 | TON_0736 | 685424 | 686914 | 35 |
| TU0425 | 688531 | - | -1510 | P | TON_0738; TON_0737 | TON_0738 | TON_0737 | 688531 | 686911 | 0 |
| TU0426 | 688589 | + | 21 | P | TON_0739 | TON_0739 | TON_0739 | 688599 | 689390 | 10 |
| TU0427 | 689425 | + | 16 | P | TON_0740; TON_0741 | TON_0740 | TON_0741 | 689434 | 691358 | 9 |
| TU0428 | 693542 | - | -714 | P | TON_0743 | TON_0743 | TON_0743 | 693533 | 692235 | 9 |
| TU0429 | 693642 | + | 19 | P | TON_0744 | TON_0744 | TON_0744 | 693681 | 693860 | 39 |
| TU0430 | 693923 | + | 4 | P | TON_0745 | TON_0745 | TON_0745 | 693931 | 694809 | 8 |
| TU0431 | 695487 | - | -1 | P | TON_0746 | TON_0746 | TON_0746 | 695470 | 694826 | 17 |
| TU0432 | 695581 | - | -628 | N | TON_nc026 | TON_nc026 | TON_nc026 | - | - | - |

| | | | | | | | | | | |
|---|---|---|---|---|---|---|---|---|---|---|
| TU0433 | 696018 | - | -6768 | P | TON_0747 | TON_0747 | TON_0747 | 695989 | 695654 | 29 |
| TU0434 | 696116 | + | 20 | P | TON_0748 | TON_0748 | TON_0748 | 696117 | 696956 | 1 |
| TU0435 | 697194 | - | -2 | N | TON_nc027 | TON_nc027 | TON_nc027 | - | - | - |
| TU0436 | 701463 | - | -220 | P | TON_0752 | TON_0752 | TON_0752 | 701437 | 700151 | 26 |
| TU0437 | 701558 | - | -58 | N | TON_nc028 | TON_nc028 | TON_nc028 | 701437 | 700151 | - |
| TU0438 | 705305 | - | -75 | P | TON_0755 | TON_0755 | TON_0755 | 705273 | 703075 | 32 |
| TU0439 | 706499 | - | -3 | P | TON_0756 | TON_0756 | TON_0756 | 706482 | 705418 | 17 |
| TU0440 | 707336 | - | -63 | P | TON_0757 | TON_0757 | TON_0757 | 707315 | 706509 | 21 |
| TU0441 | 714521 | - | -10 | P | TON_0767; TON_0766; TON_0765; TON_0764; TON_0763; TON_0762 | TON_0767 | TON_0762 | 714500 | 708536 | 21 |
| TU0442 | 714671 | + | 24 | P | TON_0768; TON_0769; TON_0770 | TON_0768 | TON_0770 | 714681 | 716981 | 10 |
| TU0443 | 718404 | - | -663 | P | TON_0771 | TON_0771 | TON_0771 | 718395 | 717010 | 9 |
| TU0444 | 718455 | + | 3398 | P | TON_0772 | TON_0772 | TON_0772 | 718455 | 719603 | 0 |
| TU0445 | 719802 | - | -17 | P | TON_0773 | TON_0773 | TON_0773 | 719792 | 719598 | 10 |
| TU0446 | 726288 | - | -19 | P | TON_0779; TON_0778; TON_0777 | TON_0779 | TON_0777 | 726278 | 725041 | 10 |
| TU0447 | 727003 | - | -868 | P | TON_0780 | TON_0780 | TON_0780 | 726994 | 726320 | 9 |
| TU0448 | 728091 | - | -19 | P | TON_0782 | TON_0782 | TON_0782 | 728091 | 727480 | 0 |
| TU0449 | 728579 | + | 717 | N | TON_nc029 | TON_nc029 | TON_nc029 | - | - | - |
| TU0450 | 732734 | - | -17 | P | TON_0784; TON_0783 | TON_0784 | TON_0783 | 732713 | 730258 | 21 |
| TU0451 | 733558 | - | -8 | I | TON_0785 | TON_0785 | TON_0785 | 734209 | 732788 | - |
| TU0452 | 734220 | - | -10 | P | TON_0785 | TON_0785 | TON_0785 | 734209 | 732788 | 11 |
| TU0453 | 734297 | + | 87 | P | TON_0786 | TON_0786 | TON_0786 | 734306 | 734767 | 9 |
| TU0454 | 735503 | + | 32 | P | TON_0788 | TON_0788 | TON_0788 | 735506 | 735706 | 3 |
| TU0455 | 737444 | - | -1487 | P | TON_0790 | TON_0790 | TON_0790 | 737433 | 736795 | 11 |
| TU0456 | 740743 | + | 330 | P | TON_0797 | TON_0797 | TON_0797 | 740787 | 741296 | 44 |
| TU0457 | 741359 | + | 3 | P | TON_0798 | TON_0798 | TON_0798 | 741380 | 742594 | 21 |
| TU0458 | 742636 | + | 179 | P | TON_0799 | TON_0799 | TON_0799 | 742731 | 743468 | 95 |
| TU0459 | 746597 | + | 6 | P | TON_0804; TON_0805 | TON_0804 | TON_0805 | 746619 | 749373 | 22 |
| TU0460 | 753672 | - | -362 | P | TON_0811; TON_0810; TON_0809; TON_0808 | TON_0811 | TON_0808 | 753662 | 751740 | 10 |
| TU0461 | 753846 | + | 3 | P | TON_0812 | TON_0812 | TON_0812 | 753871 | 754386 | 25 |
| TU0462 | 756952 | + | 11 | N | TON_nc030 | TON_nc030 | TON_nc030 | - | - | - |
| TU0463 | 759676 | - | -25 | P | TON_0818 | TON_0818 | TON_0818 | 759544 | 759128 | 132 |

| | | | | | | | | | | |
|---|---|---|---|---|---|---|---|---|---|---|
| TU0464 | 760293 | - | -42 | P | TON_0819 | TON_0819 | TON_0819 | 760293 | 759541 | 0 |
| TU0465 | 760428 | + | 129 | P | TON_0820 | TON_0820 | TON_0820 | 760441 | 761625 | 13 |
| TU0466 | 761752 | + | 161 | P | TON_0821 | TON_0821 | TON_0821 | 761764 | 763047 | 12 |
| TU0467 | 763216 | + | 2769 | P | TON_0822; TON_0823 | TON_0822 | TON_0823 | 763497 | 764368 | 281 |
| TU0468 | 763651 | - | -9 | A | TON_nc031 | TON_nc031 | TON_nc031 | - | - | - |
| TU0469 | 764411 | + | 363 | P | TON_0824; TON_0825; TON_0826 | TON_0824 | TON_0826 | 764434 | 765682 | 23 |
| TU0470 | 768394 | + | 723 | P | TON_0829 | TON_0829 | TON_0829 | 768428 | 769078 | 34 |
| TU0471 | 771420 | - | -19 | P | TON_0832; TON_0831; TON_0830 | TON_0832 | TON_0830 | 771411 | 769158 | 9 |
| TU0472 | 775236 | + | 3 | P | TON_0836 | TON_0836 | TON_0836 | 775247 | 775855 | 11 |
| TU0473 | 776248 | - | -3 | P | TON_0837 | TON_0837 | TON_0837 | 776223 | 775888 | 25 |
| TU0474 | 777311 | - | -12 | P | TON_0839 | TON_0839 | TON_0839 | 777229 | 776996 | 82 |
| TU0475 | 779560 | - | -56 | P | TON_0840 | TON_0840 | TON_0840 | 779334 | 777406 | 226 |
| TU0476 | 780664 | + | 14 | N | TON_nc032 | TON_nc032 | TON_nc032 | - | - | - |
| TU0477 | 781188 | - | -10 | I | TON_0842 | TON_0842 | TON_0842 | 781514 | 780711 | - |
| TU0478 | 781618 | - | -6 | P | TON_0842 | TON_0842 | TON_0842 | 781514 | 780711 | 104 |
| TU0479 | 782274 | - | -1 | P | TON_0843 | TON_0843 | TON_0843 | 782263 | 782057 | 11 |
| TU0480 | 783120 | - | -36 | P | TON_0845; TON_0844 | TON_0845 | TON_0844 | 783109 | 782358 | 11 |
| TU0481 | 784061 | - | -4 | P | TON_0846 | TON_0846 | TON_0846 | 784035 | 783178 | 26 |
| TU0482 | 784888 | - | -4344 | P | TON_0847 | TON_0847 | TON_0847 | 784874 | 784305 | 14 |
| TU0483 | 785007 | + | 841 | P | TON_0848 | TON_0848 | TON_0848 | 785016 | 785591 | 9 |
| TU0484 | 789111 | - | -112 | P | TON_0851; TON_0850; TON_0849 | TON_0851 | TON_0849 | 789086 | 785823 | 25 |
| TU0485 | 791905 | - | -123 | P | TON_0854 | TON_0854 | TON_0854 | 791896 | 790922 | 9 |
| TU0486 | 792845 | + | 3 | P | TON_0857 | TON_0857 | TON_0857 | 792887 | 793216 | 42 |
| TU0487 | 794839 | + | 252 | P | TON_0860; TON_0861; TON_0862; TON_0863 | TON_0860 | TON_0863 | 794852 | 799166 | 13 |
| TU0488 | 800939 | + | 439 | P | TON_0866 | TON_0866 | TON_0866 | 800962 | 801477 | 23 |
| TU0489 | 801548 | + | 2883 | P | TON_0867 | TON_0867 | TON_0867 | 801560 | 801721 | 12 |
| TU0490 | 801659 | + | 41 | I | TON_0867 | TON_0867 | TON_0867 | 801560 | 801721 | - |
| TU0491 | 802315 | - | -5 | A | TON_nc033 | TON_nc033 | TON_nc033 | - | - | - |
| TU0492 | 802620 | + | 11 | P | TON_0870 | TON_0870 | TON_0870 | 802660 | 803010 | 40 |
| TU0493 | 802970 | + | 141 | P | TON_0871 | TON_0871 | TON_0871 | 803014 | 803538 | 44 |
| TU0494 | 804424 | - | -4 | P | TON_0873 | TON_0873 | TON_0873 | 804415 | 803936 | 9 |
| TU0495 | 804460 | + | 48 | P | TON_0874 | TON_0874 | TON_0874 | 804483 | 805007 | 23 |

| | | | | | | | | | | |
|---|---|---|---|---|---|---|---|---|---|---|
| TU0496 | 805990 | - | -139 | P | TON_0876 | TON_0876 | TON_0876 | 805979 | 805371 | 11 |
| TU0497 | 806707 | - | -1693 | P | TON_0877 | TON_0877 | TON_0877 | 806766 | 806173 | -59 |
| TU0498 | 806903 | + | 1168 | P | TON_0878; TON_0879; TON_0880; TON_0881; TON_0882; TON_0883; TON_0884; TON_0885; TON_0886; | TON_0878 | TON_0888 | 807102 | 815439 | 199 |
| TU0499 | 815652 | + | 17 | P | TON_0889 | TON_0889 | TON_0889 | 815677 | 816381 | 25 |
| TU0500 | 817328 | + | 26 | P | TON_0891 | TON_0891 | TON_0891 | 817328 | 818323 | 0 |
| TU0501 | 818787 | + | 161 | N | TON_nc034 | TON_nc034 | TON_nc034 | - | - | - |
| TU0502 | 831129 | - | -1122 | P | TON_0900 | TON_0900 | TON_0900 | 831103 | 829649 | 26 |
| TU0503 | 832525 | - | -12 | P | TON_0901 | TON_0901 | TON_0901 | 832498 | 831224 | 27 |
| TU0504 | 833345 | - | -37 | P | TON_0902 | TON_0902 | TON_0902 | 833624 | 832602 | -279 |
| TU0505 | 833362 | + | 5 | P | TON_0903 | TON_0903 | TON_0903 | 833434 | 834279 | 72 |
| TU0506 | 834371 | + | 43 | P | TON_0904 | TON_0904 | TON_0904 | 834406 | 836190 | 35 |
| TU0507 | 836350 | + | 6 | P | TON_0905; TON_0906; TON_0907; TON_0908; TON_0909 | TON_0905 | TON_0909 | 836374 | 840323 | 24 |
| TU0508 | 840388 | + | 16 | P | TON_0910 | TON_0910 | TON_0910 | 840401 | 841192 | 13 |
| TU0509 | 842216 | - | -50 | P | TON_0911 | TON_0911 | TON_0911 | 842208 | 841189 | 8 |
| TU0510 | 842331 | + | 64 | P | TON_0912 | TON_0912 | TON_0912 | 842343 | 843269 | 12 |
| TU0511 | 844804 | - | -10 | P | TON_0913 | TON_0913 | TON_0913 | 844696 | 843254 | 108 |
| TU0512 | 847301 | + | 4285 | P | TON_0917 | TON_0917 | TON_0917 | 847311 | 847709 | 10 |
| TU0513 | 848062 | + | 111 | P | TON_0918 | TON_0918 | TON_0918 | 848076 | 848615 | 14 |
| TU0514 | 848912 | + | 829 | P | TON_0919 | TON_0919 | TON_0919 | 848935 | 849528 | 23 |
| TU0515 | 849833 | + | 74 | P | TON_0921 | TON_0921 | TON_0921 | 849845 | 850252 | 12 |
| TU0516 | 850356 | + | 29 | P | TON_0922 | TON_0922 | TON_0922 | 850366 | 850470 | 10 |
| TU0517 | 851449 | + | 255 | P | TON_0924 | TON_0924 | TON_0924 | 851459 | 852040 | 10 |
| TU0518 | 852503 | + | 5 | P | TON_0926 | TON_0926 | TON_0926 | 852513 | 853088 | 10 |
| TU0519 | 853553 | - | -52 | P | TON_0927 | TON_0927 | TON_0927 | 853543 | 853085 | 10 |
| TU0520 | 853642 | + | 72 | P | TON_0928 | TON_0928 | TON_0928 | 853649 | 854005 | 7 |
| TU0521 | 857164 | + | 204 | P | TON_0930 | TON_0930 | TON_0930 | 857189 | 857464 | 25 |
| TU0522 | 857565 | + | 107 | P | TON_0931 | TON_0931 | TON_0931 | 857576 | 858205 | 11 |
| TU0523 | 858241 | + | 27001 | P | TON_0932 | TON_0932 | TON_0932 | 858255 | 858800 | 14 |
| TU0524 | 859325 | - | -73 | P | TON_0933 | TON_0933 | TON_0933 | 859315 | 858797 | 10 |
| TU0525 | 859360 | + | 111 | P | TON_0934 | TON_0934 | TON_0934 | 859371 | 860441 | 11 |

| | | | | | | | | | | |
|---|---|---|---|---|---|---|---|---|---|---|
| TU0526 | 860675 | - | -228 | P | TON_0935 | TON_0935 | TON_0935 | 860674 | 860393 | 1 |
| TU0527 | 861993 | + | 31 | P | TON_0937 | TON_0937 | TON_0937 | 861993 | 864770 | 0 |
| TU0528 | 865913 | - | -4 | P | TON_0938 | TON_0938 | TON_0938 | 865903 | 864851 | 10 |
| TU0529 | 865963 | + | 48 | P | TON_0939; TON_0940; TON_0941; TON_0942 | TON_0939 | TON_0942 | 866053 | 868065 | 90 |
| TU0530 | 869668 | - | -32 | P | TON_0943 | TON_0943 | TON_0943 | 869668 | 868088 | 0 |
| TU0531 | 869759 | + | 11 | P | TON_0944 | TON_0944 | TON_0944 | 869735 | 870481 | -24 |
| TU0532 | 871608 | + | 20 | P | TON_0946 | TON_0946 | TON_0946 | 871634 | 872848 | 26 |
| TU0533 | 876054 | - | -134 | P | TON_0948 | TON_0948 | TON_0948 | 876041 | 874248 | 13 |
| TU0534 | 877119 | - | -172 | P | TON_0951 | TON_0951 | TON_0951 | 877110 | 876598 | 9 |
| TU0535 | 877642 | - | -213 | P | TON_0953; TON_0952 | TON_0953 | TON_0952 | 877624 | 877200 | 18 |
| TU0536 | 878114 | - | -3359 | P | TON_0954 | TON_0954 | TON_0954 | 878074 | 877703 | 40 |
| TU0537 | 878873 | - | -28 | P | TON_0955 | TON_0955 | TON_0955 | 878863 | 878252 | 10 |
| TU0538 | 884548 | + | 1641 | P | TON_0961 | TON_0961 | TON_0961 | 884685 | 885977 | 137 |
| TU0539 | 886393 | + | 7 | N | TON_nc035 | TON_nc035 | TON_nc035 | - | - | - |
| TU0540 | 889005 | - | -17 | P | TON_0964 | TON_0964 | TON_0964 | 888995 | 888060 | 10 |
| TU0541 | 890103 | - | -76 | P | TON_0966; TON_0965 | TON_0966 | TON_0965 | 890055 | 889034 | 48 |
| TU0542 | 893143 | - | -3 | P | TON_0968 | TON_0968 | TON_0968 | 893128 | 890696 | 15 |
| TU0543 | 893246 | + | 9 | P | TON_0969 | TON_0969 | TON_0969 | 893255 | 895150 | 9 |
| TU0544 | 895326 | + | 4 | P | TON_0970 | TON_0970 | TON_0970 | 895326 | 895916 | 0 |
| TU0545 | 895989 | + | 4109 | P | TON_0971; TON_0972 | TON_0971 | TON_0972 | 896097 | 897076 | 108 |
| TU0546 | 897646 | + | 98 | P | TON_0974; TON_0975 | TON_0974 | TON_0975 | 897647 | 899196 | 1 |
| TU0547 | 897953 | + | 80 | I | TON_0974; TON_0975 | TON_0974 | TON_0975 | 897647 | 899196 | - |
| TU0548 | 899829 | - | -472 | P | TON_0976 | TON_0976 | TON_0976 | 899822 | 899193 | 7 |
| TU0549 | 900284 | - | -3 | P | TON_0977 | TON_0977 | TON_0977 | 900281 | 899889 | 3 |
| TU0550 | 900366 | + | 602 | P | TON_0978 | TON_0978 | TON_0978 | 900376 | 901167 | 10 |
| TU0551 | 900486 | - | -9 | A | TON_nc036 | TON_nc036 | TON_nc036 | - | - | - |
| TU0552 | 905459 | - | -73 | P | TON_0981 | TON_0981 | TON_0981 | 905448 | 904321 | 11 |
| TU0553 | 905563 | + | 3 | P | TON_0982 | TON_0982 | TON_0982 | 905572 | 906690 | 9 |
| TU0554 | 906720 | + | 14 | P | TON_0983 | TON_0983 | TON_0983 | 906746 | 908197 | 26 |
| TU0555 | 908306 | + | 110 | P | TON_0984 | TON_0984 | TON_0984 | 908345 | 910189 | 39 |
| TU0556 | 910119 | + | 19 | P | TON_0985 | TON_0985 | TON_0985 | 910200 | 911264 | 81 |
| TU0557 | 911289 | + | 55 | P | TON_0986 | TON_0986 | TON_0986 | 911299 | 911898 | 10 |

| | | | | | | | | | | |
|---|---|---|---|---|---|---|---|---|---|---|
| TU0558 | 911957 | + | 3 | P | TON_0987 | TON_0987 | TON_0987 | 911967 | 913985 | 10 |
| TU0559 | 916869 | - | -107 | P | TON_0989 | TON_0989 | TON_0989 | 916838 | 914700 | 31 |
| TU0560 | 916973 | + | 53 | P | TON_0990 | TON_0990 | TON_0990 | 916982 | 918226 | 9 |
| TU0561 | 918382 | + | 2 | A | TON_nc037 | TON_nc037 | TON_nc037 | - | - | - |
| TU0562 | 920757 | - | -364 | P | TON_0994; TON_0993; TON_0992; TON_0991 | TON_0994 | TON_0991 | 920747 | 918184 | 10 |
| TU0563 | 923164 | - | -237 | P | TON_0998; TON_0997 | TON_0998 | TON_0997 | 923152 | 921973 | 12 |
| TU0564 | 924454 | + | 22 | P | TON_1000 | TON_1000 | TON_1000 | 924458 | 925210 | 4 |
| TU0565 | 925285 | + | 41 | P | TON_1001; TON_1002 | TON_1001 | TON_1002 | 925314 | 927489 | 29 |
| TU0566 | 928675 | + | 21 | N | TON_nc038 | TON_nc038 | TON_nc038 | - | - | - |
| TU0567 | 928767 | - | -83 | P | TON_1004 | TON_1004 | TON_1004 | 928674 | 927853 | 93 |
| TU0568 | 928954 | + | 62 | S | TON_1005 | TON_1005 | TON_1005 | 929227 | 930207 | - |
| TU0569 | 929039 | + | 107 | P | TON_1005 | TON_1005 | TON_1005 | 929227 | 930207 | 188 |
| TU0570 | 930889 | - | -108 | P | TON_1006 | TON_1006 | TON_1006 | 930889 | 930299 | 0 |
| TU0571 | 932982 | - | -44 | P | TON_1009 | TON_1009 | TON_1009 | 932973 | 932647 | 9 |
| TU0572 | 933633 | - | -3997 | P | TON_1010 | TON_1010 | TON_1010 | 933623 | 933015 | 10 |
| TU0573 | 933709 | + | 116 | P | TON_1011 | TON_1011 | TON_1011 | 933720 | 934433 | 11 |
| TU0574 | 934510 | + | 11 | P | TON_1012 | TON_1012 | TON_1012 | 934533 | 935912 | 23 |
| TU0575 | 937684 | - | -217 | P | TON_1014 | TON_1014 | TON_1014 | 937656 | 936763 | 28 |
| TU0576 | 938482 | + | 586 | N | TON_nc039 | TON_nc039 | TON_nc039 | - | - | - |
| TU0577 | 938893 | + | 3 | P | TON_1016 | TON_1016 | TON_1016 | 939053 | 940156 | 160 |
| TU0578 | 953574 | + | 12 | P | TON_1032 | TON_1032 | TON_1032 | 953602 | 954648 | 28 |
| TU0579 | 954820 | + | 938 | P | TON_1033 | TON_1033 | TON_1033 | 954845 | 955219 | 25 |
| TU0580 | 956993 | - | -3 | A | TON_nc040 | TON_nc040 | TON_nc040 | - | - | - |
| TU0581 | 958375 | + | 15 | P | TON_1038 | TON_1038 | TON_1038 | 958400 | 959104 | 25 |
| TU0582 | 959457 | - | -37 | P | TON_1039 | TON_1039 | TON_1039 | 959497 | 959117 | -40 |
| TU0583 | 959496 | + | 28 | P | TON_1040 | TON_1040 | TON_1040 | 959421 | 961421 | -75 |
| TU0584 | 962974 | + | 76 | P | TON_2001 | TON_2001 | TON_2001 | 962981 | 963058 | - |
| TU0585 | 965516 | - | -192 | P | TON_1043 | TON_1043 | TON_1043 | 965516 | 964335 | 0 |
| TU0586 | 965981 | - | -28 | P | TON_2020 | TON_2020 | TON_2020 | 965970 | 965863 | - |
| TU0587 | 968226 | - | -482 | P | TON_1046; TON_1045; TON_1044 | TON_1046 | TON_1044 | 968217 | 966022 | 9 |
| TU0588 | 968277 | + | 111 | P | TON_1047 | TON_1047 | TON_1047 | 968300 | 969331 | 23 |
| TU0589 | 969186 | + | 397 | P | TON_1048 | TON_1048 | TON_1048 | 969344 | 969793 | 158 |

| | | | | | | | | | | |
|---|---|---|---|---|---|---|---|---|---|---|
| TU0590 | 972265 | - | -361 | P | TON_1051 | TON_1051 | TON_1051 | 972255 | 971488 | 10 |
| TU0591 | 972316 | + | 3 | P | TON_1052 | TON_1052 | TON_1052 | 972316 | 972801 | 0 |
| TU0592 | 972916 | - | -319234 | N | TON_nc041 | TON_nc041 | TON_nc041 | - | - | - |
| TU0593 | 974503 | - | -633 | P | TON_1055; TON_1054 | TON_1055 | TON_1054 | 974502 | 973346 | 1 |
| TU0594 | 974783 | - | -313 | P | TON_1056 | TON_1056 | TON_1056 | 974783 | 974484 | 0 |
| TU0595 | 974864 | + | 72 | P | TON_1057 | TON_1057 | TON_1057 | 974875 | 976200 | 11 |
| TU0596 | 978647 | + | 1671 | P | TON_1062 | TON_1062 | TON_1062 | 978673 | 980184 | 26 |
| TU0597 | 980736 | - | -126 | P | TON_1063 | TON_1063 | TON_1063 | 980725 | 980336 | 11 |
| TU0598 | 980787 | + | 37 | P | TON_1064 | TON_1064 | TON_1064 | 980787 | 981230 | 0 |
| TU0599 | 981216 | + | 131 | P | TON_1065 | TON_1065 | TON_1065 | 981227 | 981994 | 11 |
| TU0600 | 982246 | - | -6553 | P | TON_1066 | TON_1066 | TON_1066 | 982236 | 981997 | 10 |
| TU0601 | 983391 | + | 15 | P | TON_1068 | TON_1068 | TON_1068 | 983401 | 984144 | 10 |
| TU0602 | 986495 | - | -106 | P | TON_1069 | TON_1069 | TON_1069 | 986495 | 984126 | 0 |
| TU0603 | 986547 | + | 2 | P | TON_1070 | TON_1070 | TON_1070 | 986545 | 986970 | -2 |
| TU0604 | 991268 | + | 1357 | A | TON_nc042 | TON_nc042 | TON_nc042 | - | - | - |
| TU0605 | 991725 | - | -1403 | P | TON_1073 | TON_1073 | TON_1073 | 991633 | 991250 | 92 |
| TU0606 | 993227 | - | -162 | P | TON_1075 | TON_1075 | TON_1075 | 993216 | 992257 | 11 |
| TU0607 | 993701 | - | -62 | P | TON_1076 | TON_1076 | TON_1076 | 993691 | 993251 | 10 |
| TU0608 | 993813 | + | 65 | P | TON_1077 | TON_1077 | TON_1077 | 993807 | 994457 | -6 |
| TU0609 | 998003 | - | -13 | N | TON_nc043 | TON_nc043 | TON_nc043 | - | - | - |
| TU0610 | 998043 | + | 6 | N | TON_nc044 | TON_nc044 | TON_nc044 | - | - | - |
| TU0611 | 999027 | - | -274 | P | TON_1078 | TON_1078 | TON_1078 | 999027 | 998473 | 0 |
| TU0612 | 999076 | + | 78 | N | TON_nc045 | TON_nc045 | TON_nc045 | - | - | - |
| TU0613 | 999361 | - | -4 | P | TON_1079 | TON_1079 | TON_1079 | 999333 | 999169 | 28 |
| TU0614 | 1001256 | - | -168 | P | TON_1080 | TON_1080 | TON_1080 | 1001204 | 999396 | 52 |
| TU0615 | 1001306 | + | 91 | P | TON_1081 | TON_1081 | TON_1081 | 1001316 | 1002056 | 10 |
| TU0616 | 1002875 | + | 8 | P | TON_1083 | TON_1083 | TON_1083 | 1002918 | 1004360 | 43 |
| TU0617 | 1004492 | + | 56 | P | TON_1084 | TON_1084 | TON_1084 | 1004504 | 1005709 | 12 |
| TU0618 | 1007873 | + | 457 | P | TON_1087 | TON_1087 | TON_1087 | 1007910 | 1009091 | 37 |
| TU0619 | 1009532 | - | -16 | P | TON_1088 | TON_1088 | TON_1088 | 1009522 | 1009103 | 10 |
| TU0620 | 1009603 | + | 302 | P | TON_1089 | TON_1089 | TON_1089 | 1009604 | 1010308 | 1 |
| TU0621 | 1011700 | - | -79 | P | TON_1090 | TON_1090 | TON_1090 | 1011688 | 1010315 | 12 |

| | | | | | | | | | | |
|---|---|---|---|---|---|---|---|---|---|---|
| TU0622 | 1012978 | - | -17 | P | TON_1091 | TON_1091 | TON_1091 | 1012969 | 1011767 | 9 |
| TU0623 | 1013195 | - | -500 | P | TON_1092 | TON_1092 | TON_1092 | 1013185 | 1013033 | 10 |
| TU0624 | 1013316 | + | 383 | P | TON_1093; TON_1094 | TON_1093 | TON_1094 | 1013262 | 1016590 | -54 |
| TU0625 | 1016643 | + | 27 | P | TON_1095 | TON_1095 | TON_1095 | 1016668 | 1016985 | 25 |
| TU0626 | 1019219 | - | -174 | P | TON_1097 | TON_1097 | TON_1097 | 1019194 | 1017809 | 25 |
| TU0627 | 1019359 | + | 319 | P | TON_1098 | TON_1098 | TON_1098 | 1019409 | 1019834 | 50 |
| TU0628 | 1019782 | + | 4 | P | TON_1099 | TON_1099 | TON_1099 | 1019834 | 1020859 | 52 |
| TU0629 | 1023430 | - | -10 | P | TON_1104; TON_1103 | TON_1104 | TON_1103 | 1023397 | 1022525 | 33 |
| TU0630 | 1023571 | + | 28 | P | TON_1105 | TON_1105 | TON_1105 | 1023584 | 1024183 | 13 |
| TU0631 | 1025633 | - | -43 | P | TON_1109; TON_1108 | TON_1109 | TON_1108 | 1025601 | 1025160 | 32 |
| TU0632 | 1027428 | - | -5 | P | TON_1110 | TON_1110 | TON_1110 | 1027410 | 1025722 | 18 |
| TU0633 | 1028443 | + | 255 | P | TON_1112 | TON_1112 | TON_1112 | 1028454 | 1028939 | 11 |
| TU0634 | 1029011 | + | 10 | P | TON_1113 | TON_1113 | TON_1113 | 1029039 | 1029530 | 28 |
| TU0635 | 1034841 | - | -2 | P | TON_1121 | TON_1121 | TON_1121 | 1034830 | 1034615 | 11 |
| TU0636 | 1034884 | + | 109 | P | TON_1122 | TON_1122 | TON_1122 | 1034893 | 1035720 | 9 |
| TU0637 | 1036084 | - | -25 | P | TON_1123 | TON_1123 | TON_1123 | 1036074 | 1035739 | 10 |
| TU0638 | 1037326 | - | -35 | P | TON_1125 | TON_1125 | TON_1125 | 1037326 | 1036865 | 0 |
| TU0639 | 1038004 | + | 34 | P | TON_1128 | TON_1128 | TON_1128 | 1038013 | 1038576 | 9 |
| TU0640 | 1039534 | + | 707 | P | TON_1130; TON_1131; TON_1132; TON_1133; TON_1134; TON_1135; TON_1136; TON_1137; TON_1138; TON_1139; TON_1140; TON_1141; | TON_1130 | TON_1142 | 1039447 | 1049714 | -87 |
| TU0641 | 1050406 | - | -8 | P | TON_1143 | TON_1143 | TON_1143 | 1050397 | 1049954 | 9 |
| TU0642 | 1052552 | - | -327 | P | TON_1147; TON_1146 | TON_1147 | TON_1146 | 1052552 | 1051747 | 0 |
| TU0643 | 1053544 | + | 5 | P | TON_1149 | TON_1149 | TON_1149 | 1053634 | 1054575 | 90 |
| TU0644 | 1055988 | + | 1 | P | TON_1152 | TON_1152 | TON_1152 | 1056098 | 1057888 | 110 |
| TU0645 | 1058623 | - | -110 | P | TON_1154; TON_1153 | TON_1154 | TON_1153 | 1058611 | 1057902 | 12 |
| TU0646 | 1059059 | - | -14 | P | TON_1155; TON_1154; TON_1153 | TON_1155 | TON_1153 | 1059059 | 1057902 | 0 |
| TU0647 | 1065624 | + | 106 | P | TON_1166 | TON_1166 | TON_1166 | 1065629 | 1067236 | 5 |
| TU0648 | 1067177 | + | 274 | P | TON_1167; TON_1168 | TON_1167 | TON_1168 | 1067233 | 1068339 | 56 |
| TU0649 | 1069143 | - | -31 | P | TON_1169 | TON_1169 | TON_1169 | 1069133 | 1068423 | 10 |
| TU0650 | 1070494 | - | -1 | P | TON_1172; TON_1171 | TON_1172 | TON_1171 | 1070463 | 1069555 | 31 |
| TU0651 | 1070547 | + | 39 | P | TON_1173; TON_1174 | TON_1173 | TON_1174 | 1070557 | 1071864 | 10 |

| | | | | | | | | | | |
|---|---|---|---|---|---|---|---|---|---|---|
| TU0652 | 1071899 | + | 77 | N | TON_nc046 | TON_nc046 | TON_nc046 | - | - | - |
| TU0653 | 1072676 | - | -18 | P | TON_1175 | TON_1175 | TON_1175 | 1072667 | 1072044 | 9 |
| TU0654 | 1082328 | - | -166 | P | TON_1184 | TON_1184 | TON_1184 | 1082288 | 1081812 | 40 |
| TU0655 | 1083461 | + | 30 | A | TON_nc047 | TON_nc047 | TON_nc047 | - | - | - |
| TU0656 | 1087986 | - | -20 | P | TON_1191; TON_1190 | TON_1191 | TON_1190 | 1087893 | 1086601 | 93 |
| TU0657 | 1088223 | + | 130 | P | TON_1192 | TON_1192 | TON_1192 | 1088246 | 1089325 | 23 |
| TU0658 | 1091015 | - | -15 | P | TON_1194 | TON_1194 | TON_1194 | 1090993 | 1089767 | 22 |
| TU0659 | 1091177 | + | 2 | P | TON_1195; TON_1196; TON_1197; TON_1198; TON_1199; TON_1200; | TON_1195 | TON_1202 | 1091237 | 1099995 | 60 |
| TU0660 | 1100648 | + | 29 | P | TON_1204 | TON_1204 | TON_1204 | 1100803 | 1101828 | 155 |
| TU0661 | 1103195 | + | 1444 | P | TON_1206 | TON_1206 | TON_1206 | 1103204 | 1103950 | 9 |
| TU0662 | 1104996 | - | -15 | P | TON_1207 | TON_1207 | TON_1207 | 1104991 | 1103915 | 5 |
| TU0663 | 1105243 | + | 206 | P | TON_1208; TON_1209 | TON_1208 | TON_1209 | 1105241 | 1107188 | -2 |
| TU0664 | 1108354 | - | -53 | P | TON_1210 | TON_1210 | TON_1210 | 1108341 | 1107193 | 13 |
| TU0665 | 1109156 | - | -3 | P | TON_1211 | TON_1211 | TON_1211 | 1109140 | 1108571 | 16 |
| TU0666 | 1109185 | + | 351 | P | TON_1212 | TON_1212 | TON_1212 | 1109195 | 1110460 | 10 |
| TU0667 | 1110618 | + | 11 | P | TON_1213 | TON_1213 | TON_1213 | 1110628 | 1111566 | 10 |
| TU0668 | 1111564 | - | -6 | A | TON_nc048 | TON_nc048 | TON_nc048 | - | - | - |
| TU0669 | 1113154 | + | 129 | P | TON_1217 | TON_1217 | TON_1217 | 1113345 | 1113902 | 191 |
| TU0670 | 1113320 | - | -100 | P | TON_1216; TON_1215 | TON_1216 | TON_1215 | 1113293 | 1112422 | 27 |
| TU0671 | 1115926 | - | -10 | P | TON_1220; TON_1219; TON_1218 | TON_1220 | TON_1218 | 1115863 | 1113903 | 63 |
| TU0672 | 1115979 | + | 317 | P | TON_1221 | TON_1221 | TON_1221 | 1115979 | 1117073 | 0 |
| TU0673 | 1117559 | + | 9 | P | TON_1223 | TON_1223 | TON_1223 | 1117568 | 1118005 | 9 |
| TU0674 | 1119017 | - | -54 | P | TON_1225 | TON_1225 | TON_1225 | 1119004 | 1118357 | 13 |
| TU0675 | 1120484 | - | -1 | P | TON_1227 | TON_1227 | TON_1227 | 1120474 | 1119842 | 10 |
| TU0676 | 1121584 | - | -6 | I | TON_1228 | TON_1228 | TON_1228 | 1121835 | 1120516 | - |
| TU0677 | 1121988 | - | -4 | P | TON_1228 | TON_1228 | TON_1228 | 1121835 | 1120516 | 153 |
| TU0678 | 1122508 | - | -78 | P | TON_1229 | TON_1229 | TON_1229 | 1122508 | 1122089 | 0 |
| TU0679 | 1122585 | + | 491 | P | TON_1230 | TON_1230 | TON_1230 | 1122619 | 1122822 | 34 |
| TU0680 | 1123497 | - | -172 | P | TON_1231 | TON_1231 | TON_1231 | 1123473 | 1122877 | 24 |
| TU0681 | 1123577 | + | 2 | P | TON_1232 | TON_1232 | TON_1232 | 1123587 | 1124756 | 10 |
| TU0682 | 1126780 | - | -6619 | P | TON_1234 | TON_1234 | TON_1234 | 1126763 | 1125429 | 17 |

| | | | | | | | | | | |
|---|---|---|---|---|---|---|---|---|---|---|
| TU0683 | 1127117 | + | 82 | P | TON_1235 | TON_1235 | TON_1235 | 1127155 | 1127358 | 38 |
| TU0684 | 1129146 | - | -26 | P | TON_1238; TON_1237 | TON_1238 | TON_1237 | 1129120 | 1127891 | 26 |
| TU0685 | 1129229 | + | 5 | P | TON_1239 | TON_1239 | TON_1239 | 1129244 | 1130083 | 15 |
| TU0686 | 1130122 | + | 66 | P | TON_1240; TON_1241 | TON_1240 | TON_1241 | 1130184 | 1131051 | 62 |
| TU0687 | 1133284 | - | -4 | P | TON_1243; TON_1242 | TON_1243 | TON_1242 | 1133274 | 1131041 | 10 |
| TU0688 | 1134834 | - | -8 | P | TON_1245; TON_1244 | TON_1245 | TON_1244 | 1134845 | 1133359 | -11 |
| TU0689 | 1134996 | + | 9 | P | TON_1246 | TON_1246 | TON_1246 | 1135197 | 1135499 | 201 |
| TU0690 | 1136226 | - | -2 | P | TON_1247 | TON_1247 | TON_1247 | 1136215 | 1135538 | 11 |
| TU0691 | 1136262 | + | 179 | P | TON_1248 | TON_1248 | TON_1248 | 1136309 | 1137586 | 47 |
| TU0692 | 1136321 | + | 164 | S | TON_1248 | TON_1248 | TON_1248 | 1136309 | 1137586 | - |
| TU0693 | 1137628 | + | 35 | P | TON_1249 | TON_1249 | TON_1249 | 1137647 | 1138795 | 19 |
| TU0694 | 1138848 | + | 54 | P | TON_1250; TON_1251; TON_1252; TON_1253 | TON_1250 | TON_1253 | 1138860 | 1141089 | 12 |
| TU0695 | 1143589 | + | 20 | N | TON_nc049 | TON_nc049 | TON_nc049 | - | - | - |
| TU0696 | 1143857 | - | -2790 | P | TON_1257 | TON_1257 | TON_1257 | 1143828 | 1143637 | 29 |
| TU0697 | 1144028 | + | 21 | P | TON_1258 | TON_1258 | TON_1258 | 1144038 | 1144445 | 10 |
| TU0698 | 1144514 | + | 1 | P | TON_1259 | TON_1259 | TON_1259 | 1144531 | 1145709 | 17 |
| TU0699 | 1148205 | - | -21 | P | TON_2018 | TON_2018 | TON_2018 | 1148205 | 1148129 | - |
| TU0700 | 1149033 | - | -71 | P | TON_1263 | TON_1263 | TON_1263 | 1149018 | 1148299 | 15 |
| TU0701 | 1150345 | - | -714 | P | TON_nc050 | TON_nc050 | TON_nc050 | - | - | - |
| TU0702 | 1156188 | + | 64 | P | TON_1270 | TON_1270 | TON_1270 | 1156196 | 1156873 | 8 |
| TU0703 | 1157007 | + | 30 | P | TON_1271 | TON_1271 | TON_1271 | 1157017 | 1158336 | 10 |
| TU0704 | 1161986 | + | 40 | P | TON_1278 | TON_1278 | TON_1278 | 1161987 | 1162529 | 1 |
| TU0705 | 1163527 | - | -7 | I | TON_1279 | TON_1279 | TON_1279 | 1163751 | 1162513 | - |
| TU0706 | 1163752 | - | -13 | P | TON_1279 | TON_1279 | TON_1279 | 1163751 | 1162513 | 1 |
| TU0707 | 1163802 | + | 14 | N | TON_nc051 | TON_nc051 | TON_nc051 | - | - | - |
| TU0708 | 1164994 | - | -42 | P | TON_1280 | TON_1280 | TON_1280 | 1164986 | 1163925 | 8 |
| TU0709 | 1167732 | - | -9 | P | TON_1282; TON_1281 | TON_1282 | TON_1281 | 1167701 | 1165050 | 31 |
| TU0710 | 1169113 | - | -5 | P | TON_1283 | TON_1283 | TON_1283 | 1169086 | 1167920 | 27 |
| TU0711 | 1169190 | + | 30 | P | TON_1284 | TON_1284 | TON_1284 | 1169205 | 1169657 | 15 |
| TU0712 | 1169710 | + | 68 | P | TON_1285 | TON_1285 | TON_1285 | 1169734 | 1170234 | 24 |
| TU0713 | 1170302 | + | 3 | P | TON_1286 | TON_1286 | TON_1286 | 1170314 | 1171039 | 12 |
| TU0714 | 1171070 | + | 10 | P | TON_1287; TON_1288; TON_1289 | TON_1287 | TON_1289 | 1171096 | 1172635 | 26 |

| | | | | | | | | | | |
|---|---|---|---|---|---|---|---|---|---|---|
| TU0715 | 1172909 | - | -45 | P | TON_1290 | TON_1290 | TON_1290 | 1172900 | 1172619 | 9 |
| TU0716 | 1174092 | + | 21 | P | TON_1293 | TON_1293 | TON_1293 | 1174092 | 1174934 | 0 |
| TU0717 | 1175705 | - | -46 | P | TON_1973 | TON_1973 | TON_1973 | 1175694 | 1174861 | 11 |
| TU0718 | 1177065 | - | -50 | P | TON_1295 | TON_1295 | TON_1295 | 1177013 | 1175757 | 52 |
| TU0719 | 1177232 | + | 3397 | P | TON_1296 | TON_1296 | TON_1296 | 1177252 | 1178220 | 20 |
| TU0720 | 1180408 | - | -25 | P | TON_1298 | TON_1298 | TON_1298 | 1180382 | 1179126 | 26 |
| TU0721 | 1180652 | + | 1 | P | TON_1299; TON_1300 | TON_1299 | TON_1300 | 1180681 | 1182629 | 29 |
| TU0722 | 1182769 | + | 248 | P | TON_1301; TON_1302; TON_1303 | TON_1301 | TON_1303 | 1182802 | 1185431 | 33 |
| TU0723 | 1185844 | - | -60 | P | TON_1304 | TON_1304 | TON_1304 | 1185830 | 1185537 | 14 |
| TU0724 | 1186353 | - | -75 | P | TON_1305 | TON_1305 | TON_1305 | 1186352 | 1185849 | 1 |
| TU0725 | 1186403 | + | 47 | P | TON_1306 | TON_1306 | TON_1306 | 1186423 | 1186956 | 20 |
| TU0726 | 1187377 | - | -2140 | P | TON_1307 | TON_1307 | TON_1307 | 1187368 | 1186961 | 9 |
| TU0727 | 1188550 | + | 118 | P | TON_1309; TON_1310 | TON_1309 | TON_1310 | 1188584 | 1190190 | 34 |
| TU0728 | 1194156 | - | -242 | P | TON_1313; TON_1312; TON_1311 | TON_1313 | TON_1311 | 1194147 | 1190205 | 9 |
| TU0729 | 1194322 | + | 74 | P | TON_1314 | TON_1314 | TON_1314 | 1194334 | 1195008 | 12 |
| TU0730 | 1195044 | + | 57 | P | TON_1315 | TON_1315 | TON_1315 | 1195054 | 1196052 | 10 |
| TU0731 | 1196285 | - | -283 | P | TON_1316 | TON_1316 | TON_1316 | 1196272 | 1196126 | 13 |
| TU0732 | 1197143 | - | -1340 | P | TON_1317 | TON_1317 | TON_1317 | 1197133 | 1196321 | 10 |
| TU0733 | 1198544 | - | -66 | P | TON_1319; TON_1318 | TON_1319 | TON_1318 | 1198534 | 1197177 | 10 |
| TU0734 | 1201380 | - | -14 | P | TON_1322 | TON_1322 | TON_1322 | 1201367 | 1200519 | 13 |
| TU0735 | 1201955 | - | -24 | P | TON_1323 | TON_1323 | TON_1323 | 1201937 | 1201419 | 18 |
| TU0736 | 1203205 | - | -10 | P | TON_1324 | TON_1324 | TON_1324 | 1203205 | 1202057 | 0 |
| TU0737 | 1203284 | + | 68 | P | TON_1325 | TON_1325 | TON_1325 | 1203284 | 1203691 | 0 |
| TU0738 | 1207651 | + | 201 | P | TON_1334 | TON_1334 | TON_1334 | 1207661 | 1208065 | 10 |
| TU0739 | 1208132 | + | 24 | P | TON_1335 | TON_1335 | TON_1335 | 1208143 | 1208481 | 11 |
| TU0740 | 1208556 | - | -695 | N | TON_nc052 | TON_nc052 | TON_nc052 | - | - | - |
| TU0741 | 1211115 | - | -4 | P | TON_1337; TON_1336 | TON_1337 | TON_1336 | 1211076 | 1208759 | 39 |
| TU0742 | 1211230 | + | 70 | P | TON_1338 | TON_1338 | TON_1338 | 1211242 | 1211454 | 12 |
| TU0743 | 1212831 | - | -4 | P | TON_1340; TON_1339 | TON_1340 | TON_1339 | 1212821 | 1211441 | 10 |
| TU0744 | 1212868 | + | 141 | P | TON_1341 | TON_1341 | TON_1341 | 1212879 | 1213223 | 11 |
| TU0745 | 1213495 | - | -694 | P | TON_1342 | TON_1342 | TON_1342 | 1213469 | 1213224 | 26 |
| TU0746 | 1213600 | + | 98 | P | TON_1343 | TON_1343 | TON_1343 | 1213608 | 1215788 | 8 |

| | | | | | | | | | | |
|---|---|---|---|---|---|---|---|---|---|---|
| TU0747 | 1215829 | + | 16 | P | TON_1344 | TON_1344 | TON_1344 | 1215836 | 1216393 | 7 |
| TU0748 | 1217200 | - | -3 | P | TON_1345 | TON_1345 | TON_1345 | 1217188 | 1216379 | 12 |
| TU0749 | 1221385 | - | -39 | P | TON_1352; TON_1351; TON_1350; TON_1349 | TON_1352 | TON_1349 | 1221360 | 1219865 | 25 |
| TU0750 | 1221950 | - | -30 | N | TON_nc053 | TON_nc053 | TON_nc053 | - | - | - |
| TU0751 | 1222003 | + | 8 | P | TON_1353 | TON_1353 | TON_1353 | 1222015 | 1223142 | 12 |
| TU0752 | 1222419 | + | 8 | I | TON_1353 | TON_1353 | TON_1353 | 1222015 | 1223142 | - |
| TU0753 | 1223126 | - | -554 | A | TON_nc054 | TON_nc054 | TON_nc054 | - | - | - |
| TU0754 | 1223327 | + | 163 | P | TON_1354 | TON_1354 | TON_1354 | 1223352 | 1225169 | 25 |
| TU0755 | 1227479 | + | 5 | P | TON_1356 | TON_1356 | TON_1356 | 1227491 | 1229251 | 12 |
| TU0756 | 1231004 | - | -520 | P | TON_1358; TON_1357 | TON_1358 | TON_1357 | 1230973 | 1229253 | 31 |
| TU0757 | 1231083 | + | 74 | P | TON_1359 | TON_1359 | TON_1359 | 1231033 | 1231824 | -50 |
| TU0758 | 1232539 | + | 39 | P | TON_1361 | TON_1361 | TON_1361 | 1232550 | 1232753 | 11 |
| TU0759 | 1234049 | - | -23 | I | TON_1362 | TON_1362 | TON_1362 | 1234110 | 1232938 | - |
| TU0760 | 1234121 | - | -19 | P | TON_1362 | TON_1362 | TON_1362 | 1234110 | 1232938 | 11 |
| TU0761 | 1235326 | - | -10 | P | TON_1363 | TON_1363 | TON_1363 | 1235316 | 1234414 | 10 |
| TU0762 | 1236566 | + | 7 | P | TON_1366 | TON_1366 | TON_1366 | 1236618 | 1237940 | 52 |
| TU0763 | 1236573 | - | -7 | P | TON_1365 | TON_1365 | TON_1365 | 1236562 | 1235654 | 11 |
| TU0764 | 1240041 | - | -2 | P | TON_1370; TON_1369; TON_1368 | TON_1370 | TON_1368 | 1240007 | 1238308 | 34 |
| TU0765 | 1240156 | + | 164 | P | TON_2003 | TON_2003 | TON_2003 | 1240162 | 1240239 | - |
| TU0766 | 1241778 | - | -3 | P | TON_1372 | TON_1372 | TON_1372 | 1241768 | 1240974 | 10 |
| TU0767 | 1242126 | + | 470 | P | TON_1373 | TON_1373 | TON_1373 | 1242126 | 1242857 | 0 |
| TU0768 | 1243665 | - | -168 | P | TON_1374 | TON_1374 | TON_1374 | 1243653 | 1242868 | 12 |
| TU0769 | 1246715 | - | -136 | N | TON_nc055 | TON_nc055 | TON_nc055 | 1246635 | 1246171 | - |
| TU0770 | 1246830 | + | 602 | P | TON_1378 | TON_1378 | TON_1378 | 1246841 | 1247944 | 11 |
| TU0771 | 1248212 | - | -669 | A | TON_nc056 | TON_nc056 | TON_nc056 | - | - | - |
| TU0772 | 1248288 | + | 8 | P | TON_1379; TON_1380 | TON_1379 | TON_1380 | 1248192 | 1252192 | -96 |
| TU0773 | 1253617 | - | -148 | N | TON_nc057 | TON_nc057 | TON_nc057 | - | - | - |
| TU0774 | 1255533 | + | 2 | P | TON_1382 | TON_1382 | TON_1382 | 1255554 | 1256738 | 21 |
| TU0775 | 1259091 | - | -237 | P | TON_1384 | TON_1384 | TON_1384 | 1259065 | 1258589 | 26 |
| TU0776 | 1259223 | + | 468 | P | TON_1385 | TON_1385 | TON_1385 | 1259299 | 1260495 | 76 |
| TU0777 | 1259282 | + | 499 | S | TON_1385 | TON_1385 | TON_1385 | 1259299 | 1260495 | - |
| TU0778 | 1261685 | + | 72 | P | TON_1387 | TON_1387 | TON_1387 | 1261693 | 1262244 | 8 |

| | | | | | | | | | | |
|---|---|---|---|---|---|---|---|---|---|---|
| TU0779 | 1262276 | + | 91 | P | TON_1388; TON_1389 | TON_1388 | TON_1389 | 1262287 | 1263703 | 11 |
| TU0780 | 1264238 | - | -14 | I | TON_1390 | TON_1390 | TON_1390 | 1264640 | 1263693 | - |
| TU0781 | 1264650 | - | -16 | P | TON_1390 | TON_1390 | TON_1390 | 1264640 | 1263693 | 10 |
| TU0782 | 1267819 | + | 18 | P | TON_1394 | TON_1394 | TON_1394 | 1267819 | 1268985 | 0 |
| TU0783 | 1269706 | + | 411 | P | TON_1396 | TON_1396 | TON_1396 | 1269706 | 1270392 | 0 |
| TU0784 | 1271989 | - | -29 | P | TON_1397 | TON_1397 | TON_1397 | 1271978 | 1270398 | 11 |
| TU0785 | 1273045 | + | 2914 | P | TON_1399 | TON_1399 | TON_1399 | 1273057 | 1273665 | 12 |
| TU0786 | 1274282 | - | -51 | P | TON_1400 | TON_1400 | TON_1400 | 1274228 | 1273662 | 54 |
| TU0787 | 1274705 | - | -62 | P | TON_1401 | TON_1401 | TON_1401 | 1274461 | 1274306 | 244 |
| TU0788 | 1277882 | - | -11 | P | TON_1980 | TON_1980 | TON_1980 | 1277847 | 1274841 | - |
| TU0789 | 1279595 | - | -70 | P | TON_1979 | TON_1979 | TON_1979 | 1279595 | 1278100 | - |
| TU0790 | 1279837 | - | -8 | N | TON_nc058 | TON_nc058 | TON_nc058 | - | - | - |
| TU0791 | 1282518 | - | -16 | P | TON_1405; TON_1404 | TON_1405 | TON_1404 | 1282516 | 1281439 | 2 |
| TU0792 | 1282603 | + | 5 | P | TON_1406; TON_1407 | TON_1406 | TON_1407 | 1282616 | 1283937 | 13 |
| TU0793 | 1286149 | - | -52 | P | TON_1409; TON_1408 | TON_1409 | TON_1408 | 1286136 | 1284009 | 13 |
| TU0794 | 1286254 | + | 343 | P | TON_1410 | TON_1410 | TON_1410 | 1286264 | 1287190 | 10 |
| TU0795 | 1287219 | + | 8 | P | TON_1411; TON_1412 | TON_1411 | TON_1412 | 1287229 | 1289125 | 10 |
| TU0796 | 1291628 | - | -8 | P | TON_1414; TON_1413 | TON_1414 | TON_1413 | 1291612 | 1289130 | 16 |
| TU0797 | 1291696 | - | -3 | S | TON_1414; TON_1413 | TON_1414 | TON_1413 | 1291612 | 1289130 | - |
| TU0798 | 1291749 | + | 349 | P | TON_1415 | TON_1415 | TON_1415 | 1291759 | 1292946 | 10 |
| TU0799 | 1294380 | - | -16 | P | TON_1418; TON_1417; TON_1416 | TON_1418 | TON_1416 | 1294380 | 1292970 | 0 |
| TU0800 | 1296090 | + | 548 | N | TON_nc059 | TON_nc059 | TON_nc059 | 1296243 | 1303361 | - |
| TU0801 | 1303402 | + | 7 | P | TON_1424; TON_1425 | TON_1424 | TON_1425 | 1303402 | 1305140 | 0 |
| TU0802 | 1305165 | + | 434 | P | TON_1426 | TON_1426 | TON_1426 | 1305176 | 1305778 | 11 |
| TU0803 | 1308366 | + | 3 | P | TON_1430 | TON_1430 | TON_1430 | 1308426 | 1309214 | 60 |
| TU0804 | 1309737 | - | -58 | P | TON_1431 | TON_1431 | TON_1431 | 1309729 | 1309274 | 8 |
| TU0805 | 1309908 | + | 44 | P | TON_1432 | TON_1432 | TON_1432 | 1309938 | 1311284 | 30 |
| TU0806 | 1311364 | + | 40 | P | TON_1433 | TON_1433 | TON_1433 | 1311374 | 1311616 | 10 |
| TU0807 | 1311680 | + | 121 | N | TON_nc060 | TON_nc060 | TON_nc060 | - | - | - |
| TU0808 | 1312269 | + | 7 | A | TON_nc061 | TON_nc061 | TON_nc061 | - | - | - |
| TU0809 | 1313500 | + | 2 | P | TON_1437; TON_1438 | TON_1437 | TON_1438 | 1313508 | 1314898 | 8 |
| TU0810 | 1314937 | + | 108 | P | TON_1439 | TON_1439 | TON_1439 | 1314946 | 1316508 | 9 |

| | | | | | | | | | | |
|---|---|---|---|---|---|---|---|---|---|---|
| TU0811 | 1317088 | - | -19 | P | TON_1440 | TON_1440 | TON_1440 | 1317078 | 1316485 | 10 |
| TU0812 | 1317196 | + | 28 | P | TON_1441; TON_1442 | TON_1441 | TON_1442 | 1317212 | 1318581 | 16 |
| TU0813 | 1318605 | + | 135 | P | TON_1443 | TON_1443 | TON_1443 | 1318605 | 1320347 | 0 |
| TU0814 | 1322847 | - | -125 | P | TON_1445 | TON_1445 | TON_1445 | 1322825 | 1321476 | 22 |
| TU0815 | 1325598 | - | -876 | P | TON_1448 | TON_1448 | TON_1448 | 1325407 | 1325015 | 191 |
| TU0816 | 1326455 | - | -17 | P | TON_1449 | TON_1449 | TON_1449 | 1326444 | 1325659 | 11 |
| TU0817 | 1326667 | + | 9 | P | TON_1450 | TON_1450 | TON_1450 | 1326680 | 1327255 | 13 |
| TU0818 | 1331621 | - | -1 | P | TON_1453 | TON_1453 | TON_1453 | 1331508 | 1330213 | 113 |
| TU0819 | 1332875 | - | -5 | P | TON_1454 | TON_1454 | TON_1454 | 1332866 | 1331505 | 9 |
| TU0820 | 1332913 | + | 33 | P | TON_1455; TON_1456 | TON_1455 | TON_1456 | 1332949 | 1333709 | 36 |
| TU0821 | 1336001 | - | -19 | P | TON_1459 | TON_1459 | TON_1459 | 1335991 | 1335446 | 10 |
| TU0822 | 1336097 | + | 18 | P | TON_1460 | TON_1460 | TON_1460 | 1336109 | 1338025 | 12 |
| TU0823 | 1339297 | - | -12 | P | TON_1461 | TON_1461 | TON_1461 | 1339297 | 1338020 | 0 |
| TU0824 | 1339334 | + | 10609 | N | TON_nc062 | TON_nc062 | TON_nc062 | - | - | - |
| TU0825 | 1340504 | - | -9 | P | TON_1462 | TON_1462 | TON_1462 | 1340494 | 1339388 | 10 |
| TU0826 | 1342027 | - | -28 | P | TON_1463 | TON_1463 | TON_1463 | 1341945 | 1341022 | 82 |
| TU0827 | 1342601 | - | -150 | P | TON_1464 | TON_1464 | TON_1464 | 1342589 | 1341942 | 12 |
| TU0828 | 1343568 | + | 82 | P | TON_1466; TON_1467 | TON_1466 | TON_1467 | 1343569 | 1344814 | 1 |
| TU0829 | 1344617 | + | 4 | P | TON_1468 | TON_1468 | TON_1468 | 1344790 | 1345827 | 173 |
| TU0830 | 1348387 | + | 19 | P | TON_1472 | TON_1472 | TON_1472 | 1348397 | 1348732 | 10 |
| TU0831 | 1349142 | - | -1722 | P | TON_1473 | TON_1473 | TON_1473 | 1349132 | 1348773 | 10 |
| TU0832 | 1349241 | + | 49 | P | TON_1474 | TON_1474 | TON_1474 | 1349239 | 1350528 | -2 |
| TU0833 | 1351494 | - | -16 | P | TON_1475 | TON_1475 | TON_1475 | 1351470 | 1350508 | 24 |
| TU0834 | 1351676 | + | 423 | P | TON_1476 | TON_1476 | TON_1476 | 1351697 | 1352257 | 21 |
| TU0835 | 1354778 | + | 6 | P | TON_1480; TON_1481; TON_1482 | TON_1480 | TON_1482 | 1354815 | 1357334 | 37 |
| TU0836 | 1357451 | + | 70 | P | TON_1483 | TON_1483 | TON_1483 | 1357465 | 1358382 | 14 |
| TU0837 | 1361233 | - | -22220 | P | TON_1486 | TON_1486 | TON_1486 | 1361173 | 1360637 | 60 |
| TU0838 | 1361636 | - | -463 | P | TON_1487 | TON_1487 | TON_1487 | 1361600 | 1361316 | 36 |
| TU0839 | 1361686 | + | 242 | N | TON_nc063 | TON_nc063 | TON_nc063 | - | - | - |
| TU0840 | 1362625 | - | -20 | P | TON_1488 | TON_1488 | TON_1488 | 1362605 | 1361712 | 20 |
| TU0841 | 1362732 | + | 89 | P | TON_1489 | TON_1489 | TON_1489 | 1362747 | 1363340 | 15 |
| TU0842 | 1363615 | - | -34 | P | TON_1490 | TON_1490 | TON_1490 | 1363605 | 1363330 | 10 |

| | | | | | | | | | | |
|---|---|---|---|---|---|---|---|---|---|---|
| TU0843 | 1363666 | + | 9 | P | TON_1491; TON_1492 | TON_1491 | TON_1492 | 1363679 | 1364574 | 13 |
| TU0844 | 1364605 | + | 90 | N | TON_nc064 | TON_nc064 | TON_nc064 | - | - | - |
| TU0845 | 1367702 | + | 762 | P | TON_2004 | TON_2004 | TON_2004 | 1367760 | 1367846 | - |
| TU0846 | 1367953 | + | 491 | P | TON_1496 | TON_1496 | TON_1496 | 1368053 | 1368289 | 100 |
| TU0847 | 1368042 | + | 133 | S | TON_1496 | TON_1496 | TON_1496 | 1368053 | 1368289 | - |
| TU0848 | 1368385 | + | 5 | P | TON_1497 | TON_1497 | TON_1497 | 1368397 | 1369524 | 12 |
| TU0849 | 1369608 | + | 19 | P | TON_1498 | TON_1498 | TON_1498 | 1369681 | 1371642 | 73 |
| TU0850 | 1374525 | + | 8 | P | TON_1501 | TON_1501 | TON_1501 | 1374537 | 1375799 | 12 |
| TU0851 | 1376906 | + | 58 | P | TON_1503 | TON_1503 | TON_1503 | 1376915 | 1377757 | 9 |
| TU0852 | 1377851 | + | 35 | P | TON_1504; TON_1505 | TON_1504 | TON_1505 | 1377906 | 1380524 | 55 |
| TU0853 | 1384451 | - | -6 | P | TON_1508 | TON_1508 | TON_1508 | 1384434 | 1383799 | 17 |
| TU0854 | 1384535 | + | 262 | P | TON_1509 | TON_1509 | TON_1509 | 1384238 | 1385170 | -297 |
| TU0855 | 1387623 | - | -1523 | P | TON_1511 | TON_1511 | TON_1511 | 1387623 | 1386364 | 0 |
| TU0856 | 1387721 | + | 262 | P | TON_1512 | TON_1512 | TON_1512 | 1387731 | 1387946 | 10 |
| TU0857 | 1388024 | + | 27 | N | TON_nc065 | TON_nc065 | TON_nc065 | - | - | - |
| TU0858 | 1389443 | + | 7 | P | TON_1514 | TON_1514 | TON_1514 | 1389443 | 1390150 | 0 |
| TU0859 | 1391838 | - | -6 | P | TON_1515 | TON_1515 | TON_1515 | 1391828 | 1390140 | 10 |
| TU0860 | 1392773 | - | -15 | P | TON_1516 | TON_1516 | TON_1516 | 1392771 | 1391869 | 2 |
| TU0861 | 1394526 | - | -10 | P | TON_1517 | TON_1517 | TON_1517 | 1394503 | 1392845 | 23 |
| TU0862 | 1395779 | - | -17 | P | TON_1519; TON_1518 | TON_1519 | TON_1518 | 1395770 | 1394814 | 9 |
| TU0863 | 1395903 | + | 196 | P | TON_1520 | TON_1520 | TON_1520 | 1395932 | 1397704 | 29 |
| TU0864 | 1399492 | - | -41 | P | TON_1522 | TON_1522 | TON_1522 | 1399483 | 1398035 | 9 |
| TU0865 | 1399564 | + | 1325 | P | TON_1523 | TON_1523 | TON_1523 | 1399564 | 1399917 | 0 |
| TU0866 | 1401024 | - | -5 | P | TON_1525 | TON_1525 | TON_1525 | 1400993 | 1400553 | 31 |
| TU0867 | 1401221 | - | -219 | P | TON_2014 | TON_2014 | TON_2014 | 1401205 | 1401128 | - |
| TU0868 | 1401274 | + | 415 | P | TON_1526; TON_1527 | TON_1526 | TON_1527 | 1401283 | 1402418 | 9 |
| TU0869 | 1402777 | - | -39 | P | TON_1528 | TON_1528 | TON_1528 | 1402734 | 1402483 | 43 |
| TU0870 | 1403382 | - | -22 | P | TON_1529 | TON_1529 | TON_1529 | 1403369 | 1402842 | 13 |
| TU0871 | 1403465 | + | 16 | P | TON_1530 | TON_1530 | TON_1530 | 1403465 | 1404352 | 0 |
| TU0872 | 1405095 | - | -120 | P | TON_2013 | TON_2013 | TON_2013 | 1405094 | 1405018 | - |
| TU0873 | 1405154 | + | 8 | P | TON_1531 | TON_1531 | TON_1531 | 1405237 | 1405884 | 83 |
| TU0874 | 1406845 | - | -18 | P | TON_1532 | TON_1532 | TON_1532 | 1406688 | 1405939 | 157 |

| | | | | | | | | | | |
|---|---|---|---|---|---|---|---|---|---|---|
| TU0875 | 1408596 | - | -21 | P | TON_1534; TON_1533 | TON_1534 | TON_1533 | 1408539 | 1407539 | 57 |
| TU0876 | 1408601 | + | 1 | P | TON_1535; TON_1536 | TON_1535 | TON_1536 | 1408604 | 1410628 | 3 |
| TU0877 | 1410659 | + | 523 | P | TON_1537; TON_1538; TON_1539 | TON_1537 | TON_1539 | 1410670 | 1412191 | 11 |
| TU0878 | 1420999 | - | -23 | P | TON_1545 | TON_1545 | TON_1545 | 1420992 | 1420345 | 7 |
| TU0879 | 1421035 | + | 506 | P | TON_1546 | TON_1546 | TON_1546 | 1421045 | 1421272 | 10 |
| TU0880 | 1421328 | + | 9 | A | TON_nc066 | TON_nc066 | TON_nc066 | - | - | - |
| TU0881 | 1425467 | - | -651 | P | TON_1552 | TON_1552 | TON_1552 | 1425466 | 1424246 | 1 |
| TU0882 | 1427211 | - | -166 | P | TON_1553 | TON_1553 | TON_1553 | 1427200 | 1425563 | 11 |
| TU0883 | 1427300 | + | 373 | P | TON_1554 | TON_1554 | TON_1554 | 1427309 | 1427665 | 9 |
| TU0884 | 1428680 | - | -25 | P | TON_1556 | TON_1556 | TON_1556 | 1428649 | 1428248 | 31 |
| TU0885 | 1430101 | - | -32 | P | TON_1557 | TON_1557 | TON_1557 | 1430100 | 1428823 | 1 |
| TU0886 | 1430266 | + | 65 | A | TON_nc067 | TON_nc067 | TON_nc067 | - | - | - |
| TU0887 | 1430483 | - | -246 | P | TON_1558 | TON_1558 | TON_1558 | 1430426 | 1430214 | 57 |
| TU0888 | 1430670 | + | 2984 | P | TON_1559; TON_1560; TON_1561 | TON_1559 | TON_1561 | 1430702 | 1433387 | 32 |
| TU0889 | 1434465 | + | 1 | P | TON_1563; TON_1564; TON_1565; TON_1566; TON_1567; TON_1568; TON_1569; TON_1570; TON_1571; TON_1572; TON_1573; TON_1574; TON_1575; TON_1576; TON_1577; | TON_1563 | TON_1580 | 1434543 | 1451361 | 78 |
| TU0890 | 1451396 | + | 6 | P | TON_1581 | TON_1581 | TON_1581 | 1451413 | 1452000 | 17 |
| TU0891 | 1452078 | + | 201 | P | TON_1582; TON_1583; TON_1584; TON_1585; TON_1586; TON_1587; TON_1588; TON_1589; TON_1590; TON_1591; TON_1592; TON_1593; | TON_1582 | TON_1595 | 1452138 | 1460402 | 60 |
| TU0892 | 1461174 | - | -228 | P | TON_1596 | TON_1596 | TON_1596 | 1461164 | 1460676 | 10 |
| TU0893 | 1462653 | + | 4 | P | TON_1600; TON_1601; TON_1602 | TON_1600 | TON_1602 | 1462664 | 1464916 | 11 |
| TU0894 | 1462655 | - | -836 | P | TON_1599 | TON_1599 | TON_1599 | 1462602 | 1462117 | 53 |
| TU0895 | 1465245 | + | 87 | P | TON_1603 | TON_1603 | TON_1603 | 1465349 | 1466341 | 104 |
| TU0896 | 1466441 | + | 4 | A | TON_nc068 | TON_nc068 | TON_nc068 | - | - | - |
| TU0897 | 1469802 | - | -13 | P | TON_1608; TON_1607 | TON_1608 | TON_1607 | 1469795 | 1468841 | 7 |
| TU0898 | 1470267 | - | -3888 | P | TON_1609 | TON_1609 | TON_1609 | 1470267 | 1469962 | 0 |
| TU0899 | 1471526 | - | -97 | P | TON_1610 | TON_1610 | TON_1610 | 1471516 | 1470299 | 10 |
| TU0900 | 1473876 | - | -24 | P | TON_1612 | TON_1612 | TON_1612 | 1473863 | 1472958 | 13 |
| TU0901 | 1474027 | + | 3103 | P | TON_1613 | TON_1613 | TON_1613 | 1474039 | 1475331 | 12 |

| | | | | | | | | | | |
|---|---|---|---|---|---|---|---|---|---|---|
| TU0902 | 1475398 | + | 5 | P | TON_1614 | TON_1614 | TON_1614 | 1475513 | 1476343 | 115 |
| TU0903 | 1476909 | - | -698 | P | TON_1615 | TON_1615 | TON_1615 | 1476875 | 1476423 | 34 |
| TU0904 | 1478026 | - | -117 | P | TON_1617 | TON_1617 | TON_1617 | 1478016 | 1477528 | 10 |
| TU0905 | 1478064 | + | 1346 | P | TON_1618 | TON_1618 | TON_1618 | 1478074 | 1478991 | 10 |
| TU0906 | 1480546 | + | 85 | P | TON_1621 | TON_1621 | TON_1621 | 1480556 | 1481428 | 10 |
| TU0907 | 1482399 | - | -7 | P | TON_1623; TON_1622 | TON_1623 | TON_1622 | 1482387 | 1481421 | 12 |
| TU0908 | 1482982 | - | -188 | P | TON_1624 | TON_1624 | TON_1624 | 1482971 | 1482459 | 11 |
| TU0909 | 1483572 | - | -106 | P | TON_1625 | TON_1625 | TON_1625 | 1483533 | 1483012 | 39 |
| TU0910 | 1483609 | + | 102 | P | TON_1626; TON_1627 | TON_1626 | TON_1627 | 1483639 | 1488266 | 30 |
| TU0911 | 1491997 | + | 41 | P | TON_1632 | TON_1632 | TON_1632 | 1491997 | 1492833 | 0 |
| TU0912 | 1493357 | + | 36 | A | TON_nc069 | TON_nc069 | TON_nc069 | - | - | - |
| TU0913 | 1494576 | - | -5 | P | TON_1634; TON_1633 | TON_1634 | TON_1633 | 1494576 | 1492817 | 0 |
| TU0914 | 1497785 | + | 570 | P | TON_1638 | TON_1638 | TON_1638 | 1497785 | 1498267 | 0 |
| TU0915 | 1498576 | + | 4 | P | TON_1640 | TON_1640 | TON_1640 | 1498586 | 1499266 | 10 |
| TU0916 | 1510259 | - | -51 | P | TON_1645 | TON_1645 | TON_1645 | 1510249 | 1509623 | 10 |
| TU0917 | 1510583 | - | -1469 | N | TON_nc070 | TON_nc070 | TON_nc070 | - | - | - |
| TU0918 | 1512094 | + | 572 | P | TON_1647 | TON_1647 | TON_1647 | 1512120 | 1513181 | 26 |
| TU0919 | 1516112 | + | 12 | P | TON_1652 | TON_1652 | TON_1652 | 1516122 | 1516580 | 10 |
| TU0920 | 1516634 | + | 7 | P | TON_1653; TON_1654; TON_1655 | TON_1653 | TON_1655 | 1516653 | 1519451 | 19 |
| TU0921 | 1523057 | + | 31 | P | TON_1659 | TON_1659 | TON_1659 | 1523066 | 1524508 | 9 |
| TU0922 | 1524067 | + | 27 | I | TON_1659 | TON_1659 | TON_1659 | 1523066 | 1524508 | - |
| TU0923 | 1527617 | - | -13 | P | TON_1663 | TON_1663 | TON_1663 | 1527536 | 1526961 | 81 |
| TU0924 | 1527642 | + | 202 | P | TON_1664 | TON_1664 | TON_1664 | 1527681 | 1528997 | 39 |
| TU0925 | 1527871 | - | -10 | A | TON_nc071 | TON_nc071 | TON_nc071 | - | - | - |
| TU0926 | 1528990 | - | -21 | A | TON_nc072 | TON_nc072 | TON_nc072 | - | - | - |
| TU0927 | 1530491 | + | 145 | P | TON_1666; TON_1667 | TON_1666 | TON_1667 | 1530586 | 1532430 | 95 |
| TU0928 | 1533088 | + | 141 | P | TON_1669; TON_1670 | TON_1669 | TON_1670 | 1533149 | 1535384 | 61 |
| TU0929 | 1537746 | - | -221 | P | TON_1672; TON_1671 | TON_1672 | TON_1671 | 1537655 | 1535478 | 91 |
| TU0930 | 1537991 | + | 15 | P | TON_1673; TON_1674 | TON_1673 | TON_1674 | 1538036 | 1540461 | 45 |
| TU0931 | 1540433 | + | 35 | P | TON_1675; TON_1676; TON_1677 | TON_1675 | TON_1677 | 1540468 | 1544317 | 35 |
| TU0932 | 1544806 | - | -1919 | P | TON_1678 | TON_1678 | TON_1678 | 1544782 | 1544291 | 24 |
| TU0933 | 1545743 | - | -30 | P | TON_1680 | TON_1680 | TON_1680 | 1545732 | 1545238 | 11 |

| | | | | | | | | | | |
|---|---|---|---|---|---|---|---|---|---|---|
| TU0934 | 1545795 | + | 372 | P | TON_1681 | TON_1681 | TON_1681 | 1545802 | 1546662 | 7 |
| TU0935 | 1553800 | - | -54 | P | TON_1689 | TON_1689 | TON_1689 | 1553789 | 1552677 | 11 |
| TU0936 | 1553841 | + | 8 | P | TON_1690 | TON_1690 | TON_1690 | 1553851 | 1554453 | 10 |
| TU0937 | 1554487 | + | 396 | P | TON_1691 | TON_1691 | TON_1691 | 1554564 | 1556498 | 77 |
| TU0938 | 1557233 | - | -6 | I | TON_1692 | TON_1692 | TON_1692 | 1557608 | 1556637 | - |
| TU0939 | 1557617 | - | -7 | P | TON_1692 | TON_1692 | TON_1692 | 1557608 | 1556637 | 9 |
| TU0940 | 1557705 | + | 3 | P | TON_1693 | TON_1693 | TON_1693 | 1557715 | 1558443 | 10 |
| TU0941 | 1559590 | - | -249 | P | TON_1694 | TON_1694 | TON_1694 | 1559579 | 1558440 | 11 |
| TU0942 | 1561794 | - | -5 | P | TON_1696 | TON_1696 | TON_1696 | 1561783 | 1560512 | 11 |
| TU0943 | 1561923 | + | 171 | P | TON_1697 | TON_1697 | TON_1697 | 1561937 | 1563028 | 14 |
| TU0944 | 1563091 | + | 118 | P | TON_1698 | TON_1698 | TON_1698 | 1563114 | 1564316 | 23 |
| TU0945 | 1563811 | - | -71 | A | TON_nc073 | TON_nc073 | TON_nc073 | - | - | - |
| TU0946 | 1564214 | + | 82 | P | TON_1699; TON_1700; TON_1701 | TON_1699 | TON_1701 | 1564267 | 1566859 | 53 |
| TU0947 | 1574597 | - | -9 | P | TON_1708; TON_1707; TON_1706 | TON_1708 | TON_1706 | 1574562 | 1572205 | 35 |
| TU0948 | 1574714 | + | 3409 | N | TON_nc074 | TON_nc074 | TON_nc074 | - | - | - |
| TU0949 | 1574802 | + | 11036 | N | TON_nc075 | TON_nc075 | TON_nc075 | 1574986 | 1575849 | - |
| TU0950 | 1574978 | + | 34 | P | TON_1709 | TON_1709 | TON_1709 | 1574986 | 1575849 | 8 |
| TU0951 | 1577004 | - | -230 | P | TON_1710 | TON_1710 | TON_1710 | 1576990 | 1575839 | 14 |
| TU0952 | 1578455 | - | -156 | P | TON_1712; TON_1711 | TON_1712 | TON_1711 | 1578453 | 1577028 | 2 |
| TU0953 | 1578527 | + | 109 | P | TON_1713; TON_1714 | TON_1713 | TON_1714 | 1578527 | 1579826 | 0 |
| TU0954 | 1583313 | - | -47 | P | TON_1715 | TON_1715 | TON_1715 | 1583280 | 1579867 | 33 |
| TU0955 | 1587841 | - | -487 | P | TON_1718; TON_1717 | TON_1718 | TON_1717 | 1587832 | 1585419 | 9 |
| TU0956 | 1587980 | + | 36 | P | TON_1719; TON_1720 | TON_1719 | TON_1720 | 1587989 | 1589696 | 9 |
| TU0957 | 1589836 | + | 4 | P | TON_1721 | TON_1721 | TON_1721 | 1589836 | 1590453 | 0 |
| TU0958 | 1590504 | + | 96 | P | TON_1722 | TON_1722 | TON_1722 | 1590516 | 1591829 | 12 |
| TU0959 | 1591133 | + | 157 | I | TON_1722 | TON_1722 | TON_1722 | 1590516 | 1591829 | - |
| TU0960 | 1591962 | - | -29 | P | TON_1723 | TON_1723 | TON_1723 | 1591953 | 1591834 | 9 |
| TU0961 | 1592762 | - | -16 | P | TON_1724 | TON_1724 | TON_1724 | 1592757 | 1591993 | 5 |
| TU0962 | 1593004 | + | 23 | P | TON_1725 | TON_1725 | TON_1725 | 1593026 | 1593280 | 22 |
| TU0963 | 1594997 | + | 67 | P | TON_1727 | TON_1727 | TON_1727 | 1595089 | 1595256 | 92 |
| TU0964 | 1595224 | + | 797 | P | TON_1728 | TON_1728 | TON_1728 | 1595281 | 1595577 | 57 |
| TU0965 | 1595618 | + | 10 | P | TON_1729; TON_1730 | TON_1729 | TON_1730 | 1595636 | 1598772 | 18 |

| | | | | | | | | | | |
|---|---|---|---|---|---|---|---|---|---|---|
| TU0966 | 1599214 | - | -61 | P | TON_1731 | TON_1731 | TON_1731 | 1599203 | 1598736 | 11 |
| TU0967 | 1600050 | - | -35 | P | TON_1732 | TON_1732 | TON_1732 | 1600040 | 1599285 | 10 |
| TU0968 | 1600160 | + | 5 | P | TON_1733 | TON_1733 | TON_1733 | 1600173 | 1601951 | 13 |
| TU0969 | 1602488 | - | -5 | A | TON_nc076 | TON_nc076 | TON_nc076 | - | - | - |
| TU0970 | 1603384 | + | 12 | S | TON_1736 | TON_1736 | TON_1736 | 1603384 | 1603905 | 0 |
| TU0971 | 1603408 | + | 12 | P | TON_1736 | TON_1736 | TON_1736 | 1603384 | 1603905 | - |
| TU0972 | 1603937 | + | 2802 | P | TON_1737 | TON_1737 | TON_1737 | 1603938 | 1604447 | 1 |
| TU0973 | 1608133 | - | -143 | P | TON_1742 | TON_1742 | TON_1742 | 1608121 | 1607696 | 12 |
| TU0974 | 1608396 | + | 25 | P | TON_1743; TON_1744; TON_1745 | TON_1743 | TON_1745 | 1608429 | 1611558 | 33 |
| TU0975 | 1611645 | + | 1294 | P | TON_1746; TON_1747; TON_1748; TON_1749; TON_1750; TON_1751; TON_1752; TON_1753; TON_1754 | TON_1746 | TON_1754 | 1611688 | 1620370 | 43 |
| TU0976 | 1624225 | - | -644 | P | TON_1758 | TON_1758 | TON_1758 | 1624225 | 1623395 | 0 |
| TU0977 | 1628166 | + | 247 | P | TON_1764 | TON_1764 | TON_1764 | 1628221 | 1630653 | 55 |
| TU0978 | 1630814 | + | 164 | P | TON_1765; TON_1766; TON_1767; TON_1768; TON_1769 | TON_1765 | TON_1769 | 1630854 | 1635867 | 40 |
| TU0979 | 1635776 | + | 17 | P | TON_1770; TON_1771 | TON_1770 | TON_1771 | 1635864 | 1637504 | 88 |
| TU0980 | 1638873 | - | -1144 | P | TON_1772 | TON_1772 | TON_1772 | 1638832 | 1637501 | 41 |
| TU0981 | 1639052 | + | 20 | P | TON_1773 | TON_1773 | TON_1773 | 1639052 | 1639459 | 0 |
| TU0982 | 1639440 | + | 12 | P | TON_1774 | TON_1774 | TON_1774 | 1639465 | 1639740 | 25 |
| TU0983 | 1639771 | + | 3 | P | TON_1775 | TON_1775 | TON_1775 | 1639782 | 1641389 | 11 |
| TU0984 | 1641435 | + | 201 | N | TON_nc077 | TON_nc077 | TON_nc077 | - | - | - |
| TU0985 | 1644842 | - | -4 | P | TON_1778; TON_1777; TON_1776 | TON_1778 | TON_1776 | 1644829 | 1641743 | 13 |
| TU0986 | 1645093 | + | 17 | P | TON_1779 | TON_1779 | TON_1779 | 1645143 | 1645892 | 50 |
| TU0987 | 1645974 | + | 4 | P | TON_1780 | TON_1780 | TON_1780 | 1646085 | 1647140 | 111 |
| TU0988 | 1648578 | - | -308 | P | TON_1782 | TON_1782 | TON_1782 | 1648569 | 1648261 | 9 |
| TU0989 | 1648630 | + | 26 | P | TON_1783 | TON_1783 | TON_1783 | 1648641 | 1649831 | 11 |
| TU0990 | 1649856 | + | 45 | N | TON_nc078 | TON_nc078 | TON_nc078 | - | - | - |
| TU0991 | 1650668 | - | -89 | P | TON_1784 | TON_1784 | TON_1784 | 1650668 | 1649925 | 0 |
| TU0992 | 1656799 | - | -69 | P | TON_1789 | TON_1789 | TON_1789 | 1656790 | 1655423 | 9 |
| TU0993 | 1657224 | - | -18 | P | TON_1790 | TON_1790 | TON_1790 | 1657224 | 1656820 | 0 |
| TU0994 | 1665991 | - | -2677 | P | TON_1795 | TON_1795 | TON_1795 | 1665963 | 1664644 | 28 |
| TU0995 | 1668068 | + | 334 | P | TON_1797; TON_1798 | TON_1797 | TON_1798 | 1668118 | 1670532 | 50 |

| | | | | | | | | | | |
|---|---|---|---|---|---|---|---|---|---|---|
| TU0996 | 1668073 | - | -68 | P | TON_1796 | TON_1796 | TON_1796 | 1668047 | 1666089 | 26 |
| TU0997 | 1670958 | + | 65 | P | TON_1800 | TON_1800 | TON_1800 | 1670980 | 1672062 | 22 |
| TU0998 | 1672182 | + | 8 | P | TON_1801 | TON_1801 | TON_1801 | 1672182 | 1672958 | 0 |
| TU0999 | 1672996 | + | 876 | P | TON_1802 | TON_1802 | TON_1802 | 1672996 | 1673178 | 0 |
| TU1000 | 1676516 | - | -93 | P | TON_1803 | TON_1803 | TON_1803 | 1676398 | 1673201 | 118 |
| TU1001 | 1677214 | - | -10 | P | TON_1804 | TON_1804 | TON_1804 | 1677202 | 1676828 | 12 |
| TU1002 | 1678198 | + | 11 | P | TON_1806 | TON_1806 | TON_1806 | 1678209 | 1678736 | 11 |
| TU1003 | 1680921 | + | 208 | P | TON_1809 | TON_1809 | TON_1809 | 1680932 | 1681363 | 11 |
| TU1004 | 1681681 | - | -633 | P | TON_1810 | TON_1810 | TON_1810 | 1681672 | 1681364 | 9 |
| TU1005 | 1685703 | - | -30 | P | TON_1813; TON_1812 | TON_1813 | TON_1812 | 1685693 | 1683769 | 10 |
| TU1006 | 1688811 | - | -22 | P | TON_1814 | TON_1814 | TON_1814 | 1688800 | 1685834 | 11 |
| TU1007 | 1689415 | - | -1809 | P | TON_1815 | TON_1815 | TON_1815 | 1689356 | 1688892 | 59 |
| TU1008 | 1692280 | - | -7 | P | TON_1816 | TON_1816 | TON_1816 | 1692226 | 1689494 | 54 |
| TU1009 | 1693666 | - | -48 | P | TON_1818; TON_1817 | TON_1818 | TON_1817 | 1693653 | 1692416 | 13 |
| TU1010 | 1693742 | + | 106 | P | TON_1819 | TON_1819 | TON_1819 | 1693755 | 1696085 | 13 |
| TU1011 | 1697190 | + | 28 | A | TON_nc079 | TON_nc079 | TON_nc079 | - | - | - |
| TU1012 | 1698449 | - | -45 | P | TON_1820 | TON_1820 | TON_1820 | 1698436 | 1696082 | 13 |
| TU1013 | 1698583 | + | 11 | P | TON_1821 | TON_1821 | TON_1821 | 1698600 | 1699724 | 17 |
| TU1014 | 1699628 | + | 4 | P | TON_1822; TON_1823 | TON_1822 | TON_1823 | 1699714 | 1701923 | 86 |
| TU1015 | 1702890 | - | -33 | N | TON_nc080 | TON_nc080 | TON_nc080 | - | - | - |
| TU1016 | 1709196 | + | 3 | P | TON_1825 | TON_1825 | TON_1825 | 1709207 | 1709929 | 11 |
| TU1017 | 1709927 | - | -277 | A | TON_nc081 | TON_nc081 | TON_nc081 | - | - | - |
| TU1018 | 1719562 | - | -13 | P | TON_1833 | TON_1833 | TON_1833 | 1719555 | 1717813 | 7 |
| TU1019 | 1721528 | - | -23 | P | TON_1836 | TON_1836 | TON_1836 | 1721518 | 1720565 | 10 |
| TU1020 | 1721577 | + | 120 | P | TON_1837 | TON_1837 | TON_1837 | 1721579 | 1722601 | 2 |
| TU1021 | 1724083 | - | -8 | N | TON_nc082 | TON_nc082 | TON_nc082 | - | - | - |
| TU1022 | 1724170 | + | 3 | N | TON_nc083 | TON_nc083 | TON_nc083 | - | - | - |
| TU1023 | 1724396 | - | -19 | N | TON_nc084 | TON_nc084 | TON_nc084 | - | - | - |
| TU1024 | 1724623 | + | 17 | P | TON_1841 | TON_1841 | TON_1841 | 1724638 | 1725282 | 15 |
| TU1025 | 1728171 | + | 11 | P | TON_1846; TON_1847; TON_1848 | TON_1846 | TON_1848 | 1728181 | 1729344 | 10 |
| TU1026 | 1729394 | + | 251 | P | TON_1849; TON_1850; TON_1851 | TON_1849 | TON_1851 | 1729408 | 1730917 | 14 |
| TU1027 | 1730936 | + | 10 | P | TON_1852 | TON_1852 | TON_1852 | 1730948 | 1732372 | 12 |

| | | | | | | | | | | |
|---|---|---|---|---|---|---|---|---|---|---|
| TU1028 | 1732632 | + | 196 | P | TON_1853; TON_1975 | TON_1853 | TON_1975 | 1732764 | 1733761 | 132 |
| TU1029 | 1736587 | - | -18 | I | TON_1855; TON_1854 | TON_1855 | TON_1854 | 1737121 | 1734419 | - |
| TU1030 | 1737280 | - | -25 | P | TON_1855; TON_1854 | TON_1855 | TON_1854 | 1737121 | 1734419 | 159 |
| TU1031 | 1737498 | + | 45 | N | TON_nc085 | TON_nc085 | TON_nc085 | - | - | - |
| TU1032 | 1743596 | - | -4 | P | TON_1862; TON_1861; TON_1860; TON_1859; TON_1858; TON_1857; | TON_1862 | TON_1856 | 1743512 | 1737726 | 84 |
| TU1033 | 1744744 | + | 80 | P | TON_1863; TON_1864 | TON_1863 | TON_1864 | 1744746 | 1746321 | 2 |
| TU1034 | 1746375 | + | 143 | P | TON_1865; TON_1866 | TON_1865 | TON_1866 | 1746411 | 1746885 | 36 |
| TU1035 | 1749772 | - | -599 | P | TON_1869 | TON_1869 | TON_1869 | 1749760 | 1748735 | 12 |
| TU1036 | 1750038 | + | 213 | P | TON_1870; TON_1871 | TON_1870 | TON_1871 | 1750066 | 1752652 | 28 |
| TU1037 | 1763353 | + | 15 | P | TON_1878 | TON_1878 | TON_1878 | 1763378 | 1764445 | 25 |
| TU1038 | 1767248 | - | -14 | P | TON_1881 | TON_1881 | TON_1881 | 1767173 | 1766586 | 75 |
| TU1039 | 1769172 | - | -43 | P | TON_1882 | TON_1882 | TON_1882 | 1769162 | 1767180 | 10 |
| TU1040 | 1769643 | - | -78 | P | TON_1883 | TON_1883 | TON_1883 | 1769585 | 1769295 | 58 |
| TU1041 | 1769993 | + | 13 | P | TON_1884 | TON_1884 | TON_1884 | 1770019 | 1771479 | 26 |
| TU1042 | 1771548 | + | 21 | P | TON_1885 | TON_1885 | TON_1885 | 1771558 | 1772958 | 10 |
| TU1043 | 1774232 | + | 13 | P | TON_1887 | TON_1887 | TON_1887 | 1774242 | 1775150 | 10 |
| TU1044 | 1775228 | + | 248 | P | TON_1888 | TON_1888 | TON_1888 | 1775228 | 1776538 | 0 |
| TU1045 | 1779579 | - | -27 | P | TON_1892 | TON_1892 | TON_1892 | 1779569 | 1779399 | 10 |
| TU1046 | 1781374 | - | -158 | N | TON_nc086 | TON_nc086 | TON_nc086 | - | - | - |
| TU1047 | 1791063 | - | -104 | P | TON_1901; TON_1900; TON_1899; TON_1898; TON_1897; TON_1896; | TON_1901 | TON_1895 | 1791050 | 1781711 | 13 |
| TU1048 | 1795045 | - | -179 | P | TON_1904; TON_1903; TON_1902 | TON_1904 | TON_1902 | 1795034 | 1791090 | 11 |
| TU1049 | 1796102 | - | -29 | P | TON_1905 | TON_1905 | TON_1905 | 1796091 | 1795105 | 11 |
| TU1050 | 1796939 | - | -60 | P | TON_1906 | TON_1906 | TON_1906 | 1796939 | 1796355 | 0 |
| TU1051 | 1797169 | + | 285 | P | TON_1907 | TON_1907 | TON_1907 | 1797197 | 1798477 | 28 |
| TU1052 | 1803130 | - | -1 | P | TON_1915 | TON_1915 | TON_1915 | 1803034 | 1802582 | 96 |
| TU1053 | 1803350 | - | -92 | N | TON_nc087 | TON_nc087 | TON_nc087 | - | - | - |
| TU1054 | 1803797 | - | -15113 | P | TON_2012 | TON_2012 | TON_2012 | 1803791 | 1803716 | - |
| TU1055 | 1804848 | - | -5 | P | TON_2011 | TON_2011 | TON_2011 | 1804842 | 1804765 | - |
| TU1056 | 1804900 | + | 245 | P | TON_1918 | TON_1918 | TON_1918 | 1804922 | 1805980 | 22 |
| TU1057 | 1808104 | - | -120 | P | TON_1920 | TON_1920 | TON_1920 | 1808070 | 1807729 | 34 |
| TU1058 | 1808973 | - | -745 | P | TON_1922 | TON_1922 | TON_1922 | 1808947 | 1808834 | 26 |

| | | | | | | | | | | |
|---|---|---|---|---|---|---|---|---|---|---|
| TU1059 | 1810340 | + | 56 | P | TON_1924 | TON_1924 | TON_1924 | 1810351 | 1810728 | 11 |
| TU1060 | 1811557 | - | -68 | P | TON_1925 | TON_1925 | TON_1925 | 1811532 | 1810744 | 25 |
| TU1061 | 1811612 | + | 138 | P | TON_1926; TON_1927 | TON_1926 | TON_1927 | 1811628 | 1812705 | 16 |
| TU1062 | 1815652 | - | -112 | P | TON_1933 | TON_1933 | TON_1933 | 1815625 | 1815296 | 27 |
| TU1063 | 1815735 | + | 57 | P | TON_1934; TON_1935 | TON_1934 | TON_1935 | 1815744 | 1817386 | 9 |
| TU1064 | 1818540 | + | 75 | P | TON_1937 | TON_1937 | TON_1937 | 1818543 | 1819340 | 3 |
| TU1065 | 1819387 | + | 32 | P | TON_1938 | TON_1938 | TON_1938 | 1819398 | 1820726 | 11 |
| TU1066 | 1822446 | - | -291 | P | TON_1940; TON_1939 | TON_1940 | TON_1939 | 1822446 | 1820723 | 0 |
| TU1067 | 1824532 | - | -73 | P | TON_1942 | TON_1942 | TON_1942 | 1824521 | 1823751 | 11 |
| TU1068 | 1826309 | - | -487 | P | TON_1944; TON_1943 | TON_1944 | TON_1943 | 1826295 | 1824624 | 14 |
| TU1069 | 1826845 | - | -30 | P | TON_1945 | TON_1945 | TON_1945 | 1826792 | 1826415 | 53 |
| TU1070 | 1826923 | + | 680 | P | TON_1946 | TON_1946 | TON_1946 | 1826937 | 1827107 | 14 |
| TU1071 | 1827850 | + | 28 | P | TON_1948 | TON_1948 | TON_1948 | 1827860 | 1828471 | 10 |
| TU1072 | 1828994 | - | -14 | P | TON_1976 | TON_1976 | TON_1976 | 1828882 | 1828418 | 112 |
| TU1073 | 1829046 | + | 22 | P | TON_1949 | TON_1949 | TON_1949 | 1829046 | 1830113 | 0 |
| TU1074 | 1830147 | + | 144 | P | TON_1950; TON_1951 | TON_1950 | TON_1951 | 1830158 | 1831382 | 11 |
| TU1075 | 1833684 | - | -1067 | P | TON_1954; TON_1953; TON_1952 | TON_1954 | TON_1952 | 1833672 | 1831427 | 12 |
| TU1076 | 1834398 | - | -12 | P | TON_1955 | TON_1955 | TON_1955 | 1834384 | 1833797 | 14 |
| TU1077 | 1835573 | + | 142 | P | TON_1956 | TON_1956 | TON_1956 | 1835746 | 1836180 | 173 |
| TU1078 | 1835735 | + | 147 | S | TON_1956 | TON_1956 | TON_1956 | 1835746 | 1836180 | - |
| TU1079 | 1840391 | + | 15 | P | TON_1960 | TON_1960 | TON_1960 | 1840400 | 1841452 | 9 |
| TU1080 | 1844879 | + | 14 | P | TON_1965 | TON_1965 | TON_1965 | 1844891 | 1845508 | 12 |
| TU1081 | 1846861 | - | -16 | P | TON_1966 | TON_1966 | TON_1966 | 1846861 | 1845509 | 0 |
| TU1082 | 1846912 | + | 10 | P | TON_1967 | TON_1967 | TON_1967 | 1846966 | 1847520 | 54 |

**Supplementary Table 3.** The selected gene information for qRT-PCR

| Gene | Function | RPKM (log) | Primer position | | | Primer sequence (5') | |
|---|---|---|---|---|---|---|---|
| | | | F | W | bp | | |
| TON_1559 | Coenzyme F420 hydrogenase α subunit | 5.375 | 562 | 661 | 100 | F | ACCGATGAGGACATCCAGAC |
| | | | | | | R | TGAACCTGTCCCCGTAGAAC |
| TON_0286 | hydrogenase maturation protein HypF | 5.200 | 1642 | 1742 | 101 | F | TTGGCGAGCTACTACCCACT |
| | | | | | | R | GCCTTTGGACAGCACTTCTC |
| TON_0919 | iron-molybdenum cofactor-binding protein | 5.153 | 63 | 175 | 113 | F | AAGAGCTCCAGCTTTCGTCA |
| | | | | | | R | TAGCAAATGGTCCTGCTCCT |
| TON_0539 | formate dehydrogenase subunit alpha | 4.859 | 1730 | 1831 | 102 | F | GCCAGTACCACACACTCACG |
| | | | | | | R | CCAGCTTCTTTGCATCTTCC |
| TON_1552 | sodium/phosphate symporter | 4.509 | 24 | 140 | 117 | F | TGATGCCGCAAATTCTATGA |
| | | | | | | R | ACGCTCTTGCCGAAGAAGTA |
| TON_0579 | metG methionyl-tRNA synthetase | 4.155 | 1583 | 1700 | 118 | F | GGGAGTTCATCGAGATTCCA |
| | | | | | | R | GGGTTGCCTCTGGCTATGTA |
| TON_0767 | translation initiation factor IF-1 | 3.896 | 150 | 256 | 107 | F | AAAGGTCAGGAGATGCAGGA |
| | | | | | | R | CTCTCTCGTCCGTCTGAACC |
| TON_0650 | hydrolase | 3.559 | 228 | 335 | 108 | F | CAAGCTGATGCCGAAGTACA |
| | | | | | | R | TAAGTGTGCTCGCCAGAGAA |
| TON_0305 | NADH oxidase | 3.186 | 183 | 299 | 117 | F | CTATCCACCGGAGGTTTTCA |
| | | | | | | R | TAGGTGTGTTCCCCGTCTTC |
| TON_0889 | ATPase | 2.965 | 38 | 140 | 103 | F | AGATGCTTGAAGGTGGCCTA |
| | | | | | | R | ACGCCTTCAGTGAGGAATTG |

**Supplementary Table 4.** mRNA expression profiles according to the nutrient in *T. onnurineus NA1*

| Gene | Start | End | Strand | Normalized mRNA expression | | | Fold Change | | Function |
|---|---|---|---|---|---|---|---|---|---|
| | | | | YPS | MMC | MMF | MMC/YPS | MMF/YPS | |
| TON_0001 | 1 | 3927 | + | 7706.56 | 10302.24 | 10380.35 | 1.34 | 1.35 | family B-type DNA-dependent polymerase |
| TON_0002 | 4079 | 4774 | - | 1012.02 | 1387.26 | 1846.28 | 1.37 | 1.82 | hydrolase |
| TON_0003 | 4815 | 5831 | - | 1977.47 | 1845.27 | 3460.85 | 0.93 | 1.75 | hypothetical protein |
| TON_0004 | 5832 | 6473 | - | 1460.67 | 2009.96 | 2741.55 | 1.38 | 1.88 | membrane protein |
| TON_0005 | 6437 | 7906 | + | 10501.82 | 9617.03 | 8496.68 | 0.92 | 0.81 | S-layer protein |
| TON_0006 | 8069 | 9247 | + | 26.12 | 37.27 | 71.86 | 1.43 | 2.75 | major facilitator superfamily permease |
| TON_0007 | 9286 | 11511 | + | 2034.26 | 4034.34 | 3471.85 | 1.98 | 1.71 | RecJ-like exonuclease |
| TON_0008 | 11580 | 11834 | + | 494.08 | 797.01 | 435.54 | 1.61 | 0.88 | hypothetical protein |
| TON_0009 | 12484 | 12699 | + | 85.19 | 238.02 | 156.91 | 2.79 | 1.84 | hypothetical protein |
| TON_0010 | 12754 | 13932 | - | 42.03 | 108.19 | 57.93 | 2.57 | 1.38 | Permease |
| TON_0011 | 13936 | 14226 | - | 47.70 | 104.59 | 49.86 | 2.19 | 1.05 | hypothetical protein |
| TON_0012 | 14275 | 14661 | - | 92.00 | 205.56 | 256.63 | 2.23 | 2.79 | thioesterase |
| TON_0013 | 14754 | 15152 | + | 2772.55 | 2907.95 | 1206.16 | 1.05 | 0.44 | hypothetical protein |
| TON_0014 | 15273 | 16313 | + | 2938.38 | 8586.80 | 5103.29 | 2.92 | 1.74 | tungsten ABC transporter substrate-binding protein |
| TON_0015 | 16353 | 17060 | + | 575.86 | 913.62 | 584.39 | 1.59 | 1.01 | Binding-protein-dependent transport systems inner membrane component |
| TON_0016 | 17057 | 17779 | + | 545.20 | 1608.45 | 805.82 | 2.95 | 1.48 | molybdenum-pterin binding domain-containing protein |
| TON_0017 | 18313 | 19359 | - | 730.33 | 910.01 | 1327.15 | 1.25 | 1.82 | membrane protein |
| TON_0018 | 19415 | 19738 | + | 1586.75 | 2423.49 | 1118.91 | 1.53 | 0.71 | hypothetical protein |
| TON_0019 | 20236 | 20919 | + | 822.34 | 893.18 | 2066.98 | 1.09 | 2.51 | hypothetical protein |
| TON_0020 | 21353 | 23269 | - | 5137.33 | 2035.20 | 1545.65 | 0.40 | 0.30 | indolepyruvate: ferredoxin oxidoreductase subunit alpha |
| TON_0021 | 23342 | 23551 | - | 9.09 | 9.62 | 9.53 | 1.06 | 1.05 | hypothetical protein |
| TON_0022 | 23624 | 23875 | - | 37.48 | 36.06 | 18.33 | 0.96 | 0.49 | membrane protein |
| TON_0023 | 23969 | 24256 | + | 480.45 | 919.63 | 470.00 | 1.91 | 0.98 | hypothetical protein |
| TON_0024 | 24266 | 24583 | + | 407.76 | 223.60 | 266.90 | 0.55 | 0.65 | nucleotide pyrophosphohydrolase |
| TON_0025 | 24573 | 25022 | - | 1669.66 | 1002.58 | 3268.01 | 0.60 | 1.96 | bis(5'-adenosyl)-triphosphatase |
| TON_0026 | 25077 | 25313 | + | 616.75 | 1009.79 | 1089.58 | 1.64 | 1.77 | membrane protein |
| TON_0027 | 25378 | 26160 | + | 15258.65 | 25744.79 | 29688.52 | 1.69 | 1.95 | proteasome subunit alpha |
| TON_0028 | 26172 | 26882 | + | 5953.99 | 11355.31 | 18533.14 | 1.91 | 3.11 | putative RNA-associated protein |

| | | | | | | | | | |
|---|---|---|---|---|---|---|---|---|---|
| TON_0029 | 26879 | 27649 | + | 7150.01 | 18616.16 | 24484.78 | 2.60 | 3.42 | exosome complex RNA-binding protein Rrp4 |
| TON_0030 | 27646 | 28395 | + | 6567.33 | 17739.81 | 16915.64 | 2.70 | 2.58 | exosome complex exonuclease Rrp41 |
| TON_0031 | 28388 | 29206 | + | 5709.79 | 16885.10 | 12132.77 | 2.96 | 2.12 | exosome complex RNA-binding protein Rrp42 |
| TON_0032 | 29247 | 29543 | + | 402.08 | 875.15 | 786.02 | 2.18 | 1.95 | membrane protein |
| TON_0033 | 29596 | 29994 | + | 5233.87 | 3638.84 | 4701.48 | 0.70 | 0.90 | hypothetical protein |
| TON_0034 | 30064 | 30699 | + | 4383.14 | 4410.61 | 4875.25 | 1.01 | 1.11 | hypothetical protein |
| TON_0035 | 30696 | 31220 | - | 1394.79 | 1015.80 | 1722.36 | 0.73 | 1.23 | hypothetical protein |
| TON_0036 | 31271 | 32272 | - | 4308.18 | 4794.09 | 6657.74 | 1.11 | 1.55 | homoserine dehydrogenase |
| TON_0037 | 32309 | 33151 | - | 6978.50 | 10686.92 | 11420.80 | 1.53 | 1.64 | hypothetical protein |
| TON_0038 | 33235 | 34362 | - | 1073.35 | 4239.91 | 1643.90 | 3.95 | 1.53 | methylmalonyl-CoA decarboxylase beta chain |
| TON_0039 | 34369 | 34833 | - | 831.42 | 1293.49 | 1541.25 | 1.56 | 1.85 | putative acetyl-CoA carboxylase biotin carboxyl carrier protein subunit |
| TON_0040 | 34836 | 35222 | - | 2245.52 | 3049.80 | 3325.94 | 1.36 | 1.48 | methylmalonyl-CoA decarboxylase subunit delta |
| TON_0041 | 35233 | 36801 | - | 7289.72 | 4394.98 | 4789.46 | 0.60 | 0.66 | methylmalonyl-CoA decarboxylase subunit alpha |
| TON_0042 | 36951 | 37382 | - | 10517.73 | 8223.76 | 6701.00 | 0.78 | 0.64 | translation initiation factor IF-2 |
| TON_0043 | 37451 | 40924 | - | 6560.52 | 8443.75 | 12059.45 | 1.29 | 1.84 | cell division control protein |
| TON_0044 | 40924 | 41418 | - | 2061.52 | 1029.02 | 2383.00 | 0.50 | 1.16 | hypothetical protein |
| TON_0045 | 41527 | 42039 | + | 7801.97 | 10531.85 | 5992.70 | 1.35 | 0.77 | metallophosphoesterase |
| TON_0046 | 42118 | 42972 | + | 3104.21 | 3909.32 | 3502.64 | 1.26 | 1.13 | hypothetical protein |
| TON_0047 | 42953 | 43543 | - | 1114.24 | 680.40 | 654.78 | 0.61 | 0.59 | hypothetical protein |
| TON_0048 | 43637 | 44659 | + | 1149.45 | 752.53 | 553.59 | 0.65 | 0.48 | RNA 3'-terminal-phosphate cyclase |
| TON_0049 | 44714 | 45553 | + | 365.74 | 387.09 | 143.71 | 1.06 | 0.39 | Membrane protein |
| TON_0050 | 45563 | 46408 | - | 966.59 | 861.93 | 1165.84 | 0.89 | 1.21 | metal-dependent hydrolase |
| TON_0051 | 46419 | 46742 | - | 1353.90 | 1146.83 | 1398.27 | 0.85 | 1.03 | hypothetical protein |
| TON_0052 | 46791 | 48038 | - | 578.13 | 1503.86 | 821.95 | 2.60 | 1.42 | hydrogenase subunit alpha |
| TON_0053 | 48035 | 48775 | - | 194.23 | 400.31 | 344.62 | 2.06 | 1.77 | Sulfhydrogenase II, delta chain |
| TON_0054 | 48772 | 49647 | - | 230.57 | 382.28 | 288.16 | 1.66 | 1.25 | cytochrome-c3 hydrogenase subunit gamma |
| TON_0055 | 49644 | 50648 | - | 681.49 | 1307.92 | 835.15 | 1.92 | 1.23 | Sulfhydrogenase II, beta chain |
| TON_0056 | 50799 | 51635 | + | 3086.03 | 1216.55 | 1475.99 | 0.39 | 0.48 | ferredoxin-NADP(+) reductase subunit alpha |
| TON_0057 | 51635 | 53059 | + | 3532.41 | 1597.63 | 1959.93 | 0.45 | 0.55 | gltB-1 glutamate synthase small chain |
| TON_0058 | 53132 | 54034 | - | 8306.28 | 1204.53 | 1904.20 | 0.15 | 0.23 | asparagine synthetase A |
| TON_0059 | 54235 | 54828 | + | 1684.43 | 1389.66 | 2138.10 | 0.83 | 1.27 | ATPase |

| | | | | | | | | | |
|---|---|---|---|---|---|---|---|---|---|
| TON_0060 | 54831 | 55592 | - | 1780.97 | 1192.51 | 1901.27 | 0.67 | 1.07 | hypothetical protein |
| TON_0061 | 55623 | 56777 | - | 11656.96 | 8449.76 | 7118.21 | 0.72 | 0.61 | serine-glyoxylate aminotransferase |
| TON_0062 | 56942 | 58108 | + | 671.27 | 548.17 | 445.80 | 0.82 | 0.66 | major facilitator superfamily permease |
| TON_0063 | 58197 | 58745 | + | 4873.82 | 3826.38 | 5425.18 | 0.79 | 1.11 | rpl10e 50S ribosomal protein L10e |
| TON_0064 | 58963 | 60105 | + | 8949.15 | 6069.55 | 3688.88 | 0.68 | 0.41 | hypothetical protein |
| TON_0065 | 60403 | 61320 | + | 10515.45 | 5922.89 | 12231.76 | 0.56 | 1.16 | hypothetical protein |
| TON_0066 | 61322 | 62365 | + | 6642.30 | 6800.44 | 10490.33 | 1.02 | 1.58 | rpl3p 50S ribosomal protein L3P |
| TON_0067 | 62376 | 63143 | + | 6228.86 | 6653.78 | 10474.20 | 1.07 | 1.68 | rpl4lp 50S ribosomal protein L4P |
| TON_0068 | 63150 | 63410 | + | 11424.11 | 7289.71 | 14197.55 | 0.64 | 1.24 | rplW 50S ribosomal protein L23 |
| TON_0069 | 63424 | 64143 | + | 5008.98 | 6168.12 | 9905.95 | 1.23 | 1.98 | rpl2p 50S ribosomal protein L2 |
| TON_0070 | 64146 | 64547 | + | 1564.03 | 3482.57 | 3177.82 | 2.23 | 2.03 | rps19p 30S ribosomal protein S19 |
| TON_0071 | 64558 | 65028 | + | 1807.10 | 3054.61 | 3627.29 | 1.69 | 2.01 | rpl22p 50S ribosomal protein L22 |
| TON_0072 | 65034 | 65660 | + | 1927.49 | 4193.02 | 3841.40 | 2.18 | 1.99 | rps3p 30S ribosomal protein S3 |
| TON_0073 | 65657 | 65857 | + | 721.25 | 2255.19 | 1761.95 | 3.13 | 2.44 | 50S ribosomal protein L29 |
| TON_0074 | 65864 | 66160 | + | 3583.52 | 11117.29 | 5603.35 | 3.10 | 1.56 | translation initiation factor Sui1 |
| TON_0075 | 66420 | 66755 | + | 2746.42 | 2611.02 | 3822.33 | 0.95 | 1.39 | rps17p 30S ribosomal protein S17 |
| TON_0076 | 66761 | 67186 | + | 4943.10 | 4244.72 | 7015.55 | 0.86 | 1.42 | rpl14p 50S ribosomal protein L14 |
| TON_0077 | 67197 | 67562 | + | 3619.87 | 4721.96 | 6021.29 | 1.30 | 1.66 | rpl24p 50S ribosomal protein L24 |
| TON_0078 | 67562 | 68293 | + | 2846.37 | 3595.57 | 4687.55 | 1.26 | 1.65 | 30S ribosomal protein S4e |
| TON_0079 | 68305 | 68856 | + | 3614.19 | 5438.43 | 7510.48 | 1.50 | 2.08 | rpl5p 50S ribosomal protein L5 |
| TON_0080 | 68858 | 69028 | + | 1907.05 | 2508.84 | 3254.08 | 1.32 | 1.71 | rps14P 30S ribosomal protein S14 |
| TON_0081 | 69040 | 69432 | + | 2621.48 | 4550.06 | 5532.96 | 1.74 | 2.11 | rps8p 30S ribosomal protein S8 |
| TON_0082 | 69443 | 69997 | + | 2986.08 | 6439.80 | 5557.89 | 2.16 | 1.86 | rpl6p 50S ribosomal protein L6 |
| TON_0083 | 70008 | 70388 | + | 2226.21 | 4105.27 | 4685.35 | 1.84 | 2.10 | rpl32e 50S ribosomal protein L32e |
| TON_0084 | 70399 | 70845 | + | 5924.46 | 9297.26 | 11584.32 | 1.57 | 1.96 | rpl19e 50S ribosomal protein L19e |
| TON_0085 | 70856 | 71461 | + | 3371.12 | 5939.72 | 5305.66 | 1.76 | 1.57 | rpl18p 50S ribosomal protein L18 |
| TON_0086 | 71458 | 72165 | + | 2823.66 | 6241.45 | 5515.36 | 2.21 | 1.95 | rps5p 30S ribosomal protein S5 |
| TON_0087 | 72177 | 72644 | + | 9755.59 | 17799.92 | 12275.75 | 1.82 | 1.26 | rpl30p 50S ribosomal protein L30 |
| TON_0088 | 72656 | 73102 | + | 12278.25 | 26725.72 | 19204.78 | 2.18 | 1.56 | rpl15p 50S ribosomal protein L15 |
| TON_0089 | 73138 | 74583 | + | 952.96 | 7024.04 | 4342.93 | 7.37 | 4.56 | preprotein translocase subunit SecY |
| TON_0090 | 74659 | 75249 | + | 28088.92 | 14699.63 | 24359.40 | 0.52 | 0.87 | adenylate kinase |
| TON_0091 | 75242 | 75769 | + | 7198.85 | 6378.49 | 7636.60 | 0.89 | 1.06 | membrane protein |

| | | | | | | | | | |
|---|---|---|---|---|---|---|---|---|---|
| TON_0092 | 75812 | 76084 | + | 5930.13 | 8289.88 | 9925.01 | 1.40 | 1.67 | rpl34e 50S ribosomal protein L34e |
| TON_0093 | 76094 | 76687 | + | 20382.35 | 21960.49 | 29978.15 | 1.08 | 1.47 | cytidylate kinase |
| TON_0094 | 76688 | 76939 | + | 1936.58 | 1019.41 | 3908.12 | 0.53 | 2.02 | 50S ribosomal protein L14e |
| TON_0095 | 77020 | 77727 | + | 3351.82 | 2006.35 | 2509.85 | 0.60 | 0.75 | short-chain alcohol dehydrogenase |
| TON_0096 | 77788 | 78792 | + | 3608.51 | 2255.19 | 3415.39 | 0.62 | 0.95 | H/ACA RNA-protein complex component Cbf5p |
| TON_0097 | 79038 | 79868 | + | 673.54 | 427.96 | 378.35 | 0.64 | 0.56 | hypothetical protein |
| TON_0098 | 79865 | 80839 | + | 506.58 | 1821.22 | 488.33 | 3.60 | 0.96 | Kef-type K+ transport system membrane protein |
| TON_0099 | 81381 | 81683 | + | 216.94 | 372.66 | 73.32 | 1.72 | 0.34 | hypothetical protein |
| TON_0100 | 81899 | 82201 | + | 67.01 | 62.51 | 12.46 | 0.93 | 0.19 | protein MmpL11 |
| TON_0101 | 82480 | 83109 | + | 2688.49 | 924.44 | 3598.70 | 0.34 | 1.34 | ribosomal RNA small subunit methyltransferase C |
| TON_0102 | 83221 | 83670 | + | 15226.85 | 10612.39 | 17649.60 | 0.70 | 1.16 | rps13p 30S ribosomal protein S13 |
| TON_0103 | 83681 | 84223 | + | 5258.86 | 6150.09 | 8085.34 | 1.17 | 1.54 | rps4p 30S ribosomal protein S4 |
| TON_0104 | 84220 | 84639 | + | 1767.34 | 3370.77 | 3317.14 | 1.91 | 1.88 | rps11p 30S ribosomal protein S11 |
| TON_0105 | 84668 | 85450 | + | 5367.90 | 7849.90 | 10732.30 | 1.46 | 2.00 | DNA-directed RNA polymerase subunit D |
| TON_0106 | 85593 | 85955 | + | 6616.17 | 6709.08 | 10452.94 | 1.01 | 1.58 | 50S ribosomal protein L18e |
| TON_0107 | 85962 | 86390 | + | 11995.43 | 9210.71 | 15719.74 | 0.77 | 1.31 | rpl13p 50S ribosomal protein L13 |
| TON_0108 | 86402 | 86809 | + | 5196.39 | 5306.20 | 7976.09 | 1.02 | 1.53 | rps9p 30S ribosomal protein S9 |
| TON_0109 | 86843 | 87040 | + | 382.77 | 495.28 | 659.91 | 1.29 | 1.72 | DNA-directed RNA polymerase subunit N |
| TON_0110 | 87156 | 87329 | + | 4772.73 | 3422.46 | 6044.02 | 0.72 | 1.27 | rpoK DNA-directed RNA polymerase subunit K |
| TON_0111 | 87336 | 88361 | + | 3911.78 | 7195.94 | 5165.61 | 1.84 | 1.32 | hypothetical protein |
| TON_0112 | 88376 | 88981 | + | 2168.29 | 3784.30 | 4069.43 | 1.75 | 1.88 | rps2P 30S ribosomal protein S2 |
| TON_0113 | 89168 | 89326 | + | 773.50 | 1087.93 | 2257.62 | 1.41 | 2.92 | rpl40e 50S ribosomal protein L40e |
| TON_0114 | 89425 | 89751 | - | 405.49 | 1777.95 | 261.03 | 4.38 | 0.64 | transcription regulator, PadR-like family |
| TON_0115 | 89876 | 90175 | + | 364.60 | 803.02 | 178.18 | 2.20 | 0.49 | hypothetical protein |
| TON_0116 | 90200 | 91522 | + | 211.26 | 437.57 | 313.09 | 2.07 | 1.48 | hypothetical protein |
| TON_0117 | 91494 | 92255 | - | 3514.24 | 958.10 | 2240.02 | 0.27 | 0.64 | hypothetical protein |
| TON_0118 | 92811 | 93263 | + | 927.97 | 573.42 | 511.06 | 0.62 | 0.55 | Lrp/AsnC family transcriptional regulator |
| TON_0119 | 93249 | 93575 | - | 728.06 | 549.37 | 665.77 | 0.75 | 0.91 | hypothetical protein |
| TON_0120 | 93581 | 94297 | - | 2955.41 | 2303.28 | 2758.41 | 0.78 | 0.93 | adenine phosphoribosyltransferase |
| TON_0121 | 94352 | 94852 | - | 1708.28 | 1874.12 | 1841.88 | 1.10 | 1.08 | hypothetical protein |
| TON_0122 | 94857 | 95096 | - | 5843.81 | 4628.20 | 6966.43 | 0.79 | 1.19 | Lrp/AsnC family transcription regulator |
| TON_0123 | 95093 | 96439 | - | 13339.11 | 12022.49 | 21528.39 | 0.90 | 1.61 | signal recognition particle protein Srp54 |

| | | | | | | | | | |
|---|---|---|---|---|---|---|---|---|---|
| TON_0124 | 96710 | 96817 | + | 4906.76 | 1511.08 | 3749.01 | 0.31 | 0.76 | hypothetical protein |
| TON_0125 | 96859 | 97710 | + | 4990.81 | 4298.81 | 4089.96 | 0.86 | 0.82 | hypothetical protein |
| TON_0126 | 97796 | 98005 | + | 1152.86 | 786.19 | 139.31 | 0.68 | 0.12 | hypothetical protein |
| TON_0127 | 98098 | 99399 | + | 5493.98 | 5558.64 | 3242.35 | 1.01 | 0.59 | hypothetical protein |
| TON_0128 | 99440 | 100243 | + | 3720.96 | 4319.25 | 4177.22 | 1.16 | 1.12 | 5'-methylthioadenosine phosphorylase |
| TON_0129 | 100367 | 101704 | + | 4539.89 | 11092.04 | 3403.66 | 2.44 | 0.75 | NADH:polysulfide oxidoreductase |
| TON_0130 | 102152 | 102877 | - | 2191.00 | 3454.92 | 4912.65 | 1.58 | 2.24 | hypothetical protein |
| TON_0131 | 103106 | 103939 | - | 5491.71 | 5647.60 | 4925.85 | 1.03 | 0.90 | uridine phosphorylase |
| TON_0132 | 104050 | 104925 | + | 6237.94 | 4005.49 | 2513.52 | 0.64 | 0.40 | hypothetical protein |
| TON_0133 | 104986 | 105990 | + | 4518.31 | 2971.66 | 2652.10 | 0.66 | 0.59 | mevalonate kinase |
| TON_0134 | 105987 | 106763 | + | 2503.36 | 2336.94 | 2357.34 | 0.93 | 0.94 | amino acid kinase |
| TON_0135 | 106821 | 107945 | + | 2572.64 | 1804.40 | 2836.14 | 0.70 | 1.10 | isopentenyl pyrophosphate isomerase |
| TON_0136 | 108093 | 109415 | + | 1440.22 | 1757.51 | 2126.37 | 1.22 | 1.48 | hydrolase |
| TON_0137 | 109485 | 110513 | + | 4111.68 | 4717.15 | 4908.25 | 1.15 | 1.19 | bifunctional short-chain isoprenyl diphosphate synthase |
| TON_0138 | 110513 | 110806 | + | 47.70 | 144.26 | 103.39 | 3.02 | 2.17 | hypothetical protein |
| TON_0139 | 110915 | 111121 | + | 0.00 | 7.21 | 2.20 | - | - | membrane-associated metallopeptidase, M50 family |
| TON_0140 | 111142 | 111951 | - | 148.79 | 114.20 | 112.18 | 0.77 | 0.75 | hypothetical protein |
| TON_0141 | 113674 | 116577 | - | 24782.53 | 19351.87 | 21045.19 | 0.78 | 0.85 | leuS leucyl-tRNA synthetase |
| TON_0142 | 116865 | 118955 | + | 1256.22 | 2288.85 | 3560.57 | 1.82 | 2.83 | hypothetical protein |
| TON_0143 | 118962 | 120032 | - | 1494.74 | 1114.37 | 1613.84 | 0.75 | 1.08 | aminopeptidase P |
| TON_0144 | 120160 | 120744 | + | 5224.79 | 1645.71 | 7927.69 | 0.31 | 1.52 | 50S ribosomal protein L15e |
| TON_0145 | 120838 | 121293 | + | 2437.48 | 1573.59 | 2005.39 | 0.65 | 0.82 | hypothetical protein |
| TON_0146 | 121290 | 121961 | + | 3599.42 | 1262.24 | 2882.33 | 0.35 | 0.80 | ribonuclease P protein component 3 |
| TON_0147 | 121958 | 123289 | - | 743.96 | 253.65 | 1195.17 | 0.34 | 1.61 | tryptophan synthase subunit beta |
| TON_0148 | 123861 | 124868 | + | 382.77 | 239.22 | 456.07 | 0.62 | 1.19 | hypothetical protein |
| TON_0149 | 124865 | 125311 | - | 3176.90 | 2116.95 | 2415.26 | 0.67 | 0.76 | hypothetical protein |
| TON_0150 | 125393 | 125809 | + | 2679.41 | 1701.01 | 1305.15 | 0.63 | 0.49 | nickel responsive regulator |
| TON_0151 | 125832 | 126245 | + | 1741.22 | 1095.14 | 757.43 | 0.63 | 0.43 | amino acid permease |
| TON_0152 | 126242 | 128191 | - | 5221.38 | 3303.45 | 5366.52 | 0.63 | 1.03 | fibronectin-binding protein |
| TON_0153 | 128316 | 130013 | + | 1386.84 | 1132.41 | 2729.09 | 0.82 | 1.97 | alpha-amylase |
| TON_0154 | 130368 | 130685 | + | 2578.32 | 1804.40 | 920.94 | 0.70 | 0.36 | nitrogen regulatory protein P-II |
| TON_0155 | 130682 | 131410 | - | 1340.27 | 891.98 | 1269.22 | 0.67 | 0.95 | alpha-glucosidase |

| | | | | | | | | | |
|---|---|---|---|---|---|---|---|---|---|
| TON_0156 | 131567 | 133150 | - | 2237.57 | 2311.69 | 2927.79 | 1.03 | 1.31 | sodium-dependent transporter |
| TON_0157 | 133762 | 135021 | + | 80776.36 | 202847.18 | 215542.76 | 2.51 | 2.67 | glutamate dehydrogenase |
| TON_0158 | 135158 | 136186 | - | 550.87 | 1868.11 | 2737.88 | 3.39 | 4.97 | branched-chain amino acid transport ATP-binding protein livM |
| TON_0159 | 136183 | 137088 | - | 169.24 | 592.65 | 720.03 | 3.50 | 4.25 | branched-chain amino acid transport ATP-binding protein livH |
| TON_0160 | 137075 | 137848 | - | 387.32 | 774.17 | 1509.72 | 2.00 | 3.90 | branched-chain amino acid transport ATP-binding protein livF |
| TON_0161 | 137851 | 138618 | - | 515.66 | 1026.62 | 2344.87 | 1.99 | 4.55 | branched-chain amino acid transport ATP-binding protein livG |
| TON_0162 | 138666 | 139952 | - | 838.24 | 4244.72 | 8575.87 | 5.06 | 10.23 | branched-chain amino acid ABC transporter substrate-binding protein |
| TON_0163 | 140219 | 141382 | - | 3379.08 | 1685.38 | 3645.62 | 0.50 | 1.08 | hypothetical protein |
| TON_0164 | 141492 | 142103 | + | 6828.57 | 5080.20 | 3567.17 | 0.74 | 0.52 | proteasome subunit beta |
| TON_0165 | 142114 | 144060 | + | 4805.67 | 8548.34 | 7728.99 | 1.78 | 1.61 | cleavage and polyadenylation specficity factor subunit-like protein |
| TON_0166 | 144066 | 145400 | + | 806.43 | 2989.69 | 1835.28 | 3.71 | 2.28 | threonine synthase |
| TON_0167 | 145449 | 145793 | + | 5906.28 | 2923.58 | 5227.20 | 0.49 | 0.89 | membrane protein |
| TON_0168 | 145798 | 146481 | + | 2574.91 | 1854.88 | 2849.34 | 0.72 | 1.11 | ribose-5-phosphate isomerase A |
| TON_0169 | 146837 | 147436 | - | 746.24 | 694.83 | 396.68 | 0.93 | 0.53 | hypothetical protein |
| TON_0170 | 147490 | 148698 | + | 7117.07 | 4390.17 | 4818.06 | 0.62 | 0.68 | hypothetical protein |
| TON_0171 | 148806 | 148976 | + | 798.48 | 872.75 | 312.36 | 1.09 | 0.39 | hypothetical protein |
| TON_0172 | 148982 | 151303 | - | 283.96 | 1142.02 | 788.96 | 4.02 | 2.78 | TRAP-type transporter |
| TON_0173 | 151305 | 151754 | - | 193.09 | 189.94 | 236.10 | 0.98 | 1.22 | hypothetical protein |
| TON_0174 | 151769 | 152758 | - | 1231.23 | 3402.02 | 3817.93 | 2.76 | 3.10 | TRAP-type transporter |
| TON_0175 | 152983 | 153801 | + | 669.00 | 631.12 | 676.77 | 0.94 | 1.01 | hypothetical protein |
| TON_0176 | 153895 | 155043 | + | 19138.63 | 15505.05 | 24069.04 | 0.81 | 1.26 | cell division protein FtsZ |
| TON_0177 | 155082 | 155267 | + | 6218.63 | 4812.12 | 8987.94 | 0.77 | 1.45 | secE preprotein translocase subunit SecE |
| TON_0178 | 155288 | 155746 | + | 4240.03 | 6216.21 | 8831.03 | 1.47 | 2.08 | nusG transcription antitermination protein NusG |
| TON_0179 | 155776 | 156267 | + | 5563.26 | 8181.69 | 12606.44 | 1.47 | 2.27 | rpl11p 50S ribosomal protein L11 |
| TON_0180 | 156347 | 156997 | + | 9529.56 | 12719.72 | 26331.05 | 1.33 | 2.76 | rpl1P 50S ribosomal protein L1P |
| TON_0181 | 157003 | 158022 | + | 16922.64 | 12375.91 | 29997.21 | 0.73 | 1.77 | rplP0 acidic ribosomal protein P0 |
| TON_0182 | 158092 | 158409 | + | 3107.61 | 6138.07 | 6085.08 | 1.98 | 1.96 | rpl12p 50S ribosomal protein L12 |
| TON_0183 | 158447 | 158914 | - | 327.12 | 397.90 | 118.05 | 1.22 | 0.36 | hypothetical protein |
| TON_0184 | 159674 | 160141 | + | 80.64 | 48.09 | 72.59 | 0.60 | 0.90 | dcd deoxycytidine triphosphate deaminase |
| TON_0185 | 160492 | 160695 | + | 6799.04 | 19897.63 | 9438.88 | 2.93 | 1.39 | histone A |
| TON_0186 | 160762 | 162036 | + | 6329.94 | 3197.66 | 2731.29 | 0.51 | 0.43 | hypothetical protein |

| | | | | | | | | | |
|---|---|---|---|---|---|---|---|---|---|
| TON_0187 | 162111 | 162512 | + | 11736.46 | 19599.50 | 9710.18 | 1.67 | 0.83 | hypothetical protein |
| TON_0188 | 162509 | 163903 | + | 20409.61 | 20198.16 | 27104.61 | 0.99 | 1.33 | DNA primase |
| TON_0189 | 164188 | 165909 | + | 8088.20 | 5532.20 | 7551.55 | 0.68 | 0.93 | gltX glutamyl-tRNA synthetase |
| TON_0190 | 165961 | 167247 | - | 319.17 | 390.69 | 478.80 | 1.22 | 1.50 | membrane protein |
| TON_0191 | 167384 | 169885 | - | 3348.41 | 7238.02 | 14279.67 | 2.16 | 4.26 | maltodextrin phosphorylase |
| TON_0192 | 169996 | 171873 | - | 6702.49 | 8533.91 | 8376.43 | 1.27 | 1.25 | phosphoenolpyruvate carboxykinase |
| TON_0193 | 172012 | 172218 | + | 13.63 | 4.81 | 7.33 | 0.35 | 0.54 | NDP-sugar synthase, N-terminus |
| TON_0194 | 172558 | 172758 | + | 2824.79 | 2797.35 | 4023.97 | 0.99 | 1.42 | NDP-sugar synthase, C-terminus |
| TON_0195 | 172769 | 173476 | + | 2526.07 | 2793.75 | 4338.53 | 1.11 | 1.72 | phosphomannomutase |
| TON_0196 | 173585 | 174202 | + | 1548.13 | 1179.29 | 2306.01 | 0.76 | 1.49 | tmk thymidylate kinase |
| TON_0197 | 174424 | 175293 | - | 525.89 | 1636.10 | 850.55 | 3.11 | 1.62 | hydrolase |
| TON_0198 | 175303 | 176613 | - | 316.89 | 742.92 | 651.84 | 2.34 | 2.06 | permease |
| TON_0199 | 176708 | 178033 | - | 20.44 | 18.03 | 22.00 | 0.88 | 1.08 | permease |
| TON_0200 | 178120 | 178857 | + | 121.53 | 289.71 | 154.71 | 2.38 | 1.27 | glycerophosphoryl diester phosphodiesterase |
| TON_0201 | 178841 | 179590 | + | 713.30 | 1137.21 | 787.49 | 1.59 | 1.10 | glycerophosphoryl diester phosphodiesterase |
| TON_0202 | 179650 | 181131 | + | 651.96 | 539.76 | 810.95 | 0.83 | 1.24 | glpK glycerol kinase |
| TON_0203 | 181334 | 182824 | + | 7289.72 | 2377.81 | 3569.37 | 0.33 | 0.49 | anaerobic glycerol 3-phosphate dehydrogenase |
| TON_0204 | 182821 | 184077 | + | 10265.57 | 5112.65 | 5702.34 | 0.50 | 0.56 | NADH oxidase |
| TON_0205 | 184074 | 184424 | + | 4581.91 | 3048.60 | 3221.82 | 0.67 | 0.70 | molybdopterin oxidoreductase |
| TON_0206 | 184469 | 184912 | - | 1021.11 | 976.13 | 398.14 | 0.96 | 0.39 | hypothetical protein |
| TON_0207 | 184932 | 185138 | - | 1175.58 | 616.69 | 1025.79 | 0.52 | 0.87 | hypothetical protein |
| TON_0208 | 185131 | 185838 | - | 1667.39 | 1726.26 | 1641.70 | 1.04 | 0.98 | membrane protein |
| TON_0209 | 185957 | 186637 | - | 1602.65 | 1747.89 | 828.55 | 1.09 | 0.52 | metallophosphoesterase |
| TON_0210 | 186729 | 187007 | + | 5394.03 | 3126.74 | 1995.85 | 0.58 | 0.37 | hypothetical protein |
| TON_0211 | 186994 | 187683 | - | 458.87 | 300.53 | 442.87 | 0.65 | 0.97 | HAD-superfamily hydrolase |
| TON_0212 | 187871 | 189157 | + | 263.51 | 332.99 | 1059.52 | 1.26 | 4.02 | hypothetical protein |
| TON_0213 | 189304 | 190644 | + | 1520.87 | 2661.51 | 2215.09 | 1.75 | 1.46 | glycine dehydrogenase subunit 1 |
| TON_0214 | 190645 | 192153 | + | 4497.86 | 9821.39 | 5198.61 | 2.18 | 1.16 | glycine dehydrogenase subunit 2 |
| TON_0215 | 192267 | 192575 | + | 216.94 | 217.59 | 208.97 | 1.00 | 0.96 | hypothetical protein |
| TON_0216 | 194333 | 194581 | + | 1893.42 | 1096.34 | 4664.08 | 0.58 | 2.46 | rpoH DNA-directed RNA polymerase subunit H |
| TON_0217 | 194598 | 197960 | + | 11453.64 | 18420.22 | 39591.54 | 1.61 | 3.46 | DNA-directed RNA polymerase subunit B |
| TON_0218 | 197966 | 200683 | + | 7414.66 | 13523.95 | 21202.10 | 1.82 | 2.86 | DNA-directed RNA polymerase subunit A' |

| | | | | | | | | | |
|---|---|---|---|---|---|---|---|---|---|
| TON_0219 | 200689 | 201864 | + | 6816.08 | 8898.16 | 12597.64 | 1.31 | 1.85 | DNA-directed RNA polymerase subunit A'' |
| TON_0220 | 201878 | 202180 | + | 4103.73 | 3285.42 | 7439.36 | 0.80 | 1.81 | 50S ribosomal protein L30e |
| TON_0221 | 202180 | 202617 | + | 2949.73 | 3099.09 | 4390.59 | 1.05 | 1.49 | transcription elongation factor NusA-like protein |
| TON_0222 | 202628 | 203071 | + | 3908.37 | 8247.80 | 8231.25 | 2.11 | 2.11 | rps12P 30S ribosomal protein S12 |
| TON_0223 | 203077 | 203724 | + | 5707.51 | 8502.66 | 12338.08 | 1.49 | 2.16 | 30S ribosomal protein S7 |
| TON_0224 | 203960 | 205084 | - | 3233.69 | 10126.73 | 2862.53 | 3.13 | 0.89 | phosphodiesterase |
| TON_0225 | 205090 | 205425 | - | 514.53 | 1461.79 | 581.45 | 2.84 | 1.13 | hypothetical protein |
| TON_0226 | 205422 | 205622 | - | 2869.09 | 2518.46 | 2782.61 | 0.88 | 0.97 | hypothetical protein |
| TON_0227 | 205689 | 206954 | - | 1813.91 | 3107.50 | 2841.27 | 1.71 | 1.57 | hypothetical protein |
| TON_0228 | 207515 | 207718 | + | 1091.53 | 183.93 | 137.85 | 0.17 | 0.13 | sulfur carrier protein ThiS |
| TON_0229 | 207885 | 209315 | + | 18229.97 | 3690.53 | 2426.99 | 0.20 | 0.13 | hypothetical protein |
| TON_0230 | 209322 | 210239 | + | 1432.27 | 1341.58 | 445.80 | 0.94 | 0.31 | hypothetical protein |
| TON_0231 | 210287 | 211486 | - | 3551.72 | 1994.33 | 2312.61 | 0.56 | 0.65 | alanine aminotransferase |
| TON_0232 | 211715 | 211963 | + | 84.05 | 120.21 | 66.72 | 1.43 | 0.79 | membrane protein |
| TON_0233 | 211960 | 212541 | + | 243.07 | 199.55 | 214.10 | 0.82 | 0.88 | membrane protein |
| TON_0234 | 212603 | 212782 | + | 1515.19 | 9827.40 | 5128.22 | 6.49 | 3.38 | hypothetical protein |
| TON_0235 | 213001 | 213285 | + | 11177.64 | 7515.71 | 9310.57 | 0.67 | 0.83 | rpl44e 50S ribosomal protein L44e |
| TON_0236 | 213289 | 213486 | + | 370.28 | 513.31 | 457.54 | 1.39 | 1.24 | rps27e 30S ribosomal protein S27e |
| TON_0237 | 213583 | 214410 | + | 12776.88 | 12444.44 | 10917.81 | 0.97 | 0.85 | translation initiation factor IF-2 |
| TON_0238 | 214422 | 214598 | + | 1607.19 | 2095.31 | 1972.39 | 1.30 | 1.23 | H/ACA RNA-protein complex component Nop10p |
| TON_0239 | 214603 | 215403 | + | 10948.20 | 10878.06 | 10426.54 | 0.99 | 0.95 | hypothetical protein |
| TON_0240 | 215538 | 216152 | + | 3000.85 | 1151.64 | 1035.32 | 0.38 | 0.35 | cobalt ABC transporter permease CbiM |
| TON_0241 | 216149 | 216397 | + | 345.29 | 194.74 | 153.98 | 0.56 | 0.45 | membrane protein |
| TON_0242 | 216399 | 217058 | + | 3666.44 | 1679.37 | 1704.76 | 0.46 | 0.46 | cobalt ABC transporter permease CbiQ |
| TON_0243 | 217055 | 217855 | + | 3636.91 | 2668.73 | 2296.48 | 0.73 | 0.63 | cobalt ABC transporter ATPase |
| TON_0244 | 217845 | 219212 | - | 2386.37 | 3159.19 | 4094.36 | 1.32 | 1.72 | phosphohexomutase |
| TON_0245 | 219214 | 220596 | - | 1414.10 | 1707.02 | 1962.13 | 1.21 | 1.39 | bifunctional mannose-6-phosphate isomerase/mannose-1-phosphate guanylyl transferase |
| TON_0246 | 220672 | 222033 | - | 1161.95 | 2758.89 | 2700.49 | 2.37 | 2.32 | ADP-dependent glucokinase |
| TON_0247 | 222108 | 222677 | - | 2183.05 | 5951.74 | 3564.97 | 2.73 | 1.63 | glucose-6-phosphate isomerase |
| TON_0248 | 222743 | 223339 | - | 3752.76 | 3611.19 | 6309.45 | 0.96 | 1.68 | peptidase |
| TON_0249 | 223336 | 223515 | - | 1148.32 | 1524.30 | 1451.80 | 1.33 | 1.26 | RNA-binding protein |

| | | | | | | | | | |
|---|---|---|---|---|---|---|---|---|---|
| TON_0250 | 223565 | 223750 | - | 2947.46 | 2931.99 | 3125.76 | 0.99 | 1.06 | hypothetical protein |
| TON_0251 | 223747 | 224448 | - | 1832.08 | 2110.94 | 1945.99 | 1.15 | 1.06 | signaling protein |
| TON_0252 | 224445 | 225380 | - | 2772.55 | 2134.98 | 2346.34 | 0.77 | 0.85 | ribonuclease Z |
| TON_0253 | 225469 | 226428 | + | 33538.60 | 33552.61 | 26744.60 | 1.00 | 0.80 | hypothetical protein |
| TON_0254 | 226396 | 227172 | - | 805.30 | 234.42 | 347.55 | 0.29 | 0.43 | ABC-type iron(III)-siderophore transport system, ATPase component |
| TON_0255 | 227169 | 228203 | - | 791.67 | 421.95 | 329.95 | 0.53 | 0.42 | ABC-type iron(III)-siderophore transport system permease |
| TON_0256 | 228209 | 229345 | - | 13410.67 | 2079.68 | 3214.48 | 0.16 | 0.24 | hemV-3 iron (III) ABC transporter ATP-binding protein |
| TON_0257 | 229347 | 230045 | - | 10939.12 | 976.13 | 2097.77 | 0.09 | 0.19 | fwdE-like tungsten formylmethanofuran dehydrogenase |
| TON_0258 | 230165 | 230539 | + | 3394.98 | 8479.82 | 6257.39 | 2.50 | 1.84 | iron-molybdenum cofactor-binding protein |
| TON_0259 | 230598 | 231623 | + | 127.21 | 269.28 | 231.70 | 2.12 | 1.82 | mae1-like C4-dicarboxylate transporter |
| TON_0260 | 231958 | 232845 | - | 2127.40 | 1675.77 | 1768.55 | 0.79 | 0.83 | cobalt/zinc/cadmium cation efflux pump protein |
| TON_0261 | 232997 | 233416 | + | 10949.34 | 6326.80 | 6901.90 | 0.58 | 0.63 | hypA hydrogenase nickel incorporation protein |
| TON_0262 | 233413 | 234159 | + | 6225.45 | 3941.78 | 3264.34 | 0.63 | 0.52 | chromosome partitioning ATPase |
| TON_0263 | 234185 | 234661 | + | 4877.23 | 2680.75 | 2747.42 | 0.55 | 0.56 | hydrogenase maturation protease HycI |
| TON_0264 | 234875 | 235456 | - | 1080.17 | 135.84 | 461.94 | 0.13 | 0.43 | mobA molybdopterin-guanine dinucleotide biosynthesis protein MobA |
| TON_0265 | 235453 | 235893 | - | 1257.36 | 339.00 | 491.26 | 0.27 | 0.39 | nucleotidyltransferase |
| TON_0266 | 235919 | 237484 | - | 849.60 | 171.90 | 69.66 | 0.20 | 0.08 | putative monovalent cation/H+ antiporter subunit D |
| TON_0267 | 237495 | 237980 | - | 1601.51 | 134.64 | 87.25 | 0.08 | 0.05 | hypothetical protein |
| TON_0268 | 237977 | 238369 | - | 132.89 | 15.63 | 4.40 | 0.12 | 0.03 | hypothetical protein |
| TON_0269 | 238366 | 239139 | - | 131.76 | 33.66 | 11.73 | 0.26 | 0.09 | Multisubunit Na+/H+ antiporter,putative MnhB subunit |
| TON_0270 | 239140 | 239394 | - | 43.16 | 3.61 | 3.67 | 0.08 | 0.08 | hypothetical protein |
| TON_0271 | 239387 | 239743 | - | 115.85 | 10.82 | 5.87 | 0.09 | 0.05 | Na+/H+ antiporter subunit |
| TON_0272 | 239740 | 240024 | - | 379.37 | 31.26 | 16.86 | 0.08 | 0.04 | Na+/H+ antiporter subunit MnhF |
| TON_0273 | 240029 | 240334 | - | 350.97 | 28.85 | 19.06 | 0.08 | 0.05 | hypothetical protein |
| TON_0274 | 240285 | 241085 | - | 1224.42 | 88.96 | 49.13 | 0.07 | 0.04 | Hydrogenase 4, component I or formate hydrogen lyase subunit 7 |
| TON_0275 | 241082 | 241699 | - | 1258.49 | 85.35 | 62.32 | 0.07 | 0.05 | Formate hydrogen lyase subunit 6 |
| TON_0276 | 241699 | 243450 | - | 3844.76 | 213.98 | 151.78 | 0.06 | 0.04 | Hydrogenase 4, component G or formate hydrogen lyase subunit 5 |
| TON_0277 | 243454 | 244356 | - | 938.19 | 52.89 | 12.46 | 0.06 | 0.01 | Hydrogenase 4, component C or formate hydrogen lyase subunit 4 |

| | | | | | | | | | |
|---|---|---|---|---|---|---|---|---|---|
| TON_0278 | 244367 | 246346 | - | 695.12 | 66.12 | 23.46 | 0.10 | 0.03 | Hydrogenase 4, component B or formate hydrogen lyase subunit 3 |
| TON_0279 | 246353 | 247804 | - | 241.93 | 69.72 | 5.13 | 0.29 | 0.02 | hydrogenase 4 subunit D |
| TON_0280 | 247819 | 248316 | - | 683.77 | 226.00 | 50.59 | 0.33 | 0.07 | Oxidoreductase iron-sulfur protein |
| TON_0281 | 248322 | 250391 | - | 7470.31 | 982.14 | 123.18 | 0.13 | 0.02 | fdhA formate dehydrogenase subunit alpha |
| TON_0282 | 250786 | 251337 | - | 112.45 | 219.99 | 404.74 | 1.96 | 3.60 | putative transcriptional regulator |
| TON_0283 | 251621 | 251866 | + | 10024.78 | 5973.38 | 8386.70 | 0.60 | 0.84 | hydrogenase expression/formation protein HypC |
| TON_0284 | 251868 | 252224 | + | 5337.23 | 3982.65 | 5552.76 | 0.75 | 1.04 | hydrogenase expression/formation protein hypD |
| TON_0285 | 252278 | 252970 | + | 5194.12 | 6163.31 | 6751.59 | 1.19 | 1.30 | hydrogenase expression/formation protein HypD |
| TON_0286 | 252934 | 255375 | + | 158479.19 | 25614.96 | 21920.67 | 0.16 | 0.14 | hydrogenase maturation protein HypF |
| TON_0287 | 255422 | 256432 | + | 66971.56 | 38619.58 | 17851.24 | 0.58 | 0.27 | hydrogenase expression/formation protein HypE |
| TON_0288 | 256511 | 256804 | + | 7373.77 | 438.78 | 352.68 | 0.06 | 0.05 | hypothetical protein |
| TON_0289 | 256801 | 258000 | + | 58950.38 | 5991.41 | 5292.46 | 0.10 | 0.09 | cysteine desulfurase |
| TON_0290 | 258042 | 259178 | - | 1589.02 | 3858.83 | 2064.78 | 2.43 | 1.30 | ATP-NAD kinase |
| TON_0291 | 259183 | 260628 | - | 4180.97 | 3530.65 | 4286.47 | 0.84 | 1.03 | membrane protein |
| TON_0293 | 260693 | 261037 | - | 900.71 | 717.67 | 324.09 | 0.80 | 0.36 | hypothetical protein |
| TON_0294 | 261034 | 261276 | - | 404.35 | 678.00 | 512.53 | 1.68 | 1.27 | protein Plut_1211 |
| TON_0295 | 261281 | 261880 | - | 5111.21 | 7552.97 | 6721.53 | 1.48 | 1.32 | hypothetical protein |
| TON_0296 | 262215 | 263825 | + | 2109.22 | 4352.91 | 1631.44 | 2.06 | 0.77 | iron(III)-siderophore ABC transporter periplasmic protein |
| TON_0297 | 263822 | 264766 | + | 57.93 | 580.63 | 112.92 | 10.02 | 1.95 | ABC-type iron(III)-siderophore transport system, pernease component |
| TON_0298 | 264753 | 265532 | + | 76.10 | 653.96 | 141.51 | 8.59 | 1.86 | ABC-type iron(III)-siderophore transport system, ATPase component |
| TON_0299 | 265571 | 266404 | + | 321.44 | 894.38 | 464.87 | 2.78 | 1.45 | hypothetical protein |
| TON_0300 | 266361 | 266732 | - | 73.83 | 31.26 | 60.12 | 0.42 | 0.81 | hypothetical protein |
| TON_0301 | 266920 | 267771 | + | 1573.12 | 14365.44 | 17574.08 | 9.13 | 11.17 | reductase 1 |
| TON_0302 | 267776 | 268567 | + | 332.80 | 6166.92 | 5742.66 | 18.53 | 17.26 | hypothetical protein |
| TON_0303 | 268577 | 269317 | + | 178.32 | 2253.99 | 1662.97 | 12.64 | 9.33 | hypothetical protein |
| TON_0304 | 269443 | 270687 | + | 212.40 | 305.34 | 606.38 | 1.44 | 2.85 | permease |
| TON_0305 | 270696 | 272021 | - | 1534.50 | 2674.74 | 2902.86 | 1.74 | 1.89 | NADH oxidase |
| TON_0306 | 272081 | 273247 | - | 3071.27 | 2175.85 | 3707.22 | 0.71 | 1.21 | nonsense-mediated mRNA decay protein |
| TON_0307 | 273301 | 273600 | - | 274.87 | 435.17 | 373.95 | 1.58 | 1.36 | hypothetical protein |
| TON_0308 | 273678 | 277922 | - | 689.44 | 501.29 | 535.26 | 0.73 | 0.78 | stetterlysin |

| | | | | | | | | | |
|---|---|---|---|---|---|---|---|---|---|
| TON_0309 | 278165 | 279202 | - | 329.39 | 551.78 | 463.40 | 1.68 | 1.41 | membrane protein |
| TON_0310 | 279260 | 281725 | + | 3626.68 | 5449.25 | 5824.79 | 1.50 | 1.61 | membrane-bound dolichyl-phosphate-mannose-protein mannosyltransferase |
| TON_0311 | 282016 | 284364 | + | 12035.19 | 28358.21 | 39078.27 | 2.36 | 3.25 | phosphoenolpyruvate synthase |
| TON_0312 | 284522 | 285907 | + | 297.59 | 492.87 | 465.60 | 1.66 | 1.56 | sodium-driven multidrug efflux pump protein |
| TON_0313 | 285947 | 287371 | + | 226.03 | 311.35 | 361.48 | 1.38 | 1.60 | sodium-driven multidrug efflux pump protein |
| TON_0314 | 287594 | 288262 | + | 3854.99 | 6317.19 | 3788.60 | 1.64 | 0.98 | upp uracil phosphoribosyltransferase |
| TON_0315 | 288910 | 291063 | + | 2867.95 | 1624.08 | 4724.21 | 0.57 | 1.65 | topA |
| TON_0316 | 291064 | 292254 | - | 8090.47 | 12645.19 | 10582.72 | 1.56 | 1.31 | geranylgeranyl hydrogenase |
| TON_0317 | 292251 | 292454 | - | 1369.80 | 1681.78 | 2061.11 | 1.23 | 1.50 | ferredoxin 2 |
| TON_0318 | 292438 | 293148 | - | 9197.90 | 8048.25 | 9275.37 | 0.88 | 1.01 | putative transcriptional regulator |
| TON_0319 | 293284 | 293964 | + | 5604.15 | 6353.25 | 20001.07 | 1.13 | 3.57 | protein disulfide oxidoreductase |
| TON_0320 | 296280 | 296537 | + | 10.22 | 26.45 | 16.13 | 2.59 | 1.58 | hypothetical protein |
| TON_0321 | 296530 | 297198 | - | 446.38 | 408.72 | 574.12 | 0.92 | 1.29 | hypothetical protein |
| TON_0322 | 297243 | 297965 | - | 103.36 | 176.71 | 161.31 | 1.71 | 1.56 | hypothetical protein |
| TON_0323 | 298165 | 299160 | - | 1230.10 | 2244.37 | 1684.23 | 1.82 | 1.37 | hypothetical protein |
| TON_0324 | 299157 | 299519 | - | 102.22 | 545.77 | 406.94 | 5.34 | 3.98 | hypothetical protein |
| TON_0325 | 299497 | 301260 | - | 335.07 | 472.44 | 367.35 | 1.41 | 1.10 | hypothetical protein |
| TON_0326 | 301262 | 302053 | - | 1065.40 | 621.50 | 527.93 | 0.58 | 0.50 | hypothetical protein |
| TON_0327 | 302216 | 302914 | - | 8473.24 | 10583.54 | 11529.32 | 1.25 | 1.36 | acetyl-CoA synthetase I subunit beta |
| TON_0328 | 303061 | 304359 | + | 4991.94 | 5240.08 | 5550.56 | 1.05 | 1.11 | hypothetical protein |
| TON_0329 | 304356 | 304589 | - | 895.03 | 2232.35 | 309.42 | 2.49 | 0.35 | hypothetical protein |
| TON_0330 | 304686 | 305216 | + | 1248.27 | 998.97 | 1555.92 | 0.80 | 1.25 | hypothetical protein |
| TON_0331 | 305268 | 308933 | + | 12564.48 | 20025.06 | 23570.44 | 1.59 | 1.88 | reverse gyrase |
| TON_0332 | 309336 | 310130 | + | 12165.81 | 12990.20 | 5903.24 | 1.07 | 0.49 | putative transcriptional regulator |
| TON_0333 | 310206 | 310418 | - | 4386.55 | 1377.64 | 2800.94 | 0.31 | 0.64 | hypothetical protein |
| TON_0334 | 310415 | 310744 | - | 4478.55 | 1299.50 | 2899.19 | 0.29 | 0.65 | hypothetical protein |
| TON_0335 | 310749 | 311474 | - | 1010.88 | 436.37 | 764.76 | 0.43 | 0.76 | arginase |
| TON_0336 | 311613 | 312833 | + | 3837.95 | 7918.42 | 3075.90 | 2.06 | 0.80 | bifunctional D-arabino 3-hexulose-6-phosphate formaldehyde lyase/phosphohexuloisomerase |
| TON_0337 | 312843 | 313598 | + | 5491.71 | 8710.62 | 5299.79 | 1.59 | 0.97 | hypothetical protein |
| TON_0338 | 313600 | 314244 | + | 2598.76 | 4075.22 | 2020.78 | 1.57 | 0.78 | hydrolase |
| TON_0339 | 314241 | 314420 | + | 4199.14 | 4119.69 | 2471.72 | 0.98 | 0.59 | hypothetical protein |

| | | | | | | | | | |
|---|---|---|---|---|---|---|---|---|---|
| TON_0340 | 314424 | 315230 | + | 3300.70 | 6746.35 | 6480.29 | 2.04 | 1.96 | hypothetical protein |
| TON_0341 | 315239 | 315859 | + | 2494.27 | 7953.28 | 4712.48 | 3.19 | 1.89 | metal-dependent phosphohydrolase |
| TON_0342 | 315962 | 316474 | + | 6061.89 | 3667.69 | 5360.65 | 0.61 | 0.88 | transcription elongation factor NusA-like protein |
| TON_0343 | 316489 | 317820 | + | 5676.85 | 7364.24 | 8545.07 | 1.30 | 1.51 | aspC aspartyl-tRNA synthetase |
| TON_0344 | 317881 | 319929 | + | 5788.16 | 12713.71 | 11703.10 | 2.20 | 2.02 | membrane protein |
| TON_0345 | 319989 | 320345 | + | 1184.66 | 1185.30 | 2044.25 | 1.00 | 1.73 | peptidyl-tRNA hydrolase |
| TON_0346 | 320349 | 321599 | + | 1501.56 | 1035.03 | 2194.56 | 0.69 | 1.46 | truD tRNA pseudouridine synthase D |
| TON_0347 | 321664 | 322371 | + | 5613.24 | 3906.92 | 3232.82 | 0.70 | 0.58 | phosphoglycolate phosphatase |
| TON_0348 | 322554 | 323702 | - | 823.47 | 492.87 | 301.36 | 0.60 | 0.37 | bifunctional carboxypeptidase/aminoacylase |
| TON_0349 | 324677 | 324964 | + | 2156.93 | 1099.95 | 2898.46 | 0.51 | 1.34 | hypothetical protein |
| TON_0350 | 324977 | 325357 | + | 2817.98 | 1078.31 | 3259.94 | 0.38 | 1.16 | hypothetical protein |
| TON_0351 | 325364 | 326263 | - | 36.35 | 45.68 | 50.59 | 1.26 | 1.39 | transmembrane efflux pump |
| TON_0352 | 326389 | 327990 | + | 6090.29 | 3309.46 | 6602.74 | 0.54 | 1.08 | pyrG CTP synthetase |
| TON_0353 | 327987 | 328877 | - | 1561.76 | 1948.65 | 1427.60 | 1.25 | 0.91 | membrane protein |
| TON_0354 | 328995 | 329387 | + | 12770.06 | 10274.59 | 19218.72 | 0.80 | 1.50 | 30S ribosomal protein S8e |
| TON_0355 | 329520 | 330446 | + | 8706.09 | 6055.12 | 5645.14 | 0.70 | 0.65 | hypothetical protein |
| TON_0356 | 330443 | 331519 | - | 3279.12 | 2214.32 | 3691.82 | 0.68 | 1.13 | GTPase |
| TON_0357 | 331582 | 332388 | - | 1763.93 | 1270.65 | 1183.43 | 0.72 | 0.67 | hydrolase |
| TON_0358 | 332446 | 333261 | + | 464.55 | 687.62 | 527.93 | 1.48 | 1.14 | bacitracin resistance protein bacA-like protein |
| TON_0359 | 333258 | 334517 | + | 3441.55 | 6200.58 | 4768.93 | 1.80 | 1.39 | sugar-phosphate nucleotydyltransferase |
| TON_0360 | 334514 | 335623 | + | 1202.84 | 3554.69 | 2031.78 | 2.96 | 1.69 | membrane protein |
| TON_0361 | 335632 | 336807 | - | 47066.26 | 31113.49 | 31290.63 | 0.66 | 0.66 | hypothetical protein |
| TON_0362 | 337129 | 338016 | - | 2185.32 | 1327.15 | 1702.56 | 0.61 | 0.78 | methionine aminopeptidase |
| TON_0363 | 338115 | 339596 | + | 1687.83 | 2320.11 | 2320.68 | 1.37 | 1.37 | Trk-type potassium transport system, membrane component |
| TON_0364 | 339593 | 340447 | + | 892.76 | 1411.30 | 1224.50 | 1.58 | 1.37 | cobalt transport ATP-binding protein |
| TON_0365 | 340407 | 341237 | - | 2080.83 | 3807.14 | 2542.11 | 1.83 | 1.22 | hypothetical protein |
| TON_0366 | 341221 | 341775 | - | 3904.96 | 4760.43 | 3683.75 | 1.22 | 0.94 | adenylate cyclase, class 2 |
| TON_0367 | 341772 | 342326 | - | 3238.23 | 2147.00 | 2471.72 | 0.66 | 0.76 | archaemetzincin-like protein |
| TON_0368 | 342323 | 342712 | - | 981.35 | 1041.04 | 656.97 | 1.06 | 0.67 | hypothetical protein |
| TON_0369 | 342852 | 343889 | - | 2624.89 | 4398.59 | 4506.44 | 1.68 | 1.72 | deblocking aminopeptidase |
| TON_0370 | 343965 | 345491 | - | 969.99 | 2061.65 | 1998.05 | 2.13 | 2.06 | membrane protein |
| TON_0371 | 345481 | 345882 | - | 913.20 | 1493.04 | 994.26 | 1.63 | 1.09 | Holliday junction resolvase |

| | | | | | | | | | |
|---|---|---|---|---|---|---|---|---|---|
| TON_0372 | 345954 | 346478 | + | 10792.60 | 4141.33 | 7093.28 | 0.38 | 0.66 | acetyltransferase |
| TON_0373 | 346485 | 347279 | + | 3256.41 | 3507.81 | 3650.02 | 1.08 | 1.12 | undecaprenyl diphosphate synthase |
| TON_0374 | 347380 | 348060 | + | 13799.12 | 14381.06 | 11055.66 | 1.04 | 0.80 | TBP-interacting protein |
| TON_0375 | 348135 | 348869 | + | 705.35 | 534.95 | 922.40 | 0.76 | 1.31 | hypothetical protein |
| TON_0376 | 348859 | 350742 | - | 5812.01 | 8775.54 | 9933.81 | 1.51 | 1.71 | threonyl-tRNA synthetase |
| TON_0377 | 350863 | 351450 | + | 5874.48 | 2048.43 | 5079.09 | 0.35 | 0.86 | exosome complex RNA-binding protein Csl4 |
| TON_0378 | 351453 | 352085 | + | 11484.31 | 5973.38 | 11997.86 | 0.52 | 1.04 | hypothetical protein |
| TON_0379 | 352075 | 352362 | + | 2718.03 | 1435.34 | 3021.64 | 0.53 | 1.11 | DNA-directed RNA polymerase subunit L |
| TON_0380 | 352465 | 353784 | + | 171.51 | 415.94 | 143.71 | 2.43 | 0.84 | hypothetical protein |
| TON_0381 | 353781 | 354167 | + | 540.65 | 388.29 | 450.20 | 0.72 | 0.83 | hypothetical protein |
| TON_0382 | 354162 | 354968 | - | 414.58 | 607.07 | 629.11 | 1.46 | 1.52 | AP endonuclease |
| TON_0383 | 355063 | 356070 | + | 5834.73 | 4001.89 | 6942.23 | 0.69 | 1.19 | endopeptidase IV |
| TON_0384 | 356083 | 356565 | + | 3987.88 | 2335.74 | 3435.19 | 0.59 | 0.86 | hypothetical protein |
| TON_0385 | 356555 | 357556 | + | 308.94 | 769.36 | 564.59 | 2.49 | 1.83 | membrane protein |
| TON_0386 | 357534 | 358352 | - | 444.11 | 739.31 | 1014.79 | 1.66 | 2.29 | dihydropteroate synthase |
| TON_0387 | 358349 | 358732 | - | 24.99 | 114.20 | 46.19 | 4.57 | 1.85 | membrane protein |
| TON_0388 | 358754 | 359731 | + | 1238.05 | 778.98 | 922.40 | 0.63 | 0.75 | GHMP kinase |
| TON_0389 | 359728 | 360519 | + | 328.25 | 322.17 | 329.95 | 0.98 | 1.01 | N-glycosylase/DNA lyase |
| TON_0390 | 360644 | 361510 | + | 500.90 | 894.38 | 645.98 | 1.79 | 1.29 | membrane protein |
| TON_0391 | 361516 | 362433 | + | 2119.45 | 4155.76 | 1588.18 | 1.96 | 0.75 | multidrug ABC transporter ATPase |
| TON_0392 | 362489 | 363754 | - | 3717.55 | 2889.92 | 2978.38 | 0.78 | 0.80 | 3-octaprenyl-4-hydroxybenzoate carboxy-lyase |
| TON_0393 | 363822 | 365198 | + | 3619.87 | 4862.61 | 12745.75 | 1.34 | 3.52 | DNA/RNA repair helicase |
| TON_0394 | 365169 | 365972 | - | 1919.54 | 2659.11 | 2198.23 | 1.39 | 1.15 | truA tRNA pseudouridine synthase A |
| TON_0395 | 366326 | 368044 | - | 15099.64 | 15496.64 | 20755.57 | 1.03 | 1.37 | pheT phenylalanyl-tRNA synthetase subunit beta |
| TON_0396 | 368058 | 369560 | - | 12400.92 | 8769.53 | 16616.48 | 0.71 | 1.34 | pheS phenylalanyl-tRNA synthetase subunit alpha |
| TON_0397 | 369632 | 370684 | - | 2074.01 | 2465.57 | 1651.24 | 1.19 | 0.80 | tdh L-threonine 3-dehydrogenase |
| TON_0398 | 370845 | 371588 | + | 1721.91 | 2568.95 | 2563.38 | 1.49 | 1.49 | ATPase |
| TON_0399 | 371606 | 372832 | + | 6490.10 | 16500.42 | 8818.57 | 2.54 | 1.36 | 3-hydroxy-3-methylglutaryl-CoA reductase |
| TON_0400 | 372822 | 372938 | + | 55.66 | 162.29 | 52.06 | 2.92 | 0.94 | hypothetical protein |
| TON_0401 | 373079 | 373765 | + | 1842.31 | 2131.37 | 1216.43 | 1.16 | 0.66 | hypothetical protein |
| TON_0402 | 373803 | 375695 | - | 5881.29 | 4929.93 | 8362.50 | 0.84 | 1.42 | glutamyl-tRNA(Gln) amidotransferase subunit E |
| TON_0403 | 375701 | 377020 | - | 9296.72 | 4105.27 | 12346.14 | 0.44 | 1.33 | glutamyl-tRNA(Gln) amidotransferase subunit D |

| | | | | | | | | | |
|---|---|---|---|---|---|---|---|---|---|
| TON_0404 | 377127 | 377504 | - | 1044.96 | 753.73 | 1717.23 | 0.72 | 1.64 | hypothetical protein |
| TON_0405 | 377466 | 378626 | + | 1576.52 | 973.72 | 2650.63 | 0.62 | 1.68 | putative pseudouridylate synthase |
| TON_0406 | 378720 | 379070 | + | 16077.58 | 8701.01 | 10242.50 | 0.54 | 0.64 | 50S ribosomal protein L21e |
| TON_0407 | 379073 | 379435 | + | 7873.53 | 6447.02 | 4289.40 | 0.82 | 0.54 | DNA-directed RNA polymerase subunit F |
| TON_0408 | 379505 | 380125 | + | 30242.44 | 19722.12 | 12299.95 | 0.65 | 0.41 | hypothetical protein |
| TON_0409 | 380122 | 380940 | + | 4881.77 | 5167.95 | 4172.82 | 1.06 | 0.85 | ksgA dimethyladenosine transferase |
| TON_0410 | 380974 | 381528 | - | 6327.67 | 6027.47 | 4289.40 | 0.95 | 0.68 | rubrerythrin-like protein |
| TON_0411 | 381622 | 383367 | + | 2184.19 | 1947.45 | 4289.40 | 0.89 | 1.96 | hypothetical protein |
| TON_0412 | 383357 | 384136 | - | 2732.79 | 3203.67 | 3455.72 | 1.17 | 1.26 | membrane protein |
| TON_0413 | 384182 | 386038 | - | 76480.68 | 154354.51 | 80758.05 | 2.02 | 1.06 | S-layer protein |
| TON_0414 | 386238 | 387062 | - | 11346.88 | 11909.49 | 12985.52 | 1.05 | 1.14 | hypothetical protein |
| TON_0415 | 387065 | 388111 | - | 5727.96 | 4134.12 | 5374.58 | 0.72 | 0.94 | pyruvate fromate-lyase activating enzyme-related protein |
| TON_0416 | 388137 | 388859 | - | 387.32 | 1247.81 | 1057.32 | 3.22 | 2.73 | polysaccharide deacetylase |
| TON_0417 | 388859 | 390082 | - | 1241.46 | 2460.76 | 2855.93 | 1.98 | 2.30 | glycosyltransferase |
| TON_0418 | 390058 | 390936 | - | 647.42 | 638.33 | 857.88 | 0.99 | 1.33 | hypothetical protein |
| TON_0419 | 391027 | 391962 | - | 1989.96 | 1574.79 | 2503.25 | 0.79 | 1.26 | thiamine monophosphate kinase |
| TON_0420 | 392104 | 392712 | + | 279.41 | 861.93 | 2450.46 | 3.08 | 8.77 | hypothetical protein |
| TON_0421 | 392765 | 394030 | - | 2853.19 | 805.43 | 2284.75 | 0.28 | 0.80 | phosphohydrolase |
| TON_0422 | 394096 | 394566 | + | 6342.44 | 5920.48 | 4912.65 | 0.93 | 0.77 | hypothetical protein |
| TON_0423 | 394587 | 396308 | + | 1127.87 | 2506.44 | 1248.69 | 2.22 | 1.11 | Voltage-gated chloride channel protein |
| TON_0424 | 396395 | 397735 | + | 218.08 | 1166.06 | 399.61 | 5.35 | 1.83 | nhaP type Na+/H+ and K+/H+ antiporter |
| TON_0425 | 397738 | 398601 | - | 3876.57 | 2798.56 | 5063.69 | 0.72 | 1.31 | Agmatinase |
| TON_0426 | 398677 | 399087 | - | 7524.83 | 9857.45 | 17893.77 | 1.31 | 2.38 | translation initiation factor IF-5A |
| TON_0427 | 399163 | 400542 | - | 1164.22 | 3405.63 | 1278.75 | 2.93 | 1.10 | membrane protein |
| TON_0428 | 400644 | 401303 | - | 799.62 | 1117.98 | 1079.32 | 1.40 | 1.35 | ribosome biogenesis protein |
| TON_0429 | 401281 | 402369 | - | 2294.36 | 2178.26 | 3380.19 | 0.95 | 1.47 | Saccharopine reductase |
| TON_0430 | 402429 | 403007 | + | 645.15 | 557.79 | 2109.51 | 0.86 | 3.27 | metallophosphoesterase |
| TON_0431 | 403007 | 403969 | + | 1310.74 | 1597.63 | 4355.39 | 1.22 | 3.32 | hypothetical protein |
| TON_0432 | 403938 | 404609 | - | 982.49 | 860.72 | 1635.84 | 0.88 | 1.66 | hypothetical protein |
| TON_0433 | 404606 | 405235 | - | 4678.46 | 1859.69 | 1908.60 | 0.40 | 0.41 | hypothetical protein |
| TON_0434 | 405296 | 406411 | - | 789.40 | 524.13 | 485.40 | 0.66 | 0.61 | tRNA/rRNA cytosine-C5-methylase |
| TON_0435 | 406293 | 407240 | - | 2325.03 | 5708.91 | 5047.56 | 2.46 | 2.17 | ornithine carbamoyltransferase |

| | | | | | | | | | |
|---|---|---|---|---|---|---|---|---|---|
| TON_0436 | 407475 | 407981 | + | 732.61 | 2038.81 | 582.92 | 2.78 | 0.80 | hypothetical protein |
| TON_0437 | 408067 | 408804 | + | 4068.52 | 2856.26 | 4502.77 | 0.70 | 1.11 | thyX FAD-dependent thymidylate synthase |
| TON_0438 | 408855 | 409511 | + | 281.68 | 298.13 | 897.47 | 1.06 | 3.19 | hypothetical protein |
| TON_0439 | 409669 | 411300 | + | 303.26 | 568.61 | 299.16 | 1.87 | 0.99 | hypothetical protein |
| TON_0440 | 411370 | 412101 | + | 6040.31 | 217.59 | 722.97 | 0.04 | 0.12 | hypothetical protein |
| TON_0441 | 412098 | 412601 | - | 5303.16 | 2716.81 | 3639.02 | 0.51 | 0.69 | bis(5'-adenosyl)-triphosphatase |
| TON_0442 | 412733 | 413965 | + | 14756.62 | 6634.55 | 9807.70 | 0.45 | 0.66 | cofactor-independent phosphoglycerate mutase |
| TON_0443 | 414074 | 414910 | + | 3394.98 | 3751.84 | 3487.98 | 1.11 | 1.03 | oxidoreductase |
| TON_0444 | 414910 | 416766 | + | 12800.73 | 11007.89 | 10271.10 | 0.86 | 0.80 | tungsten-containing oxidoreductase |
| TON_0445 | 417286 | 418620 | - | 1427.73 | 1737.08 | 1200.30 | 1.22 | 0.84 | hypothetical protein |
| TON_0446 | 418875 | 419600 | - | 812.11 | 2023.18 | 2072.84 | 2.49 | 2.55 | putative tRNA-binding protein |
| TON_0447 | 419527 | 420645 | - | 2980.40 | 6199.38 | 5955.30 | 2.08 | 2.00 | hypothetical protein |
| TON_0448 | 420696 | 421022 | - | 237.39 | 1343.98 | 1283.15 | 5.66 | 5.41 | hypothetical protein |
| TON_0449 | 421025 | 422011 | - | 1566.30 | 2535.29 | 4562.16 | 1.62 | 2.91 | phosphohydrolase |
| TON_0450 | 421989 | 422321 | - | 3310.93 | 2077.28 | 5303.46 | 0.63 | 1.60 | hypothetical protein |
| TON_0451 | 422351 | 422701 | - | 3795.92 | 3632.83 | 4791.66 | 0.96 | 1.26 | prefoldin subunit beta |
| TON_0452 | 422765 | 423043 | - | 196.50 | 158.68 | 472.93 | 0.81 | 2.41 | hypothetical protein |
| TON_0453 | 422986 | 423627 | - | 950.68 | 575.82 | 1901.27 | 0.61 | 2.00 | ribosomal biogenesis protein |
| TON_0454 | 423683 | 423832 | - | 3008.80 | 1890.95 | 4240.27 | 0.63 | 1.41 | DNA-directed RNA polymerase subunit P |
| TON_0455 | 423866 | 424126 | - | 6445.80 | 5420.40 | 8075.81 | 0.84 | 1.25 | rpl37ae 50S ribosomal protein L37Ae |
| TON_0456 | 424751 | 425251 | + | 1417.51 | 979.73 | 1655.64 | 0.69 | 1.17 | hypothetical protein |
| TON_0457 | 425305 | 425568 | + | 848.46 | 649.15 | 1440.07 | 0.77 | 1.70 | hypothetical protein |
| TON_0458 | 425565 | 426326 | + | 654.23 | 875.15 | 1351.34 | 1.34 | 2.07 | organic radical activating enzyme |
| TON_0459 | 426336 | 426770 | + | 73.83 | 111.80 | 156.91 | 1.51 | 2.13 | Permease |
| TON_0460 | 426893 | 427924 | + | 2440.88 | 2609.82 | 2135.90 | 1.07 | 0.88 | membrane protein |
| TON_0461 | 427921 | 428868 | + | 699.67 | 932.85 | 1110.11 | 1.33 | 1.59 | hypothetical protein |
| TON_0462 | 428937 | 430259 | + | 977.94 | 1743.09 | 1140.17 | 1.78 | 1.17 | TBP-interacting protein |
| TON_0463 | 430264 | 431268 | - | 708.75 | 1406.49 | 1163.64 | 1.98 | 1.64 | hypothetical protein |
| TON_0464 | 431252 | 431746 | - | 264.65 | 439.98 | 440.67 | 1.66 | 1.67 | hypothetical protein |
| TON_0465 | 431743 | 432144 | - | 85.19 | 109.39 | 124.65 | 1.28 | 1.46 | hypothetical protein |
| TON_0466 | 432205 | 432666 | - | 872.31 | 676.80 | 670.17 | 0.78 | 0.77 | hypothetical protein |
| TON_0467 | 433116 | 433223 | + | 679.22 | 690.02 | 498.60 | 1.02 | 0.73 | hypothetical protein |

| | | | | | | | | | |
|---|---|---|---|---|---|---|---|---|---|
| TON_0468 | 433262 | 433513 | - | 124.94 | 62.51 | 51.33 | 0.50 | 0.41 | membrane protein |
| TON_0469 | 433614 | 434078 | - | 201.04 | 292.12 | 185.51 | 1.45 | 0.92 | hypothetical protein |
| TON_0470 | 434172 | 434789 | + | 5570.08 | 5755.79 | 2497.38 | 1.03 | 0.45 | glutaredoxin-like protein |
| TON_0471 | 434852 | 435580 | + | 1548.13 | 548.17 | 349.75 | 0.35 | 0.23 | transmembrane electron transport protein |
| TON_0472 | 435572 | 435922 | - | 1791.19 | 947.28 | 907.01 | 0.53 | 0.51 | glutaredoxin/thioredoxin-like protein |
| TON_0473 | 435984 | 436556 | - | 828.02 | 1120.38 | 639.38 | 1.35 | 0.77 | hypothetical protein |
| TON_0474 | 436568 | 437383 | - | 6316.31 | 2840.63 | 1935.73 | 0.45 | 0.31 | hypothetical protein |
| TON_0475 | 437454 | 437840 | - | 2619.21 | 1226.17 | 715.63 | 0.47 | 0.27 | hypothetical protein |
| TON_0476 | 438181 | 439545 | + | 2204.63 | 389.49 | 253.70 | 0.18 | 0.12 | hydroxylamine reductase |
| TON_0477 | 439542 | 439796 | - | 245.34 | 90.16 | 65.99 | 0.37 | 0.27 | hypothetical protein |
| TON_0478 | 439757 | 439975 | - | 1110.84 | 617.89 | 244.90 | 0.56 | 0.22 | hypothetical protein |
| TON_0479 | 440156 | 441496 | + | 5547.36 | 5494.93 | 1705.50 | 0.99 | 0.31 | hypothetical protein |
| TON_0480 | 441498 | 441809 | + | 2545.38 | 1488.24 | 1401.94 | 0.58 | 0.55 | carbohydrate-binding protein |
| TON_0481 | 441810 | 442889 | + | 2353.43 | 1838.05 | 1948.19 | 0.78 | 0.83 | pepQ-1 X-pro dipeptidase |
| TON_0482 | 442886 | 443347 | - | 76.10 | 74.53 | 71.12 | 0.98 | 0.93 | transcriptional regulator |
| TON_0483 | 443467 | 444798 | + | 2218.26 | 1646.92 | 878.41 | 0.74 | 0.40 | 4-aminobutyrate aminotransferase |
| TON_0484 | 444877 | 446178 | + | 1713.96 | 2098.92 | 503.73 | 1.22 | 0.29 | xanthine/uracilpermease |
| TON_0485 | 446209 | 446793 | - | 2356.83 | 5070.58 | 1695.23 | 2.15 | 0.72 | membrane protein |
| TON_0486 | 446875 | 447573 | - | 3660.76 | 16758.87 | 8693.92 | 4.58 | 2.37 | NADH dehydrogenase subunit I |
| TON_0487 | 447579 | 448754 | - | 2044.48 | 11635.40 | 5741.20 | 5.69 | 2.81 | NADH dehydrogenase subunit D |
| TON_0488 | 448765 | 449298 | - | 1790.06 | 8777.94 | 3888.32 | 4.90 | 2.17 | NADH dehydrogenase subunit C |
| TON_0489 | 449304 | 449876 | - | 2769.14 | 13866.55 | 7462.82 | 5.01 | 2.69 | NADH dehydrogenase subunit B |
| TON_0490 | 449887 | 450816 | - | 3810.69 | 9990.89 | 7715.79 | 2.62 | 2.02 | NADH dehydrogenase subunit |
| TON_0491 | 450820 | 452682 | - | 6053.94 | 16585.77 | 8741.58 | 2.74 | 1.44 | NADH dehydrogenase subunit M |
| TON_0492 | 452679 | 454166 | - | 4204.82 | 11120.89 | 6164.27 | 2.64 | 1.47 | NADH dehydrogenase subunit N |
| TON_0493 | 454163 | 454507 | - | 404.35 | 2060.45 | 1275.09 | 5.10 | 3.15 | putative monovalent cation/H+ antiporter subunit C |
| TON_0494 | 454504 | 455214 | - | 2732.79 | 9320.10 | 6043.29 | 3.41 | 2.21 | putative monovalent cation/H+ antiporter subunit B |
| TON_0495 | 455208 | 455492 | - | 800.76 | 2153.01 | 1550.05 | 2.69 | 1.94 | hypothetical protein |
| TON_0496 | 455489 | 455899 | - | 1407.29 | 3232.52 | 2267.88 | 2.30 | 1.61 | putative monovalent cation/H+ antiporter subunit G |
| TON_0497 | 455896 | 456150 | - | 453.19 | 1579.60 | 1173.90 | 3.49 | 2.59 | putative monovalent cation/H+ antiporter subunit F |
| TON_0498 | 456147 | 456653 | - | 799.62 | 2185.47 | 1283.15 | 2.73 | 1.60 | putative monovalent cation/H+ antiporter subunit E |
| TON_0499 | 456928 | 457428 | - | 3012.20 | 3517.43 | 3301.01 | 1.17 | 1.10 | regulator of amino acid metabolism |

| | | | | | | | | | |
|---|---|---|---|---|---|---|---|---|---|
| TON_0500 | 457619 | 458878 | + | 536.11 | 424.35 | 1685.70 | 0.79 | 3.14 | molybdenum cofactor biosynthesis protein A |
| TON_0501 | 459188 | 460321 | - | 14242.09 | 25590.91 | 14001.04 | 1.80 | 0.98 | UDP-N-acetylglucosamine 2-epimerase |
| TON_0502 | 460318 | 461607 | - | 17839.24 | 25800.08 | 18207.59 | 1.45 | 1.02 | UDP-N-acetyl-D-mannosaminuronate dehydrogenase |
| TON_0503 | 461893 | 463020 | + | 18998.92 | 31527.02 | 10494.73 | 1.66 | 0.55 | hypothetical protein |
| TON_0504 | 463539 | 463817 | - | 1608.33 | 2885.11 | 2408.66 | 1.79 | 1.50 | lipoate-protein ligase A |
| TON_0505 | 463887 | 465815 | + | 12310.06 | 4934.74 | 7316.18 | 0.40 | 0.59 | argS arginyl-tRNA synthetase |
| TON_0506 | 465860 | 466741 | + | 512.26 | 1054.27 | 647.44 | 2.06 | 1.26 | dihydrodipicolinate synthase |
| TON_0507 | 466735 | 467982 | - | 15411.99 | 8200.92 | 17579.21 | 0.53 | 1.14 | peptide chain release factor 1 |
| TON_0508 | 468080 | 468316 | - | 1882.06 | 2478.79 | 2149.10 | 1.32 | 1.14 | membrane protein |
| TON_0509 | 468326 | 468805 | - | 4059.43 | 3880.47 | 1555.18 | 0.96 | 0.38 | hypothetical protein |
| TON_0510 | 469262 | 469438 | - | 29768.80 | 51500.39 | 41266.97 | 1.73 | 1.39 | hypothetical protein |
| TON_0511 | 469556 | 470119 | + | 531.57 | 1404.09 | 1009.66 | 2.64 | 1.90 | hypothetical protein |
| TON_0512 | 470109 | 471359 | - | 4069.66 | 5505.75 | 4263.00 | 1.35 | 1.05 | membrane-associated metallopeptidase |
| TON_0513 | 471403 | 471795 | - | 2586.27 | 3622.01 | 4216.81 | 1.40 | 1.63 | hypothetical protein |
| TON_0514 | 471805 | 472965 | - | 4254.80 | 4318.05 | 5356.98 | 1.01 | 1.26 | hypothetical protein |
| TON_0515 | 473544 | 474248 | + | 785.99 | 294.52 | 92.39 | 0.37 | 0.12 | hypothetical protein |
| TON_0516 | 474692 | 475237 | + | 5006.71 | 5877.21 | 3842.13 | 1.17 | 0.77 | competence-like protein |
| TON_0517 | 475321 | 475776 | + | 24260.06 | 7849.90 | 30167.32 | 0.32 | 1.24 | rps15p 30S ribosomal protein S15 |
| TON_0518 | 475806 | 477236 | + | 21579.51 | 5475.70 | 32446.93 | 0.25 | 1.50 | ssDNA-specific exonuclease |
| TON_0519 | 477226 | 477477 | + | 8401.69 | 2968.06 | 10765.30 | 0.35 | 1.28 | hypothetical protein |
| TON_0520 | 477496 | 478098 | + | 10259.89 | 5313.41 | 17482.42 | 0.52 | 1.70 | 30S ribosomal protein S3Ae |
| TON_0521 | 478189 | 479244 | + | 9739.69 | 3940.58 | 8756.24 | 0.40 | 0.90 | RNA-binding protein |
| TON_0522 | 479372 | 480085 | - | 2456.79 | 1484.63 | 2916.06 | 0.60 | 1.19 | membrane protein |
| TON_0523 | 480163 | 480612 | - | 4266.15 | 1353.60 | 1333.01 | 0.32 | 0.31 | putative transcriptional regulator |
| TON_0524 | 480784 | 481086 | - | 4753.42 | 5510.56 | 5238.93 | 1.16 | 1.10 | nucleotide pyrophosphohydrolase |
| TON_0525 | 481092 | 481469 | - | 4116.22 | 5068.17 | 4512.30 | 1.23 | 1.10 | transcriptional regulator |
| TON_0526 | 481526 | 481897 | - | 241.93 | 306.54 | 310.16 | 1.27 | 1.28 | metalloprotease |
| TON_0527 | 481929 | 482312 | - | 1300.52 | 1426.93 | 1637.30 | 1.10 | 1.26 | hypothetical protein |
| TON_0528 | 482389 | 483192 | - | 3527.87 | 3732.61 | 3204.95 | 1.06 | 0.91 | hypothetical protein |
| TON_0529 | 483311 | 485218 | + | 23108.33 | 21611.87 | 24091.77 | 0.94 | 1.04 | ATP-dependent protease Lon |
| TON_0530 | 485334 | 486080 | + | 12588.33 | 8598.83 | 7496.55 | 0.68 | 0.60 | Fe-S cluster assembly ABC transporter ATPase |
| TON_0531 | 486073 | 487410 | + | 21679.46 | 14099.77 | 11254.36 | 0.65 | 0.52 | Fe-S cluster assembly ABC transporter permease |

| | | | | | | | | | |
|---|---|---|---|---|---|---|---|---|---|
| TON_0532 | 487523 | 487816 | + | 188.55 | 240.43 | 151.78 | 1.28 | 0.80 | hypothetical protein |
| TON_0533 | 487831 | 488280 | - | 2031.99 | 290.92 | 396.68 | 0.14 | 0.20 | hydrogenase-specific maturation endopeptidase |
| TON_0534 | 488328 | 489617 | - | 10363.25 | 224.80 | 170.84 | 0.02 | 0.02 | cytosolic NiFe-hydrogenase subunit alpha |
| TON_0535 | 489614 | 490411 | - | 20711.74 | 311.35 | 318.22 | 0.02 | 0.02 | cytosolic NiFe-hydrogenase subunit delta |
| TON_0536 | 490422 | 491297 | - | 9345.56 | 134.64 | 144.45 | 0.01 | 0.02 | cytochrome-c3 hydrogenase subunit gamma |
| TON_0537 | 491294 | 492397 | - | 22357.55 | 113.00 | 156.18 | 0.01 | 0.01 | sulfhydrogenase subunit beta |
| TON_0538 | 492769 | 493758 | + | 16819.28 | 3720.59 | 2895.53 | 0.22 | 0.17 | formate transporter |
| TON_0539 | 493791 | 495833 | + | 72289.49 | 27798.02 | 15914.04 | 0.38 | 0.22 | formate dehydrogenase subunit alpha |
| TON_0540 | 495836 | 496327 | + | 26006.95 | 16228.74 | 6717.86 | 0.62 | 0.26 | Oxidoreductase iron-sulfur protein |
| TON_0541 | 496333 | 496710 | + | 11944.32 | 8406.49 | 5165.61 | 0.70 | 0.43 | 4Fe-4S binding protein |
| TON_0542 | 496716 | 497774 | + | 28832.88 | 22477.40 | 13127.03 | 0.78 | 0.46 | putative glutamate synthase subunit beta |
| TON_0543 | 497780 | 498286 | + | 6311.77 | 9005.15 | 4105.36 | 1.43 | 0.65 | 4Fe-4S binding protein |
| TON_0544 | 498574 | 499794 | + | 24171.46 | 91048.02 | 63473.60 | 3.77 | 2.63 | alcohol dehydrogenase |
| TON_0545 | 500077 | 500250 | - | 270.33 | 126.22 | 53.53 | 0.47 | 0.20 | membrane protein |
| TON_0546 | 500315 | 502189 | - | 22845.95 | 29500.24 | 20210.04 | 1.29 | 0.88 | putative vitamin B12 transport protein |
| TON_0547 | 502293 | 503480 | - | 1865.02 | 2147.00 | 2489.32 | 1.15 | 1.33 | hypothetical protein |
| TON_0548 | 503598 | 504170 | - | 726.93 | 522.93 | 788.22 | 0.72 | 1.08 | putative transcriptional regulator |
| TON_0549 | 504458 | 505327 | - | 586.08 | 1624.08 | 557.26 | 2.77 | 0.95 | permease |
| TON_0550 | 505386 | 506213 | - | 224.89 | 456.81 | 107.05 | 2.03 | 0.48 | ubiA prenyltransferase |
| TON_0551 | 506333 | 507127 | - | 4252.52 | 10433.27 | 4565.83 | 2.45 | 1.07 | hypothetical protein |
| TON_0552 | 507269 | 508009 | + | 357.78 | 426.76 | 364.42 | 1.19 | 1.02 | hypothetical protein |
| TON_0553 | 508118 | 509824 | - | 3931.09 | 2680.75 | 7770.78 | 0.68 | 1.98 | hypothetical protein |
| TON_0554 | 509938 | 511401 | + | 1390.25 | 1383.65 | 908.47 | 1.00 | 0.65 | phosphoesterase |
| TON_0555 | 511404 | 511613 | - | 1210.79 | 2767.30 | 2010.52 | 2.29 | 1.66 | hypothetical protein |
| TON_0556 | 511655 | 512179 | - | 354.38 | 675.60 | 841.02 | 1.91 | 2.37 | hypothetical protein |
| TON_0557 | 512176 | 512811 | - | 2682.82 | 2243.17 | 3872.93 | 0.84 | 1.44 | putative transcriptional regulator |
| TON_0558 | 512808 | 513377 | - | 1915.00 | 1023.01 | 1558.12 | 0.53 | 0.81 | endonuclease V |
| TON_0559 | 513408 | 514025 | - | 1098.34 | 490.47 | 668.71 | 0.45 | 0.61 | haloacid dehalogenase superfamily protein |
| TON_0560 | 514360 | 515577 | + | 8431.22 | 4957.58 | 4207.28 | 0.59 | 0.50 | S-adenosylmethionine synthetase |
| TON_0561 | 515623 | 515898 | - | 1102.88 | 5915.67 | 15479.97 | 5.36 | 14.04 | hypothetical protein |
| TON_0562 | 515978 | 516520 | + | 2915.66 | 4152.15 | 2910.93 | 1.42 | 1.00 | putative kinase |
| TON_0563 | 516523 | 517380 | + | 2005.86 | 3064.23 | 2877.93 | 1.53 | 1.43 | zinc-dependent protease |

| | | | | | | | | | |
|---|---|---|---|---|---|---|---|---|---|
| TON_0564 | 517395 | 518045 | + | 1056.32 | 1800.79 | 1885.87 | 1.70 | 1.79 | metallophosphoesterase |
| TON_0565 | 518042 | 519211 | - | 5943.76 | 7203.15 | 6449.50 | 1.21 | 1.09 | aspartate aminotransferase |
| TON_0566 | 519291 | 520895 | - | 1584.47 | 1339.17 | 1164.37 | 0.85 | 0.73 | carbamoyl transferase |
| TON_0567 | 520987 | 521373 | + | 949.55 | 1900.57 | 1049.25 | 2.00 | 1.11 | pyrolysin |
| TON_0568 | 521451 | 522899 | - | 9520.47 | 5776.23 | 20505.54 | 0.61 | 2.15 | prolyl-tRNA synthetase |
| TON_0569 | 523025 | 524026 | + | 1974.06 | 4094.45 | 2110.24 | 2.07 | 1.07 | D-isomer specific 2-hydroxyacid dehydrogenase |
| TON_0570 | 524037 | 525032 | + | 1874.11 | 2809.37 | 2199.69 | 1.50 | 1.17 | endoglucanase |
| TON_0571 | 525061 | 525825 | + | 1123.33 | 1298.30 | 1276.56 | 1.16 | 1.14 | membrane protein |
| TON_0572 | 525810 | 526328 | - | 30.67 | 58.90 | 20.53 | 1.92 | 0.67 | membrane protein |
| TON_0573 | 526374 | 527105 | + | 540.65 | 552.98 | 594.65 | 1.02 | 1.10 | membrane protein |
| TON_0574 | 527152 | 527373 | + | 357.78 | 278.89 | 264.70 | 0.78 | 0.74 | hypothetical protein |
| TON_0575 | 527497 | 528699 | + | 3240.50 | 1725.05 | 1389.47 | 0.53 | 0.43 | hypothetical protein |
| TON_0576 | 528737 | 529585 | + | 236.25 | 211.57 | 123.18 | 0.90 | 0.52 | membrane protein |
| TON_0577 | 529740 | 530981 | + | 132727.80 | 51535.25 | 22735.29 | 0.39 | 0.17 | hypothetical protein |
| TON_0578 | 531042 | 531995 | + | 11483.18 | 3279.41 | 3726.28 | 0.29 | 0.32 | membrane protein |
| TON_0579 | 532003 | 534204 | - | 14298.88 | 7367.85 | 13734.15 | 0.52 | 0.96 | metG methionyl-tRNA synthetase |
| TON_0580 | 534384 | 534731 | + | 845.05 | 409.93 | 282.29 | 0.49 | 0.33 | 6-pyruvoyl-tetrahydropterin synthase |
| TON_0581 | 534728 | 535198 | - | 489.54 | 304.14 | 541.12 | 0.62 | 1.11 | hypothetical protein |
| TON_0582 | 535259 | 535819 | - | 770.09 | 904.00 | 345.35 | 1.17 | 0.45 | 2-oxoglutarate ferredoxin oxidoreductase subunit gamma |
| TON_0583 | 535816 | 536661 | - | 971.13 | 1668.55 | 576.32 | 1.72 | 0.59 | 2-oxoglutarate ferredoxin oxidoreductase subunit beta |
| TON_0584 | 536662 | 537810 | - | 3375.67 | 3216.90 | 1332.28 | 0.95 | 0.39 | 2-oxoglutarate ferredoxin oxidoreductase subunit alpha |
| TON_0585 | 537807 | 538319 | - | 1870.70 | 1538.72 | 439.94 | 0.82 | 0.24 | 2-oxoglutarate ferredoxin oxidoreductase subunit gamma |
| TON_0586 | 538325 | 539179 | - | 2305.72 | 2108.53 | 662.84 | 0.91 | 0.29 | 2-oxoglutarate ferredoxin oxidoreductase subunit beta |
| TON_0587 | 539179 | 540369 | - | 1640.13 | 1929.42 | 529.39 | 1.18 | 0.32 | 2-oxoglutarate ferredoxin oxidoreductase subunit alpha |
| TON_0588 | 540373 | 540645 | - | 1556.08 | 3044.99 | 435.54 | 1.96 | 0.28 | 2-oxoacid:ferredoxin oxidoreductase subunit delta |
| TON_0589 | 540796 | 541767 | - | 2130.80 | 1787.57 | 3405.86 | 0.84 | 1.60 | hypothetical protein |
| TON_0590 | 541829 | 542611 | + | 3025.83 | 2239.57 | 4162.55 | 0.74 | 1.38 | surE stationary phase survival protein SurE |
| TON_0591 | 542670 | 543167 | + | 37.48 | 105.79 | 81.39 | 2.82 | 2.17 | membrane protein |
| TON_0592 | 543199 | 544104 | - | 3073.54 | 5591.10 | 4434.58 | 1.82 | 1.44 | signal recognition particle GTPase |
| TON_0593 | 544245 | 544694 | - | 670.14 | 1560.36 | 783.09 | 2.33 | 1.17 | hypothetical protein |
| TON_0594 | 544818 | 546041 | + | 58137.13 | 200147.20 | 131422.11 | 3.44 | 2.26 | sugar binding protein |
| TON_0595 | 546088 | 547614 | + | 11070.87 | 39794.06 | 32285.62 | 3.59 | 2.92 | ABC transporter ATPase |

| | | | | | | | | | |
|---|---|---|---|---|---|---|---|---|---|
| TON_0596 | 547607 | 548641 | + | 3755.03 | 23293.65 | 11018.26 | 6.20 | 2.93 | ABC transporter permease |
| TON_0597 | 548638 | 549534 | + | 1755.98 | 9185.46 | 4572.43 | 5.23 | 2.60 | ABC transporter permease |
| TON_0598 | 549584 | 550567 | - | 655.37 | 1075.91 | 404.74 | 1.64 | 0.62 | ABC-type manganese/zinc transport system periplasmic protein |
| TON_0599 | 550659 | 552449 | + | 1155.13 | 1798.38 | 1441.53 | 1.56 | 1.25 | hypothetical protein |
| TON_0600 | 552428 | 552763 | - | 23519.50 | 11257.93 | 24076.37 | 0.48 | 1.02 | hypothetical protein |
| TON_0601 | 552776 | 553423 | - | 24925.65 | 15370.42 | 33125.91 | 0.62 | 1.33 | hypoxanthine/guanine phosphoribosyltransferase |
| TON_0602 | 553515 | 554945 | - | 12430.45 | 9166.23 | 10491.07 | 0.74 | 0.84 | cysS cysteinyl-tRNA synthetase |
| TON_0603 | 555062 | 556111 | + | 14686.20 | 15536.31 | 22001.33 | 1.06 | 1.50 | FKBP-type peptidyl-prolyl cis-trans isomerase |
| TON_0604 | 556149 | 556751 | + | 84.05 | 87.76 | 115.85 | 1.04 | 1.38 | membrane-associated metalloprotease |
| TON_0605 | 556787 | 557539 | + | 170.37 | 138.24 | 335.09 | 0.81 | 1.97 | hypothetical protein |
| TON_0606 | 557499 | 559424 | - | 3932.22 | 3970.63 | 3534.91 | 1.01 | 0.90 | ABC-type transporter, ATPase component |
| TON_0607 | 559928 | 560386 | - | 594.04 | 502.49 | 335.09 | 0.85 | 0.56 | ribH 6,7-dimethyl-8-ribityllumazine synthase |
| TON_0608 | 560452 | 561618 | - | 931.38 | 698.44 | 1001.59 | 0.75 | 1.08 | bifunctional 3,4-dihydroxy-2-butanone 4-phosphate synthase/GTP cyclohydrolase II protein |
| TON_0609 | 561615 | 562172 | - | 137.43 | 175.51 | 244.17 | 1.28 | 1.78 | riboflavin synthase subunit alpha |
| TON_0610 | 562153 | 563238 | - | 141.98 | 307.74 | 229.50 | 2.17 | 1.62 | riboflavin biosynthesis protein RibD |
| TON_0611 | 563339 | 565189 | + | 2463.60 | 5000.85 | 2894.80 | 2.03 | 1.18 | prolyl endopeptidase |
| TON_0612 | 565310 | 566014 | + | 6181.15 | 6667.01 | 5431.77 | 1.08 | 0.88 | ATPase |
| TON_0613 | 566006 | 570175 | - | 6574.15 | 11338.48 | 13324.27 | 1.72 | 2.03 | large helicase-like protein |
| TON_0614 | 570267 | 570542 | + | 362.33 | 1240.60 | 738.36 | 3.42 | 2.04 | hypothetical protein |
| TON_0615 | 570567 | 571616 | + | 959.77 | 2222.74 | 1355.74 | 2.32 | 1.41 | glycosyltransferase |
| TON_0616 | 571725 | 572642 | + | 70.42 | 1627.68 | 5085.69 | 23.11 | 72.22 | sodium/phosphate symporter |
| TON_0617 | 572684 | 574426 | + | 1723.04 | 3278.20 | 6659.20 | 1.90 | 3.86 | 7-cyano-7-deazaguanine tRNA-ribosyltransferase |
| TON_0618 | 574828 | 577053 | - | 3112.16 | 3204.87 | 1617.51 | 1.03 | 0.52 | hypothetical protein |
| TON_0619 | 577168 | 578313 | - | 3908.37 | 3215.69 | 3691.08 | 0.82 | 0.94 | hypothetical protein |
| TON_0620 | 578484 | 579212 | + | 1099.48 | 2119.35 | 802.89 | 1.93 | 0.73 | chromosome partitioning protein |
| TON_0621 | 579317 | 579970 | + | 922.29 | 1077.11 | 940.74 | 1.17 | 1.02 | phosphoglycolate phosphatase |
| TON_0622 | 580158 | 581144 | + | 3726.64 | 2928.39 | 3164.62 | 0.79 | 0.85 | membrane protein |
| TON_0623 | 581147 | 582040 | + | 806.43 | 1230.98 | 1325.68 | 1.53 | 1.64 | hypothetical protein |
| TON_0624 | 582052 | 582546 | + | 397.54 | 1609.65 | 1380.67 | 4.05 | 3.47 | hypothetical protein |
| TON_0625 | 582548 | 583531 | + | 424.80 | 1293.49 | 1093.25 | 3.04 | 2.57 | hypothetical protein |
| TON_0626 | 583575 | 584633 | - | 79.51 | 119.01 | 171.58 | 1.50 | 2.16 | protein MJECL37 |

| | | | | | | | | | |
|---|---|---|---|---|---|---|---|---|---|
| TON_0627 | 584768 | 585790 | - | 882.53 | 567.40 | 540.39 | 0.64 | 0.61 | deblocking aminopeptidase |
| TON_0628 | 585844 | 586728 | - | 159.02 | 234.42 | 104.12 | 1.47 | 0.65 | permease |
| TON_0629 | 586852 | 587256 | + | 213.53 | 128.63 | 68.92 | 0.60 | 0.32 | transcriptional regulator |
| TON_0630 | 587253 | 588584 | + | 1266.44 | 1206.94 | 471.47 | 0.95 | 0.37 | membrane protein |
| TON_0631 | 588679 | 589359 | + | 491.81 | 442.38 | 294.76 | 0.90 | 0.60 | hypothetical protein |
| TON_0632 | 589349 | 589627 | + | 243.07 | 245.23 | 169.38 | 1.01 | 0.70 | hypothetical protein |
| TON_0633 | 589701 | 590006 | + | 74.96 | 198.35 | 81.39 | 2.65 | 1.09 | hypothetical protein |
| TON_0634 | 590079 | 591314 | + | 85.19 | 114.20 | 65.26 | 1.34 | 0.77 | permease |
| TON_0635 | 591326 | 591841 | - | 550.87 | 839.09 | 852.75 | 1.52 | 1.55 | cobalamin adenosyltransferase |
| TON_0636 | 591980 | 594619 | + | 7505.52 | 12532.19 | 16081.95 | 1.67 | 2.14 | hypothetical protein |
| TON_0637 | 594677 | 595504 | - | 1543.58 | 697.23 | 1212.76 | 0.45 | 0.79 | translation initiation factor IF-2 |
| TON_0638 | 595588 | 596346 | - | 881.40 | 797.01 | 990.59 | 0.90 | 1.12 | competence damage-inducible protein A |
| TON_0639 | 596501 | 597505 | + | 595.17 | 4229.09 | 466.33 | 7.11 | 0.78 | glyceraldehyde-3-phosphate dehydrogenase |
| TON_0640 | 597575 | 598681 | - | 1510.65 | 1387.26 | 755.23 | 0.92 | 0.50 | aromatic amino acid permease |
| TON_0641 | 598786 | 599070 | - | 2952.01 | 3630.43 | 9177.12 | 1.23 | 3.11 | hypothetical protein |
| TON_0642 | 599067 | 599573 | - | 3098.53 | 4154.56 | 9902.28 | 1.34 | 3.20 | hypothetical protein |
| TON_0643 | 599611 | 601314 | - | 9695.39 | 6201.78 | 24151.89 | 0.64 | 2.49 | glycyl-tRNA synthetase |
| TON_0644 | 601464 | 601700 | + | 20092.72 | 15342.77 | 15760.07 | 0.76 | 0.78 | small nuclear ribonucleoprotein |
| TON_0645 | 601725 | 601913 | + | 8779.92 | 3428.47 | 5485.30 | 0.39 | 0.62 | rpl37e 50S ribosomal protein L37e |
| TON_0646 | 602029 | 603396 | + | 442.97 | 195.95 | 260.30 | 0.44 | 0.59 | sodium-driven multidrug efflux pump protein |
| TON_0647 | 603427 | 604521 | - | 1635.59 | 2005.15 | 1368.94 | 1.23 | 0.84 | hypothetical protein |
| TON_0648 | 604606 | 605739 | - | 2376.14 | 834.28 | 1417.34 | 0.35 | 0.60 | N(2),N(2)-dimethylguanosine tRNA methyltransferase |
| TON_0649 | 605772 | 606038 | - | 637.20 | 162.29 | 221.44 | 0.25 | 0.35 | 50S ribosomal protein L35 |
| TON_0650 | 606087 | 606743 | - | 3625.55 | 3362.35 | 2954.92 | 0.93 | 0.82 | hydrolase |
| TON_0651 | 606831 | 607877 | + | 3933.36 | 4833.76 | 4103.89 | 1.23 | 1.04 | Xaa-Pro aminopeptidase |
| TON_0652 | 607942 | 609015 | + | 3705.06 | 3811.95 | 4170.62 | 1.03 | 1.13 | cell division protein |
| TON_0653 | 609019 | 610017 | - | 1753.71 | 1745.49 | 2094.84 | 1.00 | 1.19 | HypE protein |
| TON_0654 | 610014 | 610808 | - | 991.57 | 711.66 | 1279.49 | 0.72 | 1.29 | ATPase |
| TON_0655 | 610846 | 611244 | - | 260.10 | 260.86 | 523.53 | 1.00 | 2.01 | hypothetical protein |
| TON_0656 | 611467 | 611694 | + | 387.32 | 1156.45 | 210.44 | 2.99 | 0.54 | iron(II) transport protein A |
| TON_0657 | 611706 | 613682 | + | 3382.48 | 3805.94 | 1295.62 | 1.13 | 0.38 | iron(II) transport protein B |
| TON_0658 | 613693 | 613947 | + | 61.33 | 163.49 | 53.53 | 2.67 | 0.87 | LexA-related DNA-binding protein |

| | | | | | | | | | |
|---|---|---|---|---|---|---|---|---|---|
| TON_0659 | 614035 | 615276 | + | 10589.28 | 159855.45 | 18393.83 | 15.10 | 1.74 | sugar-phosphate nucleotidyltransferase |
| TON_0660 | 615428 | 616231 | - | 1340.27 | 1710.63 | 2501.78 | 1.28 | 1.87 | hypothetical protein |
| TON_0661 | 616332 | 616817 | + | 1206.24 | 552.98 | 1106.45 | 0.46 | 0.92 | aromatic acid decarboxylase |
| TON_0662 | 616866 | 617348 | + | 9938.46 | 8002.57 | 5653.21 | 0.81 | 0.57 | putative transcriptional regulator |
| TON_0663 | 617349 | 617870 | + | 3041.74 | 4392.58 | 2753.28 | 1.44 | 0.91 | adenylate cyclase |
| TON_0664 | 617950 | 618345 | + | 808.71 | 1066.29 | 645.24 | 1.32 | 0.80 | hypothetical protein |
| TON_0665 | 618303 | 619295 | + | 775.77 | 830.67 | 475.87 | 1.07 | 0.61 | TM1410 -related protein |
| TON_0666 | 619282 | 620475 | - | 4304.77 | 3398.42 | 3911.05 | 0.79 | 0.91 | translation-associated GTPase |
| TON_0667 | 620716 | 620910 | + | 244.20 | 74.53 | 103.39 | 0.31 | 0.42 | Metal-dependent hydrolase |
| TON_0668 | 620964 | 621083 | + | 30.67 | 7.21 | 21.26 | 0.24 | 0.69 | magnesium and cobalt transporter |
| TON_0669 | 621078 | 621767 | - | 5847.22 | 1523.10 | 2052.31 | 0.26 | 0.35 | aspartate racemase |
| TON_0670 | 621770 | 622114 | - | 4836.34 | 974.93 | 1365.28 | 0.20 | 0.28 | hypothetical protein |
| TON_0671 | 622462 | 623829 | + | 3998.10 | 2928.39 | 2817.07 | 0.73 | 0.70 | zinc-dependent protease |
| TON_0672 | 623840 | 625165 | + | 3044.01 | 3665.29 | 2334.61 | 1.20 | 0.77 | zinc-dependent protease |
| TON_0673 | 625249 | 626244 | - | 2225.08 | 1728.66 | 4586.36 | 0.78 | 2.06 | Met-10+ like protein |
| TON_0674 | 626783 | 627535 | + | 16282.03 | 15759.91 | 14370.59 | 0.97 | 0.88 | hypothetical protein |
| TON_0675 | 627600 | 628757 | + | 4571.69 | 2815.39 | 9136.79 | 0.62 | 2.00 | tRNA m1G methyltransferase |
| TON_0676 | 628774 | 629355 | - | 10412.09 | 9120.55 | 5870.98 | 0.88 | 0.56 | hypothetical protein |
| TON_0677 | 629512 | 632166 | - | 8892.36 | 8851.27 | 10122.25 | 1.00 | 1.14 | valS valyl-tRNA synthetase |
| TON_0678 | 633150 | 634112 | - | 1188.07 | 1497.85 | 1580.11 | 1.26 | 1.33 | putative aminotransferase |
| TON_0679 | 634097 | 634981 | - | 1351.63 | 2376.61 | 1743.62 | 1.76 | 1.29 | cobD cobalamin biosynthesis protein |
| TON_0680 | 634969 | 635715 | - | 473.64 | 619.10 | 657.71 | 1.31 | 1.39 | uracil-DNA glycosylase |
| TON_0681 | 635758 | 636006 | + | 1489.06 | 2415.08 | 1156.31 | 1.62 | 0.78 | hypothetical protein |
| TON_0682 | 636003 | 636644 | + | 2253.47 | 2032.80 | 1556.65 | 0.90 | 0.69 | ATPase |
| TON_0683 | 636641 | 637051 | + | 621.30 | 711.66 | 395.94 | 1.15 | 0.64 | hypothetical protein |
| TON_0684 | 637055 | 637756 | + | 164.69 | 382.28 | 185.51 | 2.32 | 1.13 | cobS cobalamin synthase |
| TON_0685 | 637770 | 638885 | - | 1944.53 | 1279.06 | 2771.61 | 0.66 | 1.43 | hypothetical protein |
| TON_0686 | 638945 | 639490 | + | 89.73 | 27.65 | 55.73 | 0.31 | 0.62 | GTP:adenosylcobinamide-phosphate guanylyltransferase |
| TON_0687 | 639465 | 640919 | - | 2137.62 | 698.44 | 1049.25 | 0.33 | 0.49 | cobyric acid synthase |
| TON_0688 | 640988 | 641995 | + | 3692.56 | 3194.06 | 4092.89 | 0.86 | 1.11 | hypothetical protein |
| TON_0689 | 641992 | 642579 | - | 2354.56 | 985.75 | 1023.59 | 0.42 | 0.43 | adenosylcobinamide amidohydrolase |
| TON_0690 | 642692 | 643207 | + | 1300.52 | 2778.12 | 2003.92 | 2.14 | 1.54 | molybdenum cofactor biosynthesis protein B |

| | | | | | | | | | |
|---|---|---|---|---|---|---|---|---|---|
| TON_0691 | 643210 | 644400 | + | 9783.98 | 18581.30 | 13938.72 | 1.90 | 1.42 | molybdenum cofactor biosynthesis protein MoeA |
| TON_0692 | 644476 | 644697 | - | 7872.39 | 3917.74 | 3420.52 | 0.50 | 0.43 | hypothetical protein |
| TON_0693 | 645060 | 646199 | - | 3682.34 | 16835.81 | 12461.26 | 4.57 | 3.38 | membrane protein |
| TON_0694 | 646160 | 647218 | - | 10583.60 | 15009.78 | 32235.76 | 1.42 | 3.05 | hypothetical protein |
| TON_0695 | 647359 | 648642 | + | 2430.66 | 2139.79 | 1055.85 | 0.88 | 0.43 | coenzyme F390 synthetase I |
| TON_0696 | 648725 | 649975 | + | 16400.16 | 7571.01 | 8549.47 | 0.46 | 0.52 | metal-dependent phosphohydrolase |
| TON_0697 | 649968 | 650927 | - | 7554.36 | 6746.35 | 6288.19 | 0.89 | 0.83 | hypothetical protein |
| TON_0698 | 650970 | 651503 | + | 1035.87 | 1416.11 | 706.10 | 1.37 | 0.68 | N-acetyltransferase |
| TON_0699 | 651536 | 652819 | + | 1405.01 | 1299.50 | 2366.14 | 0.92 | 1.68 | nucleic acid-binding protein |
| TON_0700 | 652816 | 653988 | - | 1921.81 | 665.98 | 691.44 | 0.35 | 0.36 | ATPase |
| TON_0701 | 654076 | 655083 | - | 6110.73 | 3686.93 | 5269.00 | 0.60 | 0.86 | putative deoxyhypusine synthase |
| TON_0702 | 655119 | 656165 | - | 436.16 | 651.55 | 145.18 | 1.49 | 0.33 | putative glutamate synthase subunit beta |
| TON_0703 | 656336 | 657142 | + | 3406.33 | 3079.85 | 2370.54 | 0.90 | 0.70 | glucose-1-dehydrogenase |
| TON_0704 | 657511 | 658653 | - | 1288.02 | 1519.49 | 879.88 | 1.18 | 0.68 | Imidazolonepropionase-like amidohydrolase |
| TON_0705 | 658792 | 659664 | - | 3525.60 | 9817.78 | 11884.94 | 2.78 | 3.37 | heat shock protein HtpX |
| TON_0706 | 659703 | 660263 | - | 1211.92 | 1578.39 | 10481.54 | 1.30 | 8.65 | hypothetical protein |
| TON_0707 | 660336 | 661988 | - | 7663.40 | 14800.61 | 55554.71 | 1.93 | 7.25 | chaperonin subunit alpha |
| TON_0708 | 662165 | 662476 | + | 895.03 | 566.20 | 426.74 | 0.63 | 0.48 | hypothetical protein |
| TON_0709 | 662483 | 663145 | + | 2586.27 | 1151.64 | 1803.75 | 0.45 | 0.70 | serine/threonine protein kinase |
| TON_0710 | 663203 | 664462 | - | 700.80 | 670.79 | 673.84 | 0.96 | 0.96 | methyl-accepting chemotaxis protein |
| TON_0711 | 664576 | 666306 | - | 712.16 | 946.08 | 1056.59 | 1.33 | 1.48 | MutS-like DNA mismatch repair ATPase |
| TON_0712 | 666449 | 666955 | + | 339.61 | 812.64 | 645.24 | 2.39 | 1.90 | hypothetical protein |
| TON_0713 | 666969 | 667439 | - | 2501.08 | 3635.24 | 2289.88 | 1.45 | 0.92 | putative molybdenum cofactor biosynthesis protein MoaC |
| TON_0714 | 667471 | 668910 | - | 8974.14 | 10197.66 | 7520.02 | 1.14 | 0.84 | RNA terminal phosphate cyclase |
| TON_0715 | 668970 | 669416 | - | 926.83 | 671.99 | 778.69 | 0.73 | 0.84 | hypothetical protein |
| TON_0716 | 669413 | 670351 | - | 1983.15 | 1685.38 | 1697.43 | 0.85 | 0.86 | tRNA/rRNA cytosine-C5-methylase |
| TON_0717 | 670361 | 671254 | - | 934.78 | 722.48 | 684.10 | 0.77 | 0.73 | hypothetical protein |
| TON_0718 | 670847 | 671704 | + | 2522.66 | 1325.95 | 1849.94 | 0.53 | 0.73 | panB 3-methyl-2-oxobutanoate hydroxymethyltransferase |
| TON_0719 | 671705 | 672370 | + | 4263.88 | 2481.19 | 3556.90 | 0.58 | 0.83 | dolichol-phosphate mannosyltransferase |
| TON_0720 | 672360 | 673202 | - | 247.61 | 274.09 | 250.76 | 1.11 | 1.01 | hypothetical protein |
| TON_0721 | 673454 | 673879 | + | 2502.22 | 4213.46 | 2113.17 | 1.68 | 0.84 | hypothetical protein |
| TON_0722 | 674212 | 675027 | - | 272.60 | 357.03 | 283.76 | 1.31 | 1.04 | membrane-bound phosphoesterase |

| | | | | | | | | | |
|---|---|---|---|---|---|---|---|---|---|
| TON_0723 | 675166 | 675612 | + | 2116.04 | 774.17 | 1283.89 | 0.37 | 0.61 | hypothetical protein |
| TON_0724 | 675616 | 676233 | + | 1227.83 | 501.29 | 959.80 | 0.41 | 0.78 | hypothetical protein |
| TON_0725 | 676288 | 678963 | - | 1066.54 | 1922.20 | 1520.72 | 1.80 | 1.43 | DEAD/DEAH box helicase |
| TON_0726 | 679111 | 679326 | + | 48.84 | 36.06 | 41.79 | 0.74 | 0.86 | hypothetical protein |
| TON_0727 | 679421 | 681196 | + | 12073.80 | 9379.01 | 9796.70 | 0.78 | 0.81 | putative ATPase RIL |
| TON_0728 | 681403 | 682110 | + | 1029.06 | 3125.53 | 1841.88 | 3.04 | 1.79 | hypothetical protein |
| TON_0729 | 682110 | 682334 | + | 40.89 | 228.40 | 84.32 | 5.59 | 2.06 | hypothetical protein |
| TON_0730 | 682560 | 683744 | + | 25550.35 | 6844.92 | 4151.55 | 0.27 | 0.16 | hypothetical protein |
| TON_0731 | 683974 | 684804 | + | 5140.74 | 3125.53 | 1262.62 | 0.61 | 0.25 | hypothetical protein |
| TON_0732 | 684858 | 685160 | + | 2504.49 | 848.70 | 475.87 | 0.34 | 0.19 | hypothetical protein |
| TON_0733 | 685163 | 685348 | + | 683.77 | 349.82 | 178.18 | 0.51 | 0.26 | hypothetical protein |
| TON_0734 | 685424 | 685720 | + | 881.40 | 1382.45 | 1213.50 | 1.57 | 1.38 | Transcriptional regulator |
| TON_0735 | 685723 | 685920 | + | 1986.56 | 2169.84 | 4106.83 | 1.09 | 2.07 | hypothetical protein |
| TON_0736 | 685922 | 686914 | + | 2247.79 | 2455.95 | 4620.09 | 1.09 | 2.06 | HypE protein |
| TON_0737 | 686911 | 687783 | - | 5976.70 | 4275.97 | 5063.69 | 0.72 | 0.85 | restriction endonuclease |
| TON_0738 | 687785 | 688531 | - | 9002.54 | 7146.65 | 7048.55 | 0.79 | 0.78 | hypothetical protein |
| TON_0739 | 688599 | 689390 | + | 1824.13 | 1942.64 | 1296.35 | 1.06 | 0.71 | glutamine amidotransferase |
| TON_0740 | 689434 | 689841 | + | 1002.93 | 514.51 | 522.79 | 0.51 | 0.52 | hypothetical protein |
| TON_0741 | 689847 | 691358 | + | 989.30 | 741.71 | 775.76 | 0.75 | 0.78 | metallophosphoesterase |
| TON_0742 | 691348 | 692238 | - | 5250.91 | 2665.12 | 2519.38 | 0.51 | 0.48 | 2-phosphoglycerate kinase |
| TON_0743 | 692235 | 693533 | - | 6760.42 | 3097.89 | 3121.36 | 0.46 | 0.46 | cyclic 2,3-diphospoglycerate synthetase |
| TON_0744 | 693681 | 693860 | + | 3193.94 | 8146.83 | 18313.18 | 2.55 | 5.73 | hypothetical protein |
| TON_0745 | 693931 | 694809 | + | 400.95 | 1456.98 | 1005.26 | 3.63 | 2.51 | zinc-dependent hydrolase |
| TON_0746 | 694826 | 695470 | - | 6149.35 | 967.71 | 1423.20 | 0.16 | 0.23 | exonuclease |
| TON_0747 | 695654 | 695989 | - | 4809.08 | 17605.17 | 4393.52 | 3.66 | 0.91 | hypothetical protein |
| TON_0748 | 696117 | 696956 | + | 1482.25 | 889.58 | 1904.20 | 0.60 | 1.28 | hypothetical protein |
| TON_0749 | 697357 | 699222 | + | 1589.02 | 2008.76 | 1247.23 | 1.26 | 0.78 | formaldehyde:ferredoxin oxidoreductase |
| TON_0750 | 699287 | 699517 | + | 147.66 | 125.02 | 141.51 | 0.85 | 0.96 | hypothetical protein |
| TON_0751 | 699796 | 700104 | - | 14170.53 | 13786.01 | 12167.23 | 0.97 | 0.86 | rps10p 30S ribosomal protein S10P |
| TON_0752 | 700151 | 701437 | - | 30022.09 | 35807.80 | 39410.43 | 1.19 | 1.31 | elongation factor 1-alpha |
| TON_0753 | 701688 | 702080 | - | 140.84 | 54.10 | 28.60 | 0.38 | 0.20 | DEXX-box atpase, C-terminus |
| TON_0754 | 702389 | 702925 | - | 208.99 | 488.06 | 350.48 | 2.34 | 1.68 | DEXX-box atpase, N-terminus |

| | | | | | | | | | |
|---|---|---|---|---|---|---|---|---|---|
| TON_0755 | 703075 | 705273 | - | 13280.05 | 20039.48 | 33679.50 | 1.51 | 2.54 | elongation factor EF-2 |
| TON_0756 | 705418 | 706482 | - | 2225.08 | 2650.69 | 2921.19 | 1.19 | 1.31 | metal-dependent phosphohydrolase |
| TON_0757 | 706509 | 707315 | - | 7614.56 | 6005.83 | 6596.15 | 0.79 | 0.87 | GTP cyclohydrolase |
| TON_0758 | 707341 | 707508 | - | 126.08 | 314.96 | 109.98 | 2.50 | 0.87 | hypothetical protein |
| TON_0759 | 707512 | 707637 | - | 15.90 | 158.68 | 27.86 | 9.98 | 1.75 | hypothetical protein |
| TON_0760 | 707732 | 708133 | - | 107.90 | 349.82 | 228.03 | 3.24 | 2.11 | general substrate transporter |
| TON_0761 | 708121 | 708531 | - | 421.39 | 2607.42 | 1085.18 | 6.19 | 2.58 | DNA-binding protein |
| TON_0762 | 708536 | 709783 | - | 2725.98 | 12125.87 | 8139.60 | 4.45 | 2.99 | hypothetical protein |
| TON_0763 | 709780 | 710940 | - | 3082.63 | 12807.48 | 9229.18 | 4.15 | 2.99 | DNA topoisomerase VI subunit A |
| TON_0764 | 710933 | 712624 | - | 4589.86 | 19109.04 | 13498.05 | 4.16 | 2.94 | DNA topoisomerase VI subunit B |
| TON_0765 | 712627 | 713331 | - | 3446.09 | 7262.06 | 7437.16 | 2.11 | 2.16 | putative RNA-processing protein |
| TON_0766 | 713337 | 714116 | - | 2236.44 | 3409.24 | 3578.90 | 1.52 | 1.60 | serine/threonine protein kinase |
| TON_0767 | 714144 | 714500 | - | 7878.07 | 5496.13 | 7630.73 | 0.70 | 0.97 | translation initiation factor IF-1 |
| TON_0768 | 714681 | 715475 | + | 1808.23 | 770.56 | 2169.63 | 0.43 | 1.20 | ABC-type manganese/zinc transport system, ATPase component |
| TON_0769 | 715472 | 716293 | + | 491.81 | 817.45 | 630.58 | 1.66 | 1.28 | manganese/zinc ABC transporter permease |
| TON_0770 | 716298 | 716981 | + | 1276.67 | 3055.81 | 1863.87 | 2.39 | 1.46 | rnhB ribonuclease HII |
| TON_0771 | 717010 | 718395 | - | 1010.88 | 1193.71 | 835.88 | 1.18 | 0.83 | membrane-bound dolichyl-phosphate-mannose-protein mannosyltransferase |
| TON_0772 | 718455 | 719603 | + | 4784.09 | 1333.16 | 4283.53 | 0.28 | 0.90 | hypothetical protein |
| TON_0773 | 719598 | 719792 | - | 449.79 | 361.84 | 258.10 | 0.80 | 0.57 | hypothetical protein |
| TON_0774 | 719961 | 721769 | + | 3926.54 | 4588.52 | 9094.26 | 1.17 | 2.32 | glucosamine--fructose-6-phosphate aminotransferase |
| TON_0775 | 721780 | 724644 | + | 2614.67 | 8280.26 | 9118.46 | 3.17 | 3.49 | oligosaccharyl transferase |
| TON_0776 | 724641 | 724835 | - | 52.25 | 98.57 | 27.86 | 1.89 | 0.53 | inosine-5'-monophosphate dehydrogenase-like protein III |
| TON_0777 | 725041 | 725712 | - | 895.03 | 2204.70 | 858.61 | 2.46 | 0.96 | UbiE/COQ5 methyltransferase |
| TON_0778 | 725713 | 725994 | - | 1305.06 | 2672.33 | 1250.16 | 2.05 | 0.96 | hypothetical protein |
| TON_0779 | 725994 | 726278 | - | 2889.54 | 2568.95 | 1545.65 | 0.89 | 0.53 | molybdopterin converting factor protein subunit 1 |
| TON_0780 | 726320 | 726994 | - | 4043.53 | 5922.89 | 1701.10 | 1.46 | 0.42 | adk adenylate kinase |
| TON_0781 | 727050 | 727496 | + | 314.62 | 477.25 | 224.37 | 1.52 | 0.71 | hypothetical protein |
| TON_0782 | 727480 | 728091 | - | 538.38 | 278.89 | 454.60 | 0.52 | 0.84 | hypothetical protein |
| TON_0783 | 730258 | 732516 | - | 1554.94 | 4340.89 | 8073.61 | 2.79 | 5.19 | cationic amino acid transporter (cat-1) |
| TON_0784 | 732519 | 732713 | - | 114.72 | 161.09 | 270.56 | 1.40 | 2.36 | hypothetical protein |
| TON_0785 | 732788 | 734209 | - | 2045.62 | 2235.96 | 2731.29 | 1.09 | 1.34 | pyruvate kinase |

| | | | | | | | | | |
|---|---|---|---|---|---|---|---|---|---|
| TON_0786 | 734306 | 734767 | + | 10485.92 | 23917.55 | 3655.16 | 2.28 | 0.35 | peroxiredoxin, bacterioferritin comigratory protein |
| TON_0787 | 734929 | 735396 | - | 1007.48 | 774.17 | 790.42 | 0.77 | 0.78 | hypothetical protein |
| TON_0788 | 735506 | 735706 | + | 123.80 | 116.61 | 184.77 | 0.94 | 1.49 | hypothetical protein |
| TON_0789 | 735703 | 736755 | - | 4563.74 | 2174.65 | 2078.71 | 0.48 | 0.46 | pyruvate formate lyase activating protein-like protein |
| TON_0790 | 736795 | 737433 | - | 1235.78 | 539.76 | 539.66 | 0.44 | 0.44 | putative zinc-dependent protease |
| TON_0791 | 737617 | 738474 | + | 850.73 | 431.56 | 666.51 | 0.51 | 0.78 | hypothetical protein |
| TON_0792 | 738507 | 739166 | + | 182.87 | 138.24 | 113.65 | 0.76 | 0.62 | hypothetical protein |
| TON_0793 | 739157 | 739456 | + | 104.50 | 85.35 | 78.46 | 0.82 | 0.75 | hypothetical protein |
| TON_0794 | 739453 | 739878 | + | 205.58 | 193.54 | 326.29 | 0.94 | 1.59 | hypothetical protein |
| TON_0795 | 739912 | 740283 | + | 47.70 | 67.32 | 51.33 | 1.41 | 1.08 | camphor resistance protein CrcB |
| TON_0796 | 740288 | 740668 | + | 333.93 | 234.42 | 313.09 | 0.70 | 0.94 | hypothetical protein |
| TON_0797 | 740787 | 741296 | + | 8299.46 | 7345.01 | 2578.04 | 0.88 | 0.31 | hypothetical protein |
| TON_0798 | 741380 | 742594 | + | 15851.55 | 28739.29 | 13279.54 | 1.81 | 0.84 | bifunctional phosphopantothenoylcysteine decarboxylase/phosphopantothenate synthase |
| TON_0799 | 742731 | 743468 | + | 3455.18 | 7519.31 | 6560.22 | 2.18 | 1.90 | small-conductance mechanosensitive channel |
| TON_0800 | 743555 | 744277 | + | 751.92 | 2398.25 | 1543.45 | 3.19 | 2.05 | ATPase |
| TON_0801 | 744330 | 745010 | + | 4068.52 | 2185.47 | 1424.67 | 0.54 | 0.35 | aspartate racemase |
| TON_0802 | 745052 | 745345 | + | 398.67 | 597.46 | 471.47 | 1.50 | 1.18 | nucleotide pyrophosphohydrolase |
| TON_0803 | 745342 | 746478 | - | 432.75 | 729.69 | 784.56 | 1.69 | 1.81 | permease |
| TON_0804 | 746619 | 748040 | + | 884.81 | 647.95 | 443.60 | 0.73 | 0.50 | zinc-dependent protease |
| TON_0805 | 748051 | 749373 | + | 741.69 | 628.71 | 462.67 | 0.85 | 0.62 | zinc-dependent protease |
| TON_0806 | 749627 | 750235 | + | 30.67 | 26.45 | 25.66 | 0.86 | 0.84 | membrane protein |
| TON_0807 | 750255 | 751712 | + | 757.59 | 724.88 | 665.04 | 0.96 | 0.88 | hypothetical protein |
| TON_0808 | 751740 | 752207 | - | 148.79 | 456.81 | 381.28 | 3.07 | 2.56 | membrane protein |
| TON_0809 | 752200 | 752850 | - | 241.93 | 455.61 | 508.13 | 1.88 | 2.10 | membrane-associated phosphatase |
| TON_0810 | 752843 | 753127 | - | 258.97 | 543.36 | 682.64 | 2.10 | 2.64 | hypothetical protein |
| TON_0811 | 753129 | 753662 | - | 3965.16 | 2750.47 | 4027.64 | 0.69 | 1.02 | regulatory protein |
| TON_0812 | 753871 | 754386 | + | 18123.20 | 5319.42 | 7972.42 | 0.29 | 0.44 | hypothetical protein |
| TON_0813 | 754452 | 755324 | + | 729.20 | 253.65 | 324.82 | 0.35 | 0.45 | membrane protein |
| TON_0814 | 755300 | 756523 | + | 796.21 | 371.46 | 533.06 | 0.47 | 0.67 | N-acetylglucosaminyl-phosphatidylinositol biosynthetic protein |
| TON_0815 | 757004 | 757261 | - | 916.61 | 1846.47 | 1337.41 | 2.01 | 1.46 | hypothetical protein |
| TON_0816 | 757828 | 758409 | - | 8217.68 | 4819.33 | 4179.42 | 0.59 | 0.51 | hypothetical protein |

| | | | | | | | | | |
|---|---|---|---|---|---|---|---|---|---|
| TON_0817 | 758366 | 759094 | - | 11599.03 | 8101.14 | 4837.86 | 0.70 | 0.42 | hydrolase |
| TON_0818 | 759128 | 759544 | - | 10764.20 | 4956.38 | 2965.92 | 0.46 | 0.28 | hypothetical protein |
| TON_0819 | 759541 | 760293 | - | 6635.48 | 3858.83 | 1497.99 | 0.58 | 0.23 | NAD-dependent deacetylase |
| TON_0820 | 760441 | 761625 | + | 24831.37 | 7587.84 | 9379.49 | 0.31 | 0.38 | aromatic amino acid permease |
| TON_0821 | 761764 | 763047 | + | 4775.00 | 5486.51 | 2454.12 | 1.15 | 0.51 | glyA serine hydroxymethyltransferase |
| TON_0822 | 763497 | 763949 | + | 2694.17 | 579.43 | 665.77 | 0.22 | 0.25 | hypothetical protein |
| TON_0823 | 763946 | 764368 | + | 1355.04 | 191.14 | 264.70 | 0.14 | 0.20 | hypothetical protein |
| TON_0824 | 764434 | 764766 | + | 4012.86 | 1918.60 | 3007.71 | 0.48 | 0.75 | DNA-directed RNA polymerase subunit M |
| TON_0825 | 764763 | 764930 | + | 1358.44 | 1126.39 | 1389.47 | 0.83 | 1.02 | hypothetical protein |
| TON_0826 | 764933 | 765682 | + | 2685.09 | 2698.78 | 3838.46 | 1.01 | 1.43 | DNA polymerase sliding clamp |
| TON_0827 | 765717 | 766286 | + | 1917.27 | 1116.78 | 2297.95 | 0.58 | 1.20 | hypothetical protein |
| TON_0828 | 766292 | 768202 | + | 955.23 | 1132.41 | 1231.09 | 1.19 | 1.29 | molybdopterin oxidoreductase, molybdopterin-binding subunit |
| TON_0829 | 768428 | 769078 | + | 27135.96 | 61539.37 | 65814.07 | 2.27 | 2.43 | peroxiredoxin |
| TON_0830 | 769158 | 769862 | - | 465.69 | 592.65 | 303.56 | 1.27 | 0.65 | permease |
| TON_0831 | 769859 | 770563 | - | 906.39 | 711.66 | 527.93 | 0.79 | 0.58 | membrane protein |
| TON_0832 | 770560 | 771411 | - | 3760.71 | 3450.11 | 1858.01 | 0.92 | 0.49 | ATP-binding transport protein |
| TON_0833 | 771663 | 772019 | - | 52.25 | 39.67 | 24.20 | 0.76 | 0.46 | methyltransferase |
| TON_0834 | 772400 | 772717 | - | 182.87 | 900.39 | 331.42 | 4.92 | 1.81 | hypothetical protein |
| TON_0835 | 772728 | 775130 | - | 793.94 | 1120.38 | 1198.83 | 1.41 | 1.51 | heavy-metal transporting P-type ATPase |
| TON_0836 | 775247 | 775855 | + | 1665.12 | 1479.82 | 1031.66 | 0.89 | 0.62 | transcriptional regulatory protein |
| TON_0837 | 775888 | 776223 | - | 880.26 | 4677.48 | 1635.84 | 5.31 | 1.86 | hypothetical protein |
| TON_0838 | 776366 | 776980 | + | 290.77 | 244.03 | 186.97 | 0.84 | 0.64 | ubiquinone/menaquinone biosynthesis methyltransferase |
| TON_0839 | 776996 | 777229 | - | 1424.32 | 6202.98 | 2300.15 | 4.36 | 1.61 | hypothetical protein |
| TON_0840 | 777406 | 779334 | - | 11091.32 | 18646.22 | 4308.46 | 1.68 | 0.39 | copper-transporting ATPase |
| TON_0841 | 780424 | 780540 | - | 5.68 | 2.40 | 6.60 | 0.42 | 1.16 | Transcription regulatory protein |
| TON_0842 | 780711 | 781514 | - | 168.10 | 263.27 | 250.03 | 1.57 | 1.49 | putative transport protein |
| TON_0843 | 782057 | 782263 | - | 1598.10 | 1192.51 | 591.72 | 0.75 | 0.37 | hypothetical protein |
| TON_0844 | 782358 | 782735 | - | 897.30 | 500.09 | 494.93 | 0.56 | 0.55 | hypothetical protein |
| TON_0845 | 782741 | 783109 | - | 701.94 | 861.93 | 357.08 | 1.23 | 0.51 | hypothetical protein |
| TON_0846 | 783178 | 784035 | - | 2586.27 | 9802.16 | 666.51 | 3.79 | 0.26 | hypothetical protein |
| TON_0847 | 784305 | 784874 | - | 26240.93 | 100473.91 | 25896.98 | 3.83 | 0.99 | alkyl hydroperoxide reductase subunit c |
| TON_0848 | 785016 | 785591 | + | 1468.62 | 2731.24 | 2192.36 | 1.86 | 1.49 | hypothetical protein |

| | | | | | | | | | |
|---|---|---|---|---|---|---|---|---|---|
| TON_0849 | 785823 | 786938 | - | 2104.68 | 1128.80 | 1163.64 | 0.54 | 0.55 | hypothetical protein |
| TON_0850 | 786938 | 788365 | - | 4894.26 | 2475.18 | 3323.00 | 0.51 | 0.68 | hypothetical protein |
| TON_0851 | 788343 | 789086 | - | 4219.58 | 1995.53 | 2099.97 | 0.47 | 0.50 | ATP-dependent transporter |
| TON_0852 | 789237 | 789761 | - | 15174.60 | 43163.63 | 14774.60 | 2.84 | 0.97 | hypothetical protein |
| TON_0853 | 789829 | 790920 | - | 10479.11 | 2062.85 | 2492.99 | 0.20 | 0.24 | thiI |
| TON_0854 | 790922 | 791896 | - | 18621.83 | 1501.46 | 2641.10 | 0.08 | 0.14 | MoeB protein |
| TON_0855 | 792029 | 792568 | + | 591.76 | 614.29 | 634.24 | 1.04 | 1.07 | NAD(P)H oxidase |
| TON_0856 | 792678 | 792884 | + | 213.53 | 227.20 | 206.77 | 1.06 | 0.97 | hypothetical protein |
| TON_0857 | 792887 | 793216 | + | 447.51 | 532.54 | 579.25 | 1.19 | 1.29 | hypothetical protein |
| TON_0858 | 793276 | 794571 | + | 461.14 | 929.25 | 308.69 | 2.02 | 0.67 | cytochrome c-type biogenesis protein |
| TON_0859 | 794589 | 794705 | + | 20.44 | 64.91 | 16.13 | 3.18 | 0.79 | hypothetical protein |
| TON_0860 | 794852 | 796564 | + | 24194.18 | 66968.18 | 33362.74 | 2.77 | 1.38 | 2-oxoacid:ferredoxin oxidoreductase subunit alpha |
| TON_0861 | 796561 | 797442 | + | 6454.89 | 23252.77 | 12178.97 | 3.60 | 1.89 | 2-oxoacid:ferredoxin oxidoreductase subunit beta |
| TON_0862 | 797444 | 797896 | + | 4261.61 | 20133.25 | 12942.99 | 4.72 | 3.04 | thioredoxin peroxidase |
| TON_0863 | 797937 | 799166 | + | 9350.10 | 44925.95 | 21546.72 | 4.80 | 2.30 | type A flavoprotein |
| TON_0864 | 799239 | 799778 | + | 1892.28 | 7490.46 | 3174.89 | 3.96 | 1.68 | hypothetical protein |
| TON_0865 | 799788 | 800873 | + | 6219.77 | 24608.77 | 14004.71 | 3.96 | 2.25 | NAD(P)H:rubredoxin oxidoreductase |
| TON_0866 | 800962 | 801477 | + | 23003.83 | 68127.03 | 23125.37 | 2.96 | 1.01 | rubrerythrin |
| TON_0867 | 801560 | 801721 | + | 35701.21 | 169797.06 | 44799.67 | 4.76 | 1.25 | rubredoxin |
| TON_0868 | 801732 | 802079 | + | 24170.33 | 101734.94 | 37012.03 | 4.21 | 1.53 | sor superoxide reductase |
| TON_0869 | 802297 | 802656 | + | 873.45 | 3923.75 | 604.92 | 4.49 | 0.69 | hypothetical protein |
| TON_0870 | 802660 | 803010 | + | 3653.94 | 7141.85 | 2666.03 | 1.95 | 0.73 | hypothetical protein |
| TON_0871 | 803014 | 803538 | + | 6392.41 | 12148.71 | 5010.17 | 1.90 | 0.78 | rubrerythrin-like protein |
| TON_0872 | 803592 | 803906 | - | 1707.14 | 3206.08 | 1201.77 | 1.88 | 0.70 | hypothetical protein |
| TON_0873 | 803936 | 804415 | - | 812.11 | 1808.00 | 739.83 | 2.23 | 0.91 | rubrerythrin-like protein |
| TON_0874 | 804483 | 805007 | + | 1259.63 | 1810.41 | 1039.72 | 1.44 | 0.83 | N-acetyltransferase |
| TON_0875 | 804995 | 805348 | - | 1954.75 | 1048.26 | 1175.37 | 0.54 | 0.60 | hypothetical protein |
| TON_0876 | 805371 | 805979 | - | 8067.75 | 10933.36 | 7092.54 | 1.36 | 0.88 | hypothetical protein |
| TON_0877 | 806173 | 806766 | - | 14883.83 | 17349.12 | 10050.40 | 1.17 | 0.68 | hypothetical protein |
| TON_0878 | 807102 | 807989 | + | 29964.16 | 3405.63 | 25567.76 | 0.11 | 0.85 | ATP phosphoribosyltransferase |
| TON_0879 | 807989 | 808606 | + | 2382.96 | 538.55 | 3721.15 | 0.23 | 1.56 | hisG ATP phosphoribosyltransferase catalytic subunit |
| TON_0880 | 808596 | 809729 | + | 12037.46 | 3424.86 | 12629.17 | 0.28 | 1.05 | hisD histidinol dehydrogenase |

| | | | | | | | | | |
|---|---|---|---|---|---|---|---|---|---|
| TON_0881 | 809726 | 810256 | + | 3324.56 | 873.95 | 4919.98 | 0.26 | 1.48 | hisB imidazoleglycerol-phosphate dehydratase |
| TON_0882 | 810256 | 810843 | + | 3961.75 | 1167.27 | 5794.72 | 0.29 | 1.46 | hisH imidazole glycerol phosphate synthase subunit HisH |
| TON_0883 | 810845 | 811534 | + | 3736.86 | 815.04 | 5732.40 | 0.22 | 1.53 | 1-(5-phosphoribosyl)-5-[(5-phosphoribosylamino)methylideneamino] imidazole-4-carboxamide isomerase |
| TON_0884 | 811528 | 812286 | + | 1863.89 | 569.81 | 2579.51 | 0.31 | 1.38 | imidazole glycerol phosphate synthase subunit HisF |
| TON_0885 | 812296 | 812922 | + | 1929.76 | 685.21 | 2619.10 | 0.36 | 1.36 | bifunctional phosphoribosyl-AMP cyclohydrolase/phosphoribosyl-ATP pyrophosphatase |
| TON_0886 | 812919 | 813926 | + | 2231.89 | 823.46 | 2474.65 | 0.37 | 1.11 | histidinol-phosphate aminotransferase |
| TON_0887 | 813923 | 814645 | + | 947.28 | 378.67 | 1168.04 | 0.40 | 1.23 | hydrolase |
| TON_0888 | 814651 | 815439 | + | 952.96 | 667.18 | 1343.28 | 0.70 | 1.41 | pyrroline-5-carboxylate reductase |
| TON_0889 | 815677 | 816381 | + | 923.42 | 1460.59 | 1585.98 | 1.58 | 1.72 | ATPase |
| TON_0890 | 816496 | 816990 | + | 210.13 | 390.69 | 641.58 | 1.86 | 3.05 | DNA topoisomerase VI subunit B |
| TON_0891 | 817328 | 818323 | + | 690.58 | 962.91 | 1703.30 | 1.39 | 2.47 | N2, N2-dimethylguanosine tRNA methyltransferase |
| TON_0892 | 820128 | 820841 | + | 1535.63 | 424.35 | 464.87 | 0.28 | 0.30 | hypothetical protein |
| TON_0893 | 820846 | 823179 | + | 840.51 | 396.70 | 321.16 | 0.47 | 0.38 | hypothetical protein |
| TON_0894 | 823185 | 823745 | + | 101.09 | 176.71 | 116.58 | 1.75 | 1.15 | hypothetical protein |
| TON_0895 | 823745 | 824617 | + | 374.82 | 454.40 | 407.68 | 1.21 | 1.09 | hypothetical protein |
| TON_0896 | 824617 | 825486 | + | 233.98 | 292.12 | 315.29 | 1.25 | 1.35 | hypothetical protein |
| TON_0897 | 825483 | 826676 | + | 516.80 | 760.95 | 743.50 | 1.47 | 1.44 | hypothetical protein |
| TON_0898 | 826673 | 827971 | + | 312.35 | 461.62 | 514.73 | 1.48 | 1.65 | hypothetical protein |
| TON_0899 | 828943 | 829587 | - | 3203.02 | 2653.10 | 2943.92 | 0.83 | 0.92 | alanyl-tRNA synthetase |
| TON_0900 | 829649 | 831103 | - | 8048.45 | 7347.41 | 9158.79 | 0.91 | 1.14 | hypothetical protein |
| TON_0901 | 831224 | 832498 | - | 2472.69 | 1634.89 | 654.04 | 0.66 | 0.26 | proton/glutamate symporter |
| TON_0902 | 832602 | 833624 | - | 245.34 | 324.57 | 284.49 | 1.32 | 1.16 | biotin--protein ligase |
| TON_0903 | 833434 | 834279 | + | 904.12 | 984.54 | 4385.45 | 1.09 | 4.85 | fructose-bisphosphate aldolase |
| TON_0904 | 834406 | 836190 | + | 2656.69 | 10261.37 | 91765.31 | 3.86 | 34.54 | pyruvate carboxylase subunit B |
| TON_0905 | 836374 | 838074 | + | 897.30 | 7716.46 | 4962.51 | 8.60 | 5.53 | cstA |
| TON_0906 | 838098 | 838379 | + | 116.99 | 560.19 | 727.37 | 4.79 | 6.22 | hypothetical protein |
| TON_0907 | 838389 | 839381 | + | 1568.57 | 3704.96 | 5576.95 | 2.36 | 3.56 | arsA protein |
| TON_0908 | 839371 | 839664 | + | 533.84 | 2092.91 | 2252.49 | 3.92 | 4.22 | hypothetical protein |
| TON_0909 | 839667 | 840323 | + | 1177.85 | 4711.14 | 4886.98 | 4.00 | 4.15 | membrane protein |
| TON_0910 | 840401 | 841192 | + | 1790.06 | 3469.34 | 4661.15 | 1.94 | 2.60 | lysophospholipase |

| | | | | | | | | | |
|---|---|---|---|---|---|---|---|---|---|
| TON_0911 | 841189 | 842208 | - | 3830.00 | 1400.48 | 1557.38 | 0.37 | 0.41 | adenylosuccinate synthetase |
| TON_0912 | 842343 | 843269 | + | 5640.50 | 5861.58 | 4864.25 | 1.04 | 0.86 | UDP-glucose 4-epimerase |
| TON_0913 | 843254 | 844696 | - | 15291.59 | 15325.94 | 10398.68 | 1.00 | 0.68 | hypothetical protein |
| TON_0914 | 844895 | 845338 | - | 885.94 | 1369.22 | 1050.72 | 1.55 | 1.19 | prefoldin subunit alpha |
| TON_0915 | 845483 | 846370 | - | 6013.05 | 786.19 | 163.51 | 0.13 | 0.03 | ATPase, N-terminus |
| TON_0916 | 846370 | 847239 | - | 17972.14 | 2440.32 | 340.95 | 0.14 | 0.02 | ATPase C-terminus |
| TON_0917 | 847311 | 847709 | + | 3301.84 | 313.76 | 75.52 | 0.10 | 0.02 | iron-molybdenum cofactor-binding protein |
| TON_0918 | 848076 | 848615 | + | 1068.81 | 438.78 | 689.24 | 0.41 | 0.64 | hypothetical protein |
| TON_0919 | 848935 | 849528 | + | 142389.11 | 181.52 | 92.39 | 0.00 | 0.00 | iron-molybdenum cofactor-binding protein |
| TON_0920 | 849562 | 849789 | + | 25621.91 | 39.67 | 17.60 | 0.00 | 0.00 | hypothetical protein |
| TON_0921 | 849845 | 850252 | + | 11974.99 | 611.88 | 53.53 | 0.05 | 0.00 | iron-molybdenum cofactor-binding protein |
| TON_0922 | 850366 | 850470 | + | 16108.25 | 1544.74 | 357.08 | 0.10 | 0.02 | short-chain alcohol dehydrogenase |
| TON_0923 | 850542 | 851156 | + | 8191.56 | 308.95 | 135.65 | 0.04 | 0.02 | short-chain alcohol dehydrogenase |
| TON_0924 | 851459 | 852040 | + | 4016.27 | 1555.55 | 766.96 | 0.39 | 0.19 | membrane protein |
| TON_0925 | 852037 | 852456 | - | 1074.49 | 417.14 | 225.84 | 0.39 | 0.21 | DNA-binding protein |
| TON_0926 | 852513 | 853088 | + | 1506.10 | 585.44 | 881.34 | 0.39 | 0.59 | hypothetical protein |
| TON_0927 | 853085 | 853543 | - | 3592.61 | 3590.76 | 2374.20 | 1.00 | 0.66 | 6-pyruvoyl-tetrahydropterin synthase |
| TON_0928 | 853649 | 854005 | + | 6100.51 | 12726.94 | 8404.29 | 2.09 | 1.38 | hypothetical protein |
| TON_0929 | 855641 | 856996 | - | 252.15 | 199.55 | 326.29 | 0.79 | 1.29 | adenylosuccinate lyase |
| TON_0930 | 857189 | 857464 | + | 6449.21 | 7108.19 | 15533.50 | 1.10 | 2.41 | DNA/RNA-binding protein albA |
| TON_0931 | 857576 | 858205 | + | 3633.50 | 1386.05 | 1360.14 | 0.38 | 0.37 | transcriptional regulator |
| TON_0932 | 858255 | 858800 | + | 19130.68 | 4499.57 | 8484.21 | 0.24 | 0.44 | hypothetical protein |
| TON_0933 | 858797 | 859315 | - | 575.86 | 235.62 | 410.61 | 0.41 | 0.71 | putative NTPase |
| TON_0934 | 859371 | 860441 | + | 3444.95 | 1801.99 | 2868.40 | 0.52 | 0.83 | translation initiation factor IF-2 |
| TON_0935 | 860393 | 860674 | - | 194.23 | 10.82 | 5.87 | 0.06 | 0.03 | hypothetical protein |
| TON_0936 | 860830 | 861963 | + | 1790.06 | 2082.09 | 1167.30 | 1.16 | 0.65 | Fe-containing alcohol dehydrogenase |
| TON_0937 | 861993 | 864770 | + | 4073.06 | 2726.43 | 5670.81 | 0.67 | 1.39 | large helicase-like protein |
| TON_0938 | 864851 | 865903 | - | 2092.19 | 1942.64 | 2051.58 | 0.93 | 0.98 | acetylornithine deacetylase |
| TON_0939 | 866053 | 866754 | + | 3493.79 | 2090.50 | 4858.39 | 0.60 | 1.39 | hydrolase |
| TON_0940 | 866754 | 867017 | + | 3120.11 | 1579.60 | 4035.70 | 0.51 | 1.29 | hypothetical protein |
| TON_0941 | 867010 | 867768 | + | 5108.93 | 2631.46 | 6030.09 | 0.52 | 1.18 | TatD-related deoxyribonuclease |
| TON_0942 | 867772 | 868065 | + | 1439.09 | 827.06 | 1875.60 | 0.57 | 1.30 | hypothetical protein |

| | | | | | | | | | |
|---|---|---|---|---|---|---|---|---|---|
| TON_0943 | 868088 | 869668 | - | 2782.77 | 815.04 | 1094.71 | 0.29 | 0.39 | Pterin-binding protein |
| TON_0944 | 869735 | 870481 | + | 1987.69 | 1020.61 | 1937.20 | 0.51 | 0.97 | hypothetical protein |
| TON_0945 | 870555 | 871559 | - | 313.49 | 299.33 | 219.97 | 0.95 | 0.70 | glyoxylate reductase |
| TON_0946 | 871634 | 872848 | + | 2389.77 | 2547.31 | 803.62 | 1.07 | 0.34 | alanine glyoxylate transaminase |
| TON_0947 | 872985 | 874244 | + | 417.98 | 388.29 | 899.67 | 0.93 | 2.15 | 23S rRNA (uracil-5-)-methyltransferase |
| TON_0948 | 874248 | 876041 | - | 10029.32 | 6198.18 | 10060.66 | 0.62 | 1.00 | translation initiation factor IF-2 |
| TON_0949 | 876141 | 876419 | + | 17.04 | 12.02 | 13.93 | 0.71 | 0.82 | hypothetical protein |
| TON_0950 | 876422 | 876595 | + | 23.85 | 14.43 | 12.46 | 0.60 | 0.52 | hypothetical protein |
| TON_0951 | 876598 | 877110 | - | 4913.57 | 2689.16 | 4394.99 | 0.55 | 0.89 | ndk nucleoside diphosphate kinase |
| TON_0952 | 877200 | 877406 | - | 2678.27 | 2239.57 | 4120.76 | 0.84 | 1.54 | 50S ribosomal protein L24e |
| TON_0953 | 877412 | 877624 | - | 11231.02 | 8001.37 | 14519.44 | 0.71 | 1.29 | rps28e 30S ribosomal protein S28e |
| TON_0954 | 877703 | 878074 | - | 27606.19 | 28964.09 | 33128.11 | 1.05 | 1.20 | rpl7ae 50S ribosomal protein L7Ae |
| TON_0955 | 878252 | 878863 | - | 3907.23 | 1628.88 | 1902.73 | 0.42 | 0.49 | hypothetical protein |
| TON_0956 | 878905 | 879852 | - | 1693.51 | 1981.11 | 3850.20 | 1.17 | 2.27 | hypothetical protein |
| TON_0957 | 879804 | 881696 | - | 5269.09 | 4431.05 | 6953.96 | 0.84 | 1.32 | hypothetical protein |
| TON_0958 | 881811 | 882767 | + | 136.30 | 866.73 | 546.99 | 6.36 | 4.01 | membrane protease subunit |
| TON_0959 | 882787 | 883200 | + | 153.34 | 146.66 | 161.31 | 0.96 | 1.05 | membrane protein |
| TON_0960 | 883181 | 884443 | - | 3140.55 | 1532.71 | 1750.22 | 0.49 | 0.56 | metal-dependent amidohydrolase |
| TON_0961 | 884685 | 885977 | + | 36006.74 | 17571.51 | 25201.15 | 0.49 | 0.70 | asnC asparaginyl-tRNA synthetase |
| TON_0962 | 886004 | 886240 | - | 169.24 | 58.90 | 54.26 | 0.35 | 0.32 | hypothetical protein |
| TON_0963 | 886790 | 887932 | - | 72.69 | 119.01 | 134.18 | 1.64 | 1.85 | thiamin-binding periplasmic protein |
| TON_0964 | 888060 | 888995 | - | 1044.96 | 819.85 | 1053.65 | 0.78 | 1.01 | Ca2+/Na+ antiporter |
| TON_0965 | 889034 | 889546 | - | 664.46 | 585.44 | 657.71 | 0.88 | 0.99 | hypothetical protein |
| TON_0966 | 889543 | 890055 | - | 2273.92 | 2457.15 | 2083.84 | 1.08 | 0.92 | hypothetical protein |
| TON_0967 | 890268 | 890642 | + | 68.15 | 44.48 | 33.73 | 0.65 | 0.49 | hypothetical protein |
| TON_0968 | 890696 | 893128 | - | 2234.16 | 1597.63 | 2275.22 | 0.72 | 1.02 | ATPase |
| TON_0969 | 893255 | 895150 | + | 2351.15 | 1832.04 | 884.28 | 0.78 | 0.38 | acylamino acid-releasing protein |
| TON_0970 | 895326 | 895916 | + | 146.52 | 121.41 | 174.51 | 0.83 | 1.19 | molybdopterin-guanine dinucleotide biosynthesis protein A |
| TON_0971 | 896097 | 896462 | + | 1256.22 | 68.52 | 360.75 | 0.05 | 0.29 | hypothetical protein |
| TON_0972 | 896429 | 897076 | + | 1039.28 | 44.48 | 83.59 | 0.04 | 0.08 | pyrolysin-related protein |
| TON_0973 | 897084 | 897599 | - | 194.23 | 206.77 | 255.16 | 1.06 | 1.31 | hypothetical protein |
| TON_0974 | 897647 | 898438 | + | 1504.97 | 645.54 | 1616.04 | 0.43 | 1.07 | cobalt ABC transporter ATPase |

| | | | | | | | | | |
|---|---|---|---|---|---|---|---|---|---|
| TON_0975 | 898435 | 899196 | + | 349.83 | 269.28 | 470.00 | 0.77 | 1.34 | cobalt ABC transporter permease |
| TON_0976 | 899193 | 899822 | - | 544.06 | 412.33 | 426.74 | 0.76 | 0.78 | hypothetical protein |
| TON_0977 | 899889 | 900281 | - | 1495.88 | 3472.95 | 734.70 | 2.32 | 0.49 | hypothetical protein |
| TON_0978 | 900376 | 901167 | + | 717.84 | 757.34 | 1015.52 | 1.06 | 1.41 | membrane protein |
| TON_0979 | 901246 | 903786 | - | 2739.61 | 6600.89 | 6327.78 | 2.41 | 2.31 | DEAD/DEAH box helicase |
| TON_0980 | 903834 | 904295 | - | 871.18 | 1223.77 | 2209.22 | 1.40 | 2.54 | transcriptional regulator |
| TON_0981 | 904321 | 905448 | - | 9531.83 | 5732.95 | 8440.95 | 0.60 | 0.89 | tyrosyl-tRNA synthetase |
| TON_0982 | 905572 | 906690 | + | 11921.60 | 22602.42 | 13641.76 | 1.90 | 1.14 | hypothetical protein |
| TON_0983 | 906746 | 908197 | + | 9941.86 | 35390.67 | 26067.09 | 3.56 | 2.62 | iron(III) ABC transporter periplasmic protein |
| TON_0984 | 908345 | 910189 | + | 2931.56 | 10085.86 | 17159.07 | 3.44 | 5.85 | iron(III) ABC transporter permease |
| TON_0985 | 910200 | 911264 | + | 3752.76 | 10361.15 | 18082.94 | 2.76 | 4.82 | ABC-type iron(III) transport system, ATPase component |
| TON_0986 | 911299 | 911898 | + | 513.39 | 757.34 | 891.61 | 1.48 | 1.74 | hypothetical protein |
| TON_0987 | 911967 | 913985 | + | 8438.03 | 17130.33 | 14097.10 | 2.03 | 1.67 | metallophosphoesterase |
| TON_0988 | 914076 | 914699 | + | 6060.75 | 2267.21 | 5257.27 | 0.37 | 0.87 | hypothetical protein |
| TON_0989 | 914700 | 916838 | - | 4068.52 | 5467.28 | 4502.77 | 1.34 | 1.11 | type II restriction endonuclease |
| TON_0990 | 916982 | 918226 | + | 708.75 | 328.18 | 517.66 | 0.46 | 0.73 | pgk phosphoglycerate kinase |
| TON_0991 | 918184 | 918528 | - | 1410.69 | 32.46 | 43.99 | 0.02 | 0.03 | hypothetical protein |
| TON_0992 | 918541 | 919305 | - | 5658.67 | 122.62 | 192.11 | 0.02 | 0.03 | ABC type transporter permease |
| TON_0993 | 919310 | 920296 | - | 29773.34 | 467.63 | 629.85 | 0.02 | 0.02 | ABC-type multidrug transporter, ATPase component |
| TON_0994 | 920298 | 920747 | - | 20457.32 | 203.16 | 486.87 | 0.01 | 0.02 | hypothetical protein |
| TON_0995 | 920844 | 921275 | + | 345.29 | 697.23 | 423.81 | 2.02 | 1.23 | hypothetical protein |
| TON_0996 | 921241 | 921972 | + | 1347.09 | 1845.27 | 1269.22 | 1.37 | 0.94 | endonuclease III |
| TON_0997 | 921973 | 922551 | - | 14300.02 | 2995.70 | 4918.51 | 0.21 | 0.34 | phosphate transport system regulator |
| TON_0998 | 922544 | 923152 | - | 37256.15 | 4248.32 | 9206.45 | 0.11 | 0.25 | phosphate transport system regulator |
| TON_0999 | 923272 | 924441 | + | 203.31 | 328.18 | 329.95 | 1.61 | 1.62 | hypothetical protein |
| TON_1000 | 924458 | 925210 | + | 236.25 | 305.34 | 398.14 | 1.29 | 1.69 | hypothetical protein |
| TON_1001 | 925314 | 926732 | + | 19437.35 | 25325.24 | 18136.47 | 1.30 | 0.93 | acyl-CoA synthetase large subunit |
| TON_1002 | 926764 | 927489 | + | 5216.84 | 19933.70 | 7666.66 | 3.82 | 1.47 | acetyl-CoA synthetase II subunit beta |
| TON_1003 | 927521 | 927856 | - | 626.97 | 212.78 | 203.84 | 0.34 | 0.33 | hypothetical protein |
| TON_1004 | 927853 | 928674 | - | 52814.66 | 5499.74 | 15766.66 | 0.10 | 0.30 | cysteine synthase |
| TON_1005 | 929227 | 930207 | + | 1072.22 | 1656.53 | 1361.61 | 1.54 | 1.27 | hypothetical protein |
| TON_1006 | 930299 | 930889 | - | 9838.50 | 1543.53 | 843.22 | 0.16 | 0.09 | hypothetical protein |

| | | | | | | | | | |
|---|---|---|---|---|---|---|---|---|---|
| TON_1007 | 930932 | 931486 | - | 34.07 | 163.49 | 74.79 | 4.80 | 2.19 | membrane protein |
| TON_1008 | 931496 | 932647 | - | 9914.60 | 5830.32 | 7490.69 | 0.59 | 0.76 | thiamine biosynthesis protein ThiI |
| TON_1009 | 932647 | 932973 | - | 1507.24 | 640.73 | 1179.04 | 0.43 | 0.78 | membrane protein |
| TON_1010 | 933015 | 933623 | - | 10806.23 | 3311.86 | 2040.58 | 0.31 | 0.19 | NAD(P)H-flavin oxidoreductase |
| TON_1011 | 933720 | 934433 | + | 3443.82 | 2699.98 | 4922.18 | 0.78 | 1.43 | maleate cis-trans isomerase |
| TON_1012 | 934533 | 935912 | + | 316.89 | 355.83 | 926.07 | 1.12 | 2.92 | ADP-specific phosphofructokinase |
| TON_1013 | 935913 | 936761 | - | 2005.86 | 1247.81 | 1398.27 | 0.62 | 0.70 | integrase/recombinase |
| TON_1014 | 936763 | 937656 | - | 8194.97 | 6366.47 | 7367.50 | 0.78 | 0.90 | hypothetical protein |
| TON_1015 | 937706 | 938428 | - | 659.91 | 621.50 | 768.43 | 0.94 | 1.16 | sugar fermentation stimulation protein A |
| TON_1016 | 939053 | 940156 | + | 4314.99 | 6537.18 | 2363.94 | 1.51 | 0.55 | putative transcriptional regulator |
| TON_1017 | 940254 | 940892 | + | 180.60 | 817.45 | 91.65 | 4.53 | 0.51 | 4Fe-4S ferredoxin |
| TON_1018 | 940903 | 942795 | + | 997.25 | 4937.14 | 733.96 | 4.95 | 0.74 | carbon-monoxide dehydrogenase, catalytic subunit |
| TON_1019 | 942863 | 943672 | + | 77.24 | 801.82 | 195.04 | 10.38 | 2.53 | ATP-binding protein |
| TON_1020 | 943673 | 943867 | + | 13.63 | 195.95 | 44.73 | 14.38 | 3.28 | RNA-binding protein |
| TON_1021 | 943872 | 945776 | + | 34.07 | 519.32 | 109.98 | 15.24 | 3.23 | Hydrogenase 4 subunit 3 |
| TON_1022 | 945773 | 946654 | + | 60.20 | 549.37 | 152.51 | 9.13 | 2.53 | Respiratory-chain NADH dehydrogenase, subunit 1 |
| TON_1023 | 946658 | 948280 | + | 277.14 | 1795.98 | 479.53 | 6.48 | 1.73 | Hydrogenase 4 subunit 5 |
| TON_1024 | 948784 | 949359 | + | 137.43 | 1075.91 | 245.63 | 7.83 | 1.79 | NADH dehydrogenase (ubiquinone), 20 kDa subunit |
| TON_1025 | 949349 | 949633 | + | 31.80 | 211.57 | 79.19 | 6.65 | 2.49 | Na+/H+ antiporter subunit MnhF |
| TON_1026 | 949630 | 949989 | + | 36.35 | 282.50 | 74.06 | 7.77 | 2.04 | Na+/H+ antiporter subunit |
| TON_1027 | 949982 | 950236 | + | 1.14 | 43.28 | 3.67 | 38.10 | 3.23 | hypothetical protein |
| TON_1028 | 950237 | 951007 | + | 69.29 | 431.56 | 101.19 | 6.23 | 1.46 | Multisubunit Na+/H+ antiporter, MnhB subunit |
| TON_1029 | 951000 | 951404 | + | 134.03 | 421.95 | 126.85 | 3.15 | 0.95 | hypothetical protein |
| TON_1030 | 951401 | 951886 | + | 93.14 | 413.53 | 110.72 | 4.44 | 1.19 | hypothetical protein |
| TON_1031 | 951897 | 953459 | + | 105.63 | 798.21 | 145.18 | 7.56 | 1.37 | putative monovalent cation/H+ antiporter subunit D |
| TON_1032 | 953602 | 954648 | + | 3918.59 | 5588.70 | 3848.73 | 1.43 | 0.98 | deblocking aminopeptidase |
| TON_1033 | 954845 | 955219 | + | 2561.28 | 1000.17 | 1041.92 | 0.39 | 0.41 | hypothetical protein |
| TON_1034 | 955262 | 956338 | + | 63.61 | 86.55 | 45.46 | 1.36 | 0.71 | hypothetical protein |
| TON_1035 | 956367 | 957080 | + | 7.95 | 20.44 | 11.73 | 2.57 | 1.48 | membrane protein |
| TON_1036 | 957092 | 957538 | + | 39.75 | 85.35 | 52.06 | 2.15 | 1.31 | hypothetical protein |
| TON_1037 | 957535 | 958188 | + | 327.12 | 557.79 | 666.51 | 1.71 | 2.04 | multidrug ABC transporter ATPase |
| TON_1038 | 958400 | 959104 | + | 313.49 | 215.18 | 170.84 | 0.69 | 0.54 | hypothetical protein |

| | | | | | | | | | |
|---|---|---|---|---|---|---|---|---|---|
| TON_1039 | 959117 | 959497 | - | 1510.65 | 815.04 | 1372.61 | 0.54 | 0.91 | membrane protein |
| TON_1040 | 959421 | 961421 | + | 2566.96 | 1969.09 | 5058.56 | 0.77 | 1.97 | ERCC2/XPD/Rad3-related DNA repair helicase |
| TON_1041 | 962122 | 962868 | - | 1409.56 | 1044.65 | 678.97 | 0.74 | 0.48 | hypothetical protein |
| TON_1042 | 963130 | 964341 | + | 13047.21 | 2803.36 | 28043.15 | 0.21 | 2.15 | DEAD/DEAH box helicase |
| TON_1043 | 964335 | 965516 | - | 4416.08 | 1437.75 | 2080.91 | 0.33 | 0.47 | hypothetical protein |
| TON_1044 | 966022 | 966630 | - | 849.60 | 1151.64 | 1456.93 | 1.36 | 1.71 | integral membrane protein |
| TON_1045 | 966620 | 967402 | - | 5922.18 | 6521.55 | 7465.02 | 1.10 | 1.26 | bifunctional inositol-1 monophosphatase/fructose-1,6-bisphosphatase |
| TON_1046 | 967408 | 968217 | - | 4660.28 | 4939.55 | 4756.47 | 1.06 | 1.02 | membrane protein |
| TON_1047 | 968300 | 969331 | + | 16729.55 | 7258.45 | 5624.61 | 0.43 | 0.34 | egsA NAD(P)-dependent glycerol-1-phosphate dehydrogenase |
| TON_1048 | 969344 | 969793 | + | 15982.17 | 23216.71 | 10793.89 | 1.45 | 0.68 | hypothetical protein |
| TON_1049 | 969803 | 970804 | - | 1566.30 | 1704.62 | 849.08 | 1.09 | 0.54 | Allophanate hydrolase, subunit 2 |
| TON_1050 | 970797 | 971483 | - | 304.40 | 316.16 | 153.25 | 1.04 | 0.50 | hypothetical protein |
| TON_1051 | 971488 | 972255 | - | 2284.14 | 1574.79 | 865.21 | 0.69 | 0.38 | LamB/YcsF family protein |
| TON_1052 | 972316 | 972801 | + | 529.29 | 381.07 | 380.55 | 0.72 | 0.72 | hypothetical protein |
| TON_1053 | 972923 | 973318 | - | 2721.43 | 7425.55 | 2553.11 | 2.73 | 0.94 | hypothetical protein |
| TON_1054 | 973346 | 973678 | - | 3142.82 | 3827.58 | 2641.83 | 1.22 | 0.84 | hypothetical protein |
| TON_1055 | 973675 | 974502 | - | 5587.12 | 5808.69 | 5706.74 | 1.04 | 1.02 | 3-methyladenine DNA glycosylase |
| TON_1056 | 974484 | 974783 | - | 2385.23 | 3404.43 | 2473.19 | 1.43 | 1.04 | hypothetical protein |
| TON_1057 | 974875 | 976200 | + | 31731.50 | 30112.12 | 15140.48 | 0.95 | 0.48 | nodulation protein nfeD |
| TON_1058 | 976202 | 977008 | + | 27416.51 | 67509.14 | 23660.63 | 2.46 | 0.86 | membrane protease subunit |
| TON_1059 | 977179 | 977958 | + | 1183.53 | 1949.85 | 886.48 | 1.65 | 0.75 | ATPase |
| TON_1060 | 977955 | 978206 | + | 430.48 | 1026.62 | 359.28 | 2.38 | 0.83 | hypothetical protein |
| TON_1061 | 978297 | 978545 | + | 173.78 | 111.80 | 53.53 | 0.64 | 0.31 | hypothetical protein |
| TON_1062 | 978673 | 980184 | + | 8418.72 | 4807.31 | 22170.70 | 0.57 | 2.63 | thymidine phosphorylase |
| TON_1063 | 980336 | 980725 | - | 12456.58 | 3759.06 | 8559.00 | 0.30 | 0.69 | hypothetical protein |
| TON_1064 | 980787 | 981230 | + | 1928.63 | 552.98 | 1613.84 | 0.29 | 0.84 | hypothetical protein |
| TON_1065 | 981227 | 981994 | + | 3207.57 | 1351.19 | 2050.85 | 0.42 | 0.64 | geranylgeranylglyceryl phosphate synthase |
| TON_1066 | 981997 | 982236 | - | 20008.67 | 13610.50 | 26596.48 | 0.68 | 1.33 | hypothetical protein |
| TON_1067 | 982389 | 983231 | + | 1620.82 | 1551.95 | 510.33 | 0.96 | 0.31 | D-aminopeptidase |
| TON_1068 | 983401 | 984144 | + | 1102.88 | 266.87 | 233.90 | 0.24 | 0.21 | hypothetical protein |
| TON_1069 | 984126 | 986495 | - | 4111.68 | 6449.42 | 5864.38 | 1.57 | 1.43 | Hef nuclease |

| | | | | | | | | | |
|---|---|---|---|---|---|---|---|---|---|
| TON_1070 | 986545 | 986970 | + | 182.87 | 158.68 | 325.55 | 0.87 | 1.78 | hypothetical protein |
| TON_1071 | 986967 | 987629 | - | 4983.99 | 8725.05 | 9087.66 | 1.75 | 1.82 | hypothetical protein |
| TON_1072 | 987635 | 991201 | - | 24170.33 | 41032.26 | 46674.55 | 1.70 | 1.93 | chromosome segregation ATPase |
| TON_1073 | 991250 | 991633 | - | 2210.31 | 2312.90 | 5240.40 | 1.05 | 2.37 | hypothetical protein |
| TON_1074 | 991856 | 992260 | - | 6133.45 | 7377.46 | 10628.92 | 1.20 | 1.73 | methylmalonyl-CoA epimerase |
| TON_1075 | 992257 | 993216 | - | 9907.79 | 13472.26 | 18153.33 | 1.36 | 1.83 | lysine/arginine/ornithine transport system kinase |
| TON_1076 | 993251 | 993691 | - | 1795.74 | 5526.19 | 4788.00 | 3.08 | 2.67 | methylmalony-CoA mutase |
| TON_1077 | 993807 | 994457 | + | 740.56 | 834.28 | 973.00 | 1.13 | 1.31 | hypothetical protein |
| TON_1078 | 998473 | 999027 | - | 304.40 | 183.93 | 404.74 | 0.60 | 1.33 | Maf-like protein |
| TON_1079 | 999169 | 999333 | - | 915.47 | 708.05 | 1085.91 | 0.77 | 1.19 | hypothetical protein |
| TON_1080 | 999396 | 1001204 | - | 19257.89 | 14486.85 | 22613.57 | 0.75 | 1.17 | ATPase |
| TON_1081 | 1001316 | 1002056 | + | 7271.54 | 7658.76 | 8628.66 | 1.05 | 1.19 | ATPase |
| TON_1082 | 1002053 | 1002796 | + | 6148.21 | 5821.91 | 5936.24 | 0.95 | 0.97 | GTPase |
| TON_1083 | 1002918 | 1004360 | + | 429.34 | 459.21 | 538.92 | 1.07 | 1.26 | asparagine synthase |
| TON_1084 | 1004504 | 1005709 | + | 36550.80 | 5005.66 | 3548.84 | 0.14 | 0.10 | hypothetical protein |
| TON_1085 | 1005748 | 1006785 | + | 6297.01 | 526.53 | 994.99 | 0.08 | 0.16 | glycosyltransferase |
| TON_1086 | 1006818 | 1007837 | + | 2765.73 | 828.27 | 1662.23 | 0.30 | 0.60 | putative translation factor |
| TON_1087 | 1007910 | 1009091 | + | 5698.43 | 2944.01 | 6704.66 | 0.52 | 1.18 | hypothetical protein |
| TON_1088 | 1009103 | 1009522 | - | 3347.27 | 2228.75 | 4400.85 | 0.67 | 1.31 | hypothetical protein |
| TON_1089 | 1009604 | 1010308 | + | 6123.22 | 2377.81 | 5070.29 | 0.39 | 0.83 | nucleotidyltransferase |
| TON_1090 | 1010315 | 1011688 | - | 12458.85 | 13418.16 | 21466.80 | 1.08 | 1.72 | seryl-tRNA synthetase |
| TON_1091 | 1011767 | 1012969 | - | 7482.81 | 12419.19 | 9042.94 | 1.66 | 1.21 | molybdenum cofactor biosynthesis protein MoeA |
| TON_1092 | 1013033 | 1013185 | - | 380.50 | 354.63 | 406.94 | 0.93 | 1.07 | hypothetical protein |
| TON_1093 | 1013262 | 1014077 | + | 3466.53 | 1568.78 | 2184.29 | 0.45 | 0.63 | hypothetical protein |
| TON_1094 | 1014074 | 1016590 | + | 24007.90 | 29924.59 | 26123.55 | 1.25 | 1.09 | CDC48/VCP |
| TON_1095 | 1016668 | 1016985 | + | 772.36 | 1490.64 | 798.49 | 1.93 | 1.03 | hypothetical protein |
| TON_1096 | 1016998 | 1017705 | - | 1681.02 | 1616.86 | 376.15 | 0.96 | 0.22 | metallophosphoesterase |
| TON_1097 | 1017809 | 1019194 | - | 14036.51 | 26481.69 | 1903.47 | 1.89 | 0.14 | acetyl-CoA synthetase I subunit alpha |
| TON_1098 | 1019409 | 1019834 | + | 497.49 | 710.46 | 530.13 | 1.43 | 1.07 | hypothetical protein |
| TON_1099 | 1019834 | 1020859 | + | 916.61 | 1067.49 | 992.06 | 1.16 | 1.08 | flap endonuclease-1 |
| TON_1100 | 1020872 | 1021771 | - | 5553.04 | 2400.65 | 7348.44 | 0.43 | 1.32 | tfb transcription initiation factor IIB |
| TON_1101 | 1021810 | 1022091 | - | 1430.00 | 1245.41 | 2563.38 | 0.87 | 1.79 | hypothetical protein |

| | | | | | | | | | |
|---|---|---|---|---|---|---|---|---|---|
| TON_1102 | 1022109 | 1022447 | - | 5893.79 | 3549.89 | 6554.35 | 0.60 | 1.11 | hypothetical protein |
| TON_1103 | 1022525 | 1022977 | - | 11407.08 | 5450.45 | 18499.42 | 0.48 | 1.62 | 30S ribosomal protein S19e |
| TON_1104 | 1022981 | 1023397 | - | 11637.65 | 3750.64 | 13478.98 | 0.32 | 1.16 | RNA-binding protein |
| TON_1105 | 1023584 | 1024183 | + | 5269.09 | 1542.33 | 3990.98 | 0.29 | 0.76 | hypothetical protein |
| TON_1106 | 1024192 | 1024425 | - | 4506.95 | 5379.53 | 10194.84 | 1.19 | 2.26 | rplX 50S ribosomal protein LX |
| TON_1107 | 1024439 | 1025125 | - | 5976.70 | 7396.70 | 13875.66 | 1.24 | 2.32 | translation initiation factor IF-6 |
| TON_1108 | 1025160 | 1025435 | - | 8193.83 | 4162.97 | 12097.58 | 0.51 | 1.48 | 50S ribosomal protein L31e |
| TON_1109 | 1025446 | 1025601 | - | 14636.22 | 7318.56 | 11005.80 | 0.50 | 0.75 | rpl39e 50S ribosomal protein L39e |
| TON_1110 | 1025722 | 1027410 | - | 2376.14 | 8959.46 | 6115.15 | 3.77 | 2.57 | methylmalonyl-CoA mutase, N-terminus of large subunit |
| TON_1111 | 1027556 | 1028413 | + | 31.80 | 45.68 | 51.33 | 1.44 | 1.61 | hypothetical protein |
| TON_1112 | 1028454 | 1028939 | + | 299.86 | 399.11 | 235.37 | 1.33 | 0.78 | mechanosensitive ion channel MscS |
| TON_1113 | 1029039 | 1029530 | + | 1682.15 | 2568.95 | 1439.33 | 1.53 | 0.86 | small heat shock protein |
| TON_1114 | 1029624 | 1032017 | + | 5247.50 | 20824.47 | 6381.31 | 3.97 | 1.22 | CDC48/VCP |
| TON_1115 | 1032108 | 1032611 | + | 319.17 | 661.17 | 354.15 | 2.07 | 1.11 | Transposase ISC1173 |
| TON_1116 | 1032643 | 1032765 | + | 202.18 | 219.99 | 156.91 | 1.09 | 0.78 | hypothetical protein |
| TON_1117 | 1032825 | 1032974 | - | 30.67 | 4.81 | 9.53 | 0.16 | 0.31 | hypothetical protein |
| TON_1118 | 1033168 | 1033617 | - | 35.21 | 36.06 | 15.40 | 1.02 | 0.44 | hypothetical protein |
| TON_1119 | 1033382 | 1033828 | + | 139.71 | 191.14 | 109.25 | 1.37 | 0.78 | metalloprotease |
| TON_1120 | 1033871 | 1034605 | - | 693.99 | 638.33 | 783.82 | 0.92 | 1.13 | putative molybdopterin-guanine dinucleotide biosynthesis protein MobB/FeS domain-containing protein |
| TON_1121 | 1034615 | 1034830 | - | 427.07 | 125.02 | 274.23 | 0.29 | 0.64 | hypothetical protein |
| TON_1122 | 1034893 | 1035720 | + | 4810.21 | 6640.56 | 6041.09 | 1.38 | 1.26 | hypothetical protein |
| TON_1123 | 1035739 | 1036074 | - | 556.55 | 1614.46 | 428.94 | 2.90 | 0.77 | membrane protein |
| TON_1124 | 1036116 | 1036832 | - | 1675.34 | 1017.00 | 1929.13 | 0.61 | 1.15 | hypothetical protein |
| TON_1125 | 1036865 | 1037326 | - | 2559.01 | 764.55 | 2899.19 | 0.30 | 1.13 | hypothetical protein |
| TON_1126 | 1037390 | 1037530 | + | 10.22 | 85.35 | 50.59 | 8.35 | 4.95 | phosphonate matabolism protein PhnP |
| TON_1127 | 1037770 | 1037973 | - | 10.22 | 0.00 | 0.73 | 0.00 | 0.07 | hypothetical protein |
| TON_1128 | 1038013 | 1038576 | + | 2415.90 | 2293.66 | 1835.28 | 0.95 | 0.76 | NAD(P)H-flavin oxidoreductase |
| TON_1129 | 1038640 | 1039023 | - | 137.43 | 58.90 | 90.92 | 0.43 | 0.66 | hypothetical protein |
| TON_1130 | 1039447 | 1039869 | + | 968.86 | 821.05 | 1480.39 | 0.85 | 1.53 | hypothetical protein |
| TON_1131 | 1039862 | 1040512 | + | 1264.17 | 513.31 | 1481.86 | 0.41 | 1.17 | transketolase, N-terminal section |
| TON_1132 | 1040509 | 1041429 | + | 909.79 | 464.02 | 1028.72 | 0.51 | 1.13 | transketolase |

| | | | | | | | | | |
|---|---|---|---|---|---|---|---|---|---|
| TON_1133 | 1041426 | 1042226 | + | 519.07 | 444.79 | 497.86 | 0.86 | 0.96 | 3-deoxy-7-phosphoheptulonate synthase |
| TON_1134 | 1042214 | 1043191 | + | 1056.32 | 1113.17 | 1242.83 | 1.05 | 1.18 | aroB 3-dehydroquinate synthase |
| TON_1135 | 1043188 | 1043835 | + | 287.36 | 571.01 | 593.92 | 1.99 | 2.07 | aroD 3-dehydroquinate dehydratase |
| TON_1136 | 1043825 | 1044652 | + | 249.88 | 284.90 | 372.48 | 1.14 | 1.49 | aroE shikimate 5-dehydrogenase |
| TON_1137 | 1044615 | 1045427 | + | 128.35 | 197.15 | 360.75 | 1.54 | 2.81 | shikimate kinase |
| TON_1138 | 1045434 | 1046642 | + | 199.90 | 115.40 | 258.83 | 0.58 | 1.29 | 3-phosphoshikimate 1-carboxyvinyltransferase |
| TON_1139 | 1046639 | 1047709 | + | 212.40 | 191.14 | 321.89 | 0.90 | 1.52 | chorismate synthase |
| TON_1140 | 1047699 | 1047941 | + | 140.84 | 109.39 | 203.10 | 0.78 | 1.44 | chorismate mutase |
| TON_1141 | 1047925 | 1049019 | + | 246.47 | 593.85 | 526.46 | 2.41 | 2.14 | aromatic aminotransferase |
| TON_1142 | 1048944 | 1049714 | + | 186.28 | 342.61 | 380.55 | 1.84 | 2.04 | prephenate dehydrogenase |
| TON_1143 | 1049954 | 1050397 | - | 346.43 | 265.67 | 629.85 | 0.77 | 1.82 | protein-tyrosine phosphatase |
| TON_1144 | 1050548 | 1051285 | + | 3224.60 | 6610.51 | 3465.25 | 2.05 | 1.07 | hypothetical protein |
| TON_1145 | 1051298 | 1051525 | - | 201.04 | 128.63 | 125.38 | 0.64 | 0.62 | carbon-nitrogen hydrolase |
| TON_1146 | 1051747 | 1051857 | - | 411.17 | 207.97 | 467.80 | 0.51 | 1.14 | membrane protein |
| TON_1147 | 1051854 | 1052552 | - | 2155.79 | 1613.26 | 2476.12 | 0.75 | 1.15 | hypothetical protein |
| TON_1148 | 1052670 | 1053440 | + | 1773.02 | 2168.64 | 1888.80 | 1.22 | 1.07 | hypothetical protein |
| TON_1149 | 1053634 | 1054575 | + | 860.95 | 897.99 | 615.18 | 1.04 | 0.71 | hypothetical protein |
| TON_1150 | 1054591 | 1054743 | + | 13.63 | 21.64 | 22.73 | 1.59 | 1.67 | ribosomal protein-serine acetyltransferase RimL |
| TON_1151 | 1054779 | 1055924 | + | 81.78 | 74.53 | 40.33 | 0.91 | 0.49 | hypothetical protein |
| TON_1152 | 1056098 | 1057888 | + | 1554.94 | 551.78 | 214.10 | 0.35 | 0.14 | hypothetical protein |
| TON_1153 | 1057902 | 1058123 | - | 335.07 | 252.45 | 2213.62 | 0.75 | 6.61 | hypothetical protein |
| TON_1154 | 1058120 | 1058611 | - | 2381.82 | 3099.09 | 13661.56 | 1.30 | 5.74 | hypothetical protein |
| TON_1155 | 1058622 | 1059059 | - | 1436.82 | 2542.50 | 4266.67 | 1.77 | 2.97 | hypothetical protein |
| TON_1156 | 1059085 | 1059693 | - | 563.37 | 510.90 | 519.13 | 0.91 | 0.92 | SAM-dependent methyltransferase |
| TON_1157 | 1059717 | 1060217 | - | 570.18 | 822.26 | 476.60 | 1.44 | 0.84 | hypothetical protein |
| TON_1158 | 1060286 | 1061098 | + | 562.23 | 656.36 | 502.26 | 1.17 | 0.89 | Acetyltransferase (GNAT) family protein |
| TON_1159 | 1061150 | 1061620 | + | 166.97 | 155.07 | 52.79 | 0.93 | 0.32 | hypothetical protein |
| TON_1160 | 1061659 | 1062846 | + | 94.27 | 87.76 | 66.72 | 0.93 | 0.71 | hypothetical protein |
| TON_1161 | 1062858 | 1063442 | + | 74.96 | 70.93 | 101.92 | 0.95 | 1.36 | phosphoserine phosphatase |
| TON_1162 | 1063420 | 1063791 | - | 82.92 | 37.27 | 33.00 | 0.45 | 0.40 | hypothetical protein |
| TON_1163 | 1063778 | 1064185 | - | 584.95 | 224.80 | 292.56 | 0.38 | 0.50 | nucleotidyltransferase |
| TON_1164 | 1064230 | 1064793 | - | 682.63 | 405.12 | 547.72 | 0.59 | 0.80 | nicotinamide-nucleotide adenylyltransferase |

| | | | | | | | | | |
|---|---|---|---|---|---|---|---|---|---|
| TON_1165 | 1064806 | 1065579 | - | 1226.69 | 1307.92 | 1064.65 | 1.07 | 0.87 | hypothetical protein |
| TON_1166 | 1065629 | 1067236 | + | 19840.57 | 11400.99 | 11042.46 | 0.57 | 0.56 | hypothetical protein |
| TON_1167 | 1067233 | 1067772 | + | 13238.02 | 7694.83 | 6060.89 | 0.58 | 0.46 | tRNA 2'-O-methylase |
| TON_1168 | 1067776 | 1068339 | + | 5710.92 | 9326.11 | 3501.18 | 1.63 | 0.61 | CDP-alcohol phosphatidyltransferase |
| TON_1169 | 1068423 | 1069133 | - | 1323.23 | 1061.48 | 1904.93 | 0.80 | 1.44 | hydrolase |
| TON_1170 | 1068651 | 1069574 | + | 17919.89 | 11736.38 | 57139.22 | 0.65 | 3.19 | hypothetical protein |
| TON_1171 | 1069555 | 1070124 | - | 1507.24 | 530.14 | 2397.67 | 0.35 | 1.59 | hypothetical protein |
| TON_1172 | 1070128 | 1070463 | - | 962.04 | 524.13 | 1307.35 | 0.54 | 1.36 | hypothetical protein |
| TON_1173 | 1070557 | 1070724 | + | 905.25 | 2457.15 | 953.20 | 2.71 | 1.05 | hypothetical protein |
| TON_1174 | 1070734 | 1071864 | + | 7111.39 | 7823.45 | 6571.95 | 1.10 | 0.92 | signal peptidase |
| TON_1175 | 1072044 | 1072667 | - | 1107.43 | 917.22 | 1451.80 | 0.83 | 1.31 | phosphoserine phosphatase |
| TON_1176 | 1072706 | 1073368 | - | 81.78 | 73.33 | 63.06 | 0.90 | 0.77 | pcm protein-L-isoaspartate O-methyltransferase |
| TON_1177 | 1073418 | 1075340 | - | 72.69 | 345.01 | 144.45 | 4.75 | 1.99 | membrane protein |
| TON_1178 | 1075349 | 1077079 | - | 42.03 | 120.21 | 31.53 | 2.86 | 0.75 | flagellar assembly protein J |
| TON_1179 | 1077093 | 1078733 | - | 177.19 | 322.17 | 238.30 | 1.82 | 1.34 | flagella-related protein I |
| TON_1180 | 1078735 | 1079433 | - | 52.25 | 58.90 | 40.33 | 1.13 | 0.77 | flagellar accessory protein FlaH |
| TON_1181 | 1079489 | 1079968 | - | 24.99 | 64.91 | 22.73 | 2.60 | 0.91 | flagella-related protein G |
| TON_1182 | 1079968 | 1080444 | - | 19.31 | 38.47 | 26.40 | 1.99 | 1.37 | flagella-related protein |
| TON_1183 | 1080477 | 1081808 | - | 742.83 | 692.43 | 596.85 | 0.93 | 0.80 | flagella-related protein D |
| TON_1184 | 1081812 | 1082288 | - | 152.20 | 219.99 | 189.91 | 1.45 | 1.25 | flagella-related protein C |
| TON_1185 | 1082302 | 1083099 | - | 106.77 | 169.50 | 80.66 | 1.59 | 0.76 | flagellin |
| TON_1186 | 1083110 | 1083775 | - | 115.85 | 152.67 | 99.72 | 1.32 | 0.86 | flagellin |
| TON_1187 | 1083830 | 1084570 | - | 399.81 | 348.62 | 164.24 | 0.87 | 0.41 | flagellin |
| TON_1188 | 1084607 | 1085638 | - | 152.20 | 200.76 | 101.19 | 1.32 | 0.66 | flagellin B |
| TON_1189 | 1085678 | 1086385 | - | 32.94 | 62.51 | 30.80 | 1.90 | 0.93 | flagellin |
| TON_1190 | 1086601 | 1087554 | - | 962.04 | 2118.15 | 1564.71 | 2.20 | 1.63 | SAM-dependent methyltransferase |
| TON_1191 | 1087564 | 1087893 | - | 876.86 | 887.17 | 945.13 | 1.01 | 1.08 | hypothetical protein |
| TON_1192 | 1088246 | 1089325 | + | 7597.52 | 4428.64 | 6237.60 | 0.58 | 0.82 | hypothetical protein |
| TON_1193 | 1089322 | 1089762 | - | 116.99 | 213.98 | 96.05 | 1.83 | 0.82 | purine-binding chemotaxis protein (cheW) |
| TON_1194 | 1089767 | 1090993 | - | 466.82 | 801.82 | 550.66 | 1.72 | 1.18 | Methyl-accepting chemotaxis protein |
| TON_1195 | 1091237 | 1092100 | + | 316.89 | 426.76 | 527.93 | 1.35 | 1.67 | cheR chemotaxis protein methyltransferase |
| TON_1196 | 1092075 | 1092437 | + | 368.01 | 605.87 | 489.07 | 1.65 | 1.33 | chemotaxis response regulator |

| | | | | | | | | | |
|---|---|---|---|---|---|---|---|---|---|
| TON_1197 | 1092449 | 1093561 | + | 899.57 | 1701.01 | 885.74 | 1.89 | 0.98 | chemotaxis-specific methylesterase |
| TON_1198 | 1093533 | 1095878 | + | 1458.40 | 6479.47 | 4134.69 | 4.44 | 2.84 | cheA chemotaxis histidine kinase |
| TON_1199 | 1095868 | 1096500 | + | 272.60 | 1878.93 | 915.07 | 6.89 | 3.36 | histidine kinase |
| TON_1200 | 1096497 | 1097222 | + | 184.00 | 913.62 | 567.52 | 4.97 | 3.08 | chemotaxis protein cheC |
| TON_1201 | 1097226 | 1097714 | + | 143.11 | 1452.17 | 509.60 | 10.15 | 3.56 | chemoreceptor glutamine deamidase CheD |
| TON_1202 | 1097752 | 1099995 | + | 2256.88 | 7645.54 | 4704.41 | 3.39 | 2.08 | methyl-accepting chemotaxis protein |
| TON_1203 | 1100033 | 1100782 | + | 1441.36 | 1692.60 | 1693.03 | 1.17 | 1.17 | hypothetical protein |
| TON_1204 | 1100803 | 1101828 | + | 3221.20 | 1890.95 | 2528.91 | 0.59 | 0.79 | hypothetical protein |
| TON_1205 | 1101851 | 1103134 | - | 1436.82 | 836.68 | 812.42 | 0.58 | 0.57 | membrane protein |
| TON_1206 | 1103204 | 1103950 | + | 796.21 | 552.98 | 274.96 | 0.69 | 0.35 | hypothetical protein |
| TON_1207 | 1103915 | 1104991 | - | 7939.41 | 3311.86 | 5199.34 | 0.42 | 0.65 | hypothetical protein |
| TON_1208 | 1105241 | 1106779 | + | 3397.25 | 2680.75 | 4298.93 | 0.79 | 1.27 | hypothetical protein |
| TON_1209 | 1106784 | 1107188 | + | 246.47 | 210.37 | 300.62 | 0.85 | 1.22 | ribonuclease P protein component 4 |
| TON_1210 | 1107193 | 1108341 | - | 84897.13 | 7694.83 | 6407.70 | 0.09 | 0.08 | hypothetical protein |
| TON_1211 | 1108571 | 1109140 | - | 2739.61 | 1979.91 | 1340.35 | 0.72 | 0.49 | cyclase-related protein |
| TON_1212 | 1109195 | 1110460 | + | 4486.50 | 4550.06 | 6587.35 | 1.01 | 1.47 | S-adenosyl-L-homocysteine hydrolase |
| TON_1213 | 1110628 | 1111566 | + | 964.31 | 346.21 | 1146.04 | 0.36 | 1.19 | polysaccharide deacetylase |
| TON_1214 | 1111608 | 1112267 | + | 3951.53 | 2755.28 | 1239.89 | 0.70 | 0.31 | hypothetical protein |
| TON_1215 | 1112422 | 1112967 | - | 650.83 | 1084.32 | 1088.85 | 1.67 | 1.67 | metal-dependent phosphohydrolase |
| TON_1216 | 1112979 | 1113293 | - | 791.67 | 738.11 | 1055.12 | 0.93 | 1.33 | hypothetical protein |
| TON_1217 | 1113345 | 1113902 | + | 2318.22 | 596.26 | 845.42 | 0.26 | 0.36 | GTP-binding protein |
| TON_1218 | 1113903 | 1114307 | - | 160.15 | 223.60 | 358.55 | 1.40 | 2.24 | hypothetical protein |
| TON_1219 | 1114308 | 1114601 | - | 377.09 | 455.61 | 603.45 | 1.21 | 1.60 | hypothetical protein |
| TON_1220 | 1114580 | 1115863 | - | 2261.42 | 2130.17 | 2755.48 | 0.94 | 1.22 | hypothetical protein |
| TON_1221 | 1115979 | 1117073 | + | 3121.24 | 1530.31 | 5519.76 | 0.49 | 1.77 | DNA methylase |
| TON_1222 | 1117070 | 1117231 | - | 102.22 | 64.91 | 38.13 | 0.64 | 0.37 | CoA-binding protein |
| TON_1223 | 1117568 | 1118005 | + | 1938.85 | 650.35 | 1041.92 | 0.34 | 0.54 | membrane protein |
| TON_1224 | 1117977 | 1118360 | - | 528.16 | 430.36 | 420.87 | 0.81 | 0.80 | Membrane protein |
| TON_1225 | 1118357 | 1119004 | - | 380.50 | 229.61 | 262.50 | 0.60 | 0.69 | putative zinc-dependent protease |
| TON_1226 | 1119042 | 1119803 | + | 256.70 | 103.38 | 67.46 | 0.40 | 0.26 | hypothetical protein |
| TON_1227 | 1119842 | 1120474 | - | 772.36 | 669.59 | 639.38 | 0.87 | 0.83 | hypothetical protein |
| TON_1228 | 1120516 | 1121835 | - | 540.65 | 199.55 | 200.17 | 0.37 | 0.37 | glycerate kinase |

| | | | | | | | | | |
|---|---|---|---|---|---|---|---|---|---|
| TON_1229 | 1122089 | 1122508 | - | 509.98 | 123.82 | 341.69 | 0.24 | 0.67 | hypothetical protein |
| TON_1230 | 1122619 | 1122822 | + | 5086.22 | 2416.28 | 8285.51 | 0.48 | 1.63 | rps17E 30S ribosomal protein S17e |
| TON_1231 | 1122877 | 1123473 | - | 5491.71 | 3261.37 | 3684.49 | 0.59 | 0.67 | heat shock regulator |
| TON_1232 | 1123587 | 1124756 | + | 1621.96 | 1709.43 | 2851.53 | 1.05 | 1.76 | threonine synthase (TS) |
| TON_1233 | 1124753 | 1125331 | - | 1382.30 | 932.85 | 963.47 | 0.67 | 0.70 | thymidine kinase |
| TON_1234 | 1125429 | 1126763 | - | 38488.52 | 25986.41 | 58352.71 | 0.68 | 1.52 | rbcL ribulose bisophosphate carboxylase |
| TON_1235 | 1127155 | 1127358 | + | 2619.21 | 2948.82 | 3856.79 | 1.13 | 1.47 | histone B |
| TON_1236 | 1127387 | 1127881 | - | 373.69 | 640.73 | 766.96 | 1.71 | 2.05 | metallophosphoesterase |
| TON_1237 | 1127891 | 1128802 | - | 6881.95 | 15710.62 | 12742.82 | 2.28 | 1.85 | tfb transcription initiation factor IIB |
| TON_1238 | 1128809 | 1129120 | - | 16856.76 | 21646.73 | 29835.17 | 1.28 | 1.77 | H/ACA RNA-protein complex component Gar1 |
| TON_1239 | 1129244 | 1130083 | + | 1159.68 | 351.02 | 2295.01 | 0.30 | 1.98 | carbohydrate/pyrimidine kinase |
| TON_1240 | 1130184 | 1130693 | + | 1922.95 | 1377.64 | 1069.78 | 0.72 | 0.56 | ADP-ribose pyrophosphatase |
| TON_1241 | 1130698 | 1131051 | + | 1424.32 | 753.73 | 819.02 | 0.53 | 0.58 | hypothetical protein |
| TON_1242 | 1131041 | 1132504 | - | 1216.47 | 1776.75 | 1127.71 | 1.46 | 0.93 | membrane protein |
| TON_1243 | 1132513 | 1133274 | - | 1658.30 | 1703.42 | 1506.06 | 1.03 | 0.91 | ABC transporter |
| TON_1244 | 1133359 | 1134669 | - | 1774.16 | 5404.77 | 1447.40 | 3.05 | 0.82 | putative transposase |
| TON_1245 | 1134666 | 1134845 | - | 1614.01 | 4320.45 | 2255.42 | 2.68 | 1.40 | hypothetical protein |
| TON_1246 | 1135197 | 1135499 | + | 30.67 | 10.82 | 16.13 | 0.35 | 0.53 | hypothetical protein |
| TON_1247 | 1135538 | 1136215 | - | 1218.74 | 435.17 | 1564.71 | 0.36 | 1.28 | ATP-binding protein |
| TON_1248 | 1136309 | 1137586 | + | 2581.73 | 1594.02 | 1004.53 | 0.62 | 0.39 | bifunctional sugar nucleotidyltransferase/CDP-alcohol phosphatidyltransferase synthase |
| TON_1249 | 1137647 | 1138795 | + | 850.73 | 19389.13 | 2861.80 | 22.79 | 3.36 | myo-inositol-1-phosphate synthase |
| TON_1250 | 1138860 | 1139462 | + | 2564.69 | 2151.81 | 1498.72 | 0.84 | 0.58 | hypothetical protein |
| TON_1251 | 1139467 | 1140114 | + | 2608.99 | 1707.02 | 1613.11 | 0.65 | 0.62 | orotidine 5'-phosphate decarboxylase |
| TON_1252 | 1140117 | 1140635 | + | 2223.94 | 1990.72 | 1794.95 | 0.90 | 0.81 | putative RNA-binding protein |
| TON_1253 | 1140637 | 1141089 | + | 1947.94 | 1880.13 | 1731.16 | 0.97 | 0.89 | glycerol-3-phosphate cytidylyltransferase |
| TON_1254 | 1141186 | 1141530 | - | 4501.27 | 5537.00 | 2454.86 | 1.23 | 0.55 | putative transcriptional regulator |
| TON_1255 | 1141517 | 1142287 | - | 1693.51 | 2612.23 | 2010.52 | 1.54 | 1.19 | ATPase |
| TON_1256 | 1142321 | 1143571 | - | 11750.09 | 22449.75 | 12299.22 | 1.91 | 1.05 | cell division protein FtsZ |
| TON_1257 | 1143637 | 1143828 | - | 12563.34 | 11809.71 | 7774.45 | 0.94 | 0.62 | hypothetical protein |
| TON_1258 | 1144038 | 1144445 | + | 2682.82 | 419.54 | 825.62 | 0.16 | 0.31 | hypothetical protein |
| TON_1259 | 1144531 | 1145709 | + | 1966.11 | 1691.39 | 1945.26 | 0.86 | 0.99 | aspartate aminotransferase |

| | | | | | | | | | |
|---|---|---|---|---|---|---|---|---|---|
| TON_1260 | 1145723 | 1145986 | + | 421.39 | 702.04 | 742.03 | 1.67 | 1.76 | membrane protein |
| TON_1261 | 1145999 | 1147204 | + | 313.49 | 724.88 | 485.40 | 2.31 | 1.55 | permease |
| TON_1262 | 1147194 | 1147520 | + | 9.09 | 46.88 | 32.26 | 5.16 | 3.55 | membrane protein |
| TON_1263 | 1148299 | 1149018 | - | 1785.51 | 2165.03 | 2370.54 | 1.21 | 1.33 | putative transcriptional regulator |
| TON_1264 | 1149064 | 1149966 | - | 1186.94 | 572.21 | 559.46 | 0.48 | 0.47 | dihydroorotate dehydrogenase 1B |
| TON_1265 | 1150468 | 1151904 | + | 879.13 | 248.84 | 283.76 | 0.28 | 0.32 | proline dehydrogenase subunit alpha |
| TON_1266 | 1151901 | 1152407 | + | 297.59 | 121.41 | 138.58 | 0.41 | 0.47 | proline dehydrogenase subunit gamma |
| TON_1267 | 1152613 | 1152885 | + | 229.44 | 155.07 | 136.38 | 0.68 | 0.59 | hypothetical protein |
| TON_1268 | 1152878 | 1154044 | + | 739.42 | 427.96 | 313.82 | 0.58 | 0.42 | sarcosine oxidase subunit beta |
| TON_1269 | 1154207 | 1156117 | + | 1364.12 | 992.96 | 942.20 | 0.73 | 0.69 | putative UPF0069 protein |
| TON_1270 | 1156196 | 1156873 | + | 1975.20 | 1220.16 | 1873.40 | 0.62 | 0.95 | pyrH uridylate kinase |
| TON_1271 | 1157017 | 1158336 | + | 3438.14 | 3266.18 | 3722.61 | 0.95 | 1.08 | NADH oxidase |
| TON_1272 | 1158402 | 1158596 | + | 2.27 | 6.01 | 8.80 | 2.65 | 3.87 | membrane-bound metal-dependent hydrolase, N-terminus |
| TON_1273 | 1158620 | 1158922 | + | 3.41 | 4.81 | 1.47 | 1.41 | 0.43 | membrane-bound metal-dependent hydrolase |
| TON_1274 | 1159107 | 1159451 | + | 47.70 | 16.83 | 12.46 | 0.35 | 0.26 | membrane-bound metal-dependent hydrolase |
| TON_1275 | 1159482 | 1159652 | + | 22.72 | 8.41 | 27.86 | 0.37 | 1.23 | membrane-bound metal-dependent hydrolase, C-terminus |
| TON_1276 | 1159737 | 1160900 | + | 168.10 | 435.17 | 310.89 | 2.59 | 1.85 | Heat shock protein/ Zn-dependent protease with chaperone function, family M48 |
| TON_1277 | 1161095 | 1161949 | + | 99.95 | 476.04 | 579.25 | 4.76 | 5.80 | hypothetical protein |
| TON_1278 | 1161987 | 1162529 | + | 534.97 | 177.92 | 428.21 | 0.33 | 0.80 | RNA 2'-phosphotransferase-like protein |
| TON_1279 | 1162513 | 1163751 | - | 87.46 | 97.37 | 177.44 | 1.11 | 2.03 | multidrug-efflux transporter |
| TON_1280 | 1163925 | 1164986 | - | 143.11 | 180.32 | 197.24 | 1.26 | 1.38 | galactokinase |
| TON_1281 | 1165050 | 1166210 | - | 528.16 | 1127.60 | 846.88 | 2.13 | 1.60 | sarcosine oxidase, beta subunit |
| TON_1282 | 1166217 | 1167701 | - | 771.22 | 1918.60 | 1569.85 | 2.49 | 2.04 | sarcosine oxidase, alpha subunit |
| TON_1283 | 1167920 | 1169086 | - | 1693.51 | 1416.11 | 3014.31 | 0.84 | 1.78 | GTPase |
| TON_1284 | 1169205 | 1169657 | + | 874.58 | 2856.26 | 920.20 | 3.27 | 1.05 | transcriptional regulator |
| TON_1285 | 1169734 | 1170234 | + | 1184.66 | 1080.71 | 1530.25 | 0.91 | 1.29 | intracellular protease I |
| TON_1286 | 1170314 | 1171039 | + | 3669.85 | 3427.27 | 3489.45 | 0.93 | 0.95 | hypothetical protein |
| TON_1287 | 1171096 | 1171509 | + | 988.17 | 568.61 | 1059.52 | 0.58 | 1.07 | transcriptional regulator |
| TON_1288 | 1171511 | 1171738 | + | 157.88 | 152.67 | 192.84 | 0.97 | 1.22 | membrane protein |
| TON_1289 | 1171739 | 1172635 | + | 1066.54 | 599.86 | 850.55 | 0.56 | 0.80 | ATP:dephospho-CoA triphosphoribosyl transferase |
| TON_1290 | 1172619 | 1172900 | - | 354.38 | 816.25 | 250.76 | 2.30 | 0.71 | hypothetical protein |

| | | | | | | | | | |
|---|---|---|---|---|---|---|---|---|---|
| TON_1291 | 1172944 | 1173585 | - | 538.38 | 360.64 | 261.76 | 0.67 | 0.49 | small neutral amino acid transporter A |
| TON_1292 | 1173626 | 1174033 | - | 1527.68 | 705.65 | 648.91 | 0.46 | 0.42 | stress-inducible protein |
| TON_1293 | 1174092 | 1174934 | + | 116.99 | 67.32 | 225.84 | 0.58 | 1.93 | hypothetical protein |
| TON_1294 | 1175500 | 1175718 | + | 2.27 | 2.40 | 0.00 | 1.06 | 0.00 | hypothetical protein |
| TON_1295 | 1175757 | 1177013 | - | 8620.90 | 16858.65 | 8125.66 | 1.96 | 0.94 | multiple substrate aminotransferase |
| TON_1296 | 1177252 | 1178220 | + | 10114.51 | 8096.34 | 14636.02 | 0.80 | 1.45 | translation initiation factor IF-2 |
| TON_1297 | 1178275 | 1179057 | - | 544.06 | 938.86 | 449.47 | 1.73 | 0.83 | hypothetical protein |
| TON_1298 | 1179126 | 1180382 | - | 963.18 | 1491.84 | 618.85 | 1.55 | 0.64 | hypothetical protein |
| TON_1299 | 1180681 | 1181943 | + | 7644.09 | 5058.56 | 5765.39 | 0.66 | 0.75 | C/D box methylation guide ribonucleoprotein complex aNOP56 subunit |
| TON_1300 | 1181949 | 1182629 | + | 5757.49 | 2627.85 | 5466.97 | 0.46 | 0.95 | fibrillarin |
| TON_1301 | 1182802 | 1183854 | + | 2386.37 | 8042.24 | 12888.00 | 3.37 | 5.40 | hypothetical protein |
| TON_1302 | 1183865 | 1185025 | + | 1643.54 | 9649.49 | 12830.81 | 5.87 | 7.81 | acetyl-CoA acetyltransferase |
| TON_1303 | 1185027 | 1185431 | + | 2394.32 | 6951.91 | 11334.28 | 2.90 | 4.73 | hypothetical protein |
| TON_1304 | 1185537 | 1185830 | - | 2049.03 | 1108.36 | 1180.50 | 0.54 | 0.58 | hypothetical protein |
| TON_1305 | 1185849 | 1186352 | - | 2522.66 | 739.31 | 948.07 | 0.29 | 0.38 | N-acetyltransferase |
| TON_1306 | 1186423 | 1186956 | + | 5329.28 | 3810.75 | 4155.95 | 0.72 | 0.78 | transcription factor |
| TON_1307 | 1186961 | 1187368 | - | 6097.10 | 5101.83 | 6114.41 | 0.84 | 1.00 | hypothetical protein |
| TON_1308 | 1188031 | 1188366 | + | 414.58 | 94.97 | 140.05 | 0.23 | 0.34 | hypothetical protein |
| TON_1309 | 1188584 | 1189162 | + | 3314.33 | 2891.12 | 4769.67 | 0.87 | 1.44 | transcription factor |
| TON_1310 | 1189183 | 1190190 | + | 2499.95 | 3561.91 | 4941.98 | 1.42 | 1.98 | acetylpolyamine aminohydrolase |
| TON_1311 | 1190205 | 1190813 | - | 3812.96 | 7007.21 | 4780.67 | 1.84 | 1.25 | indolepyruvate oxidoreductase subunit beta |
| TON_1312 | 1190810 | 1192756 | - | 58259.80 | 69605.65 | 49546.61 | 1.19 | 0.85 | indolepyruvate: ferredoxin oxidoreductase subunit alpha |
| TON_1313 | 1192768 | 1194147 | - | 48058.97 | 34865.34 | 35380.59 | 0.73 | 0.74 | acetyl-CoA synthetase II subunit alpha |
| TON_1314 | 1194334 | 1195008 | + | 3949.26 | 6592.47 | 3593.56 | 1.67 | 0.91 | metal-dependent hydrolase |
| TON_1315 | 1195054 | 1196052 | + | 13660.55 | 7325.77 | 11809.42 | 0.54 | 0.86 | putative transcriptional regulator |
| TON_1316 | 1196126 | 1196272 | - | 4763.64 | 1792.37 | 4059.90 | 0.38 | 0.85 | nucleic acid-binding protein |
| TON_1317 | 1196321 | 1197133 | - | 8874.19 | 7140.64 | 5135.55 | 0.80 | 0.58 | hypothetical protein |
| TON_1318 | 1197177 | 1197818 | - | 2162.61 | 1162.46 | 1352.81 | 0.54 | 0.63 | membrane protein |
| TON_1319 | 1197818 | 1198534 | - | 3941.31 | 2834.62 | 2573.64 | 0.72 | 0.65 | amidase |
| TON_1320 | 1198545 | 1199687 | - | 339.61 | 916.02 | 733.23 | 2.70 | 2.16 | glycosyltransferase |
| TON_1321 | 1199688 | 1200476 | - | 101.09 | 193.54 | 139.31 | 1.91 | 1.38 | 4-hydroxybenzoate octaprenyltransferase |

| | | | | | | | | | |
|---|---|---|---|---|---|---|---|---|---|
| TON_1322 | 1200519 | 1201367 | - | 1674.20 | 1037.44 | 1783.22 | 0.62 | 1.07 | spermidine synthase |
| TON_1323 | 1201419 | 1201937 | - | 1014.29 | 816.25 | 599.78 | 0.80 | 0.59 | pyruvoyl-dependent arginine decarboxylase |
| TON_1324 | 1202057 | 1203205 | - | 1333.46 | 1265.84 | 3817.20 | 0.95 | 2.86 | NapA-type sodium/hydrogen antiporter |
| TON_1325 | 1203284 | 1203691 | + | 1582.20 | 1054.27 | 2144.70 | 0.67 | 1.36 | hypothetical protein |
| TON_1326 | 1203674 | 1205176 | - | 427.07 | 2655.50 | 515.46 | 6.22 | 1.21 | putative monovalent cation/H+ antiporter subunit D |
| TON_1327 | 1205173 | 1205430 | - | 34.07 | 176.71 | 25.66 | 5.19 | 0.75 | putative monovalent cation/H+ antiporter subunit C |
| TON_1328 | 1205456 | 1205857 | - | 115.85 | 275.29 | 79.19 | 2.38 | 0.68 | putative monovalent cation/H+ antiporter subunit B |
| TON_1329 | 1205854 | 1206096 | - | 197.63 | 280.10 | 145.91 | 1.42 | 0.74 | multisubunit sodium/hydrogen antiporter subunit |
| TON_1330 | 1206093 | 1206323 | - | 79.51 | 189.94 | 46.93 | 2.39 | 0.59 | multisubunit sodium/hydrogen antiporter, MnhG subunit |
| TON_1331 | 1206304 | 1206618 | - | 124.94 | 215.18 | 67.46 | 1.72 | 0.54 | putative monovalent cation/H+ antiporter subunit G |
| TON_1332 | 1206611 | 1206883 | - | 290.77 | 445.99 | 230.23 | 1.53 | 0.79 | putative monovalent cation/H+ antiporter subunit F |
| TON_1333 | 1206859 | 1207464 | - | 592.90 | 860.72 | 475.87 | 1.45 | 0.80 | putative monovalent cation/H+ antiporter subunit E |
| TON_1334 | 1207661 | 1208065 | + | 971.13 | 1281.47 | 654.78 | 1.32 | 0.67 | glycine cleavage system protein H |
| TON_1335 | 1208143 | 1208481 | + | 1461.80 | 2269.62 | 866.68 | 1.55 | 0.59 | hypothetical protein |
| TON_1336 | 1208759 | 1210210 | - | 3066.72 | 891.98 | 1439.33 | 0.29 | 0.47 | putative oxidoreductase |
| TON_1337 | 1210210 | 1211076 | - | 1942.26 | 577.02 | 813.15 | 0.30 | 0.42 | ferredoxin-NADP(+) reductase subunit alpha |
| TON_1338 | 1211242 | 1211454 | + | 213.53 | 213.98 | 217.04 | 1.00 | 1.02 | membrane protein |
| TON_1339 | 1211441 | 1212202 | - | 1868.43 | 932.85 | 1442.27 | 0.50 | 0.77 | tRNA (1-methyladenosine) methyltransferase |
| TON_1340 | 1212183 | 1212821 | - | 7254.51 | 2098.92 | 2081.64 | 0.29 | 0.29 | hypothetical protein |
| TON_1341 | 1212879 | 1213223 | + | 7157.96 | 6355.65 | 4499.10 | 0.89 | 0.63 | signal recognition particle protein Srp19 |
| TON_1342 | 1213224 | 1213469 | - | 9953.22 | 29144.41 | 20801.03 | 2.93 | 2.09 | putative transcriptional regulator |
| TON_1343 | 1213608 | 1215788 | + | 5841.54 | 3779.49 | 15333.32 | 0.65 | 2.62 | ski2-like helicase |
| TON_1344 | 1215836 | 1216393 | + | 2627.16 | 2341.75 | 7048.55 | 0.89 | 2.68 | hypothetical protein |
| TON_1345 | 1216379 | 1217188 | - | 942.73 | 1477.42 | 330.69 | 1.57 | 0.35 | heavy-metal cation transporter |
| TON_1346 | 1217259 | 1217666 | + | 1065.40 | 542.16 | 887.21 | 0.51 | 0.83 | hypothetical protein |
| TON_1347 | 1217677 | 1218954 | - | 798.48 | 922.03 | 380.55 | 1.15 | 0.48 | ABC-type sodium efflux pump system permease |
| TON_1348 | 1218951 | 1219709 | - | 364.60 | 385.88 | 167.18 | 1.06 | 0.46 | ABC-type sodium efflux pump system, ATPase component |
| TON_1349 | 1219865 | 1220071 | - | 56.79 | 68.52 | 19.80 | 1.21 | 0.35 | putative transcriptional regulator |
| TON_1350 | 1220083 | 1220382 | - | 180.60 | 168.30 | 35.93 | 0.93 | 0.20 | hypothetical protein |
| TON_1351 | 1220383 | 1220751 | - | 208.99 | 128.63 | 57.19 | 0.62 | 0.27 | hypothetical protein |
| TON_1352 | 1220752 | 1221360 | - | 341.88 | 175.51 | 63.79 | 0.51 | 0.19 | hypothetical protein |

| | | | | | | | | | |
|---|---|---|---|---|---|---|---|---|---|
| TON_1353 | 1222015 | 1223142 | + | 3627.82 | 2484.80 | 6296.99 | 0.68 | 1.74 | cmo tungsten-containing aldehyde ferredoxin oxidoreductase cofactor modifying protein |
| TON_1354 | 1223352 | 1225169 | + | 53766.48 | 8688.99 | 8029.61 | 0.16 | 0.15 | aor-2 tungsten-containing aldehyde ferredoxin oxidoreductase |
| TON_1355 | 1225480 | 1227450 | + | 233.98 | 106.99 | 110.72 | 0.46 | 0.47 | subtilisin-like serine protease |
| TON_1356 | 1227491 | 1229251 | + | 1202.84 | 595.05 | 1457.66 | 0.49 | 1.21 | membrane protein |
| TON_1357 | 1229253 | 1229924 | - | 809.84 | 763.35 | 1100.58 | 0.94 | 1.36 | hypothetical protein |
| TON_1358 | 1229921 | 1230973 | - | 5512.15 | 3399.62 | 4963.24 | 0.62 | 0.90 | hypothetical protein |
| TON_1359 | 1231033 | 1231824 | + | 992.71 | 780.18 | 2098.51 | 0.79 | 2.11 | hypothetical protein |
| TON_1360 | 1231888 | 1232418 | + | 1169.90 | 1893.35 | 1016.99 | 1.62 | 0.87 | hypothetical protein |
| TON_1361 | 1232550 | 1232753 | + | 4280.92 | 14885.96 | 5901.04 | 3.48 | 1.38 | 4Fe-4S ferredoxin |
| TON_1362 | 1232938 | 1234110 | - | 11706.93 | 7363.04 | 7899.83 | 0.63 | 0.67 | nicotinate phosphoribosyltransferase |
| TON_1363 | 1234414 | 1235316 | - | 1840.03 | 670.79 | 710.50 | 0.36 | 0.39 | hypothetical protein |
| TON_1364 | 1235368 | 1235697 | + | 582.68 | 1675.77 | 717.83 | 2.88 | 1.23 | hypothetical protein |
| TON_1365 | 1235654 | 1236562 | - | 682.63 | 393.10 | 366.62 | 0.58 | 0.54 | UDP-N-acetylglucosamine--dolichyl-phosphate N-acetylglucosaminephosphotransferase |
| TON_1366 | 1236618 | 1237940 | + | 2773.68 | 3287.82 | 2848.60 | 1.19 | 1.03 | bifunctional phosphatase/dolichol-phosphate glucosyltransferase |
| TON_1367 | 1237946 | 1238311 | + | 240.79 | 1258.63 | 394.48 | 5.23 | 1.64 | hypothetical protein |
| TON_1368 | 1238308 | 1238853 | - | 4236.62 | 9555.72 | 2242.95 | 2.26 | 0.53 | deoxycytidylate deaminase |
| TON_1369 | 1238853 | 1239635 | - | 1702.60 | 3287.82 | 1213.50 | 1.93 | 0.71 | zinc-dependent protease |
| TON_1370 | 1239642 | 1240007 | - | 1333.46 | 1443.76 | 526.46 | 1.08 | 0.39 | putative transcriptional regulator |
| TON_1371 | 1240483 | 1240587 | + | 20.44 | 25.24 | 0.00 | 1.23 | 0.00 | hypothetical protein |
| TON_1372 | 1240974 | 1241768 | - | 1525.41 | 687.62 | 1076.38 | 0.45 | 0.71 | diphthine synthase |
| TON_1373 | 1242126 | 1242857 | + | 18536.64 | 8434.13 | 20787.83 | 0.45 | 1.12 | putative transcriptional regulator |
| TON_1374 | 1242868 | 1243653 | - | 3839.08 | 1505.06 | 1409.27 | 0.39 | 0.37 | hypothetical protein |
| TON_1375 | 1243858 | 1244706 | + | 3513.10 | 1594.02 | 871.08 | 0.45 | 0.25 | ferredoxin-NADP(+) reductase subunit alpha |
| TON_1376 | 1244707 | 1246155 | + | 3793.65 | 1601.24 | 1212.03 | 0.42 | 0.32 | putative oxidoreductase |
| TON_1377 | 1246171 | 1246635 | - | 1959.30 | 768.16 | 2558.24 | 0.39 | 1.31 | hypothetical protein |
| TON_1378 | 1246841 | 1247944 | + | 742.83 | 373.86 | 309.42 | 0.50 | 0.42 | putative tyrosine recombinase |
| TON_1379 | 1248192 | 1250594 | + | 2329.57 | 1250.21 | 1047.79 | 0.54 | 0.45 | hypothetical protein |
| TON_1380 | 1250591 | 1252192 | + | 2892.94 | 1233.38 | 1113.78 | 0.43 | 0.38 | UvrD/REP helicase |
| TON_1381 | 1254314 | 1254889 | - | 128.35 | 73.33 | 55.73 | 0.57 | 0.43 | hypothetical protein |
| TON_1382 | 1255554 | 1256738 | + | 630.38 | 476.04 | 806.55 | 0.76 | 1.28 | Type II restriction enzyme AccI |

| | | | | | | | | | |
|---|---|---|---|---|---|---|---|---|---|
| TON_1383 | 1256859 | 1258358 | + | 1281.21 | 1648.12 | 2218.02 | 1.29 | 1.73 | Adenine-specific DNA methylase |
| TON_1384 | 1258589 | 1259065 | - | 1507.24 | 3377.98 | 1696.70 | 2.24 | 1.13 | hypothetical protein |
| TON_1385 | 1259299 | 1260495 | + | 22760.77 | 11209.85 | 19751.04 | 0.49 | 0.87 | proteasome-activating nucleotidase |
| TON_1386 | 1260482 | 1261609 | - | 257.83 | 119.01 | 76.26 | 0.46 | 0.30 | permease |
| TON_1387 | 1261693 | 1262244 | + | 1710.55 | 1109.56 | 1159.24 | 0.65 | 0.68 | hypothetical protein |
| TON_1388 | 1262287 | 1263222 | + | 5238.42 | 3482.57 | 8498.15 | 0.66 | 1.62 | serine/threonine protein kinase |
| TON_1389 | 1263212 | 1263703 | + | 3954.94 | 2758.89 | 6176.74 | 0.70 | 1.56 | hypothetical protein |
| TON_1390 | 1263693 | 1264640 | - | 1852.53 | 1270.65 | 675.31 | 0.69 | 0.36 | biotin synthase |
| TON_1391 | 1264643 | 1265827 | - | 897.30 | 665.98 | 332.89 | 0.74 | 0.37 | hypothetical protein |
| TON_1392 | 1265828 | 1266748 | - | 1922.95 | 728.49 | 479.53 | 0.38 | 0.25 | L-asparaginase |
| TON_1393 | 1266815 | 1267729 | - | 1788.92 | 357.03 | 395.21 | 0.20 | 0.22 | transcriptional regulator |
| TON_1394 | 1267819 | 1268985 | + | 56.79 | 43.28 | 35.93 | 0.76 | 0.63 | permease |
| TON_1395 | 1268989 | 1269570 | - | 1548.13 | 925.64 | 582.92 | 0.60 | 0.38 | indolepyruvate oxidoreductase subunit B |
| TON_1396 | 1269706 | 1270392 | + | 9342.15 | 4126.91 | 5527.09 | 0.44 | 0.59 | SAM-dependent methyltransferase |
| TON_1397 | 1270398 | 1271978 | - | 12991.55 | 6354.45 | 7301.51 | 0.49 | 0.56 | lysK lysyl-tRNA synthetase |
| TON_1398 | 1272111 | 1272986 | + | 973.40 | 378.67 | 387.15 | 0.39 | 0.40 | PhoI-related type II restriction endonuclease |
| TON_1399 | 1273057 | 1273665 | + | 1442.50 | 2560.53 | 4592.96 | 1.78 | 3.18 | membrane protein |
| TON_1400 | 1273662 | 1274228 | - | 22396.17 | 4144.94 | 6643.07 | 0.19 | 0.30 | hypothetical protein |
| TON_1401 | 1274306 | 1274461 | - | 9330.79 | 1424.52 | 2812.67 | 0.15 | 0.30 | hypothetical protein |
| TON_1402 | 1279974 | 1280240 | - | 2.27 | 7.21 | 0.00 | 3.18 | 0.00 | hypothetical protein |
| TON_1403 | 1280533 | 1281372 | - | 1601.51 | 1993.13 | 1725.29 | 1.24 | 1.08 | ribose-phosphate pyrophosphokinase |
| TON_1404 | 1281439 | 1282143 | - | 3237.10 | 4322.85 | 3303.94 | 1.34 | 1.02 | membrane protein |
| TON_1405 | 1282133 | 1282516 | - | 42405.97 | 14080.53 | 22089.31 | 0.33 | 0.52 | hypothetical protein |
| TON_1406 | 1282616 | 1283251 | + | 1691.24 | 791.00 | 1148.24 | 0.47 | 0.68 | hydrolase |
| TON_1407 | 1283263 | 1283937 | + | 651.96 | 216.38 | 477.33 | 0.33 | 0.73 | radB DNA repair and recombination protein RadB |
| TON_1408 | 1284009 | 1285424 | - | 7226.11 | 9329.72 | 9622.92 | 1.29 | 1.33 | RNA-binding protein FAU-1 |
| TON_1409 | 1285390 | 1286136 | - | 4268.42 | 3486.17 | 3998.31 | 0.82 | 0.94 | hypothetical protein |
| TON_1410 | 1286264 | 1287190 | + | 2788.45 | 17428.46 | 9679.38 | 6.25 | 3.47 | moaA molybdenum cofactor biosynthesis protein A |
| TON_1411 | 1287229 | 1288176 | + | 7505.52 | 9490.81 | 5628.28 | 1.26 | 0.75 | hypothetical protein |
| TON_1412 | 1288112 | 1289125 | + | 2721.43 | 4081.23 | 3048.77 | 1.50 | 1.12 | Met-10+ like protein |
| TON_1413 | 1289130 | 1290632 | - | 5247.50 | 6692.25 | 4848.12 | 1.28 | 0.92 | replication factor C large subunit |
| TON_1414 | 1290632 | 1291612 | - | 4670.51 | 5565.86 | 3023.11 | 1.19 | 0.65 | DNA replication ATPase |

| | | | | | | | | | |
|---|---|---|---|---|---|---|---|---|---|
| TON_1415 | 1291759 | 1292946 | + | 13108.54 | 18316.83 | 7449.63 | 1.40 | 0.57 | 2-amino-3-ketobutyrate coenzyme A ligase |
| TON_1416 | 1292970 | 1293365 | - | 185.14 | 298.13 | 193.57 | 1.61 | 1.05 | adenylyl cyclase class-3/4/guanylyl cyclase |
| TON_1417 | 1293365 | 1293880 | - | 3950.39 | 1684.18 | 2678.49 | 0.43 | 0.68 | tRNA-splicing endonuclease subunit alpha |
| TON_1418 | 1293877 | 1294380 | - | 4719.35 | 2143.40 | 3449.85 | 0.45 | 0.73 | ribosomal protein-alanine acetyltransferase |
| TON_1419 | 1294418 | 1295986 | - | 194.23 | 401.51 | 333.62 | 2.07 | 1.72 | protein cytosolic protein |
| TON_1420 | 1296243 | 1298003 | + | 9527.29 | 7533.74 | 11720.70 | 0.79 | 1.23 | bipolar DNA helicase |
| TON_1421 | 1298013 | 1299383 | + | 4981.72 | 4309.63 | 5738.27 | 0.87 | 1.15 | DNA repair exonuclease |
| TON_1422 | 1299380 | 1302034 | + | 5148.69 | 9619.43 | 10850.35 | 1.87 | 2.11 | chromosome segregation protein |
| TON_1423 | 1302027 | 1303361 | + | 1959.30 | 3147.17 | 3028.98 | 1.61 | 1.55 | 5'-3' nuclease |
| TON_1424 | 1303402 | 1304418 | + | 3209.84 | 1301.91 | 1410.74 | 0.41 | 0.44 | ABC-type iron(III)-siderophore transport system permease |
| TON_1425 | 1304418 | 1305140 | + | 4893.13 | 4021.12 | 2084.58 | 0.82 | 0.43 | ABC-type iron(III)-siderophore transport system, ATPase component |
| TON_1426 | 1305176 | 1305778 | + | 20721.96 | 36514.66 | 13091.84 | 1.76 | 0.63 | proteasome subunit beta 2 |
| TON_1427 | 1305818 | 1306906 | + | 21906.63 | 8551.94 | 17085.75 | 0.39 | 0.78 | hypothetical protein |
| TON_1428 | 1306914 | 1308047 | + | 6210.68 | 5062.16 | 6360.78 | 0.82 | 1.02 | threonine synthase |
| TON_1429 | 1308031 | 1308303 | - | 27.26 | 39.67 | 12.46 | 1.46 | 0.46 | membrane protein |
| TON_1430 | 1308426 | 1309214 | + | 589.49 | 131.03 | 85.05 | 0.22 | 0.14 | hypothetical protein |
| TON_1431 | 1309274 | 1309729 | - | 620.16 | 431.56 | 874.01 | 0.70 | 1.41 | alanyl-tRNA synthetase |
| TON_1432 | 1309938 | 1311284 | + | 4209.36 | 2476.38 | 1303.68 | 0.59 | 0.31 | hypothetical protein |
| TON_1433 | 1311374 | 1311616 | + | 823.47 | 4036.75 | 612.25 | 4.90 | 0.74 | hypothetical protein |
| TON_1434 | 1311808 | 1312221 | - | 244.20 | 241.63 | 132.71 | 0.99 | 0.54 | hypothetical protein |
| TON_1435 | 1312208 | 1312501 | - | 115.85 | 171.90 | 65.26 | 1.48 | 0.56 | hypothetical protein |
| TON_1436 | 1312498 | 1313388 | - | 93.14 | 153.87 | 87.99 | 1.65 | 0.94 | putative transcriptional regulator |
| TON_1437 | 1313508 | 1314440 | + | 1487.93 | 328.18 | 457.54 | 0.22 | 0.31 | pyrB aspartate carbamoyltransferase catalytic subunit |
| TON_1438 | 1314443 | 1314898 | + | 858.68 | 286.11 | 228.77 | 0.33 | 0.27 | aspartate carbamoyltransferase regulatory subunit |
| TON_1439 | 1314946 | 1316508 | + | 1621.96 | 919.63 | 1974.59 | 0.57 | 1.22 | hypothetical protein |
| TON_1440 | 1316485 | 1317078 | - | 90.87 | 554.18 | 208.24 | 6.10 | 2.29 | membrane protein |
| TON_1441 | 1317212 | 1318012 | + | 502.03 | 525.33 | 549.19 | 1.05 | 1.09 | hypothetical protein |
| TON_1442 | 1318009 | 1318581 | + | 885.94 | 782.59 | 1099.11 | 0.88 | 1.24 | hypothetical protein |
| TON_1443 | 1318605 | 1320347 | + | 1484.52 | 1652.93 | 1363.08 | 1.11 | 0.92 | TPR repeat protein |
| TON_1444 | 1320904 | 1321479 | + | 374.82 | 421.95 | 307.22 | 1.13 | 0.82 | hypothetical protein |
| TON_1445 | 1321476 | 1322825 | - | 909.79 | 2358.58 | 1651.97 | 2.59 | 1.82 | glmM phosphoglucosamine mutase |

| | | | | | | | | | |
|---|---|---|---|---|---|---|---|---|---|
| TON_1446 | 1322884 | 1323267 | - | 2096.73 | 2981.28 | 4017.37 | 1.42 | 1.92 | hypothetical protein |
| TON_1447 | 1323272 | 1324975 | - | 4046.94 | 6142.88 | 7295.65 | 1.52 | 1.80 | hypothetical protein |
| TON_1448 | 1325015 | 1325407 | - | 7410.11 | 2840.63 | 3004.05 | 0.38 | 0.41 | hypothetical protein |
| TON_1449 | 1325659 | 1326444 | - | 338.48 | 508.50 | 357.82 | 1.50 | 1.06 | hypothetical protein |
| TON_1450 | 1326680 | 1327255 | + | 640.60 | 620.30 | 622.51 | 0.97 | 0.97 | hypothetical protein |
| TON_1451 | 1327289 | 1329445 | - | 205.58 | 227.20 | 413.54 | 1.11 | 2.01 | cyclomaltodextrin glucanotransferase |
| TON_1452 | 1329486 | 1330148 | - | 101.09 | 241.63 | 217.04 | 2.39 | 2.15 | hypothetical protein |
| TON_1453 | 1330213 | 1331508 | - | 1656.03 | 1614.46 | 1745.09 | 0.97 | 1.05 | zinc-dependent protease |
| TON_1454 | 1331505 | 1332866 | - | 5499.66 | 2356.17 | 3762.21 | 0.43 | 0.68 | zinc-dependent protease |
| TON_1455 | 1332949 | 1333458 | + | 8637.94 | 6177.74 | 13123.37 | 0.72 | 1.52 | hypothetical protein |
| TON_1456 | 1333455 | 1333709 | + | 1243.73 | 1111.97 | 2101.44 | 0.89 | 1.69 | hypothetical protein |
| TON_1457 | 1333706 | 1334155 | - | 1527.68 | 3023.35 | 1738.49 | 1.98 | 1.14 | universal stress protein |
| TON_1458 | 1334159 | 1335436 | - | 1418.64 | 1896.96 | 1495.79 | 1.34 | 1.05 | arsenical pump membrane protein |
| TON_1459 | 1335446 | 1335991 | - | 2430.66 | 1454.58 | 2874.27 | 0.60 | 1.18 | hypothetical protein |
| TON_1460 | 1336109 | 1338025 | + | 20039.33 | 18205.04 | 16839.38 | 0.91 | 0.84 | serine protease |
| TON_1461 | 1338020 | 1339297 | - | 1213.06 | 807.83 | 1416.60 | 0.67 | 1.17 | hypothetical protein |
| TON_1462 | 1339388 | 1340494 | - | 2095.59 | 1319.94 | 1370.41 | 0.63 | 0.65 | ArgE/DapE-related deacylase |
| TON_1463 | 1341022 | 1341945 | - | 11818.24 | 7746.52 | 6997.22 | 0.66 | 0.59 | D-3-phosphoglycerate dehydrogenase |
| TON_1464 | 1341942 | 1342589 | - | 12714.41 | 8751.50 | 8028.15 | 0.69 | 0.63 | phosphate transport system regulator PhoU |
| TON_1465 | 1342624 | 1343523 | - | 11171.96 | 6658.59 | 8451.95 | 0.60 | 0.76 | 2-dehydropantoate 2-reductase |
| TON_1466 | 1343569 | 1344288 | + | 674.68 | 546.97 | 1219.36 | 0.81 | 1.81 | putative tRNA/rRNA methyltransferase |
| TON_1467 | 1344275 | 1344814 | + | 1927.49 | 1745.49 | 1954.06 | 0.91 | 1.01 | methylated-DNA--protein-cysteine methyltransferase |
| TON_1468 | 1344790 | 1345827 | + | 16643.22 | 28741.69 | 19602.93 | 1.73 | 1.18 | membrane protein |
| TON_1469 | 1345861 | 1346340 | + | 80.64 | 114.20 | 65.26 | 1.42 | 0.81 | hypothetical protein |
| TON_1470 | 1346347 | 1347513 | + | 56.79 | 76.94 | 28.60 | 1.35 | 0.50 | NapA-type sodium/hydrogen antiporter |
| TON_1471 | 1347538 | 1348290 | - | 1506.10 | 373.86 | 896.74 | 0.25 | 0.60 | hypothetical protein |
| TON_1472 | 1348397 | 1348732 | + | 2095.59 | 809.03 | 2597.84 | 0.39 | 1.24 | nac nascent polypeptide-associated complex protein |
| TON_1473 | 1348773 | 1349132 | - | 1643.54 | 605.87 | 222.17 | 0.37 | 0.14 | carboxymuconolactone decarboxylase-related protein |
| TON_1474 | 1349239 | 1350528 | + | 7420.34 | 2296.07 | 4804.86 | 0.31 | 0.65 | HflX-related GTP-binding protein |
| TON_1475 | 1350508 | 1351470 | - | 82.92 | 1626.48 | 3695.48 | 19.62 | 44.57 | sodium/phosphate symporter |
| TON_1476 | 1351697 | 1352257 | + | 13645.78 | 38131.52 | 29776.51 | 2.79 | 2.18 | pyruvate/ketoisovalerate ferredoxin oxidoreductase subunit gamma |

| | | | | | | | | | |
|---|---|---|---|---|---|---|---|---|---|
| TON_1477 | 1352305 | 1352613 | + | 7966.67 | 17135.14 | 18169.46 | 2.15 | 2.28 | vorD 2-ketoisovalerate ferredoxin oxidoreductase subunit delta |
| TON_1478 | 1352624 | 1353811 | + | 20879.84 | 103667.96 | 72377.22 | 4.96 | 3.47 | vorA 2-ketoisovalerate ferredoxin oxidoreductase subunit alpha |
| TON_1479 | 1353817 | 1354752 | + | 4728.43 | 28518.10 | 20656.58 | 6.03 | 4.37 | 2-ketoisovalerate ferredoxin oxidoreductase subunit beta |
| TON_1480 | 1354815 | 1355132 | + | 5208.89 | 37366.97 | 19846.36 | 7.17 | 3.81 | porD pyuvate ferredoxin oxidoreductase subunit delta |
| TON_1481 | 1355144 | 1356328 | + | 14315.92 | 79211.86 | 75788.21 | 5.53 | 5.29 | porA pyruvate ferredoxin oxidoreductase subunit alpha |
| TON_1482 | 1356339 | 1357334 | + | 3951.53 | 31969.41 | 21297.42 | 8.09 | 5.39 | pyruvate ferredoxin oxidoreductase subunit beta |
| TON_1483 | 1357465 | 1358382 | + | 30810.35 | 14479.64 | 8395.49 | 0.47 | 0.27 | ATPase |
| TON_1484 | 1358513 | 1359877 | + | 429.34 | 353.43 | 215.57 | 0.82 | 0.50 | membrane protein |
| TON_1485 | 1359861 | 1360595 | - | 9735.14 | 2168.64 | 4524.03 | 0.22 | 0.46 | hypothetical protein |
| TON_1486 | 1360637 | 1361173 | - | 53298.52 | 14724.87 | 46636.42 | 0.28 | 0.88 | transcription initiation factor E subunit alpha |
| TON_1487 | 1361316 | 1361600 | - | 1876.38 | 1220.16 | 1523.65 | 0.65 | 0.81 | hypothetical protein |
| TON_1488 | 1361712 | 1362605 | - | 3356.36 | 2140.99 | 8597.13 | 0.64 | 2.56 | carbohydrate/pyrimidine kinase |
| TON_1489 | 1362747 | 1363340 | + | 4362.70 | 1606.04 | 3545.90 | 0.37 | 0.81 | hypothetical protein |
| TON_1490 | 1363330 | 1363605 | - | 467.96 | 295.72 | 371.01 | 0.63 | 0.79 | acylphosphatase |
| TON_1491 | 1363679 | 1363993 | + | 3182.58 | 1030.22 | 1473.06 | 0.32 | 0.46 | divalent cation tolerance protein |
| TON_1492 | 1363966 | 1364574 | + | 1614.01 | 891.98 | 556.52 | 0.55 | 0.34 | hypothetical protein |
| TON_1493 | 1364668 | 1365504 | - | 180.60 | 174.31 | 137.11 | 0.97 | 0.76 | universal stress protein |
| TON_1494 | 1365607 | 1366803 | + | 3676.66 | 4809.72 | 3368.46 | 1.31 | 0.92 | gcvT glycine cleavage system aminomethyltransferase T |
| TON_1495 | 1366836 | 1367663 | + | 663.32 | 168.30 | 156.18 | 0.25 | 0.24 | permease |
| TON_1496 | 1368053 | 1368289 | + | 4441.07 | 7292.11 | 4018.84 | 1.64 | 0.90 | hypothetical protein |
| TON_1497 | 1368397 | 1369524 | + | 1482.25 | 12220.84 | 1362.34 | 8.24 | 0.92 | thermophile-specific fructose-1,6-bisphosphatase |
| TON_1498 | 1369681 | 1371642 | + | 6024.41 | 5151.12 | 15339.92 | 0.86 | 2.55 | tungsten-containing glyceraldehyde-3-phosphate:ferredoxin oxidoreductase |
| TON_1499 | 1371745 | 1373418 | - | 207.86 | 162.29 | 197.97 | 0.78 | 0.95 | hypothetical protein |
| TON_1500 | 1373454 | 1374305 | - | 115.85 | 348.62 | 368.08 | 3.01 | 3.18 | hypothetical protein |
| TON_1501 | 1374537 | 1375799 | + | 2119.45 | 997.77 | 1596.24 | 0.47 | 0.75 | oxidoreductase |
| TON_1502 | 1375920 | 1376867 | + | 475.91 | 209.17 | 249.30 | 0.44 | 0.52 | carbamate kinase-like carbamoyl phosphate synthetase |
| TON_1503 | 1376915 | 1377757 | + | 4566.01 | 5302.59 | 1949.66 | 1.16 | 0.43 | hypothetical protein |
| TON_1504 | 1377906 | 1378808 | + | 28422.85 | 16050.82 | 21916.27 | 0.56 | 0.77 | hypothetical protein |
| TON_1505 | 1378818 | 1380524 | + | 3580.12 | 6790.82 | 5991.23 | 1.90 | 1.67 | RNA-binding protein |
| TON_1506 | 1380525 | 1382825 | - | 1067.67 | 1572.38 | 1196.63 | 1.47 | 1.12 | hypothetical protein |

| | | | | | | | | | |
|---|---|---|---|---|---|---|---|---|---|
| TON_1507 | 1382924 | 1383220 | + | 715.57 | 376.27 | 725.90 | 0.53 | 1.01 | hypothetical protein |
| TON_1508 | 1383799 | 1384434 | - | 2052.43 | 904.00 | 1261.89 | 0.44 | 0.61 | hypothetical protein |
| TON_1509 | 1384238 | 1385170 | + | 751.92 | 670.79 | 322.62 | 0.89 | 0.43 | ubiE ubiquinone/menaquinone biosynthesis methyltransferase |
| TON_1510 | 1385875 | 1386300 | - | 14110.34 | 23758.87 | 7964.35 | 1.68 | 0.56 | transcriptional regulator |
| TON_1511 | 1386364 | 1387623 | - | 6763.83 | 2921.17 | 4514.50 | 0.43 | 0.67 | 23S rRNA (uracil-5-)-methyltransferase |
| TON_1512 | 1387731 | 1387946 | + | 4230.94 | 6610.51 | 1629.97 | 1.56 | 0.39 | hypothetical protein |
| TON_1513 | 1388778 | 1389335 | - | 547.47 | 221.19 | 286.69 | 0.40 | 0.52 | pyrE orotate phosphoribosyltransferase |
| TON_1514 | 1389443 | 1390150 | + | 472.50 | 131.03 | 309.42 | 0.28 | 0.65 | membrane protein |
| TON_1515 | 1390140 | 1391828 | - | 6481.01 | 5410.78 | 6882.11 | 0.83 | 1.06 | ATP-dependent DNA ligase |
| TON_1516 | 1391869 | 1392771 | - | 1470.89 | 1023.01 | 1797.15 | 0.70 | 1.22 | GHMP kinase |
| TON_1517 | 1392845 | 1394503 | - | 170.37 | 1023.01 | 731.03 | 6.00 | 4.29 | NhaC-type sodium/hydrogen antiporter |
| TON_1518 | 1394814 | 1395401 | - | 2741.88 | 1753.91 | 3952.85 | 0.64 | 1.44 | uracil-DNA glycosylase |
| TON_1519 | 1395411 | 1395770 | - | 4591.00 | 1898.16 | 4409.65 | 0.41 | 0.96 | hypothetical protein |
| TON_1520 | 1395932 | 1397704 | + | 103014.65 | 62350.80 | 14833.99 | 0.61 | 0.14 | hypothetical protein |
| TON_1521 | 1397715 | 1398020 | + | 48022.62 | 33389.12 | 8124.20 | 0.70 | 0.17 | hypothetical protein |
| TON_1522 | 1398035 | 1399483 | - | 1233.50 | 839.09 | 802.15 | 0.68 | 0.65 | hypothetical protein |
| TON_1523 | 1399564 | 1399917 | + | 887.08 | 395.50 | 618.85 | 0.45 | 0.70 | effector of murein hydrolase |
| TON_1524 | 1399914 | 1400588 | + | 817.79 | 284.90 | 612.25 | 0.35 | 0.75 | effector of murein hydrolase |
| TON_1525 | 1400553 | 1400993 | - | 27368.80 | 3985.06 | 7666.66 | 0.15 | 0.28 | tfx putative transcriptional regulator |
| TON_1526 | 1401283 | 1401864 | + | 1826.40 | 1428.13 | 3162.42 | 0.78 | 1.73 | hypothetical protein |
| TON_1527 | 1401861 | 1402418 | + | 2739.61 | 1565.17 | 5523.43 | 0.57 | 2.02 | L-fuculose phosphate aldolase |
| TON_1528 | 1402483 | 1402734 | - | 489.54 | 659.97 | 420.14 | 1.35 | 0.86 | hypothetical protein |
| TON_1529 | 1402842 | 1403369 | - | 1573.12 | 1357.20 | 1555.92 | 0.86 | 0.99 | cob(I)yrinic acid a,c-diamide adenosyltransferase |
| TON_1530 | 1403465 | 1404352 | + | 199.90 | 64.91 | 89.45 | 0.32 | 0.45 | hypothetical protein |
| TON_1531 | 1405237 | 1405884 | + | 456.60 | 224.80 | 461.20 | 0.49 | 1.01 | hypothetical protein |
| TON_1532 | 1405939 | 1406688 | - | 902.98 | 425.55 | 1039.72 | 0.47 | 1.15 | lipoate-protein ligase A, N-terminal section |
| TON_1533 | 1407539 | 1408015 | - | 2850.92 | 1899.36 | 1493.59 | 0.67 | 0.52 | signal peptidase I |
| TON_1534 | 1407988 | 1408539 | - | 2492.00 | 1566.37 | 1795.68 | 0.63 | 0.72 | hypothetical protein |
| TON_1535 | 1408604 | 1409683 | + | 1985.42 | 2137.38 | 5475.77 | 1.08 | 2.76 | hypothetical protein |
| TON_1536 | 1409693 | 1410628 | + | 1142.64 | 1432.94 | 3017.25 | 1.25 | 2.64 | microsomal dipeptidase |
| TON_1537 | 1410670 | 1410870 | + | 8155.21 | 5484.11 | 4414.05 | 0.67 | 0.54 | hypothetical protein |
| TON_1538 | 1410861 | 1411337 | + | 1709.41 | 1709.43 | 2760.61 | 1.00 | 1.61 | nucleic acid-binding protein |

| | | | | | | | | | |
|---|---|---|---|---|---|---|---|---|---|
| TON_1539 | 1411343 | 1412191 | + | 4128.72 | 2541.30 | 5961.17 | 0.62 | 1.44 | SAM-dependent methyltransferase |
| TON_1540 | 1412197 | 1413594 | - | 1845.71 | 1208.14 | 4155.95 | 0.65 | 2.25 | membrane protein |
| TON_1541 | 1413601 | 1414923 | - | 614.48 | 477.25 | 1897.60 | 0.78 | 3.09 | membrane protein |
| TON_1542 | 1414934 | 1417909 | - | 3293.89 | 1853.68 | 6489.83 | 0.56 | 1.97 | hypothetical protein |
| TON_1543 | 1418111 | 1419982 | - | 590.63 | 1585.61 | 643.78 | 2.68 | 1.09 | acylamino acid-releasing protein |
| TON_1544 | 1420051 | 1420377 | + | 366.87 | 240.43 | 841.75 | 0.66 | 2.29 | membrane protein |
| TON_1545 | 1420345 | 1420992 | - | 1050.64 | 565.00 | 1610.18 | 0.54 | 1.53 | hypothetical protein |
| TON_1546 | 1421045 | 1421272 | + | 5503.07 | 15753.90 | 6580.75 | 2.86 | 1.20 | regulatory protein AsnC |
| TON_1547 | 1421316 | 1422170 | - | 17.04 | 42.07 | 28.60 | 2.47 | 1.68 | hypothetical protein |
| TON_1548 | 1422170 | 1422832 | - | 20.44 | 12.02 | 7.33 | 0.59 | 0.36 | hypothetical protein |
| TON_1549 | 1422874 | 1423023 | + | 96.55 | 10.82 | 16.13 | 0.11 | 0.17 | hypothetical protein |
| TON_1550 | 1423097 | 1423444 | - | 1620.82 | 270.48 | 139.31 | 0.17 | 0.09 | hypothetical protein |
| TON_1551 | 1423567 | 1424241 | - | 91262.29 | 3125.53 | 1919.60 | 0.03 | 0.02 | phosphate transport system regulator PhoU |
| TON_1552 | 1424246 | 1425466 | - | 32303.96 | 1166.06 | 838.82 | 0.04 | 0.03 | sodium/phosphate symporter |
| TON_1553 | 1425563 | 1427200 | - | 28493.27 | 20017.85 | 15382.45 | 0.70 | 0.54 | metallophosphoesterase |
| TON_1554 | 1427309 | 1427665 | + | 2180.78 | 1630.09 | 4315.06 | 0.75 | 1.98 | hypothetical protein |
| TON_1555 | 1427662 | 1428228 | - | 93.14 | 50.49 | 116.58 | 0.54 | 1.25 | signal sequence peptidase |
| TON_1556 | 1428248 | 1428649 | - | 1048.37 | 776.58 | 1830.14 | 0.74 | 1.75 | hypothetical protein |
| TON_1557 | 1428823 | 1430100 | - | 10399.60 | 6456.63 | 16202.94 | 0.62 | 1.56 | 2-methylthioadenine synthetase |
| TON_1558 | 1430214 | 1430426 | - | 8427.81 | 18397.38 | 33056.98 | 2.18 | 3.92 | RNA-binding protein |
| TON_1559 | 1430702 | 1431880 | + | 236952.10 | 53840.94 | 70785.38 | 0.23 | 0.30 | Coenzyme F420 hydrogenase subunit alpha |
| TON_1560 | 1431883 | 1432560 | + | 60792.68 | 22274.24 | 17063.02 | 0.37 | 0.28 | Coenzyme F420 hydrogenase subunit gamma |
| TON_1561 | 1432557 | 1433387 | + | 70409.70 | 39566.86 | 18512.61 | 0.56 | 0.26 | Coenzyme F420 hydrogenase/dehydrogenase beta subunit |
| TON_1562 | 1433434 | 1434192 | + | 12145.36 | 11296.40 | 3808.40 | 0.93 | 0.31 | TonB-dependent receptor protein:Formate dehydrogenase subunit FdhD |
| TON_1563 | 1434543 | 1436678 | + | 17227.04 | 273948.28 | 93744.30 | 15.90 | 5.44 | formate dehydrogenase subunit alpha |
| TON_1564 | 1436684 | 1437184 | + | 4849.97 | 119564.91 | 26014.30 | 24.65 | 5.36 | 4Fe-4S binding protein |
| TON_1565 | 1437189 | 1438631 | + | 4461.51 | 55593.64 | 13598.50 | 12.46 | 3.05 | hydrogenase 4 subunit D |
| TON_1566 | 1438635 | 1439849 | + | 474.77 | 16049.62 | 3401.46 | 33.80 | 7.16 | NADH dehydrogenase (quinone) |
| TON_1567 | 1439852 | 1441924 | + | 1988.83 | 69397.68 | 19387.36 | 34.89 | 9.75 | Hydrogenase 4, component B or formate hydrogen lyase subunit 3 |
| TON_1568 | 1441935 | 1442858 | + | 2041.07 | 61219.60 | 17851.97 | 29.99 | 8.75 | Hydrogenase 4, component C or formate hydrogen lyase subunit 4 |

| | | | | | | | | | |
|---|---|---|---|---|---|---|---|---|---|
| TON_1569 | 1442869 | 1444623 | + | 3414.29 | 141446.06 | 40054.94 | 41.43 | 11.73 | Hydrogenase 4, component G or formate hydrogen lyase subunit 5 |
| TON_1570 | 1444625 | 1445122 | + | 616.75 | 37018.35 | 11769.82 | 60.02 | 19.08 | Formate hydrogen lyase subunit 6 (hydrogenase 3 component F) |
| TON_1571 | 1445119 | 1445940 | + | 984.76 | 63079.29 | 15411.05 | 64.06 | 15.65 | Hydrogenase 4, component I or formate hydrogen lyase subunit 7 |
| TON_1572 | 1445900 | 1446181 | + | 461.14 | 47109.02 | 22054.85 | 102.16 | 47.83 | hypothetical protein |
| TON_1573 | 1446187 | 1447215 | + | 136.30 | 51505.20 | 10462.47 | 377.88 | 76.76 | formate transporter |
| TON_1574 | 1447258 | 1447539 | + | 74.96 | 12625.96 | 2710.75 | 168.43 | 36.16 | Na+/H+ antiporter subunit MnhF |
| TON_1575 | 1447536 | 1447895 | + | 64.74 | 10875.66 | 2908.73 | 167.98 | 44.93 | Na+/H+ antiporter subunit MnhG |
| TON_1576 | 1447888 | 1448142 | + | 6.81 | 2003.95 | 556.52 | 294.05 | 81.66 | hypothetical protein |
| TON_1577 | 1448143 | 1448898 | + | 185.14 | 36900.54 | 12571.24 | 199.31 | 67.90 | Multisubunit Na+/H+ antiporter MnhB subunit |
| TON_1578 | 1448895 | 1449299 | + | 170.37 | 26070.56 | 12952.52 | 153.02 | 76.02 | hypothetical protein |
| TON_1579 | 1449296 | 1449781 | + | 134.03 | 40966.14 | 8894.09 | 305.66 | 66.36 | hypothetical protein |
| TON_1580 | 1449793 | 1451361 | + | 173.78 | 50970.25 | 10375.95 | 293.30 | 59.71 | putative monovalent cation/H+ antiporter subunit D |
| TON_1581 | 1451413 | 1452000 | + | 113.58 | 18630.59 | 3484.31 | 164.03 | 30.68 | mobA molybdopterin-guanine dinucleotide biosynthesis protein MobA |
| TON_1582 | 1452138 | 1452644 | + | 36470.16 | 1862.10 | 526.46 | 0.05 | 0.01 | putative monovalent cation/H+ antiporter subunit E |
| TON_1583 | 1452641 | 1452904 | + | 25617.36 | 1603.64 | 302.82 | 0.06 | 0.01 | putative monovalent cation/H+ antiporter subunit F |
| TON_1584 | 1452901 | 1453272 | + | 13333.43 | 1446.16 | 238.30 | 0.11 | 0.02 | putative monovalent cation/H+ antiporter subunit G |
| TON_1585 | 1453269 | 1453535 | + | 6607.09 | 699.64 | 167.18 | 0.11 | 0.03 | hypothetical protein |
| TON_1586 | 1453532 | 1453828 | + | 25466.30 | 2221.53 | 677.51 | 0.09 | 0.03 | membrane bound hydrogenase subunit MbhE |
| TON_1587 | 1453825 | 1454271 | + | 13140.34 | 1544.74 | 417.21 | 0.12 | 0.03 | putative monovalent cation/H+ antiporter subunit B |
| TON_1588 | 1454268 | 1454627 | + | 976.81 | 455.61 | 66.72 | 0.47 | 0.07 | putative monovalent cation/H+ antiporter subunit C |
| TON_1589 | 1454624 | 1456222 | + | 27034.87 | 9053.23 | 1840.41 | 0.33 | 0.07 | putative monovalent cation/H+ antiporter subunit D |
| TON_1590 | 1456224 | 1456571 | + | 24218.03 | 7424.35 | 1759.02 | 0.31 | 0.07 | membrane bound hydrogenase subunit MbhI |
| TON_1591 | 1456576 | 1457103 | + | 7178.40 | 1875.32 | 493.46 | 0.26 | 0.07 | membrane bound hydrogenase, NiFe-hydrogenase small subunit |
| TON_1592 | 1457103 | 1457672 | + | 9221.75 | 2630.26 | 1066.85 | 0.29 | 0.12 | membrane bound hydrogenase, NiFe-hydrogenase large subunit 1 |
| TON_1593 | 1457669 | 1458949 | + | 25392.47 | 7717.67 | 3338.40 | 0.30 | 0.13 | membrane bound hydrogenase, NiFe-hydrogenase large subunit 2 |
| TON_1594 | 1458955 | 1459947 | + | 5153.23 | 2032.80 | 914.34 | 0.39 | 0.18 | membrane bound hydrogenase, MbhM subunit |
| TON_1595 | 1459944 | 1460402 | + | 4145.76 | 2882.70 | 664.31 | 0.70 | 0.16 | NADH-plastoquinone oxidoreductase subunit |
| TON_1596 | 1460676 | 1461164 | - | 1127.87 | 1490.64 | 1237.69 | 1.32 | 1.10 | rubrerythrin-like protein |

| | | | | | | | | | |
|---|---|---|---|---|---|---|---|---|---|
| TON_1597 | 1461211 | 1461384 | - | 1.14 | 12.02 | 2.20 | 10.58 | 1.94 | membrane protein |
| TON_1598 | 1461381 | 1462079 | - | 993.85 | 330.59 | 197.97 | 0.33 | 0.20 | NAD(P)H-flavin oxidoreductase |
| TON_1599 | 1462117 | 1462602 | - | 2355.70 | 720.08 | 544.79 | 0.31 | 0.23 | RNA-binding protein |
| TON_1600 | 1462664 | 1463869 | + | 2371.60 | 2977.67 | 1867.54 | 1.26 | 0.79 | ATPase |
| TON_1601 | 1463866 | 1464393 | + | 1436.82 | 1286.28 | 1118.18 | 0.90 | 0.78 | membrane-bound metal-dependent hydrolase |
| TON_1602 | 1464413 | 1464916 | + | 1928.63 | 821.05 | 898.21 | 0.43 | 0.47 | hypothetical protein |
| TON_1603 | 1465349 | 1466341 | + | 9501.16 | 3351.53 | 3479.18 | 0.35 | 0.37 | thioredoxin reductase |
| TON_1604 | 1466361 | 1466552 | - | 19.31 | 2.40 | 6.60 | 0.12 | 0.34 | hypothetical protein |
| TON_1605 | 1466833 | 1468170 | + | 1113.11 | 489.27 | 934.14 | 0.44 | 0.84 | 4-aminobutyrate aminotransferase |
| TON_1606 | 1468245 | 1468844 | + | 435.02 | 364.24 | 616.65 | 0.84 | 1.42 | membrane protein |
| TON_1607 | 1468841 | 1469125 | - | 6550.29 | 4613.77 | 3199.82 | 0.70 | 0.49 | hypothetical protein |
| TON_1608 | 1469112 | 1469795 | - | 22304.17 | 15043.44 | 11518.32 | 0.67 | 0.52 | deoxyribose-phosphate aldolase |
| TON_1609 | 1469962 | 1470267 | - | 3128.06 | 1687.79 | 1442.27 | 0.54 | 0.46 | hypothetical protein |
| TON_1610 | 1470299 | 1471516 | - | 1773.02 | 877.55 | 508.13 | 0.49 | 0.29 | hypothetical protein |
| TON_1611 | 1471678 | 1472895 | - | 77.24 | 48.09 | 50.59 | 0.62 | 0.66 | permease |
| TON_1612 | 1472958 | 1473863 | - | 139.71 | 233.21 | 132.71 | 1.67 | 0.95 | arylsulfatase regulator (Fe-S oxidoreductase) |
| TON_1613 | 1474039 | 1475331 | + | 9094.54 | 12611.53 | 11117.25 | 1.39 | 1.22 | eno phosphopyruvate hydratase |
| TON_1614 | 1475513 | 1476343 | + | 3773.21 | 1952.26 | 4491.77 | 0.52 | 1.19 | biotin synthase |
| TON_1615 | 1476423 | 1476875 | - | 18981.88 | 12404.76 | 13447.45 | 0.65 | 0.71 | transcriptional regulator |
| TON_1616 | 1476931 | 1477485 | - | 1225.55 | 1501.46 | 1050.72 | 1.23 | 0.86 | putative deoxyribonucleotide triphosphate pyrophosphatase |
| TON_1617 | 1477528 | 1478016 | - | 3150.77 | 4605.35 | 2806.81 | 1.46 | 0.89 | hypothetical protein |
| TON_1618 | 1478074 | 1478991 | + | 3830.00 | 1440.15 | 3413.19 | 0.38 | 0.89 | hypothetical protein |
| TON_1619 | 1478988 | 1479920 | - | 806.43 | 286.11 | 424.54 | 0.35 | 0.53 | hypothetical protein |
| TON_1620 | 1480028 | 1480456 | - | 6519.63 | 3359.95 | 2323.61 | 0.52 | 0.36 | molybdopterin converting factor subunit 2 |
| TON_1621 | 1480556 | 1481428 | + | 3559.67 | 2782.93 | 3939.65 | 0.78 | 1.11 | hypothetical protein |
| TON_1622 | 1481421 | 1482119 | - | 3201.89 | 1871.71 | 2433.59 | 0.58 | 0.76 | hypothetical protein |
| TON_1623 | 1482121 | 1482387 | - | 4982.86 | 3069.03 | 3173.42 | 0.62 | 0.64 | molybdopterin converting factor subunit 1 |
| TON_1624 | 1482459 | 1482971 | - | 8876.46 | 2126.57 | 3223.28 | 0.24 | 0.36 | hypothetical protein |
| TON_1625 | 1483012 | 1483533 | - | 5472.40 | 907.61 | 769.16 | 0.17 | 0.14 | putative transcriptional regulator |
| TON_1626 | 1483639 | 1485897 | + | 24355.46 | 1156.45 | 101.19 | 0.05 | 0.00 | membrane protein |
| TON_1627 | 1485894 | 1488266 | + | 37498.08 | 3629.23 | 445.80 | 0.10 | 0.01 | hypothetical protein |
| TON_1628 | 1488321 | 1489220 | + | 38992.82 | 7221.19 | 11659.10 | 0.19 | 0.30 | hypothetical protein |

| | | | | | | | | | |
|---|---|---|---|---|---|---|---|---|---|
| TON_1629 | 1489266 | 1489784 | + | 5319.06 | 1007.38 | 2817.81 | 0.19 | 0.53 | hypothetical protein |
| TON_1630 | 1489794 | 1490945 | + | 4748.88 | 2179.46 | 3504.11 | 0.46 | 0.74 | tRNA/rRNA cytosine-C5-methylase |
| TON_1631 | 1490948 | 1491994 | + | 1218.74 | 1622.87 | 1649.04 | 1.33 | 1.35 | membrane protein |
| TON_1632 | 1491997 | 1492833 | + | 3435.87 | 2978.87 | 3062.71 | 0.87 | 0.89 | ppnK inorganic polyphosphate/ATP-NAD kinase |
| TON_1633 | 1492817 | 1493608 | - | 2464.74 | 3195.26 | 5000.63 | 1.30 | 2.03 | membrane protein |
| TON_1634 | 1493599 | 1494576 | - | 3240.50 | 3646.06 | 6335.85 | 1.13 | 1.96 | putative DNA-binding/iron metalloprotein/AP endonuclease |
| TON_1635 | 1494608 | 1495141 | - | 1030.19 | 1491.84 | 1112.31 | 1.45 | 1.08 | hypothetical protein |
| TON_1636 | 1495190 | 1496506 | - | 3547.18 | 2899.53 | 2490.05 | 0.82 | 0.70 | acyl-CoA synthetase large subunit |
| TON_1637 | 1496507 | 1497712 | - | 4038.99 | 2302.08 | 2623.50 | 0.57 | 0.65 | hypothetical protein |
| TON_1638 | 1497785 | 1498267 | + | 935.92 | 247.64 | 255.16 | 0.26 | 0.27 | phosphopantetheine adenylyltransferase |
| TON_1639 | 1498245 | 1498472 | - | 22.72 | 6.01 | 18.33 | 0.26 | 0.81 | hypothetical protein |
| TON_1640 | 1498586 | 1499266 | + | 1013.15 | 979.73 | 860.08 | 0.97 | 0.85 | triosephosphate isomerase |
| TON_1641 | 1499968 | 1500654 | - | 156.74 | 197.15 | 309.42 | 1.26 | 1.97 | hypothetical protein |
| TON_1642 | 1500693 | 1506008 | - | 2979.27 | 19295.37 | 9732.91 | 6.48 | 3.27 | DNA polymerase II large subunit |
| TON_1643 | 1506011 | 1508116 | - | 927.97 | 5267.73 | 3512.18 | 5.68 | 3.78 | DNA polymerase II small subunit |
| TON_1644 | 1508116 | 1509363 | - | 667.86 | 996.56 | 815.35 | 1.49 | 1.22 | cdc6 cell division control protein 6 |
| TON_1645 | 1509623 | 1510249 | - | 2756.64 | 5806.28 | 2282.55 | 2.11 | 0.83 | hypothetical protein |
| TON_1646 | 1510927 | 1511943 | + | 113.58 | 167.10 | 294.76 | 1.47 | 2.60 | calcium-gated potassium channel protein |
| TON_1647 | 1512120 | 1513181 | + | 34749.39 | 18640.21 | 25850.79 | 0.54 | 0.74 | radA DNA repair and recombination protein RadA |
| TON_1648 | 1513217 | 1513969 | + | 1154.00 | 557.79 | 1100.58 | 0.48 | 0.95 | hypothetical protein |
| TON_1649 | 1513966 | 1514346 | - | 950.68 | 797.01 | 412.81 | 0.84 | 0.43 | hypothetical protein |
| TON_1650 | 1514356 | 1515084 | - | 1083.58 | 1412.50 | 555.79 | 1.30 | 0.51 | hypothetical protein |
| TON_1651 | 1515202 | 1515975 | + | 2916.79 | 1198.52 | 1716.49 | 0.41 | 0.59 | 5'-methylthioadenosine phosphorylase |
| TON_1652 | 1516122 | 1516580 | + | 205.58 | 281.30 | 145.18 | 1.37 | 0.71 | nuclease |
| TON_1653 | 1516653 | 1517927 | + | 2331.85 | 1829.64 | 4010.04 | 0.78 | 1.72 | N-ethylammeline chlorohydrolase |
| TON_1654 | 1517924 | 1518883 | + | 654.23 | 1632.49 | 1946.73 | 2.50 | 2.98 | hypothetical protein |
| TON_1655 | 1518894 | 1519451 | + | 389.59 | 848.70 | 435.54 | 2.18 | 1.12 | hypothetical protein |
| TON_1656 | 1519547 | 1520119 | + | 48.84 | 51.69 | 35.20 | 1.06 | 0.72 | daunorubicin resistance ATP-binding protein drrA |
| TON_1657 | 1520112 | 1520819 | + | 11.36 | 12.02 | 5.87 | 1.06 | 0.52 | hypothetical protein |
| TON_1658 | 1520990 | 1523023 | + | 218.08 | 372.66 | 258.10 | 1.71 | 1.18 | S-layer protein |
| TON_1659 | 1523066 | 1524508 | + | 1568.57 | 1622.87 | 1670.30 | 1.03 | 1.06 | putative YjeF-ralted carbohydrate kinase |
| TON_1660 | 1524547 | 1525923 | - | 2690.77 | 2308.09 | 3314.94 | 0.86 | 1.23 | alpha-amylase |

| | | | | | | | | | |
|---|---|---|---|---|---|---|---|---|---|
| TON_1661 | 1526073 | 1526630 | - | 2260.29 | 2512.45 | 2547.98 | 1.11 | 1.13 | transcriptional regulator |
| TON_1662 | 1526631 | 1526936 | - | 8259.71 | 5714.92 | 7341.11 | 0.69 | 0.89 | hypothetical protein |
| TON_1663 | 1526961 | 1527536 | - | 10554.07 | 5498.54 | 6744.99 | 0.52 | 0.64 | transcriptional regulator |
| TON_1664 | 1527681 | 1528997 | + | 1407.29 | 1557.96 | 1010.39 | 1.11 | 0.72 | cation transporter |
| TON_1665 | 1529056 | 1530465 | + | 7096.63 | 10796.32 | 5766.13 | 1.52 | 0.81 | succinyl-CoA synthetase large subunit |
| TON_1666 | 1530586 | 1531740 | + | 2729.38 | 2778.12 | 3957.98 | 1.02 | 1.45 | tryptophanyl-tRNA synthetase |
| TON_1667 | 1531753 | 1532430 | + | 362.33 | 632.32 | 646.71 | 1.75 | 1.78 | 2-hydroxyhepta-2,4-diene-1,7-dioate isomerase |
| TON_1668 | 1532405 | 1532791 | + | 21.58 | 111.80 | 42.53 | 5.18 | 1.97 | hypothetical protein |
| TON_1669 | 1533149 | 1534861 | + | 5814.28 | 17892.48 | 6746.46 | 3.08 | 1.16 | oligopeptide transporter |
| TON_1670 | 1534872 | 1535384 | + | 1399.33 | 6930.27 | 1657.10 | 4.95 | 1.18 | Cell division GTPase |
| TON_1671 | 1535478 | 1536575 | - | 1093.80 | 1808.00 | 3050.24 | 1.65 | 2.79 | N6-adenine-specific DNA methylase |
| TON_1672 | 1536582 | 1537655 | - | 2184.19 | 1340.37 | 3389.73 | 0.61 | 1.55 | GTPase |
| TON_1673 | 1538036 | 1539466 | + | 23992.00 | 22527.89 | 29626.19 | 0.94 | 1.23 | hypothetical protein |
| TON_1674 | 1539478 | 1540461 | + | 5172.54 | 5987.80 | 7426.16 | 1.16 | 1.44 | hypothetical protein |
| TON_1675 | 1540468 | 1542327 | + | 7469.18 | 12363.89 | 9231.38 | 1.66 | 1.24 | type II/IV secretion system ATPase |
| TON_1676 | 1542339 | 1543406 | + | 2280.73 | 6907.43 | 2556.04 | 3.03 | 1.12 | hypothetical protein |
| TON_1677 | 1543403 | 1544317 | + | 834.83 | 3133.95 | 1640.24 | 3.75 | 1.96 | hypothetical protein |
| TON_1678 | 1544291 | 1544782 | - | 7881.48 | 16574.95 | 14017.18 | 2.10 | 1.78 | FKBP-type peptidyl-prolyl cis-trans isomerase |
| TON_1679 | 1544905 | 1545198 | + | 90.87 | 117.81 | 102.65 | 1.30 | 1.13 | hypothetical protein |
| TON_1680 | 1545238 | 1545732 | - | 2611.26 | 2964.45 | 1928.40 | 1.14 | 0.74 | hypothetical protein |
| TON_1681 | 1545802 | 1546662 | + | 127.21 | 84.15 | 44.73 | 0.66 | 0.35 | hypothetical protein |
| TON_1682 | 1546649 | 1547101 | + | 74.96 | 82.95 | 91.65 | 1.11 | 1.22 | hypothetical protein |
| TON_1683 | 1547070 | 1548023 | + | 208.99 | 278.89 | 288.16 | 1.33 | 1.38 | hypothetical protein |
| TON_1684 | 1548033 | 1549340 | + | 155.61 | 147.86 | 181.84 | 0.95 | 1.17 | hypothetical protein |
| TON_1685 | 1549337 | 1549762 | + | 3.41 | 14.43 | 14.66 | 4.23 | 4.30 | hypothetical protein |
| TON_1686 | 1549759 | 1550592 | - | 1857.07 | 972.52 | 1766.35 | 0.52 | 0.95 | carbohydrate/pyrimidine kinase |
| TON_1687 | 1550749 | 1552248 | + | 2796.40 | 1850.08 | 783.82 | 0.66 | 0.28 | carboxypeptidase |
| TON_1688 | 1552317 | 1552667 | - | 4489.91 | 2618.24 | 3213.75 | 0.58 | 0.72 | hypothetical protein |
| TON_1689 | 1552677 | 1553789 | - | 3432.46 | 2440.32 | 2399.86 | 0.71 | 0.70 | geranylgeranyl hydrogenase |
| TON_1690 | 1553851 | 1554453 | + | 697.40 | 528.94 | 857.15 | 0.76 | 1.23 | pyrrolidone-carboxylate peptidase |
| TON_1691 | 1554564 | 1556498 | + | 7143.19 | 3618.41 | 3320.80 | 0.51 | 0.46 | hypothetical protein |
| TON_1692 | 1556637 | 1557608 | - | 2827.06 | 1465.39 | 2730.55 | 0.52 | 0.97 | ATPase |

| | | | | | | | | | |
|---|---|---|---|---|---|---|---|---|---|
| TON_1693 | 1557715 | 1558443 | + | 475.91 | 450.80 | 231.70 | 0.95 | 0.49 | hypothetical protein |
| TON_1694 | 1558440 | 1559579 | - | 5918.78 | 3680.92 | 5337.92 | 0.62 | 0.90 | membrane-associated metalloprotease |
| TON_1695 | 1559631 | 1560515 | - | 545.20 | 504.89 | 824.15 | 0.93 | 1.51 | glycosyltransferase |
| TON_1696 | 1560512 | 1561783 | - | 1198.29 | 762.15 | 1767.82 | 0.64 | 1.48 | hypothetical protein |
| TON_1697 | 1561937 | 1563028 | + | 2649.88 | 3226.51 | 2349.27 | 1.22 | 0.89 | glycosyltransferase |
| TON_1698 | 1563114 | 1564316 | + | 679.22 | 2026.79 | 1761.22 | 2.98 | 2.59 | glycosyltransferase protein |
| TON_1699 | 1564267 | 1564704 | + | 1301.65 | 1896.96 | 2743.02 | 1.46 | 2.11 | o-acetyltransferase |
| TON_1700 | 1564701 | 1565834 | + | 3055.37 | 5742.57 | 5774.19 | 1.88 | 1.89 | Pleiotropic regulatory protein degT |
| TON_1701 | 1565834 | 1566859 | + | 3174.63 | 9276.83 | 8091.94 | 2.92 | 2.55 | hypothetical protein |
| TON_1702 | 1567851 | 1569401 | - | 85.19 | 876.35 | 265.43 | 10.29 | 3.12 | polysaccharide biosynthesis-like protein |
| TON_1703 | 1569422 | 1570165 | - | 208.99 | 635.93 | 377.61 | 3.04 | 1.81 | permease |
| TON_1704 | 1570184 | 1570729 | - | 544.06 | 3044.99 | 874.01 | 5.60 | 1.61 | cysC adenylylsulfate kinase |
| TON_1705 | 1570769 | 1572199 | - | 563.37 | 2386.23 | 1201.77 | 4.24 | 2.13 | mitomycin biosynthesis protein |
| TON_1706 | 1572205 | 1573140 | - | 1005.20 | 3792.72 | 2033.98 | 3.77 | 2.02 | hypothetical protein |
| TON_1707 | 1573170 | 1574309 | - | 436.16 | 3102.69 | 1552.98 | 7.11 | 3.56 | sat sulfate adenylyltransferase |
| TON_1708 | 1574335 | 1574562 | - | 300.99 | 1489.44 | 1374.81 | 4.95 | 4.57 | hypothetical protein |
| TON_1709 | 1574986 | 1575849 | + | 504.31 | 560.19 | 197.24 | 1.11 | 0.39 | cobalt/zinc/cadmium cation efflux pump protein |
| TON_1710 | 1575839 | 1576990 | - | 5634.82 | 2840.63 | 4398.65 | 0.50 | 0.78 | L-tyrosine decarboxylase |
| TON_1711 | 1577028 | 1578203 | - | 968.86 | 1503.86 | 494.93 | 1.55 | 0.51 | NapA-type sodium/hydrogen antiporter |
| TON_1712 | 1578205 | 1578453 | - | 332.80 | 247.64 | 172.31 | 0.74 | 0.52 | hypothetical protein |
| TON_1713 | 1578527 | 1579225 | + | 750.78 | 659.97 | 751.56 | 0.88 | 1.00 | hydrolase |
| TON_1714 | 1579197 | 1579826 | + | 381.64 | 506.10 | 462.67 | 1.33 | 1.21 | hypothetical protein |
| TON_1715 | 1579867 | 1583280 | - | 4188.92 | 7823.45 | 9895.68 | 1.87 | 2.36 | hypothetical protein |
| TON_1716 | 1583391 | 1585349 | + | 315.76 | 542.16 | 2322.14 | 1.72 | 7.35 | 4-alpha-Glucanotransferase |
| TON_1717 | 1585419 | 1586336 | - | 20191.54 | 2963.25 | 2329.47 | 0.15 | 0.12 | ATPase |
| TON_1718 | 1586378 | 1587832 | - | 54462.74 | 9013.56 | 3240.88 | 0.17 | 0.06 | dehydrogenase |
| TON_1719 | 1587989 | 1588534 | + | 1991.10 | 3433.28 | 2357.34 | 1.72 | 1.18 | hypothetical protein |
| TON_1720 | 1588554 | 1589696 | + | 654.23 | 1455.78 | 1077.12 | 2.23 | 1.65 | NapA-type sodium/hydrogen antiporter |
| TON_1721 | 1589836 | 1590453 | + | 461.14 | 435.17 | 995.73 | 0.94 | 2.16 | hypothetical protein |
| TON_1722 | 1590516 | 1591829 | + | 7426.01 | 4138.93 | 7264.85 | 0.56 | 0.98 | hisS histidyl-tRNA synthetase |
| TON_1723 | 1591834 | 1591953 | - | 35.21 | 68.52 | 33.00 | 1.95 | 0.94 | hypothetical protein |
| TON_1724 | 1591993 | 1592757 | - | 450.92 | 694.83 | 354.88 | 1.54 | 0.79 | phospholipase D-related protein |

| | | | | | | | | | |
|---|---|---|---|---|---|---|---|---|---|
| TON_1725 | 1593026 | 1593280 | + | 19.31 | 18.03 | 8.07 | 0.93 | 0.42 | hypothetical protein |
| TON_1726 | 1593266 | 1594894 | - | 2445.43 | 1996.74 | 1266.29 | 0.82 | 0.52 | hypothetical protein |
| TON_1727 | 1595089 | 1595256 | + | 775.77 | 751.33 | 624.71 | 0.97 | 0.81 | hypothetical protein |
| TON_1728 | 1595281 | 1595577 | + | 1202.84 | 556.59 | 809.49 | 0.46 | 0.67 | hypothetical protein |
| TON_1729 | 1595636 | 1598365 | + | 8725.40 | 8908.97 | 14623.56 | 1.02 | 1.68 | alaS alanyl-tRNA synthetase |
| TON_1730 | 1598383 | 1598772 | + | 193.09 | 378.67 | 641.58 | 1.96 | 3.32 | hypothetical protein |
| TON_1731 | 1598736 | 1599203 | - | 1618.55 | 812.64 | 1212.03 | 0.50 | 0.75 | hypothetical protein |
| TON_1732 | 1599285 | 1600040 | - | 960.91 | 760.95 | 1050.72 | 0.79 | 1.09 | NGG1p interacting factor 3 |
| TON_1733 | 1600173 | 1601951 | + | 2899.76 | 882.36 | 2649.16 | 0.30 | 0.91 | histone acetyltransferase Elp3 |
| TON_1734 | 1602228 | 1602803 | + | 219.21 | 283.70 | 167.18 | 1.29 | 0.76 | hypothetical protein |
| TON_1735 | 1602825 | 1603352 | + | 1294.84 | 346.21 | 857.15 | 0.27 | 0.66 | hypothetical protein |
| TON_1736 | 1603384 | 1603905 | + | 3954.94 | 2564.14 | 4854.72 | 0.65 | 1.23 | hypothetical protein |
| TON_1737 | 1603938 | 1604447 | + | 3320.01 | 1108.36 | 2514.25 | 0.33 | 0.76 | hypothetical protein |
| TON_1738 | 1604546 | 1605454 | - | 241.93 | 343.81 | 310.16 | 1.42 | 1.28 | protein DppA |
| TON_1739 | 1605464 | 1606114 | - | 908.66 | 935.26 | 1067.58 | 1.03 | 1.17 | hypothetical protein |
| TON_1740 | 1606317 | 1607063 | + | 114.72 | 100.98 | 63.06 | 0.88 | 0.55 | hypothetical protein |
| TON_1741 | 1607084 | 1607692 | + | 204.45 | 181.52 | 107.78 | 0.89 | 0.53 | putative transcriptional regulator |
| TON_1742 | 1607696 | 1608121 | - | 2145.57 | 1078.31 | 1753.89 | 0.50 | 0.82 | S-adenosylmethionine decarboxylase |
| TON_1743 | 1608429 | 1609346 | + | 1804.82 | 2722.82 | 1605.04 | 1.51 | 0.89 | preprotein translocase subunit SecF |
| TON_1744 | 1609343 | 1610866 | + | 2053.57 | 5107.84 | 4118.56 | 2.49 | 2.01 | secD preprotein translocase subunit SecD |
| TON_1745 | 1610866 | 1611558 | + | 1573.12 | 5647.60 | 3067.10 | 3.59 | 1.95 | Trk-type pottasium transport system, NAD-binding component |
| TON_1746 | 1611688 | 1611999 | + | 3682.34 | 3825.17 | 3878.06 | 1.04 | 1.05 | V-type ATP synthase subunit H |
| TON_1747 | 1612001 | 1613980 | + | 5453.09 | 17263.77 | 9641.99 | 3.17 | 1.77 | V-type ATP synthase subunit I |
| TON_1748 | 1613996 | 1614484 | + | 1017.70 | 8245.40 | 2691.69 | 8.10 | 2.64 | V-type ATP synthase subunit K |
| TON_1749 | 1614522 | 1615133 | + | 2420.44 | 9962.04 | 5859.98 | 4.12 | 2.42 | V-type ATP synthase subunit E |
| TON_1750 | 1615139 | 1616239 | + | 4302.50 | 22722.63 | 10788.03 | 5.28 | 2.51 | V-type ATP synthase subunit C |
| TON_1751 | 1616236 | 1616544 | + | 1092.66 | 7551.77 | 2457.79 | 6.91 | 2.25 | V-type ATP synthase subunit F |
| TON_1752 | 1616550 | 1618307 | + | 4369.51 | 32239.89 | 12217.83 | 7.38 | 2.80 | V-type ATP synthase subunit A |
| TON_1753 | 1618307 | 1619704 | + | 3093.98 | 21709.24 | 7767.11 | 7.02 | 2.51 | V-type ATP synthase subunit B |
| TON_1754 | 1619732 | 1620370 | + | 1585.61 | 11378.15 | 5347.45 | 7.18 | 3.37 | V-type ATP synthase subunit D |
| TON_1755 | 1620478 | 1621245 | + | 849.60 | 2246.78 | 875.48 | 2.64 | 1.03 | flavoprotein |
| TON_1756 | 1621256 | 1622029 | + | 739.42 | 1346.38 | 863.01 | 1.82 | 1.17 | Methyl-accepting chemotaxis protein |

| | | | | | | | | | |
|---|---|---|---|---|---|---|---|---|---|
| TON_1757 | 1622035 | 1623249 | + | 499.76 | 2126.57 | 743.50 | 4.26 | 1.49 | hypothetical protein |
| TON_1758 | 1623395 | 1624225 | - | 793.94 | 699.64 | 2114.64 | 0.88 | 2.66 | ATPase |
| TON_1759 | 1624248 | 1624490 | - | 68.15 | 18.03 | 11.73 | 0.26 | 0.17 | hypothetical protein |
| TON_1760 | 1624670 | 1624885 | - | 21.58 | 7.21 | 16.13 | 0.33 | 0.75 | hypothetical protein |
| TON_1761 | 1625000 | 1625902 | - | 1733.27 | 888.37 | 1814.75 | 0.51 | 1.05 | hypothetical protein |
| TON_1762 | 1625951 | 1626652 | - | 435.02 | 282.50 | 688.50 | 0.65 | 1.58 | dihydroorotate dehydrogenase electron transfer subunit |
| TON_1763 | 1626636 | 1627862 | - | 1246.00 | 573.42 | 1767.09 | 0.46 | 1.42 | dihydroorotase |
| TON_1764 | 1628221 | 1630653 | + | 24270.28 | 46990.00 | 24437.85 | 1.94 | 1.01 | dipeptide/oligopeptide ABC transporter |
| TON_1765 | 1630854 | 1631897 | + | 3355.22 | 6442.21 | 1280.95 | 1.92 | 0.38 | oligopeptide transport system permease protein appB |
| TON_1766 | 1631909 | 1633369 | + | 1679.88 | 5173.96 | 2087.51 | 3.08 | 1.24 | dipeptide/oligopeptide ABC transporter permease |
| TON_1767 | 1633380 | 1634339 | + | 2070.61 | 4275.97 | 2144.70 | 2.07 | 1.04 | dipeptide/oligopeptide ABC transporter ATPase |
| TON_1768 | 1634350 | 1635357 | + | 1548.13 | 3653.27 | 1440.80 | 2.36 | 0.93 | dipeptide/oligopeptide ABC transporter ATPase |
| TON_1769 | 1635391 | 1635867 | + | 1381.16 | 2988.49 | 1215.70 | 2.16 | 0.88 | membrane protein |
| TON_1770 | 1635864 | 1636625 | + | 2334.12 | 4434.65 | 2536.98 | 1.90 | 1.09 | NAD synthetase |
| TON_1771 | 1636635 | 1637504 | + | 149.93 | 473.64 | 140.05 | 3.16 | 0.93 | permease |
| TON_1772 | 1637501 | 1638832 | - | 17052.12 | 4972.00 | 18446.62 | 0.29 | 1.08 | glnA glutamine synthetase |
| TON_1773 | 1639052 | 1639459 | + | 1339.14 | 1816.42 | 1104.25 | 1.36 | 0.82 | hypothetical protein |
| TON_1774 | 1639465 | 1639740 | + | 110.17 | 455.61 | 181.11 | 4.14 | 1.64 | membrane protein |
| TON_1775 | 1639782 | 1641389 | + | 2732.79 | 3126.74 | 2102.17 | 1.14 | 0.77 | membrane protein |
| TON_1776 | 1641743 | 1642594 | - | 138.57 | 449.60 | 450.20 | 3.24 | 3.25 | permease |
| TON_1777 | 1642591 | 1643631 | - | 2184.19 | 2817.79 | 4292.33 | 1.29 | 1.97 | DNA primase small subunit |
| TON_1778 | 1643624 | 1644829 | - | 3907.23 | 3647.26 | 5670.07 | 0.93 | 1.45 | DNA primase large subunit |
| TON_1779 | 1645143 | 1645892 | + | 11117.44 | 18421.42 | 22418.53 | 1.66 | 2.02 | ATPase |
| TON_1780 | 1646085 | 1647140 | + | 344.15 | 489.27 | 701.70 | 1.42 | 2.04 | hypothetical protein |
| TON_1781 | 1647122 | 1648249 | + | 17.04 | 57.70 | 22.73 | 3.39 | 1.33 | membrane protein |
| TON_1782 | 1648261 | 1648569 | - | 3145.10 | 3944.18 | 2283.28 | 1.25 | 0.73 | hypothetical protein |
| TON_1783 | 1648641 | 1649831 | + | 6399.23 | 6673.02 | 5943.57 | 1.04 | 0.93 | putative tRNA/rRNA methyltransferase |
| TON_1784 | 1649925 | 1650668 | - | 830.29 | 506.10 | 1398.27 | 0.61 | 1.68 | hypothetical protein |
| TON_1785 | 1650705 | 1651973 | - | 1579.93 | 1370.43 | 2100.71 | 0.87 | 1.33 | diaminopimelate aminotransferase |
| TON_1786 | 1651987 | 1652322 | - | 204.45 | 189.94 | 247.10 | 0.93 | 1.21 | hypothetical protein |
| TON_1787 | 1652362 | 1654239 | - | 354.38 | 360.64 | 221.44 | 1.02 | 0.62 | oligopeptide transporter |
| TON_1788 | 1654575 | 1655420 | + | 4057.16 | 1988.32 | 2917.53 | 0.49 | 0.72 | hydrolase |

| | | | | | | | | | |
|---|---|---|---|---|---|---|---|---|---|
| TON_1789 | 1655423 | 1656790 | - | 6260.66 | 7143.05 | 6541.15 | 1.14 | 1.04 | phosphopentomutase |
| TON_1790 | 1656820 | 1657224 | - | 598.58 | 621.50 | 880.61 | 1.04 | 1.47 | hypothetical protein |
| TON_1791 | 1657257 | 1658384 | - | 415.71 | 2192.68 | 1607.24 | 5.27 | 3.87 | ABC-type maltdextrin transport system, ATPase component |
| TON_1792 | 1658415 | 1662449 | - | 1964.97 | 10666.49 | 7344.04 | 5.43 | 3.74 | pullulanase |
| TON_1793 | 1662459 | 1663706 | - | 395.27 | 2042.42 | 1447.40 | 5.17 | 3.66 | ABC-type maltodextrin transport system permease |
| TON_1794 | 1663706 | 1664605 | - | 187.41 | 543.36 | 536.73 | 2.90 | 2.86 | sugar transport inner membrane protein (malf-like) |
| TON_1795 | 1664644 | 1665963 | - | 945.01 | 2948.82 | 2954.92 | 3.12 | 3.13 | ABC-type maltodextrin transport system |
| TON_1796 | 1666089 | 1668047 | - | 2101.27 | 2540.10 | 3087.64 | 1.21 | 1.47 | cyclomaltodextrinase |
| TON_1797 | 1668118 | 1669140 | + | 2265.97 | 5748.58 | 5071.76 | 2.54 | 2.24 | putative transcriptional regulator |
| TON_1798 | 1669180 | 1670532 | + | 947.28 | 3153.18 | 3965.31 | 3.33 | 4.19 | glycogen synthase |
| TON_1799 | 1670535 | 1670897 | - | 178.32 | 113.00 | 390.81 | 0.63 | 2.19 | ribonuclease P protein component 2 |
| TON_1800 | 1670980 | 1672062 | + | 1926.36 | 772.97 | 1820.61 | 0.40 | 0.95 | hypothetical protein |
| TON_1801 | 1672182 | 1672958 | + | 369.14 | 284.90 | 130.52 | 0.77 | 0.35 | membrane protein |
| TON_1802 | 1672996 | 1673178 | + | 460.01 | 241.63 | 317.49 | 0.53 | 0.69 | hypothetical protein |
| TON_1803 | 1673201 | 1676398 | - | 46053.10 | 20449.41 | 16624.54 | 0.44 | 0.36 | ileS isoleucyl-tRNA synthetase |
| TON_1804 | 1676828 | 1677202 | - | 638.33 | 512.11 | 333.62 | 0.80 | 0.52 | hypothetical protein |
| TON_1805 | 1677265 | 1678095 | - | 320.30 | 266.87 | 347.55 | 0.83 | 1.09 | permease |
| TON_1806 | 1678209 | 1678736 | + | 210.13 | 515.71 | 305.76 | 2.45 | 1.46 | oxidoreductase, Fe-S subunit |
| TON_1807 | 1678741 | 1680612 | + | 1606.05 | 2411.47 | 2090.44 | 1.50 | 1.30 | formaldehyde:ferredoxin oxidoreductase wor5 |
| TON_1808 | 1680637 | 1680858 | + | 46.57 | 179.12 | 91.65 | 3.85 | 1.97 | hypothetical protein |
| TON_1809 | 1680932 | 1681363 | + | 26830.42 | 12770.21 | 5241.13 | 0.48 | 0.20 | hypothetical protein |
| TON_1810 | 1681364 | 1681672 | - | 2234.16 | 1157.65 | 1051.45 | 0.52 | 0.47 | hypothetical protein |
| TON_1811 | 1681789 | 1683525 | + | 6995.54 | 1840.46 | 4524.03 | 0.26 | 0.65 | hypothetical protein |
| TON_1812 | 1683769 | 1685133 | - | 4757.96 | 2852.65 | 5498.50 | 0.60 | 1.16 | tRNA CCA-pyrophosphorylase |
| TON_1813 | 1685139 | 1685693 | - | 3338.19 | 2666.32 | 3042.91 | 0.80 | 0.91 | 2'-5' RNA ligase |
| TON_1814 | 1685834 | 1688800 | - | 2570.37 | 3547.48 | 2574.37 | 1.38 | 1.00 | thiol protease |
| TON_1815 | 1688892 | 1689356 | - | 4656.88 | 2328.52 | 3164.62 | 0.50 | 0.68 | xanthine/guanine phosphoribosyltransferase |
| TON_1816 | 1689494 | 1692226 | - | 6093.69 | 19018.88 | 21267.36 | 3.12 | 3.49 | ribonucleotide reductase subunit alpha |
| TON_1817 | 1692416 | 1692850 | - | 392.99 | 329.38 | 484.67 | 0.84 | 1.23 | hypothetical protein |
| TON_1818 | 1692847 | 1693653 | - | 3161.00 | 2095.31 | 3478.45 | 0.66 | 1.10 | sugar phosphatase |
| TON_1819 | 1693755 | 1696085 | + | 987.03 | 2523.27 | 1244.29 | 2.56 | 1.26 | dolichyl-phosphate-mannose-protein mannosyltransferase |
| TON_1820 | 1696082 | 1698436 | - | 4581.91 | 8651.72 | 6255.19 | 1.89 | 1.37 | oligosaccharyl transferase |

| | | | | | | | | | |
|---|---|---|---|---|---|---|---|---|---|
| TON_1821 | 1698600 | 1699724 | + | 1130.14 | 954.49 | 1462.80 | 0.84 | 1.29 | lps biosynthesis rfbU related protein |
| TON_1822 | 1699714 | 1700832 | + | 3564.21 | 3447.70 | 4259.34 | 0.97 | 1.20 | dolichol-phosphate mannosyltransferase |
| TON_1823 | 1700832 | 1701923 | + | 621.30 | 3733.81 | 2009.05 | 6.01 | 3.23 | glycosyltransferase |
| TON_1824 | 1703315 | 1709128 | + | 408.90 | 471.23 | 349.02 | 1.15 | 0.85 | hypothetical protein |
| TON_1825 | 1709207 | 1709929 | + | 1484.52 | 658.77 | 901.87 | 0.44 | 0.61 | hypothetical protein |
| TON_1826 | 1709932 | 1712184 | + | 1897.96 | 5004.46 | 1444.46 | 2.64 | 0.76 | hypothetical protein |
| TON_1827 | 1712287 | 1712883 | - | 135.16 | 234.42 | 170.11 | 1.73 | 1.26 | hypothetical protein |
| TON_1828 | 1712968 | 1713957 | - | 574.73 | 923.23 | 1051.45 | 1.61 | 1.83 | hypothetical protein |
| TON_1829 | 1714069 | 1714623 | - | 328.25 | 815.04 | 607.85 | 2.48 | 1.85 | hypothetical protein |
| TON_1830 | 1714620 | 1715903 | - | 1576.52 | 3963.42 | 2665.29 | 2.51 | 1.69 | hypothetical protein |
| TON_1831 | 1715900 | 1716820 | - | 648.56 | 2321.31 | 1412.94 | 3.58 | 2.18 | hypothetical protein |
| TON_1832 | 1716822 | 1717820 | - | 2531.75 | 2725.23 | 3177.82 | 1.08 | 1.26 | hypothetical protein |
| TON_1833 | 1717813 | 1719555 | - | 1008.61 | 1059.08 | 1322.75 | 1.05 | 1.31 | hypothetical protein |
| TON_1834 | 1719614 | 1720333 | + | 598.58 | 403.92 | 489.07 | 0.67 | 0.82 | hypothetical protein |
| TON_1835 | 1720324 | 1720563 | + | 104.50 | 44.48 | 105.59 | 0.43 | 1.01 | hypothetical protein |
| TON_1836 | 1720565 | 1721518 | - | 2103.55 | 2112.14 | 2208.49 | 1.00 | 1.05 | galE-2 UDP-glucose 4-epimerase |
| TON_1837 | 1721579 | 1722601 | + | 1298.25 | 1288.68 | 1286.82 | 0.99 | 0.99 | sugar-phosphate nucleotydyltransferase |
| TON_1838 | 1722829 | 1723050 | + | 35.21 | 21.64 | 19.80 | 0.61 | 0.56 | hypothetical protein |
| TON_1839 | 1723160 | 1723423 | + | 15.90 | 12.02 | 11.00 | 0.76 | 0.69 | hypothetical protein |
| TON_1840 | 1723469 | 1723873 | + | 198.77 | 96.17 | 45.46 | 0.48 | 0.23 | hypothetical protein |
| TON_1841 | 1724638 | 1725282 | + | 2220.54 | 1976.30 | 1519.25 | 0.89 | 0.68 | hypothetical protein |
| TON_1842 | 1725405 | 1726463 | + | 2556.74 | 1924.61 | 1684.23 | 0.75 | 0.66 | nucleotidyltransferase |
| TON_1843 | 1726473 | 1727474 | + | 2146.71 | 2116.95 | 1753.16 | 0.99 | 0.82 | rfbB dTDP-glucose 4,6-dehydratase |
| TON_1844 | 1727520 | 1727807 | + | 922.29 | 1452.17 | 666.51 | 1.57 | 0.72 | hypothetical protein |
| TON_1845 | 1727765 | 1728106 | + | 406.62 | 833.08 | 398.14 | 2.05 | 0.98 | hypothetical protein |
| TON_1846 | 1728181 | 1728384 | + | 646.28 | 561.39 | 346.82 | 0.87 | 0.54 | hypothetical protein |
| TON_1847 | 1728384 | 1728782 | + | 498.63 | 750.13 | 415.74 | 1.50 | 0.83 | nucleic acid-binding protein |
| TON_1848 | 1728787 | 1729344 | + | 835.97 | 1628.88 | 1101.31 | 1.95 | 1.32 | dTDP-4-dehydrorhamnose 3,5-epimerase |
| TON_1849 | 1729408 | 1729596 | + | 842.78 | 1258.63 | 947.33 | 1.49 | 1.12 | hypothetical protein |
| TON_1850 | 1729584 | 1730054 | + | 388.45 | 782.59 | 618.11 | 2.01 | 1.59 | nucleic acid-binding protein |
| TON_1851 | 1730051 | 1730917 | + | 982.49 | 2544.91 | 2383.73 | 2.59 | 2.43 | dTDP-4-dehydrorhamnose reductase |
| TON_1852 | 1730948 | 1732372 | + | 445.24 | 3998.28 | 669.44 | 8.98 | 1.50 | lipopolysaccharide O-side chain biosynthesis protein |

| | | | | | | | | | |
|---|---|---|---|---|---|---|---|---|---|
| TON_1853 | 1732764 | 1733333 | + | 442.97 | 1695.00 | 508.13 | 3.83 | 1.15 | hypothetical protein |
| TON_1854 | 1734419 | 1735939 | - | 595.17 | 4755.62 | 1386.54 | 7.99 | 2.33 | hypothetical protein |
| TON_1855 | 1735946 | 1737121 | - | 875.72 | 3255.36 | 2159.37 | 3.72 | 2.47 | glycosyltransferase |
| TON_1856 | 1737726 | 1738394 | - | 249.88 | 1538.72 | 670.91 | 6.16 | 2.68 | hypothetical protein |
| TON_1857 | 1738401 | 1739576 | - | 524.75 | 3029.36 | 1630.71 | 5.77 | 3.11 | membrane-bound galactosyl-transferase |
| TON_1858 | 1739589 | 1740710 | - | 826.88 | 3594.36 | 2690.22 | 4.35 | 3.25 | phosphatidylinositol glycantransferase-class A |
| TON_1859 | 1740707 | 1741186 | - | 922.29 | 1161.26 | 1426.13 | 1.26 | 1.55 | beta-1,4-galactosyltransferase |
| TON_1860 | 1741183 | 1741626 | - | 570.18 | 1505.06 | 906.27 | 2.64 | 1.59 | capsular polysaccharide biosynthesis protein |
| TON_1861 | 1741623 | 1742618 | - | 1378.89 | 2406.66 | 1522.92 | 1.75 | 1.10 | glycosyltransferase |
| TON_1862 | 1742622 | 1743512 | - | 2031.99 | 1371.63 | 1099.11 | 0.68 | 0.54 | Glycosyl transferase |
| TON_1863 | 1744746 | 1745537 | + | 1774.16 | 955.69 | 1630.71 | 0.54 | 0.92 | hypothetical protein |
| TON_1864 | 1745530 | 1746321 | + | 1392.52 | 730.89 | 1077.12 | 0.52 | 0.77 | hypothetical protein |
| TON_1865 | 1746411 | 1746590 | + | 1659.44 | 1168.47 | 2644.03 | 0.70 | 1.59 | hypothetical protein |
| TON_1866 | 1746610 | 1746885 | + | 605.39 | 337.80 | 952.47 | 0.56 | 1.57 | ef1B elongation factor 1-beta |
| TON_1867 | 1746996 | 1748003 | + | 649.69 | 587.84 | 601.98 | 0.90 | 0.93 | hypothetical protein |
| TON_1868 | 1747978 | 1748721 | + | 240.79 | 268.07 | 306.49 | 1.11 | 1.27 | hypothetical protein |
| TON_1869 | 1748735 | 1749760 | - | 16480.80 | 6307.57 | 19977.61 | 0.38 | 1.21 | hypothetical protein |
| TON_1870 | 1750066 | 1751910 | + | 13318.67 | 2435.51 | 1365.28 | 0.18 | 0.10 | anaerobic ribonucleoside triphosphate reductase |
| TON_1871 | 1751939 | 1752652 | + | 1059.72 | 450.80 | 126.12 | 0.43 | 0.12 | anaerobic ribonucleoside-triphosphate reductase activating enzyme |
| TON_1872 | 1752785 | 1753915 | + | 1633.31 | 3944.18 | 11276.36 | 2.41 | 6.90 | putative vitamin B12 transport protein |
| TON_1873 | 1753918 | 1754964 | + | 93.14 | 194.74 | 378.35 | 2.09 | 4.06 | iron(III) ABC transporter permease |
| TON_1874 | 1754957 | 1755730 | + | 82.92 | 234.42 | 314.56 | 2.83 | 3.79 | ABC transporter ATPase |
| TON_1875 | 1755727 | 1759485 | + | 1026.78 | 3117.12 | 5172.94 | 3.04 | 5.04 | cobalamin biosynthesis protein |
| TON_1876 | 1759461 | 1761383 | + | 785.99 | 1995.53 | 2777.48 | 2.54 | 3.53 | magnesium chelatase subunit ChlI |
| TON_1877 | 1761620 | 1763254 | + | 400.95 | 1174.48 | 789.69 | 2.93 | 1.97 | chaperonin beta subunit |
| TON_1878 | 1763378 | 1764445 | + | 1261.90 | 1494.25 | 1488.46 | 1.18 | 1.18 | hypothetical protein |
| TON_1879 | 1764762 | 1765724 | + | 105.63 | 76.94 | 59.39 | 0.73 | 0.56 | hypothetical protein |
| TON_1880 | 1765781 | 1766572 | + | 36.35 | 48.09 | 33.73 | 1.32 | 0.93 | hypothetical protein |
| TON_1881 | 1766586 | 1767173 | - | 4066.25 | 1179.29 | 1797.15 | 0.29 | 0.44 | hypothetical protein |
| TON_1882 | 1767180 | 1769162 | - | 27294.98 | 3091.87 | 5510.23 | 0.11 | 0.20 | DNA helicase |
| TON_1883 | 1769295 | 1769585 | - | 9898.70 | 3323.89 | 6901.17 | 0.34 | 0.70 | hypothetical protein |

| | | | | | | | | | |
|---|---|---|---|---|---|---|---|---|---|
| TON_1884 | 1770019 | 1771479 | + | 1784.38 | 923.23 | 1319.82 | 0.52 | 0.74 | inosine 5'-monophosphate dehydrogenase |
| TON_1885 | 1771558 | 1772958 | + | 3122.38 | 1265.84 | 1454.73 | 0.41 | 0.47 | L-aspartate oxidase |
| TON_1886 | 1773009 | 1774196 | + | 111.31 | 42.07 | 27.86 | 0.38 | 0.25 | hypothetical protein |
| TON_1887 | 1774242 | 1775150 | + | 779.17 | 694.83 | 621.05 | 0.89 | 0.80 | quinolinate synthetase |
| TON_1888 | 1775228 | 1776538 | + | 2990.62 | 1535.12 | 2704.16 | 0.51 | 0.90 | hypothetical protein |
| TON_1889 | 1776525 | 1777358 | + | 1827.54 | 1487.03 | 1867.54 | 0.81 | 1.02 | nicotinate-nucleotide pyrophosphorylase |
| TON_1890 | 1777491 | 1778498 | + | 294.18 | 170.70 | 211.90 | 0.58 | 0.72 | pyridoxal biosynthesis lyase PdxS |
| TON_1891 | 1778531 | 1779124 | + | 57.93 | 48.09 | 84.32 | 0.83 | 1.46 | glutamine amidotransferase subunit PdxT |
| TON_1892 | 1779399 | 1779569 | - | 49.98 | 52.89 | 67.46 | 1.06 | 1.35 | hypothetical protein |
| TON_1893 | 1779817 | 1780740 | + | 346.43 | 177.92 | 384.95 | 0.51 | 1.11 | GMP synthase |
| TON_1894 | 1780751 | 1781317 | + | 115.85 | 61.31 | 101.19 | 0.53 | 0.87 | GMP synthase |
| TON_1895 | 1781711 | 1782448 | - | 2540.84 | 7370.25 | 3288.54 | 2.90 | 1.29 | hypothetical protein |
| TON_1896 | 1782453 | 1785872 | - | 14234.14 | 38483.74 | 21448.47 | 2.70 | 1.51 | hypothetical protein |
| TON_1897 | 1785872 | 1788040 | - | 4561.47 | 13264.29 | 4910.45 | 2.91 | 1.08 | hypothetical protein |
| TON_1898 | 1788037 | 1789518 | - | 8279.02 | 11578.90 | 6418.70 | 1.40 | 0.78 | hypothetical protein |
| TON_1899 | 1789515 | 1789988 | - | 3003.12 | 2175.85 | 1734.82 | 0.72 | 0.58 | hypothetical protein |
| TON_1900 | 1789969 | 1790412 | - | 4246.84 | 1990.72 | 2028.85 | 0.47 | 0.48 | hypothetical protein |
| TON_1901 | 1790415 | 1791050 | - | 9001.40 | 6730.72 | 4083.36 | 0.75 | 0.45 | membrane protein |
| TON_1902 | 1791090 | 1791719 | - | 3480.16 | 4057.18 | 2656.50 | 1.17 | 0.76 | hypothetical protein |
| TON_1903 | 1791716 | 1792807 | - | 9283.09 | 9960.84 | 5661.28 | 1.07 | 0.61 | cell division GTPase |
| TON_1904 | 1792815 | 1795034 | - | 17132.76 | 13255.87 | 10372.28 | 0.77 | 0.61 | hypothetical protein |
| TON_1905 | 1795105 | 1796091 | - | 2572.64 | 1759.92 | 1440.80 | 0.68 | 0.56 | tRNA-modifying protein |
| TON_1906 | 1796355 | 1796939 | - | 832.56 | 312.55 | 431.87 | 0.38 | 0.52 | N5-glutamine methyltransferase |
| TON_1907 | 1797197 | 1798477 | + | 765.55 | 274.09 | 1539.05 | 0.36 | 2.01 | subtilisin-like serine protease |
| TON_1908 | 1798778 | 1800058 | + | 59.06 | 86.55 | 78.46 | 1.47 | 1.33 | subtilisin-like serine protease |
| TON_1909 | 1800169 | 1800324 | - | 2183.05 | 2859.86 | 2535.51 | 1.31 | 1.16 | 30S ribosomal protein S27ae |
| TON_1910 | 1800327 | 1800623 | - | 3843.63 | 4824.14 | 4887.72 | 1.26 | 1.27 | rps24e 30S ribosomal protein S24e |
| TON_1911 | 1800613 | 1801146 | - | 6185.70 | 6773.99 | 10683.91 | 1.10 | 1.73 | hypothetical protein |
| TON_1912 | 1801146 | 1801331 | - | 905.25 | 1348.79 | 1918.13 | 1.49 | 2.12 | DNA-directed RNA polymerase subunit E'' |
| TON_1913 | 1801328 | 1801894 | - | 2069.47 | 3428.47 | 4279.14 | 1.66 | 2.07 | DNA-directed RNA polymerase subunit E' |
| TON_1914 | 1801934 | 1802470 | - | 817.79 | 858.32 | 1067.58 | 1.05 | 1.31 | inorganic pyrophosphatase |
| TON_1915 | 1802582 | 1803034 | - | 3056.50 | 271.68 | 487.60 | 0.09 | 0.16 | hypothetical protein |

| | | | | | | | | | |
|---|---|---|---|---|---|---|---|---|---|
| TON_1916 | 1803162 | 1803278 | + | 23.85 | 16.83 | 22.73 | 0.71 | 0.95 | hypothetical protein |
| TON_1917 | 1803917 | 1804174 | + | 31.80 | 22.84 | 1.47 | 0.72 | 0.05 | membrane protein |
| TON_1918 | 1804922 | 1805980 | + | 567.91 | 429.16 | 294.03 | 0.76 | 0.52 | dolichol-phosphate mannosyltransferase |
| TON_1919 | 1806044 | 1807732 | + | 2087.64 | 2512.45 | 2475.39 | 1.20 | 1.19 | putative leucine aminopeptidase |
| TON_1920 | 1807729 | 1808070 | - | 5087.35 | 1880.13 | 570.45 | 0.37 | 0.11 | transcriptional regulator |
| TON_1921 | 1808140 | 1808790 | - | 2322.76 | 1293.49 | 2992.32 | 0.56 | 1.29 | exonuclease SbcD |
| TON_1922 | 1808834 | 1808947 | - | 1528.82 | 1637.30 | 2033.25 | 1.07 | 1.33 | 50S ribosomal protein L41 |
| TON_1923 | 1809305 | 1810099 | + | 3045.14 | 2799.76 | 1446.66 | 0.92 | 0.48 | carbon-nitrogen hydrolase |
| TON_1924 | 1810351 | 1810728 | + | 3542.63 | 556.59 | 657.71 | 0.16 | 0.19 | drug exporter |
| TON_1925 | 1810744 | 1811532 | - | 397.54 | 501.29 | 451.67 | 1.26 | 1.14 | SAM-dependent methyltransferase |
| TON_1926 | 1811628 | 1812332 | + | 13105.13 | 329.38 | 140.78 | 0.03 | 0.01 | hypothetical protein |
| TON_1927 | 1812316 | 1812705 | + | 3100.80 | 217.59 | 76.26 | 0.07 | 0.02 | hypothetical protein |
| TON_1928 | 1812728 | 1813219 | - | 15.90 | 10.82 | 15.40 | 0.68 | 0.97 | membrane protein |
| TON_1929 | 1813243 | 1813443 | - | 18.17 | 48.09 | 31.53 | 2.65 | 1.73 | membrane protein |
| TON_1930 | 1813448 | 1814224 | - | 78.37 | 185.13 | 109.25 | 2.36 | 1.39 | membrane protein |
| TON_1931 | 1814212 | 1814580 | - | 228.30 | 406.32 | 255.90 | 1.78 | 1.12 | transcriptional regulator |
| TON_1932 | 1814586 | 1815290 | - | 809.84 | 920.83 | 730.30 | 1.14 | 0.90 | membrane protein |
| TON_1933 | 1815296 | 1815625 | - | 1038.14 | 668.38 | 568.99 | 0.64 | 0.55 | hypothetical protein |
| TON_1934 | 1815744 | 1816778 | + | 2415.90 | 1418.51 | 2468.79 | 0.59 | 1.02 | diphthine synthase, DPH2 subunit |
| TON_1935 | 1816775 | 1817386 | + | 392.99 | 239.22 | 338.02 | 0.61 | 0.86 | DNA methylase |
| TON_1936 | 1817464 | 1818423 | + | 654.23 | 168.30 | 240.50 | 0.26 | 0.37 | hypothetical protein |
| TON_1937 | 1818543 | 1819340 | + | 736.01 | 2044.82 | 1247.23 | 2.78 | 1.69 | hypothetical protein |
| TON_1938 | 1819398 | 1820726 | + | 2354.56 | 1256.22 | 2741.55 | 0.53 | 1.16 | tRNA/rRNA cytosine-C5-methylase |
| TON_1939 | 1820723 | 1821805 | - | 3776.61 | 4102.86 | 4021.77 | 1.09 | 1.06 | small-conductance mechanosensitive channel |
| TON_1940 | 1821808 | 1822446 | - | 743.96 | 778.98 | 885.01 | 1.05 | 1.19 | hypothetical protein |
| TON_1941 | 1823067 | 1823729 | - | 897.30 | 561.39 | 953.20 | 0.63 | 1.06 | hypothetical protein |
| TON_1942 | 1823751 | 1824521 | - | 1720.77 | 772.97 | 1289.02 | 0.45 | 0.75 | metal-dependent phosphohydrolase |
| TON_1943 | 1824624 | 1825034 | - | 709.89 | 526.53 | 1005.99 | 0.74 | 1.42 | nucleic acid-binding protein |
| TON_1944 | 1825063 | 1826295 | - | 28481.91 | 32330.05 | 16215.40 | 1.14 | 0.57 | translation initiation factor IF-2 |
| TON_1945 | 1826415 | 1826792 | - | 22619.93 | 7530.13 | 37928.57 | 0.33 | 1.68 | 30S ribosomal protein S6e |
| TON_1946 | 1826937 | 1827107 | + | 288.50 | 474.84 | 327.75 | 1.65 | 1.14 | preprotein translocase subunit SecG |
| TON_1947 | 1827104 | 1827766 | - | 4048.08 | 4027.13 | 5255.07 | 0.99 | 1.30 | hypothetical protein |

| | | | | | | | | | |
|---|---|---|---|---|---|---|---|---|---|
| TON_1948 | 1827860 | 1828471 | + | 1040.41 | 246.44 | 805.82 | 0.24 | 0.77 | GTP-binding protein |
| TON_1949 | 1829046 | 1830113 | + | 1222.15 | 998.97 | 1090.31 | 0.82 | 0.89 | hypothetical protein |
| TON_1950 | 1830158 | 1830988 | + | 4953.33 | 6177.74 | 1371.88 | 1.25 | 0.28 | ubiA prenyltransferase UbiA-like protein |
| TON_1951 | 1831002 | 1831382 | + | 16797.69 | 14465.21 | 4974.97 | 0.86 | 0.30 | hypothetical protein |
| TON_1952 | 1831427 | 1832233 | - | 4602.36 | 6026.27 | 5634.15 | 1.31 | 1.22 | replication factor A complex, RPA32 subunit |
| TON_1953 | 1832230 | 1832607 | - | 6124.36 | 7209.17 | 9424.22 | 1.18 | 1.54 | replication factor A complex, RPA14 subunit |
| TON_1954 | 1832608 | 1833672 | - | 21262.62 | 17915.32 | 25745.20 | 0.84 | 1.21 | replication factor A |
| TON_1955 | 1833797 | 1834384 | - | 967.72 | 1383.65 | 1368.94 | 1.43 | 1.41 | putative transcriptional regulator |
| TON_1956 | 1835746 | 1836180 | + | 1475.43 | 2008.76 | 1394.61 | 1.36 | 0.95 | manganese-dependent transcription regulator |
| TON_1957 | 1836231 | 1837565 | - | 111.31 | 171.90 | 52.06 | 1.54 | 0.47 | xanthine/uracilpermease |
| TON_1958 | 1837698 | 1838702 | - | 458.87 | 479.65 | 199.44 | 1.05 | 0.43 | sugar-binding transport ATP-binding protein |
| TON_1959 | 1838696 | 1840315 | - | 162.42 | 164.69 | 70.39 | 1.01 | 0.43 | iron(III) ABC transporter permease |
| TON_1960 | 1840400 | 1841452 | + | 1228.96 | 1020.61 | 937.80 | 0.83 | 0.76 | D-aminopeptidase |
| TON_1961 | 1841442 | 1842287 | - | 5439.46 | 14441.17 | 11689.17 | 2.65 | 2.15 | endonuclease IV |
| TON_1962 | 1842330 | 1843391 | - | 3284.80 | 7024.04 | 23026.38 | 2.14 | 7.01 | hypothetical protein |
| TON_1963 | 1843369 | 1844502 | - | 5060.09 | 6766.78 | 20203.44 | 1.34 | 3.99 | membrane protein |
| TON_1964 | 1844495 | 1844797 | - | 790.53 | 1488.24 | 3238.68 | 1.88 | 4.10 | hypothetical protein |
| TON_1965 | 1844891 | 1845508 | + | 571.32 | 476.04 | 3985.84 | 0.83 | 6.98 | hypothetical protein |
| TON_1966 | 1845509 | 1846861 | - | 2093.32 | 1162.46 | 2656.50 | 0.56 | 1.27 | tRNA/rRNA cytosine-C5-methylase |
| TON_1967 | 1846966 | 1847520 | + | 386.18 | 473.64 | 480.27 | 1.23 | 1.24 | hypothetical protein |
| TON_1968 | 17841 | 18380 | + | 739.42 | 1464.19 | 854.95 | 1.98 | 1.16 | nicotinamidase-like amidase |
| TON_1969 | 66046 | 66423 | + | 9274.00 | 13046.70 | 14342.00 | 1.41 | 1.55 | ribonuclease P protein component 1 |
| TON_1970 | 432720 | 433067 | + | 645.15 | 974.93 | 497.13 | 1.51 | 0.77 | phospholipid-binding protein |
| TON_1971 | 462955 | 463527 | - | 940.46 | 1398.08 | 1605.04 | 1.49 | 1.71 | membrane protein |
| TON_1972 | 632410 | 633228 | + | 139.71 | 182.72 | 211.90 | 1.31 | 1.52 | SAM-dependent methyltransferase |
| TON_1973 | 1174861 | 1175694 | - | 5419.01 | 4286.79 | 4757.20 | 0.79 | 0.88 | glutamine amidotransferase |
| TON_1974 | 1187582 | 1188121 | + | 505.44 | 213.98 | 228.03 | 0.42 | 0.45 | hypothetical protein |
| TON_1975 | 1733339 | 1733761 | + | 1021.11 | 2381.42 | 808.75 | 2.33 | 0.79 | glycosyltransferase family protein |
| TON_1976 | 1828418 | 1828882 | - | 579.27 | 896.79 | 1102.05 | 1.55 | 1.90 | putative transcriptional regulator |
| TON_1977 | 1105064 | 1105189 | + | 3.41 | 0.00 | 5.13 | 0.00 | 1.51 | 7S ribosomal RNA |
| TON_1978 | 1150028 | 1150345 | + | 323.71 | 673.19 | 472.20 | 2.08 | 1.46 | 7S ribosomal RNA |
| TON_1979 | 1278100 | 1279595 | - | 914807.06 | 696223.59 | 187065.53 | 0.76 | 0.20 | 16S ribosomal RNA |

| | | | | | | | | | |
|---|---|---|---|---|---|---|---|---|---|
| TON_1980 | 1274841 | 1277847 | - | 15084033.26 | 12099865.45 | 3331711.04 | 0.80 | 0.22 | 23S ribosomal RNA |
| TON_1981 | 1834483 | 1834608 | + | 55.66 | 54.10 | 29.33 | 0.97 | 0.53 | 5S ribosomal RNA |
| TON_1982 | 92666 | 92743 | - | 365.74 | 75.73 | 6.60 | 0.21 | 0.02 | Arg tRNA |
| TON_1983 | 101858 | 101935 | - | 40.89 | 145.46 | 35.20 | 3.56 | 0.86 | Gly tRNA |
| TON_1984 | 83095 | 83181 | + | 552.01 | 224.80 | 1215.70 | 0.41 | 2.20 | Ser tRNA |
| TON_1985 | 85452 | 85539 | + | 612.21 | 602.27 | 1144.57 | 0.98 | 1.87 | Leu tRNA |
| TON_1986 | 87057 | 87134 | + | 1275.53 | 641.94 | 2306.01 | 0.50 | 1.81 | Pro tRNA |
| TON_1987 | 89022 | 89109 | + | 717.84 | 590.25 | 1297.82 | 0.82 | 1.81 | Leu tRNA |
| TON_1988 | 102041 | 102118 | + | 14.77 | 2.40 | 18.33 | 0.16 | 1.24 | Arg tRNA |
| TON_1989 | 158981 | 159056 | + | 18.17 | 7.21 | 5.87 | 0.40 | 0.32 | Arg tRNA |
| TON_1990 | 192832 | 192907 | + | 7.95 | 3.61 | 11.00 | 0.45 | 1.38 | Asn tRNA |
| TON_1991 | 192913 | 192990 | + | 29.53 | 14.43 | 38.13 | 0.49 | 1.29 | Met tRNA |
| TON_1992 | 207066 | 207151 | + | 81.78 | 43.28 | 43.99 | 0.53 | 0.54 | Ser tRNA |
| TON_1993 | 207172 | 207246 | + | 1206.24 | 116.61 | 190.64 | 0.10 | 0.16 | Cys tRNA |
| TON_1994 | 288355 | 288442 | + | 21.58 | 33.66 | 32.26 | 1.56 | 1.49 | Leu tRNA |
| TON_1995 | 323818 | 323895 | + | 84.05 | 25.24 | 45.46 | 0.30 | 0.54 | Ala tRNA |
| TON_1996 | 323906 | 323983 | + | 584.95 | 169.50 | 593.18 | 0.29 | 1.01 | Val tRNA |
| TON_1997 | 336950 | 337037 | + | 12.49 | 12.02 | 8.07 | 0.96 | 0.65 | Ser tRNA |
| TON_1998 | 474335 | 474412 | + | 110.17 | 34.86 | 695.10 | 0.32 | 6.31 | Met tRNA |
| TON_1999 | 574470 | 574546 | + | 78.37 | 24.04 | 103.39 | 0.31 | 1.32 | Val tRNA |
| TON_2000 | 626359 | 626436 | + | 157.88 | 50.49 | 44.73 | 0.32 | 0.28 | Glu tRNA |
| TON_2001 | 962981 | 963058 | + | 146.52 | 156.28 | 592.45 | 1.07 | 4.04 | Lys tRNA |
| TON_2002 | 1221460 | 1221547 | + | 10.22 | 8.41 | 37.39 | 0.82 | 3.66 | Leu tRNA |
| TON_2003 | 1240162 | 1240239 | + | 174.92 | 82.95 | 19.06 | 0.47 | 0.11 | Arg tRNA |
| TON_2004 | 1367760 | 1367846 | + | 1015.43 | 908.81 | 1219.36 | 0.90 | 1.20 | Xaa tRNA |
| TON_2005 | 1385181 | 1385258 | + | 48.84 | 60.11 | 31.53 | 1.23 | 0.65 | Gly tRNA |
| TON_2006 | 1499342 | 1499417 | + | 23.85 | 21.64 | 4.40 | 0.91 | 0.18 | Gln tRNA |
| TON_2007 | 1743948 | 1744025 | + | 39.75 | 22.84 | 50.59 | 0.57 | 1.27 | Glu tRNA |
| TON_2008 | 1744039 | 1744116 | + | 43.16 | 15.63 | 27.13 | 0.36 | 0.63 | Ile tRNA |
| TON_2009 | 1834615 | 1834692 | + | 477.05 | 78.14 | 160.58 | 0.16 | 0.34 | Asp tRNA |
| TON_2010 | 1835049 | 1835126 | + | 36.35 | 31.26 | 5.87 | 0.86 | 0.16 | Lys tRNA |
| TON_2011 | 1804765 | 1804842 | - | 109.04 | 48.09 | 236.83 | 0.44 | 2.17 | Pro tRNA |

| | | | | | | | | | |
|---|---|---|---|---|---|---|---|---|---|
| TON_2012 | 1803716 | 1803791 | - | 94.27 | 54.10 | 103.39 | 0.57 | 1.10 | Gln tRNA |
| TON_2013 | 1405018 | 1405094 | - | 12876.83 | 1643.31 | 12338.81 | 0.13 | 0.96 | His tRNA |
| TON_2014 | 1401128 | 1401205 | - | 604.26 | 105.79 | 43.99 | 0.18 | 0.07 | Tyr tRNA |
| TON_2015 | 1340837 | 1340914 | - | 26.12 | 21.64 | 15.40 | 0.83 | 0.59 | Ala tRNA |
| TON_2016 | 1277980 | 1278056 | - | 893.89 | 1710.63 | 962.73 | 1.91 | 1.08 | Ala tRNA |
| TON_2017 | 1240854 | 1240931 | - | 27.26 | 14.43 | 52.79 | 0.53 | 1.94 | Val tRNA |
| TON_2018 | 1148129 | 1148205 | - | 24.99 | 26.45 | 46.93 | 1.06 | 1.88 | Phe tRNA |
| TON_2019 | 1148039 | 1148116 | - | 29.53 | 32.46 | 15.40 | 1.10 | 0.52 | Gly tRNA |
| TON_2020 | 965863 | 965970 | - | 219.21 | 32.46 | 324.82 | 0.15 | 1.48 | Xaa tRNA |
| TON_2021 | 961960 | 962047 | - | 831.42 | 384.68 | 698.77 | 0.46 | 0.84 | Leu tRNA |
| TON_2022 | 886650 | 886726 | - | 6.81 | 7.21 | 0.73 | 1.06 | 0.11 | Arg tRNA |
| TON_2023 | 699691 | 699777 | - | 120.40 | 19.23 | 58.66 | 0.16 | 0.49 | Ser tRNA |
| TON_2024 | 674095 | 674171 | - | 9.09 | 7.21 | 0.73 | 0.79 | 0.08 | Thr tRNA |
| TON_2025 | 192581 | 192657 | - | 0.00 | 1.20 | 1.47 | - | - | Thr tRNA |
| TON_2026 | 146734 | 146810 | - | 18.17 | 44.48 | 38.86 | 2.45 | 2.14 | Thr tRNA |
| TON_2027 | 146629 | 146706 | - | 1585.61 | 437.57 | 733.96 | 0.28 | 0.46 | Thr tRNA |

**Supplementary Table 5.** The list of non-coding RNAs

| Gene | TSS position | Strand | TSS abundance | Category | Located | Normalized mRNA expression | | |
|---|---|---|---|---|---|---|---|---|
| | | | | | | YPS | MMC | MMF |
| TON_nc001 | 36882 | + | 200 | N | TON_0041 / TON_0042 | 276.8556165 | 489.0442927 | 268.9715698 |
| TON_nc002 | 81023 | + | 62 | N | TON_0098 / TON_0099 | 130.5498029 | 408.339939 | 153.6980399 |
| TON_nc003 | 112251 | + | 6 | N | TON_0140 / TON_0141 | 43.89174407 | 28.90902223 | 21.42905364 |
| TON_nc004 | 112601 | + | 102 | N | TON_0140 / TON_0141 | 1150.188781 | 262.5902852 | 234.2417242 |
| TON_nc005 | 133450 | + | 549 | N | TON_0156 / TON_0157 | 184.570411 | 271.0220834 | 233.5027914 |
| TON_nc006 | 159404 | - | -95 | N | TON_1989 / TON_0184 | 74.27833612 | 34.93173519 | 20.69012075 |
| TON_nc007 | 171388 | + | 17 | A | TON_0192 | 12.37972269 | 8.43179815 | 39.90237574 |
| TON_nc008 | 174325 | + | 363 | N | TON_0196 / TON_0197 | 2300.377561 | 1080.474706 | 551.2439315 |
| TON_nc009 | 209468 | - | -9 | A | TON_0230 | 42.76631474 | 19.27268149 | 19.95118787 |
| TON_nc010 | 211611 | + | 247 | N | TON_0231 / TON_0232 | 961.1166523 | 693.8165335 | 485.4789048 |
| TON_nc011 | 278589 | + | 21 | A | TON_0309 | 69.77661878 | 36.13627779 | 71.67648975 |
| TON_nc012 | 295789 | - | -73 | N | TON_0319 / TON_0320 | 9947.669894 | 3100.492634 | 705.6809043 |
| TON_nc013 | 334794 | - | -18 | A | TON_0360 | 181.194123 | 28.90902223 | 21.42905364 |
| TON_nc014 | 334908 | - | -4 | A | TON_0360 | 534.5789342 | 89.13615187 | 97.53914069 |
| TON_nc015 | 366125 | + | 24 | N | TON_0394 / TON_0395 | 156.4346776 | 128.8860574 | 255.6707779 |
| TON_nc016 | 366207 | + | 4 | N | TON_0394 / TON_0395 | 618.9861344 | 140.9314834 | 748.5390115 |
| TON_nc017 | 396068 | - | -5 | A | TON_0423 | 177.817835 | 140.9314834 | 90.88874474 |
| TON_nc018 | 397415 | - | -15 | A | TON_0424 | 96.78692283 | 25.29539445 | 42.85810727 |
| TON_nc019 | 424692 | - | -25 | N | TON_0455 / TON_0456 | 230.7130137 | 72.27255557 | 11.82292614 |
| TON_nc020 | 459096 | - | -21 | N | TON_0500 / TON_0501 | 2633.504644 | 2799.356986 | 933.2722325 |
| TON_nc021 | 504419 | - | -77 | N | TON_0548 / TON_0549 | 124.9226562 | 114.4315463 | 266.0158383 |
| TON_nc022 | 508051 | + | 355 | N | TON_0552 / TON_0553 | 47.26803208 | 31.31810741 | 33.25197978 |
| TON_nc023 | 515633 | + | 15 | A | TON_0561 | 28.13573338 | 66.24984261 | 39.90237574 |
| TON_nc024 | 544214 | - | -304 | N | TON_0592 / TON_0593 | 157.5601069 | 340.8855538 | 217.2462679 |
| TON_nc025 | 645021 | - | -8 | N | TON_0692 / TON_0693 | 1928.985881 | 1149.133634 | 823.1712328 |
| TON_nc026 | 695581 | - | -327 | N | TON_0746 / TON_0747 | 12985.20367 | 2993.288343 | 4995.186296 |
| TON_nc027 | 697194 | - | -2 | N | TON_0748 / TON_0749 | 58.52232543 | 79.49981113 | 16.25652345 |
| TON_nc028 | 701558 | - | -54 | N | TON_0752 / TON_0753 | 9145.238778 | 3476.309923 | 13205.46957 |
| TON_nc029 | 728579 | + | 702 | N | TON_0782 / TON_0783 | 1467.559853 | 522.7714853 | 140.397248 |

| | | | | | | | | |
|---|---|---|---|---|---|---|---|---|
| TON_nc030 | 756952 | + | 11 | N | TON_0814 / TON_0815 | 64.14947211 | 122.8633445 | 42.11917439 |
| TON_nc031 | 763651 | - | -9 | A | TON_0822 | 74.27833612 | 97.56795002 | 122.6628588 |
| TON_nc032 | 780664 | + | 14 | N | TON_0841 / TON_0842 | 41.6408854 | 15.65905371 | 13.30079191 |
| TON_nc033 | 802315 | - | -5 | A | TON_0869 | 289.2353392 | 2494.60771 | 532.0316765 |
| TON_nc034 | 818787 | + | 155 | N | TON_0891 / TON_0892 | 2360.025316 | 855.2252409 | 1773.438922 |
| TON_nc035 | 886393 | + | 7 | N | TON_0962 / TON_2022 | 347.7576646 | 257.7721149 | 29.55731536 |
| TON_nc036 | 900486 | - | -9 | A | TON_0978 | 57.3968961 | 28.90902223 | 36.9466442 |
| TON_nc037 | 918382 | + | 2 | A | TON_0991 | 39.39002673 | 19.27268149 | 16.25652345 |
| TON_nc038 | 928675 | + | 21 | N | TON_1004 / TON_1005 | 82.15634147 | 8.43179815 | 10.34506038 |
| TON_nc039 | 938482 | + | 583 | N | TON_1015 / TON_1016 | 4186.597127 | 2127.222219 | 704.9419714 |
| TON_nc040 | 956993 | - | -3 | A | TON_1035 | 94.53606416 | 36.13627779 | 49.50850323 |
| TON_nc041 | 972916 | - | -254407 | N | TON_1052 / TON_1053 | 17359.7475 | 32221.51436 | 17502.36429 |
| TON_nc042 | 991268 | + | 1342 | A | TON_1073 | 56.27146676 | 71.06801298 | 42.11917439 |
| TON_nc043 | 998003 | - | -12 | N | TON_1077 / TON_1078 | 10536.26944 | 3484.741721 | 1594.617164 |
| TON_nc044 | 998043 | + | 6 | N | TON_1077 / TON_1078 | 69.77661878 | 20.47722408 | 28.07944959 |
| TON_nc045 | 999076 | + | 77 | N | TON_1078 / TON_1079 | 163.1872536 | 162.61325 | 83.4994159 |
| TON_nc046 | 1071899 | + | 2 | N | TON_1174 / TON_1175 | 129.4243736 | 57.81804446 | 104.9284695 |
| TON_nc047 | 1083461 | + | 15 | A | TON_1186 | 186.8212696 | 62.63621483 | 26.60158383 |
| TON_nc048 | 1111564 | - | -6 | A | TON_1213 | 101.2886402 | 61.43167224 | 79.06581859 |
| TON_nc049 | 1143589 | + | 20 | N | TON_1256 / TON_1257 | 38.2645974 | 77.09072594 | 28.07944959 |
| TON_nc050* | 1150345 | - | -27 | P | TON_1264/TON_1265 | 510244.9011 | 178531.2804 | 278584.3477 |
| TON_nc051 | 1163802 | + | 14 | N | TON_1279 / TON_1280 | 9.003434682 | 20.47722408 | 8.128261724 |
| TON_nc052 | 1208556 | - | -657 | N | TON_1335 / TON_1336 | 398.4019847 | 325.2265001 | 1408.406077 |
| TON_nc053 | 1221950 | - | -20 | N | TON_2002 / TON_1353 | 405.1545607 | 204.7722408 | 472.9170458 |
| TON_nc054 | 1223126 | - | -519 | A | TON_1353 | 151.9329603 | 154.1814519 | 129.3132547 |
| TON_nc055 | 1246715 | - | -114 | N | TON_1377 / TON_1378 | 1917.731587 | 685.3847353 | 2234.533041 |
| TON_nc056 | 1248212 | - | -665 | A | TON_1379 | 467.0531741 | 778.134515 | 47.29170458 |
| TON_nc057 | 1253617 | - | -148 | N | TON_1380 / TON_1381 | 1906.477294 | 381.8400019 | 207.6401404 |
| TON_nc058 | 1279837 | - | -2 | N | TON_1979/TON_1402 | 561.5892383 | 872.0888372 | 847.556018 |
| TON_nc059 | 1296090 | + | 103 | N | TON_1419 / TON_1420 | 48.39346141 | 27.70447964 | 21.42905364 |
| TON_nc060 | 1311680 | + | 66 | N | TON_1433 / TON_1434 | 388.2731207 | 319.2037871 | 48.76957035 |
| TON_nc061 | 1312269 | + | 7 | A | TON_1435 | 1993.135353 | 119.2497167 | 227.5913283 |

| | | | | | | | | |
|---|---|---|---|---|---|---|---|---|
| TON_nc062 | 1339334 | + | 8902 | N | TON_1461 / TON_1462 | 7.878005347 | 12.04542593 | 2.216798652 |
| TON_nc063 | 1361686 | + | 221 | N | TON_1487 / TON_1488 | 27.01030405 | 15.65905371 | 10.34506038 |
| TON_nc064 | 1364605 | + | 87 | N | TON_1492 / TON_1493 | 535.7043636 | 314.3856167 | 720.4595619 |
| TON_nc065 | 1388024 | + | 27 | N | TON_1512 / TON_1513 | 6816.725483 | 2188.653891 | 1729.102949 |
| TON_nc066 | 1421328 | + | 9 | A | TON_1547 | 1969.501337 | 796.2026539 | 710.1145016 |
| TON_nc067 | 1430266 | + | 58 | A | TON_1558 | 292.6116272 | 1198.51988 | 2208.67039 |
| TON_nc068 | 1466441 | + | 4 | A | TON_1604 | 40.51545607 | 13.24996852 | 8.128261724 |
| TON_nc069 | 1493357 | + | 28 | A | TON_1633 | 72.02747745 | 59.02258705 | 19.95118787 |
| TON_nc070 | 1510583 | - | -1466 | N | TON_1645 / TON_1646 | 169.9398296 | 142.136026 | 19.21225498 |
| TON_nc071 | 1527871 | - | -10 | A | TON_1664 | 31.51202139 | 19.27268149 | 26.60158383 |
| TON_nc072 | 1528990 | - | -21 | A | TON_1664 | 192.4484163 | 115.6360889 | 174.3881606 |
| TON_nc073 | 1563811 | - | -71 | A | TON_1698 | 75.40376546 | 33.7271926 | 11.82292614 |
| TON_nc074 | 1574714 | + | 2993 | N | TON_1708 / TON_1709 | 28.13573338 | 55.40895927 | 15.51759056 |
| TON_nc075 | 1574802 | + | 4588 | N | TON_1708 / TON_1709 | 68.65118945 | 225.2494649 | 25.12371806 |
| TON_nc076 | 1602488 | - | -5 | A | TON_1734 | 127.1735149 | 42.15899075 | 59.11463072 |
| TON_nc077 | 1641435 | + | 46 | N | TON_1775 / TON_1776 | 80892.48433 | 39587.29231 | 44282.76987 |
| TON_nc078 | 1649856 | + | 45 | N | TON_1783 / TON_1784 | 131.6752322 | 130.0906 | 82.76048301 |
| TON_nc079 | 1697190 | + | 28 | A | TON_1820 | 79.9054828 | 89.13615187 | 52.46423477 |
| TON_nc080 | 1702890 | - | -33 | N | TON_1823 / TON_1824 | 1115.300471 | 1135.883665 | 999.7761921 |
| TON_nc081 | 1709927 | - | -277 | A | TON_1825 | 28.13573338 | 2.409085186 | 10.34506038 |
| TON_nc082 | 1724083 | - | -8 | N | TON_1840 / TON_1841 | 965.6183696 | 1592.405308 | 1610.134754 |
| TON_nc083 | 1724170 | + | 3 | N | TON_1840 / TON_1841 | 29.26116272 | 15.65905371 | 17.73438922 |
| TON_nc084 | 1724396 | - | -11 | N | TON_1840 / TON_1841 | 910.4723322 | 1100.95193 | 1682.550177 |
| TON_nc085 | 1737498 | + | 45 | N | TON_1855 / TON_1856 | 28.13573338 | 34.93173519 | 11.82292614 |
| TON_nc086 | 1781374 | - | -109 | N | TON_1894 / TON_1895 | 213.8315737 | 397.4990556 | 182.5164224 |
| TON_nc087 | 1803350 | - | -90 | N | TON_1916 / TON_1917 | 749.5359373 | 457.7261853 | 490.651435 |

*In our study, the direction of genome annotation of TON_1978 was proven as reverse strand, unlikely annotated as positive strand in current NCBI genome database. Although TON_nc050 corresponding in reverse strand of TON_1978 was classified to the ncRNAs (Primary) here, we did not count TON_nc050 in the number of total 86 ncRNAs .

**Supplementary Table 6.** The function of ncRNAs from Rfam database in *T. onnurineus* NA1

| RNA name | Accession | Query name | Mdl | Score | E-value | Description |
|---|---|---|---|---|---|---|
| sR58 | RF01308 | TON_nc005 | cm | 71.1 | 9.00E-18 | Small nucleolar RNA sR58 |
| sR34 | RF01273 | TON_nc008 | cm | 76.5 | 1.20E-15 | Small nucleolar RNA sR34 |
| CRISPR-DR6 | RF01319 | TON_nc012 | cm | 39.4 | 1.10E-06 | CRISPR RNA direct repeat element |
| HgcF | RF00058 | TON_nc021 | cm | 112.8 | 1.00E-27 | HgcF RNA |
| sR28 | RF01136 | TON_nc024 | cm | 57.3 | 5.50E-15 | Small nucleolar RNA sR28 |
| HgcG | RF00064 | TON_nc025 | cm | 180.6 | 1.50E-53 | HgcG RNA |
| snoPyro_CD | RF00095 | TON_nc026 | cm | 45.6 | 1.10E-09 | Pyrococcus C/D box small nucleolar RNA |
| SscA | RF00063 | TON_nc028 | cm | 75.4 | 1.90E-14 | SscA RNA |
| CRISPR-DR6 | RF01319 | TON_nc029 | cm | 48.2 | 3.20E-09 | CRISPR RNA direct repeat element |
| tRNA | RF00005 | TON_nc035 | cm | 74.2 | 1.10E-11 | tRNA |
| CRISPR-DR6 | RF01319 | TON_nc043 | cm | 48.4 | 5.90E-09 | CRISPR RNA direct repeat element |
| sR39 | RF01122 | TON_nc045 | cm | 69.2 | 4.20E-17 | Small nucleolar RNA sR39 |
| snoR9 | RF00065 | TON_nc046 | cm | 98.2 | 1.80E-24 | Small nucleolar RNA snoR9 |
| Archaea_SRP | RF01857 | TON_nc050 | cm | 220.8 | 2.70E-26 | Archaeal signal recognition particle RNA |
| sR55 | RF01307 | TON_nc055 | cm | 56.6 | 6.80E-15 | Small nucleolar RNA sR55 |
| sR5 | RF01304 | TON_nc059 | cm | 49.2 | 2.20E-11 | Small nucleolar RNA sR5 |
| snoPyro_CD | RF00095 | TON_nc062 | cm | 49.9 | 8.10E-11 | Pyrococcus C/D box small nucleolar RNA |
| sR22 | RF01275 | TON_nc064 | cm | 44.2 | 1.00E-09 | Small nucleolar RNA sR22 |
| sR1 | RF01152 | TON_nc074 | cm | 49.4 | 1.30E-12 | Small nucleolar RNA sR1 |
| sR60 | RF01309 | TON_nc075 | cm | 60.5 | 1.90E-15 | Small nucleolar RNA sR60 |
| RNaseP_arch | RF00373 | TON_nc077 | cm | 190.6 | 1.00E-27 | Archaeal RNase P |
| sR43 | RF01128 | TON_nc078 | cm | 67.3 | 4.30E-11 | Small nucleolar RNA sR43 |

**Supplementary Table 7.** BlastP result of *T. kodakarensis* for *T. onnurineus in coding region**

| Query id | Subject id | % identity | e-value | bit score | Function |
|---|---|---|---|---|---|
| TON_0002 | TK0110 | 73.85 | 3.00E-89 | 320 | hydrolase |
| TON_0003 | TK0109 | 67.26 | 1.00E-116 | 412 | hypothetical protein |
| TON_0004 | TK0108 | 62.78 | 5.00E-61 | 226 | membrane protein |
| TON_0006 | TK1992 | 59.48 | 5.00E-125 | 439 | major facilitator superfamily permease |
| TON_0007 | TK0155 | 91.63 | 0 | 1410 | RecJ-like exonuclease |
| TON_0008 | TK1658 | 66.27 | 4.00E-23 | 98.2 | hypothetical protein |
| TON_0009 | TK1657 | 84.48 | 1.00E-14 | 70.1 | hypothetical protein |
| TON_0010 | TK1655 | 76.78 | 3.00E-153 | 533 | Permease |
| TON_0012 | TK1652 | 73.91 | 6.00E-53 | 198 | thioesterase |
| TON_0013 | TK1651 | 66.15 | 2.00E-33 | 133 | hypothetical protein |
| TON_0016 | TK1868 | 35.2 | 1.00E-33 | 135 | molybdenum-pterin binding domain-containing protein |
| TON_1968 | TK1650 | 82.02 | 2.00E-88 | 317 | nicotinamidase-like amidase |
| TON_0017 | TK1649 | 59.65 | 3.00E-113 | 400 | membrane protein |
| TON_0018 | TK1648 | 45.71 | 2.00E-20 | 89.4 | hypothetical protein |
| TON_0019 | TK1646 | 78.85 | 5.00E-100 | 355 | hypothetical protein |
| TON_0020 | TK1643 | 88.35 | 0 | 1096 | indolepyruvate: ferredoxin oxidoreductase subunit alpha |
| TON_0022 | TK1642 | 37.35 | 1.00E-13 | 67 | membrane protein |
| TON_0023 | TK1641 | 69.47 | 4.00E-35 | 138 | hypothetical protein |
| TON_0024 | TK1640 | 79.61 | 2.00E-36 | 142 | nucleotide pyrophosphohydrolase |
| TON_0025 | TK1639 | 68.71 | 5.00E-50 | 189 | bis(5'-adenosyl)-triphosphatase |
| TON_0026 | TK1638 | 49.32 | 1.00E-11 | 60.1 | membrane protein |
| TON_0027 | TK1637 | 89.36 | 3.00E-115 | 406 | proteasome subunit alpha |
| TON_0028 | TK1636 | 94.07 | 3.00E-130 | 456 | putative RNA-associated protein |
| TON_0029 | TK1635 | 86.33 | 2.00E-119 | 421 | exosome complex RNA-binding protein Rrp4 |
| TON_0030 | TK1634 | 93.98 | 3.00E-136 | 476 | exosome complex exonuclease Rrp41 |
| TON_0031 | TK1633 | 80.37 | 3.00E-127 | 446 | exosome complex RNA-binding protein Rrp42 |
| TON_0033 | TK1631 | 72.46 | 1.00E-46 | 177 | hypothetical protein |
| TON_0034 | TK1630 | 86.53 | 2.00E-98 | 350 | hypothetical protein |
| TON_0035 | TK1629 | 94.25 | 4.00E-94 | 335 | hypothetical protein |
| TON_0036 | TK1627 | 74.02 | 2.00E-146 | 511 | homoserine dehydrogenase |
| TON_0037 | TK1626 | 86.07 | 5.00E-130 | 456 | hypothetical protein |
| TON_0038 | TK1625 | 81.28 | 1.00E-161 | 561 | methylmalonyl-CoA decarboxylase beta chain |
| TON_0039 | TK1624 | 73.25 | 2.00E-41 | 160 | putative acetyl-CoA carboxylase biotin carboxyl carrier protein subunit |
| TON_0040 | TK1623 | 73.77 | 8.00E-41 | 157 | methylmalonyl-CoA decarboxylase subunit delta |
| TON_0041 | TK1622 | 96.55 | 0 | 1031 | methylmalonyl-CoA decarboxylase subunit alpha |
| TON_0042 | TK1621 | 91.61 | 1.00E-74 | 270 | translation initiation factor IF-2 |
| TON_0044 | TK1619 | 76.22 | 6.00E-54 | 202 | hypothetical protein |
| TON_0045 | TK1618 | 84.71 | 3.00E-84 | 303 | metallophosphoesterase |
| TON_0046 | TK1617 | 64.06 | 2.00E-90 | 324 | hypothetical protein |
| TON_0047 | TK1616 | 60.71 | 1.00E-59 | 221 | hypothetical protein |
| TON_0048 | TK1615 | 82.72 | 2.00E-159 | 554 | RNA 3'-terminal-phosphate cyclase |
| TON_0050 | TK1611 | 88.57 | 6.00E-149 | 518 | metal-dependent hydrolase |
| TON_0051 | TK1610 | 59.77 | 2.00E-23 | 99.8 | hypothetical protein |
| TON_0058 | TK1550 | 71.29 | 1.00E-126 | 445 | asparagine synthetase A |
| TON_0059 | TK1549 | 66.84 | 5.00E-73 | 265 | ATPase |

| | | | | | |
|---|---|---|---|---|---|
| TON_0061 | TK1548 | 93.73 | 0 | 743 | serine-glyoxylate aminotransferase |
| TON_0062 | TK1547 | 74.23 | 1.00E-160 | 558 | major facilitator superfamily permease |
| TON_0063 | TK1546 | 95.03 | 1.00E-100 | 357 | rpl10e 50S ribosomal protein L10e |
| TON_0064 | TK1545 | 85 | 0 | 654 | hypothetical protein |
| TON_0065 | TK1543 | 87.11 | 4.00E-125 | 439 | hypothetical protein |
| TON_0066 | TK1542 | 93.08 | 2.00E-178 | 617 | rpl3p 50S ribosomal protein L3P |
| TON_0067 | TK1541 | 95.29 | 6.00E-119 | 419 | rpl4lp 50S ribosomal protein L4P |
| TON_0068 | TK1540 | 87.21 | 6.00E-40 | 154 | rplW 50S ribosomal protein L23 |
| TON_0069 | TK1539 | 92.05 | 2.00E-125 | 440 | rpl2p 50S ribosomal protein L2 |
| TON_0070 | TK1538 | 93.23 | 6.00E-63 | 231 | rps19p 30S ribosomal protein S19 |
| TON_0071 | TK1537 | 96.15 | 1.00E-74 | 270 | rpl22p 50S ribosomal protein L22 |
| TON_0072 | TK1536 | 94.76 | 2.00E-83 | 300 | rps3p 30S ribosomal protein S3 |
| TON_0073 | TK1535 | 93.94 | 3.00E-29 | 118 | 50S ribosomal protein L29 |
| TON_0074 | TK1534 | 90.82 | 2.00E-42 | 162 | translation initiation factor Sui1 |
| TON_1969 | TK1533 | 77.6 | 7.00E-55 | 204 | ribonuclease P protein component 1 |
| TON_0075 | TK1532 | 81.65 | 3.00E-52 | 195 | rps17p 30S ribosomal protein S17 |
| TON_0076 | TK1531 | 97.87 | 1.00E-56 | 210 | rpl14p 50S ribosomal protein L14 |
| TON_0077 | TK1530 | 84.4 | 4.00E-51 | 192 | rpl24p 50S ribosomal protein L24 |
| TON_0078 | TK1529 | 88.89 | 1.00E-129 | 454 | 30S ribosomal protein S4e |
| TON_0079 | TK1528 | 91.26 | 3.00E-96 | 342 | rpl5p 50S ribosomal protein L5 |
| TON_0080 | TK1527 | 96.43 | 1.00E-27 | 113 | rps14P 30S ribosomal protein S14 |
| TON_0081 | TK1526 | 96.15 | 6.00E-70 | 254 | rps8p 30S ribosomal protein S8 |
| TON_0082 | TK1525 | 91.3 | 1.00E-95 | 340 | rpl6p 50S ribosomal protein L6 |
| TON_0083 | TK1524 | 95.24 | 8.00E-55 | 204 | rpl32e 50S ribosomal protein L32e |
| TON_0084 | TK1523 | 91.89 | 1.00E-76 | 277 | rpl19e 50S ribosomal protein L19e |
| TON_0085 | TK1522 | 93.03 | 3.00E-93 | 333 | rpl18p 50S ribosomal protein L18 |
| TON_0086 | TK1521 | 94.47 | 2.00E-129 | 453 | rps5p 30S ribosomal protein S5 |
| TON_0087 | TK1520 | 92.9 | 2.00E-82 | 296 | rpl30p 50S ribosomal protein L30 |
| TON_0088 | TK1519 | 84.46 | 8.00E-49 | 184 | rpl15p 50S ribosomal protein L15 |
| TON_0089 | TK1518 | 84.02 | 0 | 745 | preprotein translocase subunit SecY |
| TON_0090 | TK1517 | 85.23 | 3.00E-71 | 260 | adenylate kinase |
| TON_0091 | TK1516 | 64 | 4.00E-48 | 182 | membrane protein |
| TON_0092 | TK1515 | 93.26 | 2.00E-45 | 172 | rpl34e 50S ribosomal protein L34e |
| TON_0093 | TK1514 | 87.82 | 1.00E-100 | 357 | cytidylate kinase |
| TON_0094 | TK1513 | 90.36 | 5.00E-29 | 117 | 50S ribosomal protein L14e |
| TON_0095 | TK1512 | 57.34 | 3.00E-67 | 246 | short-chain alcohol dehydrogenase |
| TON_0096 | TK1509 | 93.45 | 3.00E-180 | 623 | H/ACA RNA-protein complex component Cbf5p |
| #N/A | TK1507 | 89.74 | 6.00E-103 | 365 | #N/A |
| TON_0102 | TK1506 | 92.62 | 2.00E-70 | 256 | rps13p 30S ribosomal protein S13 |
| TON_0103 | TK1505 | 91.53 | 2.00E-68 | 250 | rps4p 30S ribosomal protein S4 |
| TON_0104 | TK1504 | 92.42 | 2.00E-39 | 154 | rps11p 30S ribosomal protein S11 |
| TON_0105 | TK1503 | 87.94 | 6.00E-132 | 462 | DNA-directed RNA polymerase subunit D |
| TON_0106 | TK1502 | 90.83 | 9.00E-60 | 220 | 50S ribosomal protein L18e |
| TON_0107 | TK1501 | 96.48 | 4.00E-77 | 278 | rpl13p 50S ribosomal protein L13 |
| TON_0108 | TK1500 | 98.52 | 3.00E-66 | 242 | rps9p 30S ribosomal protein S9 |
| TON_0109 | TK1499 | 92.31 | 1.00E-30 | 123 | DNA-directed RNA polymerase subunit N |
| TON_0110 | TK1498 | 87.72 | 2.00E-25 | 105 | rpoK DNA-directed RNA polymerase subunit K |
| TON_0111 | TK1497 | 83.28 | 1.00E-161 | 561 | hypothetical protein |

| | | | | | |
|---|---|---|---|---|---|
| TON_0112 | TK1496 | 94.03 | 9.00E-113 | 397 | rps2P 30S ribosomal protein S2 |
| TON_0113 | TK1495 | 93.88 | 6.00E-22 | 94.4 | rpl40e 50S ribosomal protein L40e |
| TON_0114 | TK1494 | 83.18 | 3.00E-48 | 182 | transcription regulator, PadR-like family |
| TON_0115 | TK1493 | 70.71 | 3.00E-37 | 145 | hypothetical protein |
| TON_0117 | TK1492 | 83.79 | 1.00E-110 | 391 | hypothetical protein |
| TON_0118 | TK1491 | 83.67 | 2.00E-69 | 253 | Lrp/AsnC family transcriptional regulator |
| TON_0119 | TK1490 | 57.41 | 8.00E-33 | 130 | hypothetical protein |
| TON_0120 | TK1489 | 83.12 | 2.00E-86 | 310 | adenine phosphoribosyltransferase |
| TON_0121 | TK1488 | 75 | 2.00E-74 | 270 | hypothetical protein |
| TON_0122 | TK1487 | 70.51 | 5.00E-28 | 114 | Lrp/AsnC family transcription regulator |
| TON_0123 | TK1486 | 88.84 | 0 | 788 | signal recognition particle protein Srp54 |
| TON_0125 | TK0380 | 60.78 | 3.00E-95 | 340 | hypothetical protein |
| TON_0127 | TK1483 | 49.65 | 2.00E-100 | 358 | hypothetical protein |
| TON_0128 | TK1482 | 90.26 | 3.00E-144 | 503 | 5'-methylthioadenosine phosphorylase |
| TON_0129 | TK1481 | 83.78 | 0 | 707 | NADH:polysulfide oxidoreductase |
| TON_0131 | TK1479 | 92.67 | 2.00E-144 | 504 | uridine phosphorylase |
| TON_0132 | TK1477 | 81.44 | 2.00E-145 | 506 | hypothetical protein |
| TON_0133 | TK1474 | 88.22 | 7.00E-152 | 528 | mevalonate kinase |
| TON_0134 | TK1473 | 64.12 | 3.00E-95 | 340 | amino acid kinase |
| TON_0135 | TK1470 | 93.85 | 0 | 722 | isopentenyl pyrophosphate isomerase |
| TON_0136 | TK1469 | 90.54 | 0 | 833 | hydrolase |
| TON_0137 | TK1468 | 85.42 | 3.00E-166 | 576 | bifunctional short-chain isoprenyl diphosphate synthase |
| TON_0140 | TK1464 | 82.33 | 2.00E-122 | 431 | hypothetical protein |
| TON_0141 | TK1461 | 93.07 | 0 | 1794 | leuS leucyl-tRNA synthetase |
| TON_0143 | TK1455 | 74.79 | 1.00E-161 | 561 | aminopeptidase P |
| TON_0144 | TK1454 | 93.3 | 2.00E-81 | 293 | 50S ribosomal protein L15e |
| TON_0145 | TK1451 | 85.43 | 1.00E-73 | 267 | hypothetical protein |
| TON_0146 | TK1450 | 77.1 | 9.00E-96 | 341 | ribonuclease P protein component 3 |
| TON_0147 | TK1442 | 90.66 | 0 | 757 | tryptophan synthase subunit beta |
| TON_0149 | TK1440 | 62.16 | 6.00E-39 | 152 | hypothetical protein |
| TON_0150 | TK1439 | 86.23 | 1.00E-67 | 247 | nickel responsive regulator |
| TON_0152 | TK1438 | 81.54 | 0 | 932 | fibronectin-binding protein |
| TON_0154 | TK1435 | 76.19 | 2.00E-28 | 116 | nitrogen regulatory protein P-II |
| TON_0155 | TK1434 | 44.67 | 1.00E-36 | 145 | alpha-glucosidase |
| TON_0156 | TK1432 | 59.2 | 1.00E-176 | 611 | sodium-dependent transporter |
| TON_0157 | TK1431 | 85.04 | 0 | 679 | glutamate dehydrogenase |
| TON_0163 | TK1430 | 91.19 | 0 | 698 | hypothetical protein |
| TON_0164 | TK1429 | 78.33 | 2.00E-90 | 323 | proteasome subunit beta |
| TON_0165 | TK1428 | 91.98 | 0 | 1214 | cleavage and polyadenylation specficity factor subunit-like protein |
| TON_0167 | TK1427 | 62.83 | 1.00E-40 | 157 | membrane protein |
| TON_0168 | TK1426 | 89.43 | 4.00E-103 | 366 | ribose-5-phosphate isomerase A |
| TON_0169 | TK1425 | 86.43 | 9.00E-99 | 351 | hypothetical protein |
| TON_0170 | TK1424 | 70.4 | 3.00E-163 | 567 | hypothetical protein |
| TON_0172 | TK0937 | 78.12 | 0 | 1080 | TRAP-type transporter |
| TON_0173 | TK0936 | 58.27 | 2.00E-40 | 157 | hypothetical protein |
| TON_0174 | TK0935 | 85.76 | 4.00E-145 | 506 | TRAP-type transporter |
| TON_0175 | TK1422 | 83.46 | 4.00E-139 | 486 | hypothetical protein |

| | | | | | |
|---|---|---|---|---|---|
| TON_0176 | TK1421 | 90.45 | 0 | 631 | cell division protein FtsZ |
| TON_0178 | TK1419 | 85.53 | 2.00E-72 | 263 | nusG transcription antitermination protein NusG |
| TON_0179 | TK1418 | 91.41 | 1.00E-71 | 260 | rpl11p 50S ribosomal protein L11 |
| TON_0180 | TK1417 | 95.37 | 1.00E-101 | 361 | rpl1P 50S ribosomal protein L1P |
| TON_0181 | TK1416 | 91.76 | 6.00E-150 | 522 | rplP0 acidic ribosomal protein P0 |
| TON_0184 | TK1414 | 84.42 | 8.00E-75 | 271 | dcd deoxycytidine triphosphate deaminase |
| TON_0185 | TK1413 | 87.88 | 2.00E-28 | 115 | histone A |
| TON_0186 | TK1412 | 89.39 | 0 | 800 | hypothetical protein |
| TON_0187 | TK1411 | 81.95 | 7.00E-59 | 218 | hypothetical protein |
| TON_0188 | TK1410 | 82.94 | 0 | 693 | DNA primase |
| TON_0189 | TK1408 | 87.92 | 0 | 988 | gltX glutamyl-tRNA synthetase |
| TON_0190 | TK1407 | 58.97 | 4.00E-141 | 493 | membrane protein |
| TON_0191 | TK1406 | 82.43 | 0 | 1382 | maltodextrin phosphorylase |
| TON_0192 | TK1405 | 88.6 | 0 | 1098 | phosphoenolpyruvate carboxykinase |
| TON_0195 | TK1404 | 81.55 | 3.00E-109 | 386 | phosphomannomutase |
| TON_0196 | TK1403 | 82.91 | 1.00E-97 | 347 | tmk thymidylate kinase |
| TON_0197 | TK1401 | 82.53 | 3.00E-137 | 479 | hydrolase |
| TON_0198 | TK1400 | 85.45 | 0 | 671 | permease |
| TON_0199 | TK1399 | 79.74 | 0 | 659 | permease |
| TON_0200 | TK1398 | 58.22 | 2.00E-79 | 287 | glycerophosphoryl diester phosphodiesterase |
| TON_0201 | TK1397 | 83.87 | 1.00E-124 | 438 | glycerophosphoryl diester phosphodiesterase |
| TON_0202 | TK1396 | 87.02 | 0 | 843 | glpK glycerol kinase |
| TON_0203 | TK1393 | 90.12 | 0 | 839 | anaerobic glycerol 3-phosphate dehydrogenase |
| TON_0204 | TK1392 | 82.73 | 0 | 637 | NADH oxidase |
| TON_0205 | TK1391 | 77.68 | 2.00E-48 | 183 | molybdopterin oxidoreductase |
| TON_0207 | TK1388 | 70.31 | 4.00E-22 | 95.1 | hypothetical protein |
| TON_0208 | TK1387 | 70.26 | 2.00E-95 | 340 | membrane protein |
| TON_0209 | TK1386 | 73.45 | 4.00E-95 | 339 | metallophosphoesterase |
| TON_0210 | TK1385 | 76.09 | 5.00E-39 | 151 | hypothetical protein |
| TON_0211 | TK1383 | 81.66 | 1.00E-111 | 394 | HAD-superfamily hydrolase |
| TON_0212 | TK1381 | 83.29 | 0 | 698 | hypothetical protein |
| TON_0213 | TK1380 | 88.54 | 0 | 791 | glycine dehydrogenase subunit 1 |
| TON_0214 | TK1379 | 92.42 | 0 | 933 | glycine dehydrogenase subunit 2 |
| TON_0216 | TK1084 | 92.68 | 7.00E-27 | 110 | rpoH DNA-directed RNA polymerase subunit H |
| TON_0217 | TK1083 | 91.35 | 0 | 2099 | DNA-directed RNA polymerase subunit B |
| TON_0218 | TK1082 | 94.03 | 0 | 1679 | DNA-directed RNA polymerase subunit A' |
| TON_0219 | TK1081 | 88.49 | 0 | 684 | DNA-directed RNA polymerase subunit A'' |
| TON_0220 | TK1080 | 87.13 | 7.00E-40 | 154 | 50S ribosomal protein L30e |
| TON_0221 | TK1079 | 95.17 | 1.00E-77 | 280 | transcription elongation factor NusA-like protein |
| TON_0222 | TK1078 | 97.96 | 6.00E-73 | 265 | rps12P 30S ribosomal protein S12 |
| TON_0223 | TK1077 | 90.23 | 5.00E-107 | 379 | 30S ribosomal protein S7 |
| TON_0224 | TK1071 | 84.32 | 0 | 672 | phosphodiesterase |
| TON_0225 | TK1069 | 66.67 | 2.00E-39 | 152 | hypothetical protein |
| TON_0227 | TK1067 | 84.43 | 0 | 711 | hypothetical protein |
| TON_0228 | TK1093 | 82.09 | 4.00E-27 | 111 | sulfur carrier protein ThiS |
| TON_0229 | TK1739 | 27.31 | 2.00E-39 | 155 | hypothetical protein |
| TON_0231 | TK1094 | 88.19 | 0 | 738 | alanine aminotransferase |
| TON_0233 | TK1096 | 68.91 | 2.00E-50 | 191 | membrane protein |

| | | | | | |
|---|---|---|---|---|---|
| TON_0234 | TK0825 | 81.4 | 2.00E-13 | 65.9 | hypothetical protein |
| TON_0235 | TK1098 | 94.68 | 1.00E-47 | 179 | rpl44e 50S ribosomal protein L44e |
| TON_0236 | TK1099 | 93.85 | 1.00E-31 | 126 | rps27e 30S ribosomal protein S27e |
| TON_0237 | TK1100 | 88.89 | 2.00E-136 | 477 | translation initiation factor IF-2 |
| TON_0238 | TK1101 | 84.48 | 3.00E-25 | 105 | H/ACA RNA-protein complex component Nop10p |
| TON_0239 | TK1102 | 90.64 | 1.00E-129 | 454 | hypothetical protein |
| TON_0240 | TK1103 | 76.47 | 2.00E-81 | 293 | cobalt ABC transporter permease CbiM |
| TON_0241 | TK1104 | 75.31 | 6.00E-25 | 104 | membrane protein |
| TON_0242 | TK1105 | 73.17 | 1.00E-67 | 248 | cobalt ABC transporter permease CbiQ |
| TON_0243 | TK1106 | 75.19 | 6.00E-114 | 402 | cobalt ABC transporter ATPase |
| TON_0244 | TK1108 | 88.57 | 0 | 803 | phosphohexomutase |
| TON_0245 | TK1109 | 76.56 | 0 | 694 | bifunctional mannose-6-phosphate isomerase/mannose-1-phosphate guanylyl transferase |
| TON_0246 | TK1110 | 78.59 | 0 | 688 | ADP-dependent glucokinase |
| TON_0247 | TK1111 | 90.96 | 6.00E-99 | 352 | glucose-6-phosphate isomerase |
| TON_0249 | TK1112 | 85.96 | 3.00E-14 | 68.9 | RNA-binding protein |
| TON_0251 | TK1113 | 71.82 | 6.00E-81 | 292 | signaling protein |
| TON_0252 | TK1114 | 81.73 | 1.00E-149 | 521 | ribonuclease Z |
| TON_0253 | TK1115 | 65.36 | 1.00E-87 | 315 | hypothetical protein |
| TON_0254 | TK2020 | 69.76 | 9.00E-99 | 352 | ABC-type iron(III)-siderophore transport system, ATPase component |
| TON_0255 | TK2019 | 80.52 | 2.00E-135 | 474 | ABC-type iron(III)-siderophore transport system permease |
| TON_0256 | TK2018 | 65.66 | 3.00E-140 | 490 | hemV-3 iron (III) ABC transporter ATP-binding protein |
| TON_0258 | TK2016 | 61.06 | 4.00E-39 | 152 | iron-molybdenum cofactor-binding protein |
| TON_0259 | TK2015 | 61.56 | 5.00E-104 | 370 | mae1-like C4-dicarboxylate transporter |
| TON_0260 | TK2009 | 77.13 | 3.00E-110 | 390 | cobalt/zinc/cadmium cation efflux pump protein |
| TON_0261 | TK2008 | 80.29 | 2.00E-54 | 203 | hypA hydrogenase nickel incorporation protein |
| TON_0262 | TK2007 | 80.65 | 2.00E-105 | 374 | chromosome partitioning ATPase |
| TON_0263 | TK2004 | 65.36 | 1.00E-56 | 211 | hydrogenase maturation protease HycI |
| TON_0264 | TK2002 | 69.84 | 4.00E-71 | 259 | mobA molybdopterin-guanine dinucleotide biosynthesis protein MobA |
| TON_0283 | TK2001 | 74.32 | 5.00E-28 | 114 | hydrogenase expression/formation protein HypC |
| TON_0286 | TK1997 | 84.59 | 0 | 1318 | hydrogenase maturation protein HypF |
| TON_0287 | TK1993 | 88.66 | 1.00E-164 | 571 | hydrogenase expression/formation protein HypE |
| TON_0288 | TK1991 | 74.23 | 1.00E-40 | 156 | hypothetical protein |
| TON_0289 | TK1990 | 89.2 | 0 | 711 | cysteine desulfurase |
| TON_0290 | TK1301 | 75.93 | 4.00E-157 | 546 | ATP-NAD kinase |
| TON_0291 | TK1300 | 63.9 | 0 | 644 | membrane protein |
| TON_0296 | TK0706 | 62.73 | 7.00E-177 | 612 | iron(III)-siderophore ABC transporter periplasmic protein |
| TON_0297 | TK0707 | 66.88 | 8.00E-97 | 345 | ABC-type iron(III)-siderophore transport system, pernease component |
| TON_0298 | TK0708 | 62.95 | 1.00E-82 | 298 | ABC-type iron(III)-siderophore transport system, ATPase component |
| TON_0302 | TK0709 | 40.54 | 6.00E-51 | 193 | hypothetical protein |
| TON_0304 | TK1007 | 46.39 | 7.00E-75 | 273 | permease |
| TON_0305 | TK1299 | 88.86 | 0 | 741 | NADH oxidase |
| TON_0306 | TK1297 | 81.96 | 0 | 629 | nonsense-mediated mRNA decay protein |
| TON_0307 | TK1296 | 86.87 | 8.00E-40 | 153 | hypothetical protein |

| | | | | | |
|---|---|---|---|---|---|
| TON_0309 | TK1294 | 53.07 | 3.00E-63 | 234 | membrane protein |
| TON_0310 | TK1293 | 67.12 | 0 | 1114 | membrane-bound dolichyl-phosphate-mannose-protein mannosyltransferase |
| TON_0311 | TK1292 | 85.7 | 0 | 1289 | phosphoenolpyruvate synthase |
| TON_0312 | TK1289 | 70.51 | 0 | 659 | sodium-driven multidrug efflux pump protein |
| TON_0313 | TK1288 | 68.23 | 0 | 667 | sodium-driven multidrug efflux pump protein |
| TON_0314 | TK1287 | 89.14 | 9.00E-113 | 398 | upp uracil phosphoribosyltransferase |
| TON_0316 | TK1088 | 92.86 | 1.00E-180 | 624 | geranylgeranyl hydrogenase |
| TON_0317 | TK1087 | 88.06 | 3.00E-30 | 122 | ferredoxin 2 |
| TON_0318 | TK1086 | 73.31 | 5.00E-103 | 365 | putative transcriptional regulator |
| TON_0319 | TK1085 | 88.5 | 2.00E-118 | 417 | protein disulfide oxidoreductase |
| TON_0320 | TK0445 | 84.71 | 1.00E-39 | 153 | hypothetical protein |
| TON_0321 | TK0446 | 50.49 | 5.00E-52 | 196 | hypothetical protein |
| TON_0326 | TK0464 | 85.06 | 8.00E-128 | 448 | hypothetical protein |
| TON_0327 | TK0465 | 93.95 | 6.00E-115 | 405 | acetyl-CoA synthetase I subunit beta |
| TON_0328 | TK0466 | 61.02 | 1.00E-138 | 485 | hypothetical protein |
| TON_0330 | TK0469 | 54.66 | 2.00E-46 | 177 | hypothetical protein |
| TON_0332 | TK0471 | 88.59 | 1.00E-127 | 447 | putative transcriptional regulator |
| TON_0334 | TK0473 | 73.87 | 4.00E-44 | 168 | hypothetical protein |
| TON_0335 | TK0474 | 79.08 | 8.00E-109 | 385 | arginase |
| TON_0336 | TK0475 | 92.61 | 0 | 702 | bifunctional D-arabino 3-hexulose-6-phosphate formaldehyde lyase/phosphohexuloisomerase |
| TON_0337 | TK0476 | 64.54 | 3.00E-93 | 333 | hypothetical protein |
| TON_0338 | TK0477 | 74.88 | 6.00E-89 | 318 | hydrolase |
| TON_0339 | TK0478 | 71.19 | 3.00E-20 | 89 | hypothetical protein |
| TON_0340 | TK0479 | 62.31 | 5.00E-99 | 352 | hypothetical protein |
| TON_0341 | TK0480 | 85.87 | 7.00E-80 | 288 | metal-dependent phosphohydrolase |
| TON_0342 | TK0481 | 78.24 | 3.00E-74 | 269 | transcription elongation factor NusA-like protein |
| TON_0343 | TK0492 | 89.95 | 0 | 801 | aspC aspartyl-tRNA synthetase |
| TON_0344 | TK0493 | 42.13 | 1.00E-143 | 502 | membrane protein |
| TON_0345 | TK2300 | 84.75 | 3.00E-55 | 206 | peptidyl-tRNA hydrolase |
| TON_0346 | TK2302 | 83.17 | 0 | 702 | truD tRNA pseudouridine synthase D |
| TON_0347 | TK2301 | 83.19 | 8.00E-114 | 401 | phosphoglycolate phosphatase |
| TON_0348 | TK0494 | 80.63 | 1.00E-171 | 594 | bifunctional carboxypeptidase/aminoacylase |
| TON_0349 | TK1195 | 90.53 | 2.00E-45 | 172 | hypothetical protein |
| TON_0350 | TK1194 | 94.44 | 3.00E-56 | 209 | hypothetical protein |
| TON_0352 | TK1193 | 90.79 | 0 | 961 | pyrG CTP synthetase |
| TON_0353 | TK1192 | 45.95 | 3.00E-43 | 167 | membrane protein |
| TON_0354 | TK1191 | 90.77 | 2.00E-55 | 206 | 30S ribosomal protein S8e |
| TON_0356 | TK1190 | 89.39 | 0 | 656 | GTPase |
| TON_0357 | TK0735 | 66.05 | 1.00E-98 | 351 | hydrolase |
| TON_0359 | TK1188 | 84.03 | 0 | 692 | sugar-phosphate nucleotydyltransferase |
| TON_0360 | TK1187 | 48.62 | 4.00E-102 | 363 | membrane protein |
| TON_0361 | TK1186 | 84.87 | 0 | 675 | hypothetical protein |
| TON_0362 | TK1183 | 85.76 | 1.00E-152 | 531 | methionine aminopeptidase |
| TON_0363 | TK1182 | 76.23 | 0 | 705 | Trk-type potassium transport system, membrane component |
| TON_0364 | TK1181 | 63.22 | 6.00E-91 | 326 | cobalt transport ATP-binding protein |
| TON_0365 | TK1180 | 47.58 | 1.00E-60 | 225 | hypothetical protein |
| TON_0366 | TK1179 | 76.5 | 3.00E-72 | 263 | adenylate cyclase, class 2 |

| | | | | | |
|---|---|---|---|---|---|
| TON_0367 | TK1178 | 57.69 | 2.00E-59 | 220 | archaemetzincin-like protein |
| TON_0368 | TK1475 | 35.97 | 4.00E-17 | 79.3 | hypothetical protein |
| TON_0369 | TK1177 | 91.33 | 0 | 653 | deblocking aminopeptidase |
| TON_0371 | TK1175 | 65.89 | 6.00E-48 | 181 | Holliday junction resolvase |
| TON_0372 | TK1174 | 87.93 | 5.00E-85 | 305 | acetyltransferase |
| TON_0373 | TK1173 | 87.88 | 4.00E-133 | 466 | undecaprenyl diphosphate synthase |
| TON_0374 | TK1172 | 72.02 | 2.00E-94 | 337 | TBP-interacting protein |
| TON_0375 | TK1171 | 47.03 | 1.00E-49 | 189 | hypothetical protein |
| TON_0376 | TK1170 | 90.49 | 0 | 1091 | threonyl-tRNA synthetase |
| TON_0377 | TK1169 | 80.1 | 6.00E-93 | 332 | exosome complex RNA-binding protein Csl4 |
| TON_0378 | TK1168 | 78.85 | 1.00E-84 | 304 | hypothetical protein |
| TON_0379 | TK1167 | 84.95 | 3.00E-44 | 168 | DNA-directed RNA polymerase subunit L |
| TON_0381 | TK1166 | 75 | 7.00E-56 | 207 | hypothetical protein |
| TON_0382 | TK1165 | 85.07 | 2.00E-137 | 480 | AP endonuclease |
| TON_0383 | TK1164 | 55.36 | 2.00E-88 | 317 | endopeptidase IV |
| TON_0384 | TK1163 | 81.65 | 6.00E-65 | 238 | hypothetical protein |
| TON_0385 | TK1162 | 60.79 | 5.00E-99 | 353 | membrane protein |
| TON_0386 | TK1161 | 81.99 | 3.00E-134 | 469 | dihydropteroate synthase |
| TON_0388 | TK0939 | 68.52 | 2.00E-125 | 440 | GHMP kinase |
| TON_0389 | TK0940 | 71.86 | 2.00E-111 | 394 | N-glycosylase/DNA lyase |
| TON_0390 | TK0941 | 55.21 | 6.00E-47 | 179 | membrane protein |
| TON_0391 | TK2053 | 80.33 | 8.00E-145 | 505 | multidrug ABC transporter ATPase |
| TON_0392 | TK0929 | 80.76 | 0 | 719 | 3-octaprenyl-4-hydroxybenzoate carboxy-lyase |
| TON_0393 | TK0928 | 86.62 | 0 | 822 | DNA/RNA repair helicase |
| TON_0394 | TK0927 | 75.09 | 5.00E-120 | 422 | truA tRNA pseudouridine synthase A |
| TON_0395 | TK0925 | 86.41 | 0 | 1021 | pheT phenylalanyl-tRNA synthetase subunit beta |
| TON_0396 | TK0921 | 86.23 | 0 | 877 | pheS phenylalanyl-tRNA synthetase subunit alpha |
| TON_0397 | TK0916 | 94.84 | 0 | 686 | tdh L-threonine 3-dehydrogenase |
| TON_0398 | TK0915 | 81.38 | 5.00E-115 | 405 | ATPase |
| TON_0399 | TK0914 | 94.61 | 0 | 753 | 3-hydroxy-3-methylglutaryl-CoA reductase |
| TON_0402 | TK0911 | 89.82 | 0 | 979 | glutamyl-tRNA(Gln) amidotransferase subunit E |
| TON_0403 | TK0908 | 75.57 | 0 | 684 | glutamyl-tRNA(Gln) amidotransferase subunit D |
| TON_0404 | TK0904 | 78.35 | 9.00E-41 | 157 | hypothetical protein |
| TON_0405 | TK0903 | 82.12 | 0 | 626 | putative pseudouridylate synthase |
| TON_0406 | TK0902 | 94.9 | 8.00E-50 | 187 | 50S ribosomal protein L21e |
| TON_0407 | TK0901 | 83.33 | 8.00E-51 | 191 | DNA-directed RNA polymerase subunit F |
| TON_0408 | TK0900 | 84.47 | 6.00E-102 | 362 | hypothetical protein |
| TON_0409 | TK0899 | 74.63 | 3.00E-118 | 416 | ksgA dimethyladenosine transferase |
| TON_0410 | TK2095 | 34.16 | 5.00E-21 | 93.2 | rubrerythrin-like protein |
| TON_0411 | TK0897 | 84.68 | 0 | 1004 | hypothetical protein |
| TON_0412 | TK0896 | 47.08 | 2.00E-63 | 234 | membrane protein |
| TON_0414 | TK0894 | 54.35 | 4.00E-77 | 280 | hypothetical protein |
| TON_0415 | TK0893 | 81.09 | 2.00E-175 | 607 | pyruvate fromate-lyase activating enzyme-related protein |
| TON_0416 | TK0892 | 52.52 | 1.00E-72 | 265 | polysaccharide deacetylase |
| TON_0417 | TK0891 | 31.87 | 4.00E-40 | 158 | glycosyltransferase |
| TON_0419 | TK0890 | 71.34 | 8.00E-126 | 442 | thiamine monophosphate kinase |
| TON_0421 | TK0887 | 83.73 | 0 | 708 | phosphohydrolase |
| TON_0422 | TK0885 | 81.41 | 2.00E-66 | 243 | hypothetical protein |

| | | | | | |
|---|---|---|---|---|---|
| TON_0423 | TK0886 | 59.34 | 2.00E-162 | 565 | Voltage-gated chloride channel protein |
| TON_0425 | TK0882 | 82.46 | 1.00E-129 | 454 | Agmatinase |
| TON_0426 | TK0878 | 87.5 | 5.00E-66 | 241 | translation initiation factor IF-5A |
| TON_0427 | TK0877 | 45.75 | 3.00E-106 | 378 | membrane protein |
| TON_0428 | TK0876 | 74.42 | 2.00E-96 | 343 | ribosome biogenesis protein |
| TON_0429 | TK0875 | 75.48 | 4.00E-153 | 533 | Saccharopine reductase |
| TON_0430 | TK0874 | 58.33 | 5.00E-56 | 209 | metallophosphoesterase |
| TON_0431 | TK0873 | 63.12 | 2.00E-121 | 427 | hypothetical protein |
| TON_0432 | TK1608 | 29.91 | 1.00E-19 | 88.6 | hypothetical protein |
| TON_0434 | TK0872 | 87.06 | 2.00E-165 | 573 | tRNA/rRNA cytosine-C5-methylase |
| TON_0435 | TK0871 | 95.24 | 1.00E-173 | 601 | ornithine carbamoyltransferase |
| TON_0437 | TK0870 | 81.63 | 2.00E-121 | 427 | thyX FAD-dependent thymidylate synthase |
| TON_0440 | TK0869 | 54.11 | 5.00E-68 | 249 | hypothetical protein |
| TON_0441 | TK0868 | 88.34 | 7.00E-88 | 315 | bis(5'-adenosyl)-triphosphatase |
| TON_0442 | TK0866 | 92.42 | 0 | 693 | cofactor-independent phosphoglycerate mutase |
| TON_0443 | TK0845 | 88.13 | 6.00E-132 | 462 | oxidoreductase |
| TON_0444 | TK0844 | 87.52 | 0 | 1031 | tungsten-containing oxidoreductase |
| TON_0446 | TK0649 | 92.95 | 7.00E-123 | 431 | putative tRNA-binding protein |
| TON_0447 | TK0648 | 63.24 | 1.00E-117 | 415 | hypothetical protein |
| TON_0448 | TK0646 | 72.64 | 2.00E-43 | 166 | hypothetical protein |
| TON_0449 | TK0645 | 83.74 | 2.00E-152 | 531 | phosphohydrolase |
| TON_0450 | TK0644 | 73.58 | 3.00E-41 | 159 | hypothetical protein |
| TON_0452 | TK0642 | 73.08 | 1.00E-29 | 120 | hypothetical protein |
| TON_0453 | TK0617 | 87.44 | 2.00E-98 | 350 | ribosomal biogenesis protein |
| TON_0454 | TK0616 | 89.8 | 1.00E-20 | 90.1 | DNA-directed RNA polymerase subunit P |
| TON_0455 | TK0615 | 96.51 | 8.00E-44 | 167 | rpl37ae 50S ribosomal protein L37Ae |
| TON_0456 | TK1196 | 63.25 | 2.00E-62 | 230 | hypothetical protein |
| TON_0457 | TK1197 | 66.28 | 1.00E-27 | 113 | hypothetical protein |
| TON_0458 | TK1198 | 86.17 | 4.00E-133 | 466 | organic radical activating enzyme |
| TON_0462 | TK1199 | 92.27 | 0 | 824 | TBP-interacting protein |
| TON_0463 | TK1202 | 65.03 | 2.00E-119 | 420 | hypothetical protein |
| TON_0464 | TK1203 | 58.55 | 5.00E-51 | 192 | hypothetical protein |
| TON_0466 | TK1204 | 77.78 | 2.00E-71 | 260 | hypothetical protein |
| TON_0469 | TK1207 | 56.49 | 4.00E-47 | 179 | hypothetical protein |
| TON_0471 | TK1209 | 71.07 | 1.00E-71 | 261 | transmembrane electron transport protein |
| TON_0482 | TK1210 | 83.01 | 3.00E-71 | 259 | transcriptional regulator |
| TON_0483 | TK1211 | 80.81 | 0 | 727 | 4-aminobutyrate aminotransferase |
| TON_0484 | TK1212 | 81.52 | 0 | 657 | xanthine/uracilpermease |
| TON_0486 | TK1214 | 76.84 | 3.00E-87 | 313 | NADH dehydrogenase subunit I |
| TON_0487 | TK1215 | 77.98 | 0 | 634 | NADH dehydrogenase subunit D |
| TON_0488 | TK1216 | 56.14 | 6.00E-50 | 189 | NADH dehydrogenase subunit C |
| TON_0489 | TK1217 | 81.82 | 1.00E-81 | 294 | NADH dehydrogenase subunit B |
| TON_0490 | TK1218 | 58.64 | 9.00E-97 | 345 | NADH dehydrogenase subunit |
| TON_0491 | TK1219 | 60.23 | 0 | 716 | NADH dehydrogenase subunit M |
| TON_0492 | TK1220 | 74.75 | 0 | 677 | NADH dehydrogenase subunit N |
| TON_0493 | TK1221 | 90.35 | 6.00E-46 | 174 | putative monovalent cation/H+ antiporter subunit C |
| TON_0494 | TK1222 | 84.68 | 3.00E-87 | 313 | putative monovalent cation/H+ antiporter subunit B |
| TON_0495 | TK1223 | 79.79 | 6.00E-27 | 111 | hypothetical protein |

| | | | | | |
|---|---|---|---|---|---|
| TON_0496 | TK1224 | 81.67 | 1.00E-48 | 184 | putative monovalent cation/H+ antiporter subunit G |
| TON_0497 | TK1225 | 89.29 | 4.00E-36 | 141 | putative monovalent cation/H+ antiporter subunit F |
| TON_0498 | TK1226 | 77.4 | 7.00E-75 | 271 | putative monovalent cation/H+ antiporter subunit E |
| TON_0499 | TK1227 | 95.18 | 1.00E-88 | 317 | regulator of amino acid metabolism |
| TON_0500 | TK1228 | 87.11 | 0 | 753 | molybdenum cofactor biosynthesis protein A |
| TON_0501 | TK1230 | 82.76 | 1.00E-180 | 624 | UDP-N-acetylglucosamine 2-epimerase |
| TON_0502 | TK1231 | 84.45 | 0 | 749 | UDP-N-acetyl-D-mannosaminuronate dehydrogenase |
| TON_0503 | TK1232 | 81.72 | 0 | 638 | hypothetical protein |
| TON_1971 | TK1233 | 45.4 | 2.00E-26 | 111 | membrane protein |
| TON_0504 | TK1234 | 80.22 | 1.00E-31 | 126 | lipoate-protein ligase A |
| TON_0505 | TK1235 | 83.96 | 0 | 1102 | argS arginyl-tRNA synthetase |
| TON_0506 | TK1237 | 80.82 | 1.00E-144 | 504 | dihydrodipicolinate synthase |
| TON_0507 | TK1239 | 94.22 | 0 | 760 | peptide chain release factor 1 |
| TON_0509 | TK1241 | 48.73 | 2.00E-37 | 147 | hypothetical protein |
| TON_0511 | TK1246 | 65.57 | 2.00E-62 | 230 | hypothetical protein |
| TON_0512 | TK1247 | 63.3 | 1.00E-165 | 574 | membrane-associated metallopeptidase |
| TON_0513 | TK1248 | 86.92 | 7.00E-51 | 191 | hypothetical protein |
| TON_0514 | TK1249 | 90.16 | 0 | 707 | hypothetical protein |
| TON_0517 | TK1251 | 93.38 | 6.00E-71 | 258 | rps15p 30S ribosomal protein S15 |
| TON_0518 | TK1252 | 88.11 | 0 | 800 | ssDNA-specific exonuclease |
| TON_0519 | TK1253 | 70.37 | 3.00E-30 | 122 | hypothetical protein |
| TON_0520 | TK1254 | 89.5 | 2.00E-106 | 377 | 30S ribosomal protein S3Ae |
| TON_0521 | TK1257 | 85.06 | 5.00E-168 | 582 | RNA-binding protein |
| TON_0523 | TK1259 | 70.47 | 6.00E-65 | 238 | putative transcriptional regulator |
| TON_0524 | TK1260 | 60.44 | 3.00E-30 | 122 | nucleotide pyrophosphohydrolase |
| TON_0525 | TK1261 | 53.6 | 1.00E-38 | 150 | transcriptional regulator |
| TON_0526 | TK1262 | 73.98 | 3.00E-50 | 189 | metalloprotease |
| TON_0528 | TK1263 | 64.29 | 1.00E-93 | 335 | hypothetical protein |
| TON_0529 | TK1264 | 90.98 | 0 | 1113 | ATP-dependent protease Lon |
| TON_0530 | TK0731 | 75.2 | 1.00E-96 | 345 | Fe-S cluster assembly ABC transporter ATPase |
| TON_0531 | TK0730 | 86.85 | 0 | 785 | Fe-S cluster assembly ABC transporter permease |
| TON_0533 | TK2066 | 77.7 | 6.00E-63 | 231 | hydrogenase-specific maturation endopeptidase |
| TON_0534 | TK2069 | 86.25 | 0 | 720 | cytosolic NiFe-hydrogenase subunit alpha |
| TON_0535 | TK2070 | 85.66 | 1.00E-128 | 451 | cytosolic NiFe-hydrogenase subunit delta |
| TON_0536 | TK2071 | 91.29 | 1.00E-158 | 551 | cytochrome-c3 hydrogenase subunit gamma |
| TON_0537 | TK2072 | 78.75 | 0 | 625 | sulfhydrogenase subunit beta |
| TON_0541 | TK2073 | 67.2 | 7.00E-48 | 181 | 4Fe-4S binding protein |
| TON_0542 | TK2074 | 84.09 | 4.00E-179 | 619 | putative glutamate synthase subunit beta |
| TON_0543 | TK2075 | 85.12 | 5.00E-69 | 252 | 4Fe-4S binding protein |
| TON_0544 | TK1569 | 38.27 | 1.00E-57 | 216 | alcohol dehydrogenase |
| TON_0546 | TK0865 | 59.51 | 3.00E-100 | 358 | putative vitamin B12 transport protein |
| TON_0547 | TK0569 | 37.83 | 4.00E-50 | 191 | hypothetical protein |
| TON_0548 | TK1266 | 86.1 | 7.00E-93 | 332 | putative transcriptional regulator |
| TON_0549 | TK1267 | 63.1 | 1.00E-90 | 325 | permease |
| TON_0553 | TK1268 | 55.09 | 7.00E-143 | 499 | hypothetical protein |
| TON_0554 | TK1269 | 73.31 | 0 | 651 | phosphoesterase |
| TON_0555 | TK1270 | 85.51 | 9.00E-29 | 117 | hypothetical protein |
| TON_0557 | TK1272 | 65.62 | 2.00E-66 | 244 | putative transcriptional regulator |

| | | | | | |
|---|---|---|---|---|---|
| TON_0558 | TK0906 | 68.16 | 4.00E-66 | 243 | endonuclease V |
| TON_0559 | TK0907 | 70.24 | 9.00E-72 | 261 | haloacid dehalogenase superfamily protein |
| TON_0560 | TK0545 | 89.38 | 0 | 740 | S-adenosylmethionine synthetase |
| TON_0562 | TK0338 | 70.06 | 5.00E-69 | 252 | putative kinase |
| TON_0564 | TK0547 | 69.72 | 3.00E-84 | 303 | metallophosphoesterase |
| TON_0565 | TK0548 | 87.15 | 0 | 715 | aspartate aminotransferase |
| TON_0566 | TK0549 | 81.03 | 0 | 888 | carbamoyl transferase |
| TON_0568 | TK0550 | 90.25 | 0 | 824 | prolyl-tRNA synthetase |
| TON_0569 | TK0551 | 80.78 | 3.00E-159 | 553 | D-isomer specific 2-hydroxyacid dehydrogenase |
| TON_0571 | TK0979 | 81.1 | 2.00E-98 | 350 | membrane protein |
| TON_0572 | TK0980 | 42.44 | 6.00E-26 | 109 | membrane protein |
| TON_0573 | TK1053 | 57.94 | 1.00E-49 | 188 | membrane protein |
| TON_0575 | TK1459 | 46.97 | 7.00E-84 | 303 | hypothetical protein |
| TON_0579 | TK1049 | 86.91 | 0 | 1315 | metG methionyl-tRNA synthetase |
| TON_0580 | TK1119 | 75.65 | 6.00E-48 | 181 | 6-pyruvoyl-tetrahydropterin synthase |
| TON_0581 | TK1120 | 77.12 | 5.00E-69 | 252 | hypothetical protein |
| TON_0582 | TK1123 | 87.63 | 4.00E-84 | 302 | 2-oxoglutarate ferredoxin oxidoreductase subunit gamma |
| TON_0583 | TK1124 | 93.59 | 2.00E-159 | 553 | 2-oxoglutarate ferredoxin oxidoreductase subunit beta |
| TON_0584 | TK1125 | 90.58 | 0 | 654 | 2-oxoglutarate ferredoxin oxidoreductase subunit alpha |
| TON_0585 | TK1126 | 92.35 | 2.00E-88 | 316 | 2-oxoglutarate ferredoxin oxidoreductase subunit gamma |
| TON_0586 | TK1129 | 94.37 | 3.00E-163 | 566 | 2-oxoglutarate ferredoxin oxidoreductase subunit beta |
| TON_0587 | TK1130 | 91.16 | 0 | 764 | 2-oxoglutarate ferredoxin oxidoreductase subunit alpha |
| TON_0588 | TK1131 | 86.67 | 9.00E-35 | 137 | 2-oxoacid:ferredoxin oxidoreductase subunit delta |
| TON_0589 | TK1135 | 88.27 | 4.00E-174 | 602 | hypothetical protein |
| TON_0590 | TK1137 | 81.5 | 3.00E-123 | 433 | surE stationary phase survival protein SurE |
| TON_0592 | TK0653 | 87.41 | 2.00E-134 | 470 | signal recognition particle GTPase |
| TON_0593 | TK0656 | 59.18 | 6.00E-47 | 178 | hypothetical protein |
| TON_0595 | TK0658 | 75.45 | 0 | 793 | ABC transporter ATPase |
| TON_0596 | TK0659 | 66.03 | 2.00E-104 | 371 | ABC transporter permease |
| TON_0597 | TK0660 | 65.44 | 3.00E-99 | 353 | ABC transporter permease |
| TON_0598 | TK0661 | 58.62 | 2.00E-101 | 360 | ABC-type manganese/zinc transport system periplasmic protein |
| TON_0599 | TK0662 | 45.48 | 7.00E-140 | 489 | hypothetical protein |
| TON_0600 | TK0663 | 86.11 | 8.00E-33 | 130 | hypothetical protein |
| TON_0601 | TK0664 | 85.12 | 5.00E-104 | 369 | hypoxanthine/guanine phosphoribosyltransferase |
| TON_0602 | TK0444 | 88.03 | 0 | 901 | cysS cysteinyl-tRNA synthetase |
| TON_0604 | TK0439 | 67.28 | 5.00E-62 | 229 | membrane-associated metalloprotease |
| TON_0606 | TK0436 | 87.38 | 0 | 1097 | ABC-type transporter, ATPase component |
| TON_0607 | TK0429 | 83.55 | 6.00E-72 | 261 | ribH 6,7-dimethyl-8-ribityllumazine synthase |
| TON_0608 | TK0428 | 82.34 | 0 | 647 | bifunctional 3,4-dihydroxy-2-butanone 4-phosphate synthase/GTP cyclohydrolase II protein |
| TON_0609 | TK0425 | 74.18 | 1.00E-79 | 287 | riboflavin synthase subunit alpha |
| TON_0610 | TK0424 | 77.9 | 6.00E-160 | 555 | riboflavin biosynthesis protein RibD |
| TON_0611 | TK0423 | 83.41 | 0 | 1073 | prolyl endopeptidase |
| TON_0612 | TK1048 | 69.87 | 7.00E-99 | 352 | ATPase |
| TON_0613 | TK0764 | 77.66 | 0 | 2099 | large helicase-like protein |

| | | | | | |
|---|---|---|---|---|---|
| TON_0614 | TK0763 | 76.67 | 4.00E-29 | 118 | hypothetical protein |
| TON_0615 | TK0762 | 69.86 | 6.00E-142 | 496 | glycosyltransferase |
| TON_0616 | TK0761 | 66.9 | 4.00E-86 | 310 | sodium/phosphate symporter |
| TON_0617 | TK0760 | 91.72 | 0 | 1074 | 7-cyano-7-deazaguanine tRNA-ribosyltransferase |
| TON_0619 | TK0379 | 85.3 | 0 | 630 | hypothetical protein |
| TON_0620 | TK0378 | 96.28 | 1.00E-119 | 421 | chromosome partitioning protein |
| TON_0621 | TK0026 | 64.95 | 3.00E-78 | 283 | phosphoglycolate phosphatase |
| TON_0622 | TK0025 | 73.54 | 6.00E-142 | 495 | membrane protein |
| TON_0623 | TK0024 | 72.05 | 2.00E-113 | 400 | hypothetical protein |
| TON_0624 | TK0023 | 79.27 | 3.00E-62 | 229 | hypothetical protein |
| TON_0625 | TK0022 | 86.53 | 2.00E-163 | 567 | hypothetical protein |
| TON_0627 | TK0775 | 89.02 | 8.00E-176 | 608 | deblocking aminopeptidase |
| TON_0628 | TK0774 | 79.45 | 2.00E-124 | 437 | permease |
| TON_0629 | TK1041 | 83.82 | 3.00E-51 | 192 | transcriptional regulator |
| TON_0630 | TK1042 | 65.82 | 7.00E-162 | 562 | membrane protein |
| TON_0631 | TK1043 | 32.2 | 2.00E-14 | 72 | hypothetical protein |
| TON_0634 | TK1044 | 66.75 | 3.00E-162 | 563 | permease |
| TON_0635 | TK1045 | 82.89 | 4.00E-56 | 209 | cobalamin adenosyltransferase |
| TON_0637 | TK1047 | 76 | 9.00E-112 | 395 | translation initiation factor IF-2 |
| TON_0638 | TK1472 | 83.67 | 5.00E-108 | 382 | competence damage-inducible protein A |
| TON_0639 | TK0765 | 90.72 | 0 | 636 | glyceraldehyde-3-phosphate dehydrogenase |
| TON_0641 | TK0768 | 77.66 | 7.00E-38 | 147 | hypothetical protein |
| TON_0642 | TK0769 | 79.5 | 3.00E-72 | 263 | hypothetical protein |
| TON_0643 | TK0978 | 91.47 | 0 | 1070 | glycyl-tRNA synthetase |
| TON_0644 | TK0976 | 86.67 | 2.00E-33 | 132 | small nuclear ribonucleoprotein |
| TON_0645 | TK0975 | 83.61 | 2.00E-27 | 112 | rpl37e 50S ribosomal protein L37e |
| TON_0646 | TK0974 | 75.38 | 0 | 667 | sodium-driven multidrug efflux pump protein |
| TON_0647 | TK0971 | 67.58 | 6.00E-146 | 509 | hypothetical protein |
| TON_0648 | TK0970 | 69.68 | 3.00E-155 | 540 | N(2),N(2)-dimethylguanosine tRNA methyltransferase |
| TON_0649 | TK0969 | 75 | 1.00E-33 | 132 | 50S ribosomal protein L35 |
| TON_0650 | TK0968 | 87.96 | 1.00E-110 | 391 | hydrolase |
| TON_0651 | TK0967 | 80.46 | 1.00E-161 | 561 | Xaa-Pro aminopeptidase |
| TON_0652 | TK0964 | 91.6 | 0 | 630 | cell division protein |
| TON_0653 | TK0961 | 79.7 | 3.00E-159 | 553 | HypE protein |
| TON_0654 | TK0960 | 81.08 | 4.00E-118 | 416 | ATPase |
| TON_0655 | TK0959 | 51.18 | 2.00E-28 | 117 | hypothetical protein |
| TON_0656 | TK0958 | 60 | 2.00E-21 | 92.4 | iron(II) transport protein A |
| TON_0657 | TK0957 | 75.49 | 0 | 959 | iron(II) transport protein B |
| TON_0659 | TK0955 | 85.47 | 0 | 734 | sugar-phosphate nucleotidyltransferase |
| TON_0660 | TK0510 | 73.46 | 5.00E-109 | 386 | hypothetical protein |
| TON_0661 | TK0509 | 75 | 1.00E-72 | 264 | aromatic acid decarboxylase |
| TON_0662 | TK0508 | 69.54 | 8.00E-53 | 198 | putative transcriptional regulator |
| TON_0663 | TK0507 | 80.72 | 1.00E-70 | 257 | adenylate cyclase |
| TON_0666 | TK0506 | 88.16 | 0 | 728 | translation-associated GTPase |
| TON_0669 | TK0504 | 85.53 | 2.00E-103 | 367 | aspartate racemase |
| TON_0670 | TK0503 | 93.81 | 1.00E-59 | 219 | hypothetical protein |
| TON_0671 | TK0502 | 90.77 | 0 | 821 | zinc-dependent protease |
| TON_0672 | TK0499 | 82.54 | 0 | 739 | zinc-dependent protease |

| | | | | | |
|---|---|---|---|---|---|
| TON_0673 | TK0497 | 85.2 | 8.00E-159 | 551 | Met-10+ like protein |
| TON_0675 | TK0422 | 81.62 | 2.00E-164 | 571 | tRNA m1G methyltransferase |
| TON_0677 | TK1274 | 91.25 | 0 | 1638 | valS valyl-tRNA synthetase |
| TON_1972 | TK1273 | 60.26 | 4.00E-78 | 283 | SAM-dependent methyltransferase |
| TON_0678 | TK0864 | 75.31 | 7.00E-139 | 485 | putative aminotransferase |
| TON_0679 | TK0863 | 67.58 | 8.00E-109 | 385 | cobD cobalamin biosynthesis protein |
| TON_0681 | TK0862 | 55 | 9.00E-23 | 97.1 | hypothetical protein |
| TON_0682 | TK0861 | 69.57 | 5.00E-72 | 263 | ATPase |
| TON_0683 | TK0858 | 81.2 | 7.00E-54 | 201 | hypothetical protein |
| TON_0684 | TK0857 | 72.73 | 1.00E-87 | 315 | cobS cobalamin synthase |
| TON_0686 | TK0856 | 76.97 | 2.00E-80 | 290 | GTP:adenosylcobinamide-phosphate guanylyltransferase |
| TON_0687 | TK0854 | 83.44 | 0 | 800 | cobyric acid synthase |
| TON_0688 | TK0853 | 82.04 | 8.00E-158 | 548 | hypothetical protein |
| TON_0689 | TK0846 | 82.72 | 3.00E-93 | 333 | adenosylcobinamide amidohydrolase |
| TON_0690 | TK0544 | 70.18 | 1.00E-60 | 224 | molybdenum cofactor biosynthesis protein B |
| TON_0691 | TK0541 | 84.34 | 0 | 635 | molybdenum cofactor biosynthesis protein MoeA |
| TON_0693 | TK2187 | 34.37 | 3.00E-50 | 191 | membrane protein |
| TON_0696 | TK0540 | 82.32 | 0 | 702 | metal-dependent phosphohydrolase |
| TON_0697 | TK0539 | 80.94 | 3.00E-135 | 473 | hypothetical protein |
| TON_0698 | TK0538 | 65.34 | 8.00E-68 | 248 | N-acetyltransferase |
| TON_0699 | TK0553 | 85.61 | 0 | 762 | nucleic acid-binding protein |
| TON_0701 | TK0671 | 91.94 | 0 | 637 | putative deoxyhypusine synthase |
| TON_0702 | TK0672 | 80.17 | 3.00E-158 | 550 | putative glutamate synthase subunit beta |
| TON_0703 | TK0673 | 82.24 | 4.00E-124 | 436 | glucose-1-dehydrogenase |
| TON_0705 | TK0677 | 85.86 | 2.00E-111 | 394 | heat shock protein HtpX |
| TON_0706 | TK0353 | 78.7 | 1.00E-79 | 287 | hypothetical protein |
| TON_0707 | TK0678 | 89.43 | 0 | 926 | chaperonin subunit alpha |
| TON_0709 | TK0679 | 79.64 | 7.00E-96 | 342 | serine/threonine protein kinase |
| TON_0711 | TK0682 | 72.3 | 0 | 777 | MutS-like DNA mismatch repair ATPase |
| TON_0713 | TK0354 | 89.68 | 7.00E-77 | 278 | putative molybdenum cofactor biosynthesis protein MoaC |
| TON_0714 | TK0358 | 93.95 | 0 | 884 | RNA terminal phosphate cyclase |
| TON_0715 | TK0359 | 74.17 | 5.00E-60 | 222 | hypothetical protein |
| TON_0716 | TK0360 | 86.13 | 2.00E-162 | 563 | tRNA/rRNA cytosine-C5-methylase |
| TON_0717 | TK0361 | 82.39 | 2.00E-37 | 148 | hypothetical protein |
| TON_0718 | TK0363 | 87.68 | 1.00E-132 | 464 | panB 3-methyl-2-oxobutanoate hydroxymethyltransferase |
| TON_0719 | TK0364 | 77.73 | 1.00E-93 | 335 | dolichol-phosphate mannosyltransferase |
| TON_0720 | TK1876 | 52.33 | 9.00E-37 | 146 | hypothetical protein |
| TON_0722 | TK0365 | 48.36 | 5.00E-64 | 236 | membrane-bound phosphoesterase |
| TON_0723 | TK0518 | 74.32 | 2.00E-61 | 226 | hypothetical protein |
| TON_0724 | TK0519 | 87.8 | 3.00E-99 | 352 | hypothetical protein |
| TON_0725 | TK0521 | 80.2 | 0 | 1402 | DEAD/DEAH box helicase |
| TON_0727 | TK1031 | 87.16 | 0 | 1085 | putative ATPase RIL |
| TON_0736 | TK1032 | 63.83 | 1.00E-121 | 428 | HypE protein |
| TON_0737 | TK1034 | 60.46 | 4.00E-91 | 327 | restriction endonuclease |
| TON_0738 | TK0710 | 72.36 | 8.00E-100 | 355 | hypothetical protein |
| TON_0739 | TK1035 | 49.8 | 3.00E-64 | 237 | glutamine amidotransferase |
| TON_0740 | TK1036 | 73.53 | 7.00E-55 | 204 | hypothetical protein |

| | | | | | |
|---|---|---|---|---|---|
| TON_0741 | TK1037 | 78.51 | 0 | 823 | metallophosphoesterase |
| TON_0742 | TK1038 | 70.14 | 2.00E-115 | 407 | 2-phosphoglycerate kinase |
| TON_0743 | TK1039 | 78.2 | 0 | 668 | cyclic 2,3-diphospoglycerate synthetase |
| TON_0744 | TK1040 | 74.58 | 4.00E-15 | 72 | hypothetical protein |
| TON_0745 | TK0029 | 70.45 | 5.00E-122 | 429 | zinc-dependent hydrolase |
| TON_0746 | TK0069 | 77.4 | 2.00E-100 | 357 | exonuclease |
| TON_0747 | TK0070 | 61.26 | 2.00E-36 | 143 | hypothetical protein |
| TON_0748 | TK0071 | 83.09 | 7.00E-136 | 475 | hypothetical protein |
| TON_0749 | TK0072 | 36.04 | 7.00E-94 | 337 | formaldehyde:ferredoxin oxidoreductase |
| TON_0751 | TK0307 | 99.02 | 4.00E-54 | 201 | rps10p 30S ribosomal protein S10P |
| TON_0752 | TK0308 | 96.5 | 0 | 814 | elongation factor 1-alpha |
| TON_0755 | TK0309 | 93.72 | 0 | 1409 | elongation factor EF-2 |
| TON_0756 | TK0312 | 67.42 | 7.00E-139 | 485 | metal-dependent phosphohydrolase |
| TON_0757 | TK0793 | 89.77 | 4.00E-130 | 456 | GTP cyclohydrolase |
| TON_0761 | TK0796 | 68.8 | 2.00E-49 | 186 | DNA-binding protein |
| TON_0762 | TK0797 | 70.26 | 2.00E-160 | 557 | hypothetical protein |
| TON_0763 | TK0798 | 89.9 | 0 | 726 | DNA topoisomerase VI subunit A |
| TON_0764 | TK0799 | 89.82 | 0 | 966 | DNA topoisomerase VI subunit B |
| TON_0765 | TK0800 | 87.39 | 8.00E-97 | 345 | putative RNA-processing protein |
| TON_0766 | TK0801 | 88.62 | 2.00E-128 | 450 | serine/threonine protein kinase |
| TON_0767 | TK0802 | 87.83 | 7.00E-55 | 204 | translation initiation factor IF-1 |
| TON_0768 | TK0803 | 76.15 | 3.00E-104 | 370 | ABC-type manganese/zinc transport system, ATPase component |
| TON_0769 | TK0804 | 78.39 | 1.00E-109 | 388 | manganese/zinc ABC transporter permease |
| TON_0770 | TK0805 | 74.01 | 5.00E-95 | 339 | rnhB ribonuclease HII |
| TON_0771 | TK0806 | 62.82 | 5.00E-137 | 480 | membrane-bound dolichyl-phosphate-mannose-protein mannosyltransferase |
| TON_0772 | TK0807 | 76.52 | 1.00E-175 | 608 | hypothetical protein |
| TON_0773 | TK0808 | 71.88 | 1.00E-15 | 73.6 | hypothetical protein |
| TON_0774 | TK0809 | 82.72 | 0 | 929 | glucosamine--fructose-6-phosphate aminotransferase |
| TON_0775 | TK0810 | 51.72 | 0 | 947 | oligosaccharyl transferase |
| TON_0777 | TK0704 | 35.64 | 2.00E-22 | 98.2 | UbiE/COQ5 methyltransferase |
| TON_0779 | TK1065 | 47.87 | 2.00E-16 | 76.3 | molybdopterin converting factor protein subunit 1 |
| TON_0780 | TK0812 | 73.66 | 4.00E-95 | 339 | adk adenylate kinase |
| TON_0781 | TK0813 | 64.63 | 2.00E-26 | 110 | hypothetical protein |
| TON_0782 | TK0554 | 68.84 | 3.00E-79 | 286 | hypothetical protein |
| TON_0785 | TK0511 | 81.59 | 0 | 811 | pyruvate kinase |
| TON_0786 | TK1055 | 78.99 | 1.00E-53 | 201 | peroxiredoxin, bacterioferritin comigratory protein |
| TON_0788 | TK1823 | 48.48 | 5.00E-11 | 58.2 | hypothetical protein |
| TON_0790 | TK0512 | 50 | 1.00E-61 | 228 | putative zinc-dependent protease |
| TON_0791 | TK0513 | 65.16 | 5.00E-104 | 369 | hypothetical protein |
| TON_0795 | TK0514 | 85.58 | 4.00E-33 | 132 | camphor resistance protein CrcB |
| TON_0796 | TK0515 | 85.6 | 2.00E-60 | 223 | hypothetical protein |
| TON_0797 | TK0516 | 38.55 | 2.00E-31 | 127 | hypothetical protein |
| TON_0798 | TK0517 | 89.97 | 0 | 706 | bifunctional phosphopantothenoylcysteine decarboxylase/phosphopantothenate synthase |
| TON_0799 | TK1063 | 72.84 | 1.00E-107 | 381 | small-conductance mechanosensitive channel |
| TON_0801 | TK1471 | 69.47 | 1.00E-65 | 241 | aspartate racemase |
| TON_0802 | TK0700 | 88.66 | 1.00E-48 | 182 | nucleotide pyrophosphohydrolase |

| | | | | | |
|---|---|---|---|---|---|
| TON_0803 | TK0833 | 58.68 | 1.00E-97 | 348 | permease |
| TON_0804 | TK0699 | 88.79 | 0 | 788 | zinc-dependent protease |
| TON_0805 | TK0698 | 88.41 | 0 | 814 | zinc-dependent protease |
| TON_0806 | TK1927 | 59.06 | 5.00E-58 | 216 | membrane protein |
| TON_0808 | TK0689 | 79.08 | 3.00E-59 | 219 | membrane protein |
| TON_0809 | TK0688 | 49.47 | 6.00E-49 | 186 | membrane-associated phosphatase |
| TON_0816 | TK0687 | 89.12 | 3.00E-99 | 353 | hypothetical protein |
| TON_0817 | TK0686 | 79.2 | 6.00E-107 | 379 | hydrolase |
| TON_0818 | TK1074 | 47.33 | 4.00E-27 | 112 | hypothetical protein |
| TON_0819 | TK0685 | 75.2 | 4.00E-113 | 399 | NAD-dependent deacetylase |
| TON_0820 | TK0684 | 78.39 | 7.00E-159 | 552 | aromatic amino acid permease |
| TON_0821 | TK0528 | 87.62 | 0 | 739 | glyA serine hydroxymethyltransferase |
| TON_0823 | TK0532 | 58.39 | 1.00E-43 | 167 | hypothetical protein |
| TON_0824 | TK0533 | 87.27 | 1.00E-55 | 206 | DNA-directed RNA polymerase subunit M |
| TON_0826 | TK0535 | 90.36 | 7.00E-106 | 375 | DNA polymerase sliding clamp |
| TON_0827 | TK0536 | 65.78 | 8.00E-58 | 215 | hypothetical protein |
| TON_0828 | TK0690 | 82.68 | 0 | 1098 | molybdopterin oxidoreductase, molybdopterin-binding subunit |
| TON_0829 | TK0537 | 89.35 | 4.00E-105 | 372 | peroxiredoxin |
| TON_0830 | TK0692 | 70.09 | 2.00E-85 | 307 | permease |
| TON_0831 | TK0693 | 59.13 | 4.00E-64 | 236 | membrane protein |
| TON_0832 | TK0694 | 85.16 | 4.00E-143 | 499 | ATP-binding transport protein |
| TON_0834 | TK0838 | 55.17 | 6.00E-22 | 94.7 | hypothetical protein |
| TON_0835 | TK0837 | 79.39 | 0 | 1274 | heavy-metal transporting P-type ATPase |
| TON_0836 | TK0834 | 78.53 | 5.00E-69 | 252 | transcriptional regulatory protein |
| TON_0845 | TK0005 | 36.21 | 4.00E-15 | 72.8 | hypothetical protein |
| TON_0847 | TK0831 | 88.46 | 5.00E-23 | 99.8 | alkyl hydroperoxide reductase subunit c |
| TON_0852 | TK1999 | 64.52 | 1.00E-57 | 214 | hypothetical protein |
| TON_0860 | TK0817 | 79.3 | 0 | 880 | 2-oxoacid:ferredoxin oxidoreductase subunit alpha |
| TON_0861 | TK0816 | 87.97 | 4.00E-158 | 549 | 2-oxoacid:ferredoxin oxidoreductase subunit beta |
| TON_0862 | TK0815 | 73.47 | 2.00E-64 | 236 | thioredoxin peroxidase |
| TON_0863 | TK0814 | 82.31 | 0 | 672 | type A flavoprotein |
| TON_0865 | TK0828 | 63.69 | 6.00E-128 | 449 | NAD(P)H:rubredoxin oxidoreductase |
| TON_0866 | TK0523 | 83.63 | 3.00E-77 | 280 | rubrerythrin |
| TON_0867 | TK0524 | 94.34 | 4.00E-25 | 105 | rubredoxin |
| TON_0868 | TK0525 | 89.57 | 1.00E-59 | 219 | sor superoxide reductase |
| TON_0869 | TK0527 | 82.05 | 4.00E-55 | 205 | hypothetical protein |
| TON_0871 | TK0650 | 81.03 | 6.00E-72 | 261 | rubrerythrin-like protein |
| TON_0872 | TK1060 | 64 | 5.00E-26 | 108 | hypothetical protein |
| TON_0873 | TK1056 | 78.91 | 2.00E-62 | 230 | rubrerythrin-like protein |
| TON_0874 | TK1054 | 77.19 | 9.00E-73 | 265 | N-acetyltransferase |
| TON_0877 | TK0826 | 82.18 | 3.00E-81 | 293 | hypothetical protein |
| TON_0878 | TK0242 | 61.27 | 9.00E-93 | 332 | ATP phosphoribosyltransferase |
| TON_0879 | TK0243 | 69.12 | 2.00E-77 | 280 | hisG ATP phosphoribosyltransferase catalytic subunit |
| TON_0880 | TK0244 | 79.62 | 3.00E-161 | 560 | hisD histidinol dehydrogenase |
| TON_0881 | TK0245 | 75.71 | 3.00E-63 | 233 | hisB imidazoleglycerol-phosphate dehydratase |
| TON_0882 | TK0246 | 85.71 | 2.00E-94 | 336 | hisH imidazole glycerol phosphate synthase subunit HisH |

| | | | | | |
|---|---|---|---|---|---|
| TON_0883 | TK0247 | 71.23 | 2.00E-88 | 317 | 1-(5-phosphoribosyl)-5-[(5-phosphoribosylamino)methylideneamino] imidazole-4-carboxamide isomerase |
| TON_0884 | TK0248 | 83.33 | 3.00E-115 | 406 | imidazole glycerol phosphate synthase subunit HisF |
| TON_0885 | TK0249 | 80.77 | 8.00E-100 | 355 | bifunctional phosphoribosyl-AMP cyclohydrolase/phosphoribosyl-ATP pyrophosphatase |
| TON_0886 | TK0250 | 57.31 | 2.00E-98 | 351 | histidinol-phosphate aminotransferase |
| TON_0887 | TK0251 | 57.08 | 8.00E-68 | 249 | hydrolase |
| TON_0888 | TK0273 | 78.38 | 5.00E-63 | 233 | pyrroline-5-carboxylate reductase |
| TON_0891 | TK0981 | 82.78 | 9.00E-164 | 568 | N2, N2-dimethylguanosine tRNA methyltransferase |
| TON_0899 | TK0984 | 90.14 | 5.00E-103 | 365 | alanyl-tRNA synthetase |
| TON_0900 | TK0985 | 23.69 | 2.00E-33 | 135 | hypothetical protein |
| TON_0901 | TK0986 | 77.44 | 2.00E-180 | 624 | proton/glutamate symporter |
| TON_0902 | TK0987 | 55.6 | 2.00E-65 | 241 | biotin--protein ligase |
| TON_0903 | TK0989 | 90.39 | 8.00E-136 | 475 | fructose-bisphosphate aldolase |
| TON_0904 | TK0990 | 82.6 | 0 | 968 | pyruvate carboxylase subunit B |
| TON_0905 | TK0991 | 65.08 | 0 | 632 | cstA |
| TON_0906 | TK0993 | 72.83 | 2.00E-36 | 142 | hypothetical protein |
| TON_0907 | TK0994 | 76.22 | 7.00E-134 | 469 | arsA protein |
| TON_0908 | TK0995 | 41.67 | 8.00E-15 | 70.9 | hypothetical protein |
| TON_0909 | TK0996 | 73.56 | 1.00E-90 | 324 | membrane protein |
| TON_0910 | TK0999 | 82.1 | 4.00E-116 | 409 | lysophospholipase |
| TON_0911 | TK1002 | 88.79 | 6.00E-173 | 598 | adenylosuccinate synthetase |
| TON_0912 | TK1004 | 87.42 | 1.00E-160 | 557 | UDP-glucose 4-epimerase |
| TON_0915 | TK0701 | 81.85 | 7.00E-129 | 452 | ATPase, N-terminus |
| TON_0916 | TK0702 | 74.39 | 2.00E-123 | 434 | ATPase C-terminus |
| TON_0917 | TK0723 | 83.72 | 9.00E-63 | 231 | iron-molybdenum cofactor-binding protein |
| TON_0918 | TK0721 | 66.67 | 1.00E-64 | 238 | hypothetical protein |
| TON_0924 | TK0742 | 59.44 | 2.00E-41 | 160 | membrane protein |
| TON_0925 | TK0565 | 60.87 | 4.00E-45 | 172 | DNA-binding protein |
| TON_0926 | TK0564 | 53.01 | 4.00E-43 | 166 | hypothetical protein |
| TON_0927 | TK0563 | 85.43 | 4.00E-75 | 272 | 6-pyruvoyl-tetrahydropterin synthase |
| TON_0928 | TK0562 | 88.04 | 4.00E-43 | 165 | hypothetical protein |
| TON_0929 | TK0561 | 90.47 | 0 | 849 | adenylosuccinate lyase |
| TON_0930 | TK0560 | 97.8 | 2.00E-47 | 179 | DNA/RNA-binding protein albA |
| TON_0931 | TK0559 | 47.6 | 2.00E-45 | 174 | transcriptional regulator |
| TON_0932 | TK0558 | 77.53 | 1.00E-71 | 261 | hypothetical protein |
| TON_0933 | TK0557 | 73.3 | 4.00E-69 | 252 | putative NTPase |
| TON_0934 | TK0556 | 89.33 | 2.00E-179 | 620 | translation initiation factor IF-2 |
| TON_0936 | TK1008 | 84.27 | 6.00E-169 | 585 | Fe-containing alcohol dehydrogenase |
| TON_0937 | TK1015 | 85.34 | 0 | 1617 | large helicase-like protein |
| TON_0938 | TK0313 | 85.96 | 2.00E-174 | 603 | acetylornithine deacetylase |
| TON_0939 | TK0314 | 66.67 | 3.00E-92 | 330 | hydrolase |
| TON_0940 | TK0315 | 86.25 | 2.00E-36 | 142 | hypothetical protein |
| TON_0941 | TK0317 | 68.65 | 2.00E-101 | 360 | TatD-related deoxyribonuclease |
| TON_0943 | TK0319 | 83.5 | 0 | 828 | Pterin-binding protein |
| TON_0944 | TK0320 | 82.7 | 6.00E-122 | 428 | hypothetical protein |
| TON_0945 | TK0683 | 84.68 | 4.00E-161 | 559 | glyoxylate reductase |

| | | | | | |
|---|---|---|---|---|---|
| TON_0947 | TK1116 | 71.53 | 9.00E-180 | 622 | 23S rRNA (uracil-5-)-methyltransferase |
| TON_0951 | TK1307 | 91.18 | 2.00E-89 | 320 | ndk nucleoside diphosphate kinase |
| TON_0952 | TK1309 | 97.01 | 9.00E-35 | 137 | 50S ribosomal protein L24e |
| TON_0954 | TK1311 | 94.31 | 1.00E-52 | 197 | rpl7ae 50S ribosomal protein L7Ae |
| TON_0955 | TK1313 | 87.56 | 9.00E-94 | 335 | hypothetical protein |
| TON_0957 | TK1315 | 74.53 | 0 | 927 | hypothetical protein |
| TON_0958 | TK1316 | 89.27 | 5.00E-143 | 499 | membrane protease subunit |
| TON_0959 | TK1317 | 66.94 | 1.00E-28 | 117 | membrane protein |
| TON_0960 | TK0758 | 61.4 | 2.00E-149 | 521 | metal-dependent amidohydrolase |
| TON_0961 | TK0759 | 91.88 | 0 | 817 | asnC asparaginyl-tRNA synthetase |
| TON_0963 | TK0757 | 66.39 | 9.00E-138 | 482 | thiamin-binding periplasmic protein |
| TON_0964 | TK0867 | 44.19 | 2.00E-50 | 192 | Ca2+/Na+ antiporter |
| TON_0965 | TK0756 | 80.25 | 8.00E-66 | 241 | hypothetical protein |
| TON_0966 | TK0755 | 85.62 | 2.00E-77 | 280 | hypothetical protein |
| TON_0968 | TK0754 | 92.22 | 0 | 1441 | ATPase |
| TON_0969 | TK0752 | 89.54 | 0 | 1187 | acylamino acid-releasing protein |
| TON_0970 | TK0749 | 72.54 | 6.00E-82 | 295 | molybdopterin-guanine dinucleotide biosynthesis protein A |
| TON_0973 | TK0748 | 61.22 | 2.00E-38 | 150 | hypothetical protein |
| TON_0974 | TK0746 | 73.95 | 8.00E-112 | 395 | cobalt ABC transporter ATPase |
| TON_0975 | TK0745 | 61.51 | 1.00E-60 | 225 | cobalt ABC transporter permease |
| TON_0976 | TK0744 | 54.33 | 2.00E-60 | 224 | hypothetical protein |
| TON_0978 | TK0743 | 80.08 | 8.00E-119 | 418 | membrane protein |
| TON_0979 | TK0566 | 91.79 | 0 | 1535 | DEAD/DEAH box helicase |
| TON_0980 | TK0567 | 58.28 | 9.00E-48 | 181 | transcriptional regulator |
| TON_0981 | TK0568 | 95.2 | 0 | 715 | tyrosyl-tRNA synthetase |
| TON_0983 | TK0570 | 77.68 | 0 | 724 | iron(III) ABC transporter periplasmic protein |
| TON_0984 | TK0571 | 75.85 | 0 | 845 | iron(III) ABC transporter permease |
| TON_0985 | TK0572 | 84.66 | 3.00E-177 | 613 | ABC-type iron(III) transport system, ATPase component |
| TON_0986 | TK0573 | 48.68 | 8.00E-43 | 165 | hypothetical protein |
| TON_0987 | TK0574 | 45.51 | 3.00E-162 | 564 | metallophosphoesterase |
| TON_0988 | TK1148 | 88.14 | 6.00E-96 | 342 | hypothetical protein |
| TON_0989 | TK1147 | 55.6 | 0 | 781 | type II restriction endonuclease |
| TON_0990 | TK1146 | 83.17 | 0 | 714 | pgk phosphoglycerate kinase |
| TON_0992 | TK1145 | 61.96 | 1.00E-85 | 308 | ABC type transporter permease |
| TON_0993 | TK1144 | 84.36 | 2.00E-155 | 540 | ABC-type multidrug transporter, ATPase component |
| TON_0994 | TK1143 | 54.73 | 2.00E-29 | 120 | hypothetical protein |
| TON_0995 | TK1142 | 58.74 | 8.00E-45 | 171 | hypothetical protein |
| TON_0996 | TK1141 | 84.23 | 1.00E-123 | 434 | endonuclease III |
| TON_0997 | TK1456 | 75.14 | 5.00E-71 | 259 | phosphate transport system regulator |
| TON_0998 | TK1457 | 74.88 | 5.00E-77 | 279 | phosphate transport system regulator |
| TON_0999 | TK1458 | 40.1 | 4.00E-67 | 247 | hypothetical protein |
| TON_1001 | TK0944 | 89.79 | 0 | 739 | acyl-CoA synthetase large subunit |
| TON_1002 | TK0943 | 90.38 | 8.00E-123 | 431 | acetyl-CoA synthetase II subunit beta |
| TON_1004 | TK1687 | 74.07 | 4.00E-120 | 423 | cysteine synthase |
| TON_1006 | TK0366 | 72.77 | 4.00E-81 | 293 | hypothetical protein |
| TON_1007 | TK0367 | 65.54 | 4.00E-55 | 206 | membrane protein |
| TON_1008 | TK0368 | 88.98 | 0 | 661 | thiamine biosynthesis protein ThiI |

| | | | | | |
|---|---|---|---|---|---|
| TON_1009 | TK0369 | 74.04 | 4.00E-25 | 105 | membrane protein |
| TON_1010 | TK0370 | 74.26 | 1.00E-77 | 281 | NAD(P)H-flavin oxidoreductase |
| TON_1011 | TK0375 | 87.76 | 6.00E-123 | 432 | maleate cis-trans isomerase |
| TON_1012 | TK0376 | 85.37 | 0 | 753 | ADP-specific phosphofructokinase |
| TON_1013 | TK0777 | 80 | 2.00E-123 | 434 | integrase/recombinase |
| TON_1014 | TK0778 | 76 | 1.00E-125 | 441 | hypothetical protein |
| TON_1015 | TK0779 | 82.4 | 1.00E-115 | 407 | sugar fermentation stimulation protein A |
| TON_1032 | TK0781 | 94.54 | 0 | 663 | deblocking aminopeptidase |
| TON_1034 | TK0322 | 25.65 | 1.00E-11 | 63.5 | hypothetical protein |
| TON_1035 | TK0324 | 36.41 | 3.00E-18 | 84.3 | membrane protein |
| TON_1037 | TK0326 | 56.36 | 1.00E-70 | 258 | multidrug ABC transporter ATPase |
| TON_1040 | TK0784 | 84.25 | 0 | 1122 | ERCC2/XPD/Rad3-related DNA repair helicase |
| TON_1041 | TK1753 | 38.06 | 1.00E-43 | 168 | hypothetical protein |
| TON_1042 | TK0306 | 71.12 | 6.00E-153 | 532 | DEAD/DEAH box helicase |
| TON_1045 | TK0787 | 71.65 | 9.00E-108 | 381 | bifunctional inositol-1 monophosphatase/fructose-1,6-bisphosphatase |
| TON_1046 | TK0788 | 73.61 | 6.00E-111 | 392 | membrane protein |
| TON_1047 | TK0789 | 83.14 | 3.00E-170 | 590 | egsA NAD(P)-dependent glycerol-1-phosphate dehydrogenase |
| TON_1048 | TK0790 | 86.81 | 2.00E-70 | 256 | hypothetical protein |
| TON_1049 | TK0332 | 70.43 | 5.00E-140 | 489 | Allophanate hydrolase, subunit 2 |
| TON_1050 | TK0333 | 69.37 | 2.00E-82 | 297 | hypothetical protein |
| TON_1051 | TK0336 | 85.49 | 8.00E-130 | 455 | LamB/YcsF family protein |
| TON_1053 | TK0339 | 74.05 | 8.00E-39 | 151 | hypothetical protein |
| TON_1055 | TK0345 | 71.84 | 5.00E-110 | 389 | 3-methyladenine DNA glycosylase |
| TON_1056 | TK0346 | 60.82 | 2.00E-30 | 122 | hypothetical protein |
| TON_1057 | TK0347 | 64.37 | 1.00E-141 | 495 | nodulation protein nfeD |
| TON_1058 | TK0348 | 91.03 | 7.00E-101 | 358 | membrane protease subunit |
| TON_1059 | TK0349 | 93.82 | 5.00E-123 | 432 | ATPase |
| TON_1062 | TK0352 | 89.86 | 0 | 893 | thymidine phosphorylase |
| TON_1063 | TK1030 | 79.84 | 4.00E-50 | 188 | hypothetical protein |
| TON_1064 | TK1029 | 78.87 | 1.00E-54 | 204 | hypothetical protein |
| TON_1065 | TK1026 | 87.35 | 7.00E-119 | 418 | geranylgeranylglyceryl phosphate synthase |
| TON_1066 | TK1023 | 48.1 | 4.00E-19 | 85.1 | hypothetical protein |
| TON_1067 | TK1022 | 64.26 | 6.00E-101 | 359 | D-aminopeptidase |
| TON_1069 | TK1021 | 85.29 | 0 | 1283 | Hef nuclease |
| TON_1070 | TK1019 | 68.35 | 4.00E-43 | 166 | hypothetical protein |
| TON_1071 | TK1018 | 84.16 | 4.00E-75 | 273 | hypothetical protein |
| TON_1072 | TK1017 | 80.52 | 0 | 1544 | chromosome segregation ATPase |
| TON_1073 | TK1016 | 48.82 | 7.00E-33 | 131 | hypothetical protein |
| TON_1074 | TK0330 | 90.91 | 7.00E-66 | 241 | methylmalonyl-CoA epimerase |
| TON_1075 | TK0329 | 88.09 | 6.00E-166 | 575 | lysine/arginine/ornithine transport system kinase |
| TON_1076 | TK0328 | 82.52 | 7.00E-65 | 238 | methylmalony-CoA mutase |
| TON_1077 | TK0327 | 70.28 | 3.00E-89 | 320 | hypothetical protein |
| TON_1078 | TK0954 | 79.57 | 3.00E-85 | 306 | Maf-like protein |
| TON_1080 | TK0953 | 92.86 | 0 | 1056 | ATPase |
| TON_1081 | TK0952 | 79.92 | 3.00E-109 | 387 | ATPase |
| TON_1082 | TK0951 | 75.3 | 1.00E-111 | 394 | GTPase |
| TON_1083 | TK0950 | 65.97 | 3.00E-179 | 620 | asparagine synthase |

| | | | | | |
|---|---|---|---|---|---|
| TON_1085 | TK0949 | 69.77 | 2.00E-140 | 490 | glycosyltransferase |
| TON_1086 | TK0948 | 79.94 | 2.00E-151 | 527 | putative translation factor |
| TON_1087 | TK0947 | 51.3 | 1.00E-104 | 372 | hypothetical protein |
| TON_1088 | TK0946 | 58.27 | 7.00E-51 | 191 | hypothetical protein |
| TON_1089 | TK0945 | 87.61 | 1.00E-117 | 414 | nucleotidyltransferase |
| TON_1090 | TK1140 | 91.63 | 0 | 850 | seryl-tRNA synthetase |
| TON_1091 | TK1282 | 87.5 | 0 | 693 | molybdenum cofactor biosynthesis protein MoeA |
| TON_1093 | TK0670 | 74.41 | 2.00E-107 | 380 | hypothetical protein |
| TON_1094 | TK0669 | 86.28 | 0 | 1340 | CDC48/VCP |
| TON_1095 | TK0668 | 82.69 | 7.00E-43 | 164 | hypothetical protein |
| TON_1096 | TK0667 | 82.05 | 9.00E-119 | 418 | metallophosphoesterase |
| TON_1097 | TK0665 | 91.03 | 0 | 780 | acetyl-CoA synthetase I subunit alpha |
| TON_1099 | TK1281 | 94.41 | 7.00E-143 | 499 | flap endonuclease-1 |
| TON_1100 | TK1280 | 91.64 | 5.00E-152 | 529 | tfb transcription initiation factor IIB |
| TON_1102 | TK1278 | 87.5 | 1.00E-50 | 190 | hypothetical protein |
| TON_1103 | TK1276 | 94.67 | 5.00E-81 | 291 | 30S ribosomal protein S19e |
| TON_1104 | TK1275 | 80.88 | 6.00E-50 | 188 | RNA-binding protein |
| TON_1105 | TK1283 | 79.9 | 3.00E-92 | 329 | hypothetical protein |
| TON_1106 | TK1322 | 92.21 | 1.00E-24 | 103 | rplX 50S ribosomal protein LX |
| TON_1107 | TK1321 | 83.77 | 4.00E-109 | 386 | translation initiation factor IF-6 |
| TON_1108 | TK1320 | 81.11 | 1.00E-23 | 99.8 | 50S ribosomal protein L31e |
| TON_1110 | TK1149 | 92.35 | 0 | 1080 | methylmalonyl-CoA mutase, N-terminus of large subunit |
| TON_1111 | TK1153 | 83.99 | 5.00E-144 | 502 | hypothetical protein |
| TON_1113 | TK1155 | 82.82 | 5.00E-65 | 238 | small heat shock protein |
| TON_1114 | TK1157 | 90.7 | 0 | 1334 | CDC48/VCP |
| TON_1117 | TK1159 | 63.27 | 1.00E-13 | 67 | hypothetical protein |
| TON_1118 | TK0938 | 53.06 | 2.00E-12 | 64.3 | hypothetical protein |
| TON_1119 | TK1033 | 57.25 | 2.00E-39 | 153 | metalloprotease |
| TON_1120 | TK0934 | 79.51 | 1.00E-117 | 414 | putative molybdopterin-guanine dinucleotide biosynthesis protein MobB/FeS domain-containing protein |
| TON_1121 | TK0933 | 76.06 | 1.00E-27 | 113 | hypothetical protein |
| TON_1122 | TK0930 | 60.58 | 2.00E-98 | 350 | hypothetical protein |
| TON_1124 | TK0293 | 80.35 | 1.00E-102 | 364 | hypothetical protein |
| TON_1125 | TK0292 | 72.19 | 2.00E-61 | 226 | hypothetical protein |
| TON_1128 | TK0284 | 82.89 | 2.00E-82 | 297 | NAD(P)H-flavin oxidoreductase |
| TON_1130 | TK0271 | 80.58 | 2.00E-43 | 166 | hypothetical protein |
| TON_1131 | TK0270 | 77.52 | 4.00E-89 | 319 | transketolase, N-terminal section |
| TON_1132 | TK0269 | 77.81 | 7.00E-144 | 502 | transketolase |
| TON_1133 | TK0268 | 85.44 | 1.00E-133 | 468 | 3-deoxy-7-phosphoheptulonate synthase |
| TON_1134 | TK0267 | 72.31 | 2.00E-122 | 431 | aroB 3-dehydroquinate synthase |
| TON_1135 | TK0266 | 59.22 | 1.00E-63 | 234 | aroD 3-dehydroquinate dehydratase |
| TON_1136 | TK0265 | 65.93 | 8.00E-84 | 302 | aroE shikimate 5-dehydrogenase |
| TON_1137 | TK0264 | 66.05 | 4.00E-83 | 300 | shikimate kinase |
| TON_1138 | TK0263 | 65.66 | 3.00E-141 | 493 | 3-phosphoshikimate 1-carboxyvinyltransferase |
| TON_1139 | TK0262 | 76.64 | 1.00E-149 | 521 | chorismate synthase |
| TON_1140 | TK0261 | 59.18 | 5.00E-11 | 58.2 | chorismate mutase |
| TON_1141 | TK0260 | 68.47 | 7.00E-136 | 476 | aromatic aminotransferase |

| | | | | | |
|---|---|---|---|---|---|
| TON_1142 | TK0259 | 61.33 | 4.00E-85 | 306 | prephenate dehydrogenase |
| TON_1143 | TK0241 | 73.94 | 2.00E-58 | 216 | protein-tyrosine phosphatase |
| TON_1149 | TK0236 | 49.68 | 1.00E-71 | 262 | hypothetical protein |
| TON_1155 | TK0225 | 73.65 | 4.00E-59 | 219 | hypothetical protein |
| TON_1156 | TK0224 | 65.69 | 1.00E-74 | 271 | SAM-dependent methyltransferase |
| TON_1158 | TK0232 | 55.15 | 7.00E-79 | 285 | Acetyltransferase (GNAT) family protein |
| TON_1159 | TK0229 | 65.15 | 5.00E-19 | 85.9 | hypothetical protein |
| TON_1160 | TK0223 | 77.05 | 1.00E-164 | 571 | hypothetical protein |
| TON_1164 | TK0067 | 94.15 | 8.00E-101 | 358 | nicotinamide-nucleotide adenylyltransferase |
| TON_1165 | TK0062 | 78.91 | 6.00E-120 | 422 | hypothetical protein |
| TON_1166 | TK0061 | 51.56 | 3.00E-167 | 580 | hypothetical protein |
| TON_1167 | TK0060 | 85.31 | 2.00E-88 | 317 | tRNA 2'-O-methylase |
| TON_1168 | TK0059 | 79.79 | 1.00E-82 | 297 | CDP-alcohol phosphatidyltransferase |
| TON_1169 | TK0058 | 78.88 | 3.00E-108 | 383 | hydrolase |
| TON_1170 | TK0057 | 65.56 | 5.00E-25 | 107 | hypothetical protein |
| TON_1171 | TK0056 | 84.13 | 8.00E-92 | 328 | hypothetical protein |
| TON_1172 | TK0055 | 83.78 | 2.00E-52 | 196 | hypothetical protein |
| TON_1174 | TK0053 | 51.23 | 2.00E-89 | 321 | signal peptidase |
| TON_1175 | TK0052 | 78.64 | 2.00E-93 | 333 | phosphoserine phosphatase |
| TON_1176 | TK0051 | 78.24 | 2.00E-74 | 270 | pcm protein-L-isoaspartate O-methyltransferase |
| TON_1177 | TK0050 | 36.38 | 1.00E-90 | 326 | membrane protein |
| TON_1178 | TK0049 | 80.48 | 0 | 787 | flagellar assembly protein J |
| TON_1179 | TK0048 | 91.22 | 0 | 1001 | flagella-related protein I |
| TON_1180 | TK0047 | 94.4 | 1.00E-126 | 444 | flagellar accessory protein FlaH |
| TON_1181 | TK0046 | 79.25 | 7.00E-64 | 235 | flagella-related protein G |
| TON_1182 | TK0045 | 43.82 | 4.00E-16 | 76.6 | flagella-related protein |
| TON_1184 | TK0043 | 85.44 | 9.00E-52 | 194 | flagella-related protein C |
| TON_1185 | TK0042 | 69.57 | 5.00E-87 | 313 | flagellin |
| TON_1186 | TK0041 | 73.76 | 8.00E-72 | 262 | flagellin |
| TON_1187 | TK0040 | 61.63 | 9.00E-66 | 242 | flagellin |
| TON_1189 | TK0038 | 59.52 | 6.00E-67 | 246 | flagellin |
| TON_1190 | TK0037 | 55.41 | 2.00E-104 | 371 | SAM-dependent methyltransferase |
| TON_1191 | TK0036 | 85.32 | 2.00E-52 | 196 | hypothetical protein |
| TON_1193 | TK0629 | 82.88 | 1.00E-57 | 214 | purine-binding chemotaxis protein (cheW) |
| TON_1195 | TK0631 | 67.86 | 8.00E-112 | 395 | cheR chemotaxis protein methyltransferase |
| TON_1196 | TK0632 | 88.33 | 2.00E-59 | 219 | chemotaxis response regulator |
| TON_1197 | TK0633 | 75.42 | 1.00E-139 | 488 | chemotaxis-specific methylesterase |
| TON_1198 | TK0634 | 58.47 | 6.00E-149 | 520 | cheA chemotaxis histidine kinase |
| TON_1199 | TK0636 | 67.48 | 2.00E-79 | 287 | histidine kinase |
| TON_1200 | TK0637 | 65 | 5.00E-65 | 239 | chemotaxis protein cheC |
| TON_1201 | TK0639 | 78.26 | 1.00E-73 | 267 | chemoreceptor glutamine deamidase CheD |
| TON_1202 | TK0638 | 64.79 | 0 | 813 | methyl-accepting chemotaxis protein |
| TON_1203 | TK0640 | 50.39 | 1.00E-63 | 234 | hypothetical protein |
| TON_1204 | TK0641 | 61.29 | 5.00E-118 | 416 | hypothetical protein |
| TON_1205 | TK0035 | 50.63 | 2.00E-110 | 391 | membrane protein |
| TON_1206 | TK0034 | 68.29 | 6.00E-97 | 345 | hypothetical protein |
| TON_1207 | TK0033 | 48.05 | 2.00E-77 | 281 | hypothetical protein |
| TON_1208 | TK0032 | 39.28 | 9.00E-87 | 313 | hypothetical protein |

| | | | | | |
|---|---|---|---|---|---|
| TON_1211 | TK0030 | 77.01 | 1.00E-83 | 301 | cyclase-related protein |
| TON_1212 | TK0028 | 90.02 | 0 | 721 | S-adenosyl-L-homocysteine hydrolase |
| TON_1214 | TK0027 | 70.89 | 1.00E-82 | 298 | hypothetical protein |
| TON_1215 | TK0014 | 62.78 | 3.00E-61 | 226 | metal-dependent phosphohydrolase |
| TON_1216 | TK0013 | 69.61 | 1.00E-41 | 159 | hypothetical protein |
| TON_1217 | TK0012 | 90.27 | 1.00E-72 | 264 | GTP-binding protein |
| TON_1218 | TK0011 | 69.4 | 3.00E-55 | 206 | hypothetical protein |
| TON_1219 | TK0010 | 46.81 | 4.00E-18 | 81.6 | hypothetical protein |
| TON_1220 | TK0009 | 46.72 | 4.00E-108 | 384 | hypothetical protein |
| TON_1221 | TK0008 | 81.69 | 3.00E-171 | 593 | DNA methylase |
| TON_1223 | TK0004 | 44.37 | 5.00E-22 | 95.9 | membrane protein |
| TON_1224 | TK0003 | 46.96 | 4.00E-15 | 72.4 | Membrane protein |
| TON_1225 | TK0002 | 62.79 | 4.00E-75 | 273 | putative zinc-dependent protease |
| TON_1226 | TK2296 | 73.52 | 2.00E-110 | 390 | hypothetical protein |
| TON_1228 | TK1893 | 66.53 | 2.00E-86 | 312 | glycerate kinase |
| TON_1229 | TK2293 | 76.26 | 4.00E-59 | 219 | hypothetical protein |
| TON_1230 | TK2292 | 89.55 | 2.00E-31 | 126 | rps17E 30S ribosomal protein S17e |
| TON_1231 | TK2291 | 80.3 | 1.00E-81 | 295 | heat shock regulator |
| TON_1233 | TK1318 | 86.98 | 5.00E-98 | 348 | thymidine kinase |
| TON_1234 | TK2290 | 91.67 | 0 | 844 | rbcL ribulose bisophosphate carboxylase |
| TON_1236 | TK2288 | 69.51 | 4.00E-62 | 229 | metallophosphoesterase |
| TON_1237 | TK2287 | 88.12 | 2.00E-135 | 474 | tfb transcription initiation factor IIB |
| TON_1239 | TK2285 | 61.31 | 2.00E-82 | 298 | carbohydrate/pyrimidine kinase |
| TON_1240 | TK2284 | 69.23 | 2.00E-63 | 233 | ADP-ribose pyrophosphatase |
| TON_1241 | TK2283 | 68.97 | 3.00E-39 | 152 | hypothetical protein |
| TON_1242 | TK2282 | 36.53 | 2.00E-78 | 285 | membrane protein |
| TON_1243 | TK2281 | 71.71 | 2.00E-106 | 377 | ABC transporter |
| TON_1244 | TK0298 | 77.8 | 0 | 689 | putative transposase |
| TON_1245 | TK0849 | 92.16 | 1.00E-21 | 93.2 | hypothetical protein |
| TON_1247 | TK2280 | 88.84 | 5.00E-120 | 422 | ATP-binding protein |
| TON_1248 | TK2279 | 67.61 | 2.00E-161 | 560 | bifunctional sugar nucleotidyltransferase/CDP-alcohol phosphatidyltransferase synthase |
| TON_1249 | TK2278 | 87.43 | 0 | 703 | myo-inositol-1-phosphate synthase |
| TON_1250 | TK2277 | 68 | 4.00E-78 | 283 | hypothetical protein |
| TON_1251 | TK2276 | 81.04 | 2.00E-101 | 360 | orotidine 5'-phosphate decarboxylase |
| TON_1252 | TK2275 | 83.72 | 2.00E-82 | 296 | putative RNA-binding protein |
| TON_1253 | TK2274 | 84.93 | 2.00E-72 | 263 | glycerol-3-phosphate cytidylyltransferase |
| TON_1254 | TK2273 | 75.44 | 6.00E-41 | 157 | putative transcriptional regulator |
| TON_1255 | TK2272 | 88.67 | 2.00E-127 | 447 | ATPase |
| TON_1256 | TK2271 | 79.43 | 2.00E-173 | 600 | cell division protein FtsZ |
| TON_1257 | TK2270 | 87.3 | 3.00E-28 | 115 | hypothetical protein |
| TON_1258 | TK2269 | 56.3 | 2.00E-35 | 139 | hypothetical protein |
| TON_1259 | TK2268 | 86.99 | 0 | 719 | aspartate aminotransferase |
| TON_1260 | TK2267 | 75.86 | 1.00E-34 | 136 | membrane protein |
| TON_1261 | TK2266 | 76.63 | 7.00E-178 | 615 | permease |
| TON_1263 | TK2263 | 92.47 | 1.00E-131 | 461 | putative transcriptional regulator |
| TON_1264 | TK2260 | 88 | 5.00E-144 | 502 | dihydroorotate dehydrogenase 1B |
| TON_1265 | TK0119 | 83.16 | 0 | 759 | proline dehydrogenase subunit alpha |
| TON_1266 | TK0120 | 84.34 | 1.00E-80 | 290 | proline dehydrogenase subunit gamma |

| | | | | | |
|---|---|---|---|---|---|
| TON_1267 | TK0121 | 75.9 | 4.00E-31 | 124 | hypothetical protein |
| TON_1268 | TK0122 | 56.35 | 3.00E-111 | 394 | sarcosine oxidase subunit beta |
| TON_1270 | TK0305 | 94.67 | 2.00E-115 | 406 | pyrH uridylate kinase |
| TON_1271 | TK0304 | 91.57 | 0 | 768 | NADH oxidase |
| TON_1278 | TK0302 | 83.89 | 2.00E-87 | 313 | RNA 2'-phosphotransferase-like protein |
| TON_1279 | TK0123 | 68.2 | 2.00E-130 | 457 | multidrug-efflux transporter |
| TON_1280 | TK1831 | 58.62 | 3.00E-113 | 400 | galactokinase |
| TON_1281 | TK0117 | 90.16 | 0 | 688 | sarcosine oxidase, beta subunit |
| TON_1282 | TK0116 | 75.41 | 0 | 709 | sarcosine oxidase, alpha subunit |
| TON_1283 | TK1286 | 95.1 | 0 | 753 | GTPase |
| TON_1284 | TK1285 | 82.55 | 7.00E-61 | 224 | transcriptional regulator |
| TON_1285 | TK1284 | 92.12 | 2.00E-88 | 316 | intracellular protease I |
| TON_1289 | TK0153 | 66.55 | 1.00E-116 | 411 | ATP:dephospho-CoA triphosphoribosyl transferase |
| TON_1290 | TK0154 | 75 | 2.00E-24 | 102 | hypothetical protein |
| TON_1291 | TK1659 | 80.75 | 1.00E-79 | 287 | small neutral amino acid transporter A |
| TON_1292 | TK0189 | 82.44 | 5.00E-60 | 221 | stress-inducible protein |
| TON_1293 | TK0188 | 84.59 | 1.00E-142 | 497 | hypothetical protein |
| TON_1973 | TK0187 | 57.69 | 1.00E-86 | 311 | glutamine amidotransferase |
| TON_1295 | TK0186 | 86.57 | 0 | 729 | multiple substrate aminotransferase |
| TON_1296 | TK0185 | 94.1 | 0 | 627 | translation initiation factor IF-2 |
| TON_1297 | TK1749 | 27.46 | 2.00E-13 | 68.2 | hypothetical protein |
| TON_1298 | TK1750 | 36.77 | 2.00E-54 | 205 | hypothetical protein |
| TON_1299 | TK0184 | 90.05 | 3.00E-175 | 607 | C/D box methylation guide ribonucleoprotein complex aNOP56 subunit |
| TON_1300 | TK0183 | 93.81 | 1.00E-123 | 434 | fibrillarin |
| TON_1301 | TK0181 | 88.86 | 1.00E-179 | 621 | hypothetical protein |
| TON_1302 | TK0180 | 85.05 | 4.00E-179 | 619 | acetyl-CoA acetyltransferase |
| TON_1303 | TK0179 | 83.58 | 3.00E-65 | 239 | hypothetical protein |
| TON_1304 | TK0124 | 75.26 | 2.00E-37 | 145 | hypothetical protein |
| TON_1305 | TK0125 | 81.76 | 4.00E-60 | 223 | N-acetyltransferase |
| TON_1306 | TK0126 | 79.53 | 7.00E-65 | 238 | transcription factor |
| TON_1307 | TK0129 | 84.48 | 4.00E-58 | 215 | hypothetical protein |
| TON_1309 | TK0132 | 91.67 | 2.00E-99 | 353 | transcription factor |
| TON_1310 | TK0134 | 63.94 | 1.00E-129 | 454 | acetylpolyamine aminohydrolase |
| TON_1311 | TK0135 | 87.62 | 2.00E-104 | 370 | indolepyruvate oxidoreductase subunit beta |
| TON_1312 | TK0136 | 89.03 | 0 | 1031 | indolepyruvate: ferredoxin oxidoreductase subunit alpha |
| TON_1313 | TK0139 | 86.24 | 0 | 792 | acetyl-CoA synthetase II subunit alpha |
| TON_1314 | TK0141 | 81.25 | 7.00E-102 | 362 | metal-dependent hydrolase |
| TON_1315 | TK0142 | 64.97 | 2.00E-118 | 417 | putative transcriptional regulator |
| TON_1316 | TK0143 | 80.85 | 3.00E-19 | 85.5 | nucleic acid-binding protein |
| TON_1317 | TK0144 | 63.33 | 2.00E-87 | 314 | hypothetical protein |
| TON_1318 | TK0145 | 44.39 | 2.00E-37 | 147 | membrane protein |
| TON_1320 | TK1323 | 72.97 | 3.00E-164 | 570 | glycosyltransferase |
| TON_1321 | TK1324 | 53.91 | 5.00E-65 | 239 | 4-hydroxybenzoate octaprenyltransferase |
| TON_1322 | TK0147 | 80.07 | 2.00E-136 | 477 | spermidine synthase |
| TON_1323 | TK0149 | 91.72 | 4.00E-83 | 299 | pyruvoyl-dependent arginine decarboxylase |
| TON_1324 | TK0627 | 62.7 | 2.00E-118 | 418 | NapA-type sodium/hydrogen antiporter |
| TON_1325 | TK0626 | 75.21 | 1.00E-46 | 177 | hypothetical protein |

| | | | | | |
|---|---|---|---|---|---|
| TON_1326 | TK0625 | 55.2 | 2.00E-120 | 425 | putative monovalent cation/H+ antiporter subunit D |
| TON_1327 | TK0624 | 72.29 | 2.00E-22 | 95.9 | putative monovalent cation/H+ antiporter subunit C |
| TON_1328 | TK0623 | 60 | 7.00E-41 | 158 | putative monovalent cation/H+ antiporter subunit B |
| TON_1331 | TK0620 | 74.04 | 4.00E-37 | 145 | putative monovalent cation/H+ antiporter subunit G |
| TON_1332 | TK0619 | 63.33 | 7.00E-25 | 103 | putative monovalent cation/H+ antiporter subunit F |
| TON_1333 | TK0618 | 67.16 | 8.00E-80 | 288 | putative monovalent cation/H+ antiporter subunit E |
| TON_1334 | TK0150 | 91.79 | 2.00E-67 | 246 | glycine cleavage system protein H |
| TON_1336 | TK1325 | 90.55 | 0 | 856 | putative oxidoreductase |
| TON_1337 | TK1326 | 69.23 | 2.00E-120 | 424 | ferredoxin-NADP(+) reductase subunit alpha |
| TON_1339 | TK1328 | 81.42 | 6.00E-113 | 399 | tRNA (1-methyladenosine) methyltransferase |
| TON_1340 | TK1329 | 43.92 | 9.00E-35 | 139 | hypothetical protein |
| TON_1341 | TK1330 | 63.44 | 7.00E-32 | 127 | signal recognition particle protein Srp19 |
| TON_1342 | TK1331 | 92.5 | 6.00E-38 | 147 | putative transcriptional regulator |
| TON_1344 | TK1334 | 82.61 | 9.00E-87 | 311 | hypothetical protein |
| TON_1345 | TK0652 | 77.7 | 6.00E-106 | 375 | heavy-metal cation transporter |
| TON_1346 | TK1335 | 96.3 | 2.00E-73 | 266 | hypothetical protein |
| TON_1347 | TK1336 | 66.67 | 7.00E-153 | 532 | ABC-type sodium efflux pump system permease |
| TON_1348 | TK1337 | 82.14 | 3.00E-109 | 386 | ABC-type sodium efflux pump system, ATPase component |
| TON_1349 | TK1339 | 92.31 | 1.00E-29 | 119 | putative transcriptional regulator |
| TON_1353 | TK1064 | 64.89 | 1.00E-148 | 518 | cmo tungsten-containing aldehyde ferredoxin oxidoreductase cofactor modifying protein |
| TON_1354 | TK1066 | 79.17 | 0 | 949 | aor-2 tungsten-containing aldehyde ferredoxin oxidoreductase |
| TON_1355 | TK1689 | 76.17 | 0 | 939 | subtilisin-like serine protease |
| TON_1356 | TK1690 | 58.71 | 0 | 726 | membrane protein |
| TON_1358 | TK1691 | 86.32 | 0 | 633 | hypothetical protein |
| TON_1359 | TK1692 | 70.33 | 8.00E-96 | 342 | hypothetical protein |
| TON_1360 | TK1693 | 74.84 | 4.00E-69 | 252 | hypothetical protein |
| TON_1361 | TK1694 | 73.13 | 1.00E-21 | 93.2 | 4Fe-4S ferredoxin |
| TON_1362 | TK1676 | 88.21 | 0 | 676 | nicotinate phosphoribosyltransferase |
| TON_1363 | TK1681 | 83.61 | 1.00E-150 | 525 | hypothetical protein |
| TON_1365 | TK2254 | 67.72 | 4.00E-97 | 347 | UDP-N-acetylglucosamine--dolichyl-phosphate N-acetylglucosaminephosphotransferase |
| TON_1366 | TK2255 | 73.41 | 0 | 649 | bifunctional phosphatase/dolichol-phosphate glucosyltransferase |
| TON_1367 | TK2256 | 48.76 | 8.00E-27 | 111 | hypothetical protein |
| TON_1368 | TK2257 | 92.12 | 5.00E-89 | 318 | deoxycytidylate deaminase |
| TON_1369 | TK2258 | 59.39 | 5.00E-73 | 266 | zinc-dependent protease |
| TON_1370 | TK2259 | 87.6 | 2.00E-60 | 223 | putative transcriptional regulator |
| TON_1372 | TK0106 | 88.64 | 2.00E-140 | 490 | diphthine synthase |
| TON_1373 | TK1688 | 86.01 | 2.00E-120 | 423 | putative transcriptional regulator |
| TON_1374 | TK1686 | 90.8 | 5.00E-139 | 485 | hypothetical protein |
| TON_1375 | TK1685 | 83.1 | 9.00E-137 | 478 | ferredoxin-NADP(+) reductase subunit alpha |
| TON_1376 | TK1684 | 86.75 | 0 | 780 | putative oxidoreductase |
| TON_1377 | TK1683 | 55.77 | 1.00E-40 | 157 | hypothetical protein |
| TON_1385 | TK2252 | 88.44 | 0 | 681 | proteasome-activating nucleotidase |
| TON_1386 | TK2251 | 80.56 | 2.00E-148 | 517 | permease |
| TON_1388 | TK2250 | 87.42 | 2.00E-162 | 563 | serine/threonine protein kinase |
| TON_1389 | TK2249 | 61.11 | 2.00E-54 | 203 | hypothetical protein |

| | | | | | |
|---|---|---|---|---|---|
| TON_1390 | TK2248 | 63.06 | 3.00E-103 | 367 | biotin synthase |
| TON_1391 | TK2247 | 52.73 | 8.00E-106 | 376 | hypothetical protein |
| TON_1392 | TK2246 | 93.46 | 2.00E-160 | 556 | L-asparaginase |
| TON_1393 | TK2245 | 85.53 | 7.00E-151 | 525 | transcriptional regulator |
| TON_1394 | TK0827 | 78.92 | 2.00E-169 | 587 | permease |
| TON_1395 | TK2244 | 86.53 | 3.00E-97 | 346 | indolepyruvate oxidoreductase subunit B |
| TON_1396 | TK2241 | 75.88 | 1.00E-101 | 361 | SAM-dependent methyltransferase |
| TON_1397 | TK2240 | 86.88 | 0 | 904 | lysK lysyl-tRNA synthetase |
| TON_1398 | TK2239 | 72.76 | 1.00E-114 | 404 | PhoI-related type II restriction endonuclease |
| TON_1399 | TK2238 | 75.71 | 7.00E-74 | 268 | membrane protein |
| TON_1400 | TK2237 | 84.57 | 2.00E-92 | 330 | hypothetical protein |
| TON_1403 | TK2235 | 78.49 | 3.00E-132 | 463 | ribose-phosphate pyrophosphokinase |
| TON_1404 | TK2234 | 70.35 | 6.00E-70 | 256 | membrane protein |
| TON_1405 | TK2233 | 76.92 | 6.00E-52 | 194 | hypothetical protein |
| TON_1406 | TK2232 | 79.72 | 2.00E-98 | 350 | hydrolase |
| TON_1407 | TK2231 | 81.28 | 2.00E-103 | 367 | radB DNA repair and recombination protein RadB |
| TON_1408 | TK2227 | 84.29 | 0 | 800 | RNA-binding protein FAU-1 |
| TON_1409 | TK2226 | 60.89 | 3.00E-77 | 280 | hypothetical protein |
| TON_1410 | TK2225 | 83.71 | 1.00E-151 | 528 | moaA molybdenum cofactor biosynthesis protein A |
| TON_1412 | TK2223 | 84.82 | 6.00E-143 | 499 | Met-10+ like protein |
| TON_1413 | TK2219 | 90.05 | 0 | 748 | replication factor C large subunit |
| TON_1415 | TK2217 | 96.71 | 0 | 734 | 2-amino-3-ketobutyrate coenzyme A ligase |
| TON_1417 | TK2215 | 76.05 | 7.00E-73 | 265 | tRNA-splicing endonuclease subunit alpha |
| TON_1418 | TK2214 | 84.24 | 4.00E-80 | 289 | ribosomal protein-alanine acetyltransferase |
| TON_1420 | TK2213 | 68.79 | 0 | 761 | bipolar DNA helicase |
| TON_1421 | TK2212 | 74.94 | 1.00E-174 | 605 | DNA repair exonuclease |
| TON_1423 | TK2210 | 82.43 | 0 | 674 | 5'-3' nuclease |
| TON_1424 | TK2209 | 68.15 | 1.00E-126 | 444 | ABC-type iron(III)-siderophore transport system permease |
| TON_1425 | TK2208 | 72.88 | 7.00E-101 | 358 | ABC-type iron(III)-siderophore transport system, ATPase component |
| TON_1426 | TK2207 | 86.17 | 3.00E-97 | 346 | proteasome subunit beta 2 |
| TON_1427 | TK2206 | 63.34 | 6.00E-130 | 456 | hypothetical protein |
| TON_1428 | TK2205 | 81.67 | 1.00E-164 | 571 | threonine synthase |
| TON_1430 | TK2203 | 67.82 | 9.00E-91 | 325 | hypothetical protein |
| TON_1432 | TK2200 | 92.41 | 0 | 827 | hypothetical protein |
| TON_1434 | TK2198 | 85.59 | 8.00E-59 | 218 | hypothetical protein |
| TON_1436 | TK2197 | 57.09 | 5.00E-89 | 319 | putative transcriptional regulator |
| TON_1437 | TK2196 | 91.64 | 1.00E-154 | 538 | pyrB aspartate carbamoyltransferase catalytic subunit |
| TON_1438 | TK2195 | 77.03 | 6.00E-65 | 238 | aspartate carbamoyltransferase regulatory subunit |
| TON_1440 | TK2194 | 60.82 | 7.00E-64 | 235 | membrane protein |
| TON_1441 | TK2193 | 76.05 | 3.00E-114 | 403 | hypothetical protein |
| TON_1442 | TK2192 | 81.08 | 7.00E-89 | 318 | hypothetical protein |
| TON_1444 | TK2191 | 77.49 | 2.00E-85 | 307 | hypothetical protein |
| TON_1445 | TK2185 | 87.31 | 0 | 738 | glmM phosphoglucosamine mutase |
| TON_1446 | TK2183 | 60.47 | 3.00E-37 | 145 | hypothetical protein |
| TON_1449 | TK2179 | 85.71 | 4.00E-124 | 436 | hypothetical protein |
| TON_1450 | TK2173 | 77.27 | 2.00E-68 | 250 | hypothetical protein |
| TON_1451 | TK2172 | 70.14 | 0 | 932 | cyclomaltodextrin glucanotransferase |

| | | | | | |
|---|---|---|---|---|---|
| TON_1452 | TK2171 | 51.21 | 8.00E-55 | 205 | hypothetical protein |
| TON_1453 | TK2170 | 62.88 | 2.00E-153 | 534 | zinc-dependent protease |
| TON_1454 | TK2169 | 73.51 | 0 | 702 | zinc-dependent protease |
| TON_1457 | TK0881 | 73.15 | 2.00E-61 | 226 | universal stress protein |
| TON_1458 | TK0880 | 68.88 | 1.00E-145 | 508 | arsenical pump membrane protein |
| TON_1459 | TK0879 | 46.96 | 1.00E-38 | 151 | hypothetical protein |
| TON_1460 | TK2168 | 69.41 | 0 | 810 | serine protease |
| TON_1461 | TK2167 | 78.68 | 0 | 652 | hypothetical protein |
| TON_1462 | TK2166 | 74.11 | 2.00E-157 | 547 | ArgE/DapE-related deacylase |
| TON_1463 | TK1966 | 89.11 | 2.00E-155 | 540 | D-3-phosphoglycerate dehydrogenase |
| TON_1464 | TK1967 | 74.42 | 8.00E-85 | 305 | phosphate transport system regulator PhoU |
| TON_1465 | TK1968 | 82.61 | 1.00E-145 | 507 | 2-dehydropantoate 2-reductase |
| TON_1466 | TK1970 | 73.71 | 4.00E-100 | 356 | putative tRNA/rRNA methyltransferase |
| TON_1467 | TK1971 | 61.71 | 2.00E-59 | 220 | methylated-DNA--protein-cysteine methyltransferase |
| TON_1468 | TK1972 | 83.72 | 2.00E-94 | 338 | membrane protein |
| TON_1470 | TK1560 | 53.46 | 6.00E-75 | 273 | NapA-type sodium/hydrogen antiporter |
| TON_1472 | TK1973 | 74.77 | 2.00E-37 | 145 | nac nascent polypeptide-associated complex protein |
| TON_1473 | TK1974 | 88.03 | 5.00E-55 | 204 | carboxymuconolactone decarboxylase-related protein |
| TON_1474 | TK1976 | 80 | 1.00E-168 | 585 | HflX-related GTP-binding protein |
| TON_1475 | TK1977 | 84.38 | 2.00E-125 | 441 | sodium/phosphate symporter |
| TON_1476 | TK1978 | 92.97 | 9.00E-74 | 268 | pyruvate/ketoisovalerate ferredoxin oxidoreductase subunit gamma |
| TON_1477 | TK1979 | 86.27 | 3.00E-49 | 185 | vorD 2-ketoisovalerate ferredoxin oxidoreductase subunit delta |
| TON_1478 | TK1980 | 91.16 | 0 | 684 | vorA 2-ketoisovalerate ferredoxin oxidoreductase subunit alpha |
| TON_1479 | TK1981 | 89.07 | 1.00E-170 | 591 | 2-ketoisovalerate ferredoxin oxidoreductase subunit beta |
| TON_1480 | TK1982 | 92.38 | 1.00E-55 | 206 | porD pyuvate ferredoxin oxidoreductase subunit delta |
| TON_1481 | TK1983 | 94.92 | 0 | 718 | porA pyruvate ferredoxin oxidoreductase subunit alpha |
| TON_1482 | TK1984 | 90.03 | 1.00E-153 | 535 | pyruvate ferredoxin oxidoreductase subunit beta |
| TON_1483 | TK2021 | 90.81 | 4.00E-143 | 499 | ATPase |
| TON_1484 | TK2022 | 35.42 | 9.00E-64 | 236 | membrane protein |
| TON_1485 | TK2023 | 83.61 | 7.00E-112 | 395 | hypothetical protein |
| TON_1486 | TK2024 | 85.33 | 6.00E-65 | 238 | transcription initiation factor E subunit alpha |
| TON_1487 | TK2025 | 90.43 | 3.00E-46 | 175 | hypothetical protein |
| TON_1488 | TK2029 | 71.03 | 4.00E-121 | 426 | carbohydrate/pyrimidine kinase |
| TON_1490 | TK2031 | 91.21 | 4.00E-44 | 167 | acylphosphatase |
| TON_1491 | TK2032 | 74.51 | 1.00E-37 | 146 | divalent cation tolerance protein |
| TON_1492 | TK2033 | 79.9 | 3.00E-86 | 309 | hypothetical protein |
| TON_1493 | TK2034 | 70.14 | 9.00E-113 | 398 | universal stress protein |
| TON_1494 | TK2035 | 93.72 | 0 | 771 | gcvT glycine cleavage system aminomethyltransferase T |
| TON_1495 | TK2036 | 71.64 | 6.00E-108 | 382 | permease |
| TON_1496 | TK2165 | 84.62 | 2.00E-34 | 135 | hypothetical protein |
| TON_1497 | TK2164 | 90.67 | 0 | 690 | thermophile-specific fructose-1,6-bisphosphatase |
| TON_1498 | TK2163 | 86.83 | 0 | 1155 | tungsten-containing glyceraldehyde-3-phosphate:ferredoxin oxidoreductase |

| | | | | | |
|---|---|---|---|---|---|
| TON_1501 | TK2159 | 80.95 | 0 | 691 | oxidoreductase |
| TON_1502 | TK2158 | 90.76 | 8.00E-168 | 582 | carbamate kinase-like carbamoyl phosphate synthetase |
| TON_1503 | TK0130 | 85.56 | 1.00E-131 | 461 | hypothetical protein |
| TON_1504 | TK2157 | 92.62 | 5.00E-123 | 432 | hypothetical protein |
| TON_1505 | TK2156 | 88.07 | 0 | 1019 | RNA-binding protein |
| TON_1507 | TK2153 | 87.5 | 3.00E-45 | 172 | hypothetical protein |
| TON_1508 | TK2152 | 89.57 | 8.00E-107 | 378 | hypothetical protein |
| TON_1509 | TK0729 | 57.79 | 5.00E-68 | 250 | ubiE ubiquinone/menaquinone biosynthesis methyltransferase |
| TON_1510 | TK2134 | 85.11 | 2.00E-69 | 253 | transcriptional regulator |
| TON_1511 | TK2135 | 76.57 | 0 | 675 | 23S rRNA (uracil-5-)-methyltransferase |
| TON_1512 | TK2136 | 84.51 | 8.00E-31 | 124 | hypothetical protein |
| TON_1513 | TK2138 | 76.57 | 4.00E-68 | 249 | pyrE orotate phosphoribosyltransferase |
| TON_1515 | TK2140 | 88.26 | 0 | 932 | ATP-dependent DNA ligase |
| TON_1516 | TK2141 | 78.67 | 7.00E-135 | 472 | GHMP kinase |
| TON_1517 | TK2142 | 83.88 | 0 | 906 | NhaC-type sodium/hydrogen antiporter |
| TON_1518 | TK2143 | 86.32 | 3.00E-98 | 349 | uracil-DNA glycosylase |
| TON_1519 | TK2144 | 67.24 | 2.00E-45 | 173 | hypothetical protein |
| TON_1520 | TK2145 | 87.76 | 0 | 1076 | hypothetical protein |
| TON_1521 | TK2146 | 60.44 | 4.00E-19 | 85.5 | hypothetical protein |
| TON_1522 | TK2148 | 76.39 | 0 | 741 | hypothetical protein |
| TON_1523 | TK2149 | 65.18 | 2.00E-31 | 126 | effector of murein hydrolase |
| TON_1524 | TK2150 | 75.78 | 2.00E-87 | 313 | effector of murein hydrolase |
| TON_1525 | TK2151 | 84.62 | 7.00E-69 | 251 | tfx putative transcriptional regulator |
| TON_1526 | TK2133 | 56.48 | 9.00E-56 | 208 | hypothetical protein |
| TON_1527 | TK2132 | 76.5 | 3.00E-82 | 296 | L-fuculose phosphate aldolase |
| TON_1528 | TK2131 | 96.39 | 7.00E-41 | 157 | hypothetical protein |
| TON_1529 | TK2130 | 86.29 | 1.00E-87 | 313 | cob(I)yrinic acid a,c-diamide adenosyltransferase |
| TON_1530 | TK1905 | 74.28 | 1.00E-123 | 434 | hypothetical protein |
| TON_1531 | TK1907 | 88.78 | 2.00E-88 | 317 | hypothetical protein |
| TON_1532 | TK1908 | 87.55 | 6.00E-129 | 452 | lipoate-protein ligase A, N-terminal section |
| TON_1533 | TK2037 | 65.45 | 1.00E-55 | 207 | signal peptidase I |
| TON_1534 | TK2038 | 78.57 | 8.00E-75 | 271 | hypothetical protein |
| TON_1535 | TK2039 | 45.2 | 2.00E-84 | 305 | hypothetical protein |
| TON_1536 | TK2040 | 76.13 | 4.00E-143 | 499 | microsomal dipeptidase |
| TON_1539 | TK2045 | 78.72 | 5.00E-125 | 439 | SAM-dependent methyltransferase |
| TON_1540 | TK2046 | 80.26 | 0 | 678 | membrane protein |
| TON_1541 | TK2047 | 85.91 | 0 | 766 | membrane protein |
| TON_1542 | TK2048 | 62.8 | 0 | 1189 | hypothetical protein |
| TON_1543 | TK2049 | 82.77 | 0 | 1034 | acylamino acid-releasing protein |
| TON_1544 | TK2050 | 65.74 | 2.00E-35 | 139 | membrane protein |
| TON_1545 | TK2051 | 64.68 | 5.00E-74 | 269 | hypothetical protein |
| TON_1546 | TK2052 | 96 | 2.00E-36 | 142 | regulatory protein AsnC |
| TON_1550 | TK2059 | 81.44 | 2.00E-43 | 166 | hypothetical protein |
| TON_1551 | TK2060 | 83.93 | 5.00E-96 | 342 | phosphate transport system regulator PhoU |
| TON_1552 | TK2061 | 83.74 | 2.00E-180 | 624 | sodium/phosphate symporter |
| TON_1553 | TK2062 | 52.6 | 1.00E-155 | 542 | metallophosphoesterase |
| TON_1554 | TK2063 | 83.05 | 2.00E-57 | 212 | hypothetical protein |

| | | | | | |
|---|---|---|---|---|---|
| TON_1557 | TK2064 | 86.7 | 0 | 770 | 2-methylthioadenine synthetase |
| TON_1558 | TK2065 | 87.32 | 6.00E-17 | 77.8 | RNA-binding protein |
| TON_1563 | TK2076 | 83.4 | 0 | 1232 | formate dehydrogenase subunit alpha |
| TON_1564 | TK2077 | 86.14 | 1.00E-73 | 268 | 4Fe-4S binding protein |
| TON_1570 | TK2078 | 53.09 | 1.00E-40 | 157 | Formate hydrogen lyase subunit 6 (hydrogenase 3 component F) |
| TON_1573 | TK2079 | 85.67 | 6.00E-142 | 495 | formate transporter |
| TON_1582 | TK2080 | 76.19 | 2.00E-65 | 240 | putative monovalent cation/H+ antiporter subunit E |
| TON_1583 | TK2081 | 80.25 | 2.00E-32 | 129 | putative monovalent cation/H+ antiporter subunit F |
| TON_1584 | TK2082 | 80.49 | 2.00E-54 | 202 | putative monovalent cation/H+ antiporter subunit G |
| TON_1585 | TK2083 | 84.88 | 1.00E-11 | 60.1 | hypothetical protein |
| TON_1586 | TK2084 | 89.8 | 1.00E-38 | 149 | membrane bound hydrogenase subunit MbhE |
| TON_1587 | TK2085 | 87.92 | 4.00E-55 | 205 | putative monovalent cation/H+ antiporter subunit B |
| TON_1588 | TK2086 | 89.92 | 4.00E-52 | 195 | putative monovalent cation/H+ antiporter subunit C |
| TON_1589 | TK2087 | 80.08 | 0 | 749 | putative monovalent cation/H+ antiporter subunit D |
| TON_1590 | TK2088 | 80 | 7.00E-54 | 201 | membrane bound hydrogenase subunit MbhI |
| TON_1591 | TK2089 | 86.63 | 2.00E-87 | 313 | membrane bound hydrogenase, NiFe-hydrogenase small subunit |
| TON_1592 | TK2090 | 71.96 | 9.00E-79 | 285 | membrane bound hydrogenase, NiFe-hydrogenase large subunit 1 |
| TON_1593 | TK2091 | 85.68 | 0 | 771 | membrane bound hydrogenase, NiFe-hydrogenase large subunit 2 |
| TON_1594 | TK2092 | 83.54 | 4.00E-134 | 469 | membrane bound hydrogenase, MbhM subunit |
| TON_1596 | TK2096 | 63.69 | 2.00E-46 | 177 | rubrerythrin-like protein |
| TON_1597 | TK0841 | 56.36 | 3.00E-14 | 68.9 | membrane protein |
| TON_1598 | TK0840 | 71.18 | 3.00E-91 | 327 | NAD(P)H-flavin oxidoreductase |
| TON_1599 | TK2097 | 72.96 | 1.00E-56 | 211 | RNA-binding protein |
| TON_1600 | TK2098 | 85.53 | 0 | 704 | ATPase |
| TON_1601 | TK2099 | 58.86 | 1.00E-51 | 194 | membrane-bound metal-dependent hydrolase |
| TON_1603 | TK2100 | 90 | 2.00E-159 | 554 | thioredoxin reductase |
| TON_1605 | TK2101 | 84.27 | 0 | 782 | 4-aminobutyrate aminotransferase |
| TON_1606 | TK2102 | 65.83 | 3.00E-59 | 220 | membrane protein |
| TON_1607 | TK2103 | 60.64 | 2.00E-31 | 125 | hypothetical protein |
| TON_1608 | TK2104 | 85.84 | 7.00E-106 | 375 | deoxyribose-phosphate aldolase |
| TON_1609 | TK2105 | 65.98 | 3.00E-35 | 139 | hypothetical protein |
| TON_1611 | TK0235 | 65.16 | 2.00E-126 | 444 | permease |
| TON_1613 | TK2106 | 91.86 | 0 | 764 | eno phosphopyruvate hydratase |
| TON_1614 | TK2109 | 83.94 | 3.00E-139 | 486 | biotin synthase |
| TON_1615 | TK2110 | 87.33 | 2.00E-75 | 273 | transcriptional regulator |
| TON_1616 | TK2111 | 78.26 | 2.00E-84 | 303 | putative deoxyribonucleotide triphosphate pyrophosphatase |
| TON_1617 | TK2112 | 92.55 | 4.00E-84 | 302 | hypothetical protein |
| TON_1618 | TK2113 | 58.25 | 6.00E-92 | 329 | hypothetical protein |
| TON_1619 | TK2114 | 59.15 | 2.00E-106 | 377 | hypothetical protein |
| TON_1620 | TK2115 | 75.54 | 3.00E-50 | 189 | molybdopterin converting factor subunit 2 |
| TON_1622 | TK2117 | 80.6 | 3.00E-89 | 320 | hypothetical protein |
| TON_1623 | TK2118 | 82.95 | 2.00E-39 | 152 | molybdopterin converting factor subunit 1 |
| TON_1624 | TK2119 | 85.71 | 4.00E-63 | 232 | hypothetical protein |
| TON_1625 | TK0169 | 63.58 | 3.00E-44 | 169 | putative transcriptional regulator |
| TON_1628 | TK2120 | 37.32 | 7.00E-51 | 193 | hypothetical protein |

| | | | | | |
|---|---|---|---|---|---|
| TON_1629 | TK2121 | 71.93 | 5.00E-69 | 252 | hypothetical protein |
| TON_1630 | TK2122 | 90.34 | 0 | 711 | tRNA/rRNA cytosine-C5-methylase |
| TON_1631 | TK2123 | 55.49 | 8.00E-91 | 326 | membrane protein |
| TON_1632 | TK2124 | 84.17 | 6.00E-139 | 485 | ppnK inorganic polyphosphate/ATP-NAD kinase |
| TON_1633 | TK2125 | 34.75 | 2.00E-36 | 144 | membrane protein |
| TON_1634 | TK2126 | 92.62 | 7.00E-162 | 561 | putative DNA-binding/iron metalloprotein/AP endonuclease |
| TON_1635 | TK1674 | 66.67 | 3.00E-62 | 229 | hypothetical protein |
| TON_1636 | TK2127 | 86.99 | 0 | 753 | acyl-CoA synthetase large subunit |
| TON_1638 | TK2128 | 83.33 | 9.00E-74 | 268 | phosphopantetheine adenylyltransferase |
| TON_1640 | TK2129 | 93.36 | 4.00E-111 | 392 | triosephosphate isomerase |
| TON_1641 | TK1904 | 85.96 | 6.00E-120 | 422 | hypothetical protein |
| TON_1642 | TK1903 | 86.59 | 0 | 2995 | DNA polymerase II large subunit |
| TON_1643 | TK1902 | 64.45 | 0 | 904 | DNA polymerase II small subunit |
| TON_1644 | TK1901 | 89.64 | 0 | 728 | cdc6 cell division control protein 6 |
| TON_1645 | TK0133 | 57.51 | 9.00E-63 | 231 | hypothetical protein |
| TON_1646 | TK1900 | 67.08 | 2.00E-98 | 351 | calcium-gated potassium channel protein |
| TON_1648 | TK1898 | 77.11 | 3.00E-103 | 366 | hypothetical protein |
| TON_1649 | TK1897 | 63.78 | 2.00E-44 | 169 | hypothetical protein |
| TON_1650 | TK1896 | 83.06 | 8.00E-119 | 418 | hypothetical protein |
| TON_1651 | TK1895 | 95.33 | 1.00E-142 | 498 | 5'-methylthioadenosine phosphorylase |
| TON_1653 | TK1891 | 84.25 | 0 | 742 | N-ethylammeline chlorohydrolase |
| TON_1654 | TK0195 | 30.14 | 3.00E-24 | 105 | hypothetical protein |
| TON_1655 | TK1890 | 83.89 | 1.00E-74 | 271 | hypothetical protein |
| TON_1659 | TK1886 | 84.58 | 0 | 768 | putative YjeF-ralted carbohydrate kinase |
| TON_1660 | TK1884 | 82.43 | 0 | 778 | alpha-amylase |
| TON_1661 | TK1883 | 69.73 | 5.00E-74 | 268 | transcriptional regulator |
| TON_1662 | TK1882 | 92.08 | 2.00E-49 | 186 | hypothetical protein |
| TON_1663 | TK1881 | 92.15 | 9.00E-100 | 354 | transcriptional regulator |
| TON_1665 | TK1880 | 90.62 | 0 | 849 | succinyl-CoA synthetase large subunit |
| TON_1666 | TK1874 | 88.77 | 0 | 655 | tryptophanyl-tRNA synthetase |
| TON_1667 | TK1872 | 86.22 | 3.00E-116 | 410 | 2-hydroxyhepta-2,4-diene-1,7-dioate isomerase |
| TON_1671 | TK1863 | 83.29 | 0 | 627 | N6-adenine-specific DNA methylase |
| TON_1672 | TK1857 | 85.99 | 3.00E-160 | 556 | GTPase |
| TON_1674 | TK1854 | 34.15 | 1.00E-42 | 166 | hypothetical protein |
| TON_1676 | TK1852 | 68.57 | 3.00E-140 | 490 | hypothetical protein |
| TON_1677 | TK1851 | 66.67 | 4.00E-112 | 396 | hypothetical protein |
| TON_1678 | TK1850 | 71.7 | 6.00E-65 | 238 | FKBP-type peptidyl-prolyl cis-trans isomerase |
| TON_1680 | TK1849 | 74.39 | 7.00E-73 | 265 | hypothetical protein |
| TON_1683 | TK1846 | 81.39 | 1.00E-154 | 537 | hypothetical protein |
| TON_1684 | TK1845 | 64.75 | 2.00E-144 | 504 | hypothetical protein |
| TON_1686 | TK1843 | 74.37 | 6.00E-95 | 339 | carbohydrate/pyrimidine kinase |
| TON_1687 | TK1840 | 92.99 | 0 | 934 | carboxypeptidase |
| TON_1688 | TK1837 | 85.22 | 2.00E-53 | 199 | hypothetical protein |
| TON_1689 | TK1836 | 74.39 | 5.00E-157 | 546 | geranylgeranyl hydrogenase |
| TON_1690 | TK1835 | 83.59 | 1.00E-95 | 341 | pyrrolidone-carboxylate peptidase |
| TON_1691 | TK1833 | 84.4 | 0 | 931 | hypothetical protein |
| TON_1692 | TK1821 | 93.5 | 0 | 633 | ATPase |
| TON_1693 | TK1824 | 53.47 | 8.00E-70 | 255 | hypothetical protein |

| | | | | | |
|---|---|---|---|---|---|
| TON_1694 | TK1820 | 66.67 | 3.00E-144 | 503 | membrane-associated metalloprotease |
| TON_1695 | TK1717 | 77.21 | 6.00E-134 | 469 | glycosyltransferase |
| TON_1702 | TK1819 | 39.48 | 5.00E-90 | 324 | polysaccharide biosynthesis-like protein |
| TON_1709 | TK1817 | 80.42 | 1.00E-126 | 444 | cobalt/zinc/cadmium cation efflux pump protein |
| TON_1710 | TK1814 | 84.6 | 2.00E-172 | 597 | L-tyrosine decarboxylase |
| TON_1711 | TK1813 | 80.26 | 9.00E-163 | 565 | NapA-type sodium/hydrogen antiporter |
| TON_1713 | TK1811 | 62.07 | 2.00E-78 | 284 | hydrolase |
| TON_1714 | TK1810 | 63.24 | 6.00E-62 | 229 | hypothetical protein |
| TON_1716 | TK1809 | 86.2 | 0 | 1170 | 4-alpha-Glucanotransferase |
| TON_1717 | TK1556 | 86.84 | 2.00E-154 | 537 | ATPase |
| TON_1718 | TK1557 | 91.67 | 0 | 874 | dehydrogenase |
| TON_1719 | TK1558 | 68.51 | 2.00E-60 | 223 | hypothetical protein |
| TON_1720 | TK1559 | 66.84 | 5.00E-149 | 519 | NapA-type sodium/hydrogen antiporter |
| TON_1721 | TK1561 | 74.13 | 1.00E-61 | 228 | hypothetical protein |
| TON_1722 | TK1562 | 80.79 | 0 | 694 | hisS histidyl-tRNA synthetase |
| TON_1729 | TK1567 | 91.17 | 0 | 1620 | alaS alanyl-tRNA synthetase |
| TON_1730 | TK1570 | 66.41 | 4.00E-44 | 169 | hypothetical protein |
| TON_1731 | TK1571 | 66.67 | 4.00E-51 | 192 | hypothetical protein |
| TON_1732 | TK1572 | 75.4 | 2.00E-104 | 370 | NGG1p interacting factor 3 |
| TON_1733 | TK1574 | 92.74 | 0 | 1084 | histone acetyltransferase Elp3 |
| TON_1736 | TK1580 | 74.19 | 3.00E-67 | 246 | hypothetical protein |
| TON_1737 | TK1581 | 87.57 | 4.00E-87 | 312 | hypothetical protein |
| TON_1740 | TK1590 | 93.95 | 2.00E-137 | 480 | hypothetical protein |
| TON_1742 | TK1592 | 89.13 | 1.00E-71 | 260 | S-adenosylmethionine decarboxylase |
| TON_1743 | TK1593 | 73.24 | 3.00E-116 | 410 | preprotein translocase subunit SecF |
| TON_1744 | TK1594 | 70.81 | 0 | 714 | secD preprotein translocase subunit SecD |
| TON_1745 | TK1595 | 85.71 | 2.00E-109 | 387 | Trk-type pottasium transport system, NAD-binding component |
| TON_1747 | TK1597 | 66.72 | 0 | 815 | V-type ATP synthase subunit I |
| TON_1748 | TK1598 | 85.19 | 2.00E-35 | 140 | V-type ATP synthase subunit K |
| TON_1749 | TK1599 | 73.4 | 5.00E-62 | 229 | V-type ATP synthase subunit E |
| TON_1750 | TK1600 | 67.59 | 8.00E-144 | 502 | V-type ATP synthase subunit C |
| TON_1751 | TK1601 | 78.43 | 4.00E-44 | 168 | V-type ATP synthase subunit F |
| TON_1752 | TK1602 | 95.38 | 0 | 1080 | V-type ATP synthase subunit A |
| TON_1753 | TK1603 | 98.27 | 0 | 922 | V-type ATP synthase subunit B |
| TON_1754 | TK1604 | 90.57 | 1.00E-105 | 374 | V-type ATP synthase subunit D |
| TON_1755 | TK1605 | 83.92 | 2.00E-132 | 463 | flavoprotein |
| TON_1756 | TK1606 | 84.15 | 1.00E-106 | 378 | Methyl-accepting chemotaxis protein |
| TON_1757 | TK1607 | 60.93 | 5.00E-134 | 469 | hypothetical protein |
| TON_1758 | TK1551 | 78.47 | 2.00E-128 | 451 | ATPase |
| TON_1761 | TK1807 | 88 | 8.00E-162 | 561 | hypothetical protein |
| TON_1762 | TK1806 | 73.39 | 2.00E-104 | 370 | dihydroorotate dehydrogenase electron transfer subunit |
| TON_1763 | TK1805 | 69.9 | 3.00E-167 | 580 | dihydroorotase |
| TON_1765 | TK1803 | 55.4 | 5.00E-102 | 363 | oligopeptide transport system permease protein appB |
| TON_1766 | TK1802 | 53.18 | 6.00E-130 | 456 | dipeptide/oligopeptide ABC transporter permease |
| TON_1767 | TK1801 | 89.97 | 3.00E-173 | 600 | dipeptide/oligopeptide ABC transporter ATPase |
| TON_1768 | TK1800 | 91.64 | 1.00E-178 | 617 | dipeptide/oligopeptide ABC transporter ATPase |
| TON_1770 | TK1798 | 77.56 | 1.00E-102 | 364 | NAD synthetase |

| | | | | | |
|---|---|---|---|---|---|
| TON_1771 | TK1797 | 79.64 | 3.00E-121 | 426 | permease |
| TON_1772 | TK1796 | 78.33 | 0 | 732 | glnA glutamine synthetase |
| TON_1773 | TK1795 | 63.7 | 1.00E-46 | 177 | hypothetical protein |
| TON_1775 | TK1793 | 32.2 | 2.00E-50 | 192 | membrane protein |
| TON_1776 | TK1792 | 65.48 | 2.00E-89 | 321 | permease |
| TON_1777 | TK1791 | 75.87 | 1.00E-159 | 555 | DNA primase small subunit |
| TON_1778 | TK1790 | 63.59 | 8.00E-142 | 495 | DNA primase large subunit |
| TON_1779 | TK1789 | 85.54 | 7.00E-112 | 395 | ATPase |
| TON_1780 | TK1788 | 51 | 5.00E-101 | 360 | hypothetical protein |
| TON_1781 | TK1787 | 55.35 | 3.00E-100 | 357 | membrane protein |
| TON_1782 | TK1786 | 75 | 1.00E-36 | 143 | hypothetical protein |
| TON_1783 | TK1785 | 93.94 | 0 | 711 | putative tRNA/rRNA methyltransferase |
| TON_1784 | TK1784 | 80.82 | 6.00E-111 | 392 | hypothetical protein |
| TON_1785 | TK1781 | 85.07 | 0 | 734 | diaminopimelate aminotransferase |
| TON_1786 | TK1780 | 79.82 | 8.00E-46 | 174 | hypothetical protein |
| TON_1787 | TK1779 | 86.31 | 0 | 893 | oligopeptide transporter |
| TON_1788 | TK1778 | 84.87 | 1.00E-142 | 497 | hydrolase |
| TON_1789 | TK1777 | 75.72 | 0 | 711 | phosphopentomutase |
| TON_1790 | TK1776 | 79.23 | 1.00E-58 | 217 | hypothetical protein |
| TON_1791 | TK1775 | 88.27 | 0 | 678 | ABC-type maltdextrin transport system, ATPase component |
| TON_1792 | TK1774 | 80.93 | 0 | 1741 | pullulanase |
| TON_1793 | TK1773 | 75.9 | 2.00E-148 | 517 | ABC-type maltodextrin transport system permease |
| TON_1794 | TK1772 | 83.39 | 1.00E-144 | 504 | sugar transport inner membrane protein (malf-like) |
| TON_1795 | TK1771 | 80.72 | 0 | 681 | ABC-type maltodextrin transport system |
| TON_1796 | TK1770 | 62.1 | 0 | 754 | cyclomaltodextrinase |
| TON_1797 | TK1769 | 84.04 | 5.00E-167 | 579 | putative transcriptional regulator |
| TON_1798 | TK1768 | 76.89 | 0 | 655 | glycogen synthase |
| TON_1799 | TK1767 | 84.17 | 7.00E-58 | 215 | ribonuclease P protein component 2 |
| TON_1800 | TK1766 | 83.33 | 6.00E-170 | 588 | hypothetical protein |
| TON_1803 | TK1748 | 90.61 | 0 | 1968 | ileS isoleucyl-tRNA synthetase |
| TON_1804 | TK1746 | 90.27 | 4.00E-36 | 142 | hypothetical protein |
| TON_1805 | TK1745 | 71.64 | 2.00E-112 | 397 | permease |
| TON_1810 | TK1744 | 81.37 | 4.00E-45 | 171 | hypothetical protein |
| TON_1811 | TK1743 | 80.7 | 0 | 967 | hypothetical protein |
| TON_1812 | TK1741 | 75.06 | 0 | 647 | tRNA CCA-pyrophosphorylase |
| TON_1813 | TK1738 | 92.93 | 2.00E-88 | 317 | 2'-5' RNA ligase |
| TON_1815 | TK1737 | 87.01 | 7.00E-76 | 275 | xanthine/guanine phosphoribosyltransferase |
| TON_1817 | TK1735 | 71.53 | 4.00E-46 | 175 | hypothetical protein |
| TON_1818 | TK1734 | 75.1 | 4.00E-111 | 393 | sugar phosphatase |
| TON_1820 | TK1718 | 48.83 | 0 | 669 | oligosaccharyl transferase |
| TON_1821 | TK1733 | 47.73 | 6.00E-100 | 356 | lps biosynthesis rfbU related protein |
| TON_1822 | TK1732 | 65.85 | 2.00E-133 | 467 | dolichol-phosphate mannosyltransferase |
| TON_1823 | TK1731 | 45.58 | 3.00E-71 | 261 | glycosyltransferase |
| TON_1827 | TK1818 | 54.34 | 2.00E-50 | 190 | hypothetical protein |
| TON_1836 | TK1708 | 84.71 | 3.00E-147 | 513 | galE-2 UDP-glucose 4-epimerase |
| TON_1837 | TK1711 | 80.66 | 1.00E-159 | 554 | sugar-phosphate nucleotydyltransferase |
| TON_1850 | TK1118 | 39.16 | 6.00E-19 | 85.9 | nucleic acid-binding protein |

| | | | | | |
|---|---|---|---|---|---|
| TON_1852 | TK1712 | 27.49 | 2.00E-33 | 135 | lipopolysaccharide O-side chain biosynthesis protein |
| TON_1858 | TK1721 | 28.96 | 6.00E-31 | 127 | phosphatidylinositol glycantransferase-class A |
| TON_1861 | TK1722 | 30.17 | 3.00E-12 | 65.1 | glycosyltransferase |
| TON_1865 | TK0111 | 93.22 | 1.00E-28 | 116 | hypothetical protein |
| TON_1866 | TK0112 | 95.6 | 3.00E-46 | 175 | ef1B elongation factor 1-beta |
| TON_1869 | TK2297 | 77.42 | 1.00E-111 | 395 | hypothetical protein |
| TON_1870 | TK2298 | 81.51 | 0 | 1060 | anaerobic ribonucleoside triphosphate reductase |
| TON_1871 | TK2299 | 76.37 | 1.00E-104 | 371 | anaerobic ribonucleoside-triphosphate reductase activating enzyme |
| TON_1877 | TK2303 | 95.99 | 0 | 962 | chaperonin beta subunit |
| TON_1878 | TK1298 | 90.93 | 2.00E-177 | 613 | hypothetical protein |
| TON_1881 | TK0175 | 83.98 | 5.00E-89 | 319 | hypothetical protein |
| TON_1882 | TK0178 | 82.28 | 0 | 1063 | DNA helicase |
| TON_1884 | TK0194 | 85.6 | 0 | 851 | inosine 5'-monophosphate dehydrogenase |
| TON_1885 | TK0297 | 75.28 | 0 | 694 | L-aspartate oxidase |
| TON_1887 | TK0296 | 86.09 | 1.00E-155 | 541 | quinolinate synthetase |
| TON_1888 | TK1314 | 39.45 | 2.00E-85 | 308 | hypothetical protein |
| TON_1889 | TK0218 | 85.51 | 7.00E-130 | 455 | nicotinate-nucleotide pyrophosphorylase |
| TON_1890 | TK0217 | 89.97 | 2.00E-162 | 563 | pyridoxal biosynthesis lyase PdxS |
| TON_1891 | TK0216 | 78.17 | 2.00E-88 | 317 | glutamine amidotransferase subunit PdxT |
| TON_1892 | TK0209 | 64.15 | 6.00E-11 | 58.2 | hypothetical protein |
| TON_1893 | TK0193 | 92.83 | 7.00E-167 | 578 | GMP synthase |
| TON_1894 | TK0190 | 92.02 | 1.00E-103 | 367 | GMP synthase |
| TON_1895 | TK1661 | 46.85 | 2.00E-57 | 214 | hypothetical protein |
| TON_1900 | TK1666 | 46.9 | 4.00E-34 | 135 | hypothetical protein |
| TON_1901 | TK1667 | 52.75 | 2.00E-47 | 181 | membrane protein |
| TON_1902 | TK1668 | 63.59 | 7.00E-53 | 199 | hypothetical protein |
| TON_1903 | TK1669 | 89.69 | 2.00E-174 | 604 | cell division GTPase |
| TON_1904 | TK1670 | 31.59 | 1.00E-88 | 320 | hypothetical protein |
| TON_1905 | TK1671 | 87.04 | 8.00E-170 | 588 | tRNA-modifying protein |
| TON_1906 | TK1673 | 81.05 | 1.00E-88 | 317 | N5-glutamine methyltransferase |
| TON_1908 | TK1675 | 77.72 | 2.00E-162 | 564 | subtilisin-like serine protease |
| TON_1909 | TK1695 | 91.67 | 2.00E-22 | 95.9 | 30S ribosomal protein S27ae |
| TON_1910 | TK1696 | 85.71 | 7.00E-47 | 177 | rps24e 30S ribosomal protein S24e |
| TON_1911 | TK1697 | 71.75 | 2.00E-61 | 227 | hypothetical protein |
| TON_1912 | TK1698 | 75.76 | 3.00E-23 | 99 | DNA-directed RNA polymerase subunit E'' |
| TON_1913 | TK1699 | 83.63 | 2.00E-79 | 286 | DNA-directed RNA polymerase subunit E' |
| TON_1914 | TK1700 | 97.19 | 1.00E-91 | 327 | inorganic pyrophosphatase |
| TON_1918 | TK1909 | 76.42 | 2.00E-157 | 547 | dolichol-phosphate mannosyltransferase |
| TON_1919 | TK1912 | 58.78 | 9.00E-177 | 612 | putative leucine aminopeptidase |
| TON_1920 | TK1913 | 74.53 | 1.00E-41 | 160 | transcriptional regulator |
| TON_1921 | TK1914 | 65.15 | 2.00E-76 | 277 | exonuclease SbcD |
| TON_1923 | TK1916 | 85.61 | 2.00E-135 | 473 | carbon-nitrogen hydrolase |
| TON_1925 | TK1917 | 61.07 | 1.00E-79 | 288 | SAM-dependent methyltransferase |
| TON_1927 | TK1920 | 65.12 | 2.00E-47 | 179 | hypothetical protein |
| TON_1930 | TK1923 | 57.09 | 1.00E-68 | 252 | membrane protein |
| TON_1931 | TK1924 | 67.8 | 4.00E-25 | 105 | transcriptional regulator |
| TON_1932 | TK1925 | 59.19 | 6.00E-64 | 236 | membrane protein |

| | | | | | |
|---|---|---|---|---|---|
| TON_1933 | TK1926 | 85.32 | 2.00E-50 | 189 | hypothetical protein |
| TON_1934 | TK1928 | 81.71 | 3.00E-165 | 573 | diphthine synthase, DPH2 subunit |
| TON_1935 | TK1933 | 79.7 | 3.00E-94 | 336 | DNA methylase |
| TON_1936 | TK1934 | 31.32 | 2.00E-17 | 82 | hypothetical protein |
| TON_1938 | TK1935 | 85 | 0 | 708 | tRNA/rRNA cytosine-C5-methylase |
| TON_1939 | TK1940 | 63.07 | 2.00E-97 | 347 | small-conductance mechanosensitive channel |
| TON_1940 | TK1941 | 53.99 | 1.00E-64 | 238 | hypothetical protein |
| TON_1941 | TK1943 | 66.67 | 6.00E-76 | 275 | hypothetical protein |
| TON_1942 | TK1944 | 83.72 | 1.00E-119 | 421 | metal-dependent phosphohydrolase |
| TON_1943 | TK1945 | 87.41 | 1.00E-66 | 243 | nucleic acid-binding protein |
| TON_1944 | TK1946 | 96.1 | 0 | 749 | translation initiation factor IF-2 |
| TON_1945 | TK1951 | 87.2 | 9.00E-61 | 224 | 30S ribosomal protein S6e |
| TON_1946 | TK1952 | 80.36 | 6.00E-15 | 71.2 | preprotein translocase subunit SecG |
| TON_1947 | TK1953 | 78.24 | 6.00E-80 | 289 | hypothetical protein |
| TON_1948 | TK1954 | 79.12 | 2.00E-81 | 294 | GTP-binding protein |
| TON_1976 | TK1955 | 84.42 | 4.00E-71 | 258 | putative transcriptional regulator |
| TON_1949 | TK1956 | 58.82 | 2.00E-109 | 388 | hypothetical protein |
| TON_1950 | TK1957 | 76.53 | 5.00E-122 | 429 | ubiA prenyltransferase UbiA-like protein |
| TON_1951 | TK1958 | 78.57 | 9.00E-57 | 210 | hypothetical protein |
| TON_1952 | TK1959 | 86.96 | 3.00E-101 | 360 | replication factor A complex, RPA32 subunit |
| TON_1953 | TK1960 | 83.87 | 6.00E-50 | 187 | replication factor A complex, RPA14 subunit |
| TON_1954 | TK1961 | 72.44 | 2.00E-137 | 481 | replication factor A |
| TON_1955 | TK1962 | 88.76 | 8.00E-91 | 325 | putative transcriptional regulator |
| TON_1956 | TK0107 | 84.44 | 1.00E-66 | 244 | manganese-dependent transcription regulator |
| TON_1957 | TK0157 | 81.66 | 0 | 640 | xanthine/uracilpermease |
| TON_1958 | TK0158 | 74.17 | 3.00E-121 | 427 | sugar-binding transport ATP-binding protein |
| TON_1959 | TK0159 | 74.23 | 0 | 803 | iron(III) ABC transporter permease |
| TON_1960 | TK0160 | 75.51 | 3.00E-141 | 493 | D-aminopeptidase |
| TON_1961 | TK0170 | 87.14 | 9.00E-138 | 481 | endonuclease IV |
| TON_1962 | TK0171 | 40.12 | 2.00E-43 | 168 | hypothetical protein |
| TON_1963 | TK0172 | 71.43 | 2.00E-152 | 530 | membrane protein |
| TON_1964 | TK0173 | 84 | 4.00E-36 | 141 | hypothetical protein |
| TON_1965 | TK0174 | 88.78 | 2.00E-105 | 373 | hypothetical protein |
| TON_1966 | TK2304 | 92.89 | 0 | 878 | tRNA/rRNA cytosine-C5-methylase |

**TON_nc050 was classified to non-coding RNA by reversed annotation of TON_1978 in NCBI database.

**Supplementary Table 8.** The 5' UTR length of orthologous genes between *T. onnurineus* and *T. kodakarensis*

| orthologous genes | | UTR length (nt) | |
|---|---|---|---|
| TON | KOD | TON | KOD |
| TON_0002 | TK0110 | 9 | 10 |
| TON_0004 | TK0108 | -99 | 89 |
| TON_0006 | TK1992 | 0 | 1 |
| TON_0007 | TK0155 | 29 | 11 |
| TON_0008 | TK1658 | 25 | 11 |
| TON_0012 | TK1652 | 10 | 259 |
| TON_0017 | TK1649 | 8 | 9 |
| TON_0018 | TK1648 | 12 | 11 |
| TON_0020 | TK1643 | -5 | 10 |
| TON_0022 | TK1642 | 10 | 12 |
| TON_0023 | TK1641 | 9 | 7 |
| TON_0027 | TK1637 | 45 | 28 |
| TON_0033 | TK1631 | 32 | 28 |
| TON_0035 | TK1629 | 9 | 9 |
| TON_0037 | TK1626 | 16 | 12 |
| TON_0041 | TK1622 | 30 | 22 |
| TON_0042 | TK1621 | 12 | 11 |
| TON_0044 | TK1619 | 16 | 11 |
| TON_0045 | TK1618 | 9 | 13 |
| TON_0047 | TK1616 | 9 | 9 |
| TON_0058 | TK1550 | 11 | 10 |
| TON_0059 | TK1549 | 11 | 9 |
| TON_0061 | TK1548 | 12 | 12 |
| TON_0063 | TK1546 | 80 | 33 |
| TON_0064 | TK1545 | 9 | 9 |
| TON_0090 | TK1517 | 23 | 33 |
| TON_0092 | TK1515 | 12 | 11 |
| TON_0096 | TK1509 | 24 | 117 |
| TON_0113 | TK1495 | 25 | 26 |
| TON_0114 | TK1494 | 27 | 23 |
| TON_0115 | TK1493 | 10 | 8 |
| TON_0117 | TK1492 | 24 | 26 |
| TON_0118 | TK1491 | 10 | 10 |
| TON_0120 | TK1489 | 15 | 16 |
| TON_0123 | TK1486 | 11 | 12 |
| TON_0127 | TK1483 | 10 | 12 |
| TON_0131 | TK1479 | 22 | 23 |
| TON_0135 | TK1470 | 10 | 10 |
| TON_0136 | TK1469 | 49 | 50 |
| TON_0141 | TK1461 | 126 | 128 |
| TON_0143 | TK1455 | 10 | 9 |
| TON_0144 | TK1454 | 69 | 69 |
| TON_0145 | TK1451 | 11 | 13 |
| TON_0147 | TK1442 | 247 | 265 |

| | | | |
|---|---|---|---|
| TON_0149 | TK1440 | 0 | 13 |
| TON_0150 | TK1439 | 9 | 8 |
| TON_0154 | TK1435 | 9 | 8 |
| TON_0156 | TK1432 | 59 | 59 |
| TON_0157 | TK1431 | 29 | 26 |
| TON_0163 | TK1430 | 13 | 13 |
| TON_0164 | TK1429 | 46 | 43 |
| TON_0167 | TK1427 | 11 | 12 |
| TON_0169 | TK1425 | 9 | 9 |
| TON_0170 | TK1424 | 11 | 10 |
| TON_0174 | TK0935 | 52 | 33 |
| TON_0175 | TK1422 | 11 | 9 |
| TON_0176 | TK1421 | 52 | 75 |
| TON_0180 | TK1417 | 42 | 52 |
| TON_0185 | TK1413 | 27 | 4 |
| TON_0186 | TK1412 | 0 | 0 |
| TON_0187 | TK1411 | 9 | 9 |
| TON_0189 | TK1408 | 10 | 37 |
| TON_0190 | TK1407 | 22 | 23 |
| TON_0191 | TK1406 | 27 | 27 |
| TON_0192 | TK1405 | 12 | 11 |
| TON_0195 | TK1404 | 0 | 73 |
| TON_0196 | TK1403 | 28 | 23 |
| TON_0198 | TK1400 | 21 | 48 |
| TON_0200 | TK1398 | 10 | 9 |
| TON_0203 | TK1393 | 18 | 17 |
| TON_0208 | TK1387 | 13 | 33 |
| TON_0209 | TK1386 | 0 | 0 |
| TON_0210 | TK1385 | 11 | 11 |
| TON_0213 | TK1380 | 30 | 30 |
| TON_0227 | TK1067 | 1 | 1 |
| TON_0228 | TK1093 | 10 | 11 |
| TON_0231 | TK1094 | 10 | 10 |
| TON_0234 | TK0825 | 23 | 63 |
| TON_0235 | TK1098 | 81 | 79 |
| TON_0237 | TK1100 | 28 | 25 |
| TON_0240 | TK1103 | 35 | 32 |
| TON_0245 | TK1109 | 24 | 9 |
| TON_0249 | TK1112 | 11 | 10 |
| TON_0252 | TK1114 | 0 | 0 |
| TON_0258 | TK2016 | 9 | 59 |
| TON_0260 | TK2009 | 9 | 12 |
| TON_0261 | TK2008 | 9 | 9 |
| TON_0288 | TK1991 | 9 | 10 |
| TON_0305 | TK1299 | 10 | 10 |
| TON_0306 | TK1297 | 11 | 10 |
| TON_0309 | TK1294 | 9 | 8 |
| TON_0310 | TK1293 | 45 | 46 |

| | | | |
|---|---|---|---|
| TON_0311 | TK1292 | 27 | 26 |
| TON_0312 | TK1289 | 7 | 8 |
| TON_0313 | TK1288 | 8 | 12 |
| TON_0314 | TK1287 | 9 | 10 |
| TON_0318 | TK1086 | 82 | 83 |
| TON_0319 | TK1085 | 75 | 73 |
| TON_0327 | TK0465 | 23 | 23 |
| TON_0330 | TK0469 | 12 | 4 |
| TON_0332 | TK0471 | 144 | 143 |
| TON_0336 | TK0475 | 23 | 23 |
| TON_0342 | TK0481 | 41 | 33 |
| TON_0344 | TK0493 | 28 | 32 |
| TON_0348 | TK0494 | 9 | 9 |
| TON_0349 | TK1195 | 12 | 12 |
| TON_0352 | TK1193 | 19 | 21 |
| TON_0353 | TK1192 | 10 | 9 |
| TON_0361 | TK1186 | 16 | 18 |
| TON_0362 | TK1183 | 11 | 118 |
| TON_0363 | TK1182 | 9 | 10 |
| TON_0369 | TK1177 | 25 | 24 |
| TON_0371 | TK1175 | 11 | 10 |
| TON_0372 | TK1174 | 10 | 10 |
| TON_0374 | TK1172 | 11 | 31 |
| TON_0377 | TK1169 | 11 | 11 |
| TON_0381 | TK1166 | 10 | 23 |
| TON_0382 | TK1165 | 10 | 11 |
| TON_0383 | TK1164 | 10 | 8 |
| TON_0390 | TK0941 | 47 | 26 |
| TON_0392 | TK0929 | 10 | 6 |
| TON_0393 | TK0928 | 9 | 14 |
| TON_0394 | TK0927 | 0 | 0 |
| TON_0396 | TK0921 | 14 | 14 |
| TON_0397 | TK0916 | 24 | 23 |
| TON_0403 | TK0908 | 12 | 10 |
| TON_0404 | TK0904 | -83 | 0 |
| TON_0405 | TK0903 | 44 | 44 |
| TON_0406 | TK0902 | 59 | 129 |
| TON_0408 | TK0900 | 12 | 12 |
| TON_0410 | TK2095 | 10 | 97 |
| TON_0411 | TK0897 | 0 | 0 |
| TON_0414 | TK0894 | 28 | 107 |
| TON_0415 | TK0893 | 11 | 10 |
| TON_0421 | TK0887 | 5 | 31 |
| TON_0422 | TK0885 | 10 | 9 |
| TON_0427 | TK0877 | 46 | 42 |
| TON_0429 | TK0875 | 9 | 9 |
| TON_0435 | TK0871 | 10 | 10 |
| TON_0437 | TK0870 | 10 | 8 |

| | | | |
|---|---|---|---|
| TON_0440 | TK0869 | -24 | 11 |
| TON_0442 | TK0866 | 10 | 10 |
| TON_0447 | TK0648 | 14 | 104 |
| TON_0453 | TK0617 | 7 | 3 |
| TON_0455 | TK0615 | 36 | 35 |
| TON_0456 | TK1196 | 10 | 10 |
| TON_0466 | TK1204 | 8 | 9 |
| TON_0469 | TK1207 | 10 | 11 |
| TON_0471 | TK1209 | 0 | 0 |
| TON_0484 | TK1212 | 8 | 8 |
| TON_0498 | TK1226 | 54 | 40 |
| TON_0499 | TK1227 | 25 | 25 |
| TON_0502 | TK1231 | 10 | 289 |
| TON_0503 | TK1232 | 11 | 12 |
| TON_0504 | TK1234 | 10 | 10 |
| TON_0507 | TK1239 | 31 | 25 |
| TON_0509 | TK1241 | 4 | 11 |
| TON_0511 | TK1246 | 40 | 40 |
| TON_0512 | TK1247 | 14 | 11 |
| TON_0514 | TK1249 | 9 | 9 |
| TON_0517 | TK1251 | 46 | 46 |
| TON_0523 | TK1259 | 22 | 20 |
| TON_0524 | TK1260 | 44 | 219 |
| TON_0525 | TK1261 | 11 | 9 |
| TON_0528 | TK1263 | 10 | 9 |
| TON_0529 | TK1264 | 24 | 22 |
| TON_0537 | TK2072 | 53 | 52 |
| TON_0544 | TK1569 | 23 | 77 |
| TON_0546 | TK0865 | 24 | 24 |
| TON_0547 | TK0569 | 50 | 9 |
| TON_0549 | TK1267 | 0 | 0 |
| TON_0553 | TK1268 | 57 | 58 |
| TON_0554 | TK1269 | 68 | 67 |
| TON_0555 | TK1270 | 10 | 9 |
| TON_0560 | TK0545 | 25 | 32 |
| TON_0565 | TK0548 | 10 | 10 |
| TON_0568 | TK0550 | 9 | 10 |
| TON_0569 | TK0551 | 11 | 11 |
| TON_0573 | TK1053 | 0 | 112 |
| TON_0579 | TK1049 | 10 | 10 |
| TON_0581 | TK1120 | 0 | 0 |
| TON_0588 | TK1131 | 21 | 23 |
| TON_0589 | TK1135 | 11 | 9 |
| TON_0590 | TK1137 | 0 | 0 |
| TON_0593 | TK0656 | 7 | 25 |
| TON_0600 | TK0663 | 22 | 86 |
| TON_0601 | TK0664 | 12 | 12 |
| TON_0602 | TK0444 | 16 | 10 |

| | | | |
|---|---|---|---|
| TON_0606 | TK0436 | 11 | 10 |
| TON_0610 | TK0424 | 0 | 0 |
| TON_0611 | TK0423 | 10 | 11 |
| TON_0612 | TK1048 | 11 | 13 |
| TON_0613 | TK0764 | 1 | 1 |
| TON_0614 | TK0763 | 10 | 9 |
| TON_0615 | TK0762 | 0 | 0 |
| TON_0619 | TK0379 | 1 | 1 |
| TON_0620 | TK0378 | 30 | 27 |
| TON_0628 | TK0774 | 0 | 0 |
| TON_0639 | TK0765 | 9 | 10 |
| TON_0649 | TK0969 | 15 | 39 |
| TON_0650 | TK0968 | 0 | 11 |
| TON_0651 | TK0967 | 10 | 10 |
| TON_0654 | TK0960 | 0 | 0 |
| TON_0655 | TK0959 | 26 | 37 |
| TON_0659 | TK0955 | 9 | 10 |
| TON_0660 | TK0510 | 11 | 11 |
| TON_0661 | TK0509 | 10 | 10 |
| TON_0662 | TK0508 | 40 | 39 |
| TON_0670 | TK0503 | 230 | 18 |
| TON_0671 | TK0502 | 24 | 24 |
| TON_0673 | TK0497 | 10 | 1 |
| TON_0675 | TK0422 | -38 | 7 |
| TON_0677 | TK1274 | 31 | 29 |
| TON_0681 | TK0862 | 9 | 113 |
| TON_0688 | TK0853 | -44 | 9 |
| TON_0689 | TK0846 | 0 | 0 |
| TON_0690 | TK0544 | 10 | 10 |
| TON_0698 | TK0538 | 11 | 11 |
| TON_0701 | TK0671 | 0 | 0 |
| TON_0703 | TK0673 | 0 | 27 |
| TON_0705 | TK0677 | 29 | 72 |
| TON_0707 | TK0678 | 30 | 209 |
| TON_0714 | TK0358 | 11 | 2 |
| TON_0718 | TK0363 | 11 | 31 |
| TON_0720 | TK1876 | 0 | 0 |
| TON_0739 | TK1035 | 10 | 13 |
| TON_0743 | TK1039 | 9 | 9 |
| TON_0744 | TK1040 | 39 | 31 |
| TON_0745 | TK0029 | 8 | 10 |
| TON_0746 | TK0069 | 17 | 109 |
| TON_0747 | TK0070 | 29 | 33 |
| TON_0748 | TK0071 | 1 | 1 |
| TON_0752 | TK0308 | 26 | 118 |
| TON_0755 | TK0309 | 32 | 32 |
| TON_0756 | TK0312 | 17 | 23 |
| TON_0757 | TK0793 | 21 | 10 |

| | | | |
|---|---|---|---|
| TON_0767 | TK0802 | 21 | 10 |
| TON_0771 | TK0806 | 9 | 63 |
| TON_0772 | TK0807 | 0 | 0 |
| TON_0773 | TK0808 | 10 | 7 |
| TON_0779 | TK1065 | 10 | 12 |
| TON_0780 | TK0812 | 9 | 14 |
| TON_0785 | TK0511 | 11 | 10 |
| TON_0786 | TK1055 | 9 | 10 |
| TON_0790 | TK0512 | 11 | 10 |
| TON_0797 | TK0516 | 44 | 30 |
| TON_0798 | TK0517 | 21 | 10 |
| TON_0799 | TK1063 | 95 | 10 |
| TON_0804 | TK0699 | 22 | 22 |
| TON_0818 | TK1074 | 132 | 8 |
| TON_0819 | TK0685 | 0 | 0 |
| TON_0820 | TK0684 | 13 | 107 |
| TON_0821 | TK0528 | 12 | 12 |
| TON_0824 | TK0533 | 23 | 21 |
| TON_0829 | TK0537 | 34 | 35 |
| TON_0845 | TK0005 | 11 | 8 |
| TON_0847 | TK0831 | 14 | 180 |
| TON_0866 | TK0523 | 23 | 24 |
| TON_0867 | TK0524 | 12 | 11 |
| TON_0871 | TK0650 | 44 | 34 |
| TON_0873 | TK1056 | 9 | 8 |
| TON_0877 | TK0826 | -59 | 11 |
| TON_0878 | TK0242 | 199 | 148 |
| TON_0891 | TK0981 | 0 | 0 |
| TON_0900 | TK0985 | 26 | 9 |
| TON_0901 | TK0986 | 27 | 37 |
| TON_0902 | TK0987 | -279 | 9 |
| TON_0903 | TK0989 | 72 | 10 |
| TON_0904 | TK0990 | 35 | 11 |
| TON_0905 | TK0991 | 24 | 25 |
| TON_0911 | TK1002 | 8 | 7 |
| TON_0912 | TK1004 | 12 | 10 |
| TON_0917 | TK0723 | 10 | 11 |
| TON_0924 | TK0742 | 10 | 10 |
| TON_0926 | TK0564 | 10 | 12 |
| TON_0927 | TK0563 | 10 | 9 |
| TON_0928 | TK0562 | 7 | 9 |
| TON_0930 | TK0560 | 25 | 25 |
| TON_0931 | TK0559 | 11 | 10 |
| TON_0932 | TK0558 | 14 | 12 |
| TON_0937 | TK1015 | 0 | 0 |
| TON_0938 | TK0313 | 10 | 0 |
| TON_0939 | TK0314 | 90 | 94 |
| TON_0943 | TK0319 | 0 | 0 |

| | | | |
|---|---|---|---|
| TON_0951 | TK1307 | 9 | 9 |
| TON_0955 | TK1313 | 10 | 10 |
| TON_0961 | TK0759 | 137 | 132 |
| TON_0964 | TK0867 | 10 | 35 |
| TON_0966 | TK0755 | 48 | 27 |
| TON_0970 | TK0749 | 0 | 0 |
| TON_0974 | TK0746 | 1 | 1 |
| TON_0976 | TK0744 | 7 | 1 |
| TON_0978 | TK0743 | 10 | 10 |
| TON_0981 | TK0568 | 11 | 64 |
| TON_0983 | TK0570 | 26 | 12 |
| TON_0984 | TK0571 | 39 | 39 |
| TON_0987 | TK0574 | 10 | 9 |
| TON_0989 | TK1147 | 31 | 49 |
| TON_0990 | TK1146 | 9 | 9 |
| TON_0994 | TK1143 | 10 | 13 |
| TON_0998 | TK1457 | 12 | 12 |
| TON_1001 | TK0944 | 29 | 29 |
| TON_1004 | TK1687 | 93 | 82 |
| TON_1006 | TK0366 | 0 | 0 |
| TON_1010 | TK0370 | 10 | 71 |
| TON_1011 | TK0375 | 11 | 10 |
| TON_1012 | TK0376 | 23 | 23 |
| TON_1032 | TK0781 | 28 | 26 |
| TON_1040 | TK0784 | -75 | 0 |
| TON_1046 | TK0788 | 9 | 9 |
| TON_1047 | TK0789 | 23 | 10 |
| TON_1048 | TK0790 | 158 | 169 |
| TON_1051 | TK0336 | 10 | 10 |
| TON_1055 | TK0345 | 1 | 1 |
| TON_1056 | TK0346 | 0 | 0 |
| TON_1057 | TK0347 | 11 | 1 |
| TON_1062 | TK0352 | 26 | 18 |
| TON_1063 | TK1030 | 11 | 13 |
| TON_1064 | TK1029 | 0 | 0 |
| TON_1066 | TK1023 | 10 | 10 |
| TON_1069 | TK1021 | 0 | 0 |
| TON_1070 | TK1019 | -2 | 0 |
| TON_1073 | TK1016 | 92 | 32 |
| TON_1075 | TK0329 | 11 | 9 |
| TON_1076 | TK0328 | 10 | 10 |
| TON_1077 | TK0327 | -6 | 0 |
| TON_1080 | TK0953 | 52 | 10 |
| TON_1083 | TK0950 | 43 | 11 |
| TON_1087 | TK0947 | 37 | 34 |
| TON_1088 | TK0946 | 10 | 11 |
| TON_1089 | TK0945 | 1 | 1 |
| TON_1090 | TK1140 | 12 | 10 |

| | | | |
|---|---|---|---|
| TON_1091 | TK1282 | 9 | 10 |
| TON_1093 | TK0670 | -54 | 0 |
| TON_1095 | TK0668 | 25 | 26 |
| TON_1097 | TK0665 | 25 | 25 |
| TON_1104 | TK1275 | 33 | 12 |
| TON_1105 | TK1283 | 13 | 135 |
| TON_1110 | TK1149 | 12 | 12 |
| TON_1113 | TK1155 | 28 | 19 |
| TON_1128 | TK0284 | 9 | 9 |
| TON_1130 | TK0271 | -87 | 58 |
| TON_1155 | TK0225 | 0 | 0 |
| TON_1166 | TK0061 | 5 | 3 |
| TON_1167 | TK0060 | 56 | 47 |
| TON_1169 | TK0058 | 10 | 10 |
| TON_1172 | TK0055 | 31 | 26 |
| TON_1191 | TK0036 | 93 | 67 |
| TON_1195 | TK0631 | 60 | 79 |
| TON_1204 | TK0641 | 155 | 34 |
| TON_1206 | TK0034 | 9 | 9 |
| TON_1208 | TK0032 | -2 | 1 |
| TON_1211 | TK0030 | 16 | 9 |
| TON_1212 | TK0028 | 10 | 10 |
| TON_1216 | TK0013 | 31 | 29 |
| TON_1217 | TK0012 | 191 | 110 |
| TON_1220 | TK0009 | 63 | 38 |
| TON_1221 | TK0008 | 0 | -2 |
| TON_1223 | TK0004 | 9 | 1 |
| TON_1229 | TK2293 | 0 | 0 |
| TON_1230 | TK2292 | 34 | 31 |
| TON_1231 | TK2291 | 24 | 20 |
| TON_1234 | TK2290 | 17 | 16 |
| TON_1239 | TK2285 | 15 | 12 |
| TON_1240 | TK2284 | 62 | 62 |
| TON_1245 | TK0849 | -11 | 10 |
| TON_1247 | TK2280 | 11 | 63 |
| TON_1248 | TK2279 | 47 | 56 |
| TON_1249 | TK2278 | 19 | 20 |
| TON_1250 | TK2277 | 12 | 11 |
| TON_1257 | TK2270 | 29 | 29 |
| TON_1258 | TK2269 | 10 | 12 |
| TON_1259 | TK2268 | 17 | 17 |
| TON_1270 | TK0305 | 8 | 9 |
| TON_1271 | TK0304 | 10 | 9 |
| TON_1278 | TK0302 | 1 | 0 |
| TON_1279 | TK0123 | 1 | 0 |
| TON_1280 | TK1831 | 8 | 9 |
| TON_1282 | TK0116 | 31 | 25 |
| TON_1283 | TK1286 | 27 | 13 |

| | | | |
|---|---|---|---|
| TON_1285 | TK1284 | 24 | 22 |
| TON_1290 | TK0154 | 9 | 108 |
| TON_1293 | TK0188 | 0 | 0 |
| TON_1295 | TK0186 | 52 | 58 |
| TON_1296 | TK0185 | 20 | 16 |
| TON_1298 | TK1750 | 26 | 24 |
| TON_1299 | TK0184 | 29 | 24 |
| TON_1301 | TK0181 | 33 | 32 |
| TON_1304 | TK0124 | 14 | 14 |
| TON_1305 | TK0125 | 1 | 0 |
| TON_1306 | TK0126 | 20 | 21 |
| TON_1307 | TK0129 | 9 | 9 |
| TON_1309 | TK0132 | 34 | 34 |
| TON_1313 | TK0139 | 9 | 12 |
| TON_1314 | TK0141 | 12 | 233 |
| TON_1315 | TK0142 | 10 | 10 |
| TON_1316 | TK0143 | 13 | 13 |
| TON_1317 | TK0144 | 10 | 9 |
| TON_1322 | TK0147 | 13 | 10 |
| TON_1323 | TK0149 | 18 | 67 |
| TON_1324 | TK0627 | 0 | 11 |
| TON_1325 | TK0626 | 0 | 0 |
| TON_1334 | TK0150 | 10 | 10 |
| TON_1337 | TK1326 | 39 | 38 |
| TON_1340 | TK1329 | 10 | 11 |
| TON_1342 | TK1331 | 26 | 22 |
| TON_1344 | TK1334 | 7 | 11 |
| TON_1345 | TK0652 | 12 | 12 |
| TON_1354 | TK1066 | 25 | 10 |
| TON_1356 | TK1690 | 12 | 12 |
| TON_1358 | TK1691 | 31 | 31 |
| TON_1359 | TK1692 | -50 | 0 |
| TON_1361 | TK1694 | 11 | 11 |
| TON_1362 | TK1676 | 11 | 12 |
| TON_1363 | TK1681 | 10 | 276 |
| TON_1365 | TK2254 | 11 | 10 |
| TON_1366 | TK2255 | 52 | 49 |
| TON_1372 | TK0106 | 10 | 32 |
| TON_1373 | TK1688 | 0 | 0 |
| TON_1374 | TK1686 | 12 | 10 |
| TON_1385 | TK2252 | 76 | 26 |
| TON_1388 | TK2250 | 11 | 39 |
| TON_1390 | TK2248 | 10 | 11 |
| TON_1394 | TK0827 | 0 | 0 |
| TON_1396 | TK2241 | 0 | 0 |
| TON_1397 | TK2240 | 11 | 10 |
| TON_1399 | TK2238 | 12 | 11 |
| TON_1406 | TK2232 | 13 | 39 |

| | | | |
|---|---|---|---|
| TON_1409 | TK2226 | 14 | 11 |
| TON_1410 | TK2225 | 10 | 11 |
| TON_1415 | TK2217 | 10 | 10 |
| TON_1418 | TK2214 | 0 | 0 |
| TON_1424 | TK2209 | 0 | 0 |
| TON_1426 | TK2207 | 11 | 12 |
| TON_1432 | TK2200 | 30 | 29 |
| TON_1437 | TK2196 | 8 | 8 |
| TON_1440 | TK2194 | 10 | 11 |
| TON_1441 | TK2193 | 16 | 1 |
| TON_1445 | TK2185 | 22 | 22 |
| TON_1454 | TK2169 | 9 | 12 |
| TON_1460 | TK2168 | 12 | 9 |
| TON_1461 | TK2167 | 0 | 0 |
| TON_1463 | TK1966 | 82 | 75 |
| TON_1464 | TK1967 | 12 | 17 |
| TON_1466 | TK1970 | 1 | 1 |
| TON_1468 | TK1972 | 173 | 173 |
| TON_1472 | TK1973 | 10 | 9 |
| TON_1473 | TK1974 | 10 | 9 |
| TON_1474 | TK1976 | -2 | 1 |
| TON_1475 | TK1977 | 24 | 23 |
| TON_1476 | TK1978 | 21 | 25 |
| TON_1480 | TK1982 | 37 | 10 |
| TON_1483 | TK2021 | 14 | 12 |
| TON_1486 | TK2024 | 60 | 67 |
| TON_1487 | TK2025 | 36 | 34 |
| TON_1488 | TK2029 | 15 | 16 |
| TON_1491 | TK2032 | 13 | 10 |
| TON_1496 | TK2165 | 100 | 11 |
| TON_1497 | TK2164 | 12 | 10 |
| TON_1498 | TK2163 | 73 | 71 |
| TON_1503 | TK0130 | 5 | 13 |
| TON_1504 | TK2157 | 55 | 50 |
| TON_1508 | TK2152 | 10 | 10 |
| TON_1511 | TK2135 | 0 | 5 |
| TON_1512 | TK2136 | 10 | 12 |
| TON_1516 | TK2141 | 2 | 11 |
| TON_1517 | TK2142 | 23 | 22 |
| TON_1519 | TK2144 | 9 | 9 |
| TON_1520 | TK2145 | 29 | 26 |
| TON_1522 | TK2148 | 9 | 32 |
| TON_1523 | TK2149 | 0 | 0 |
| TON_1525 | TK2151 | 31 | 10 |
| TON_1526 | TK2133 | 9 | 9 |
| TON_1528 | TK2131 | 43 | 53 |
| TON_1529 | TK2130 | 13 | 10 |
| TON_1531 | TK1907 | 83 | 84 |

| | | | |
|---|---|---|---|
| TON_1532 | TK1908 | 157 | 124 |
| TON_1534 | TK2038 | 57 | 0 |
| TON_1535 | TK2039 | 3 | 4 |
| TON_1545 | TK2051 | 7 | 4 |
| TON_1546 | TK2052 | 10 | 12 |
| TON_1552 | TK2061 | 1 | 1 |
| TON_1553 | TK2062 | 11 | 17 |
| TON_1554 | TK2063 | 9 | 8 |
| TON_1557 | TK2064 | 1 | 1 |
| TON_1558 | TK2065 | 56 | 59 |
| TON_1563 | TK2076 | 78 | 45 |
| TON_1582 | TK2080 | 60 | 37 |
| TON_1596 | TK2096 | 10 | 0 |
| TON_1599 | TK2097 | 53 | 53 |
| TON_1600 | TK2098 | 11 | 11 |
| TON_1608 | TK2104 | 7 | 11 |
| TON_1609 | TK2105 | 0 | 12 |
| TON_1613 | TK2106 | 12 | 12 |
| TON_1617 | TK2112 | 10 | 13 |
| TON_1618 | TK2113 | 10 | 21 |
| TON_1623 | TK2118 | 12 | 12 |
| TON_1624 | TK2119 | 11 | 10 |
| TON_1632 | TK2124 | 0 | 22 |
| TON_1634 | TK2126 | 1 | 2 |
| TON_1638 | TK2128 | 0 | 0 |
| TON_1640 | TK2129 | 10 | 10 |
| TON_1653 | TK1891 | 19 | 23 |
| TON_1663 | TK1881 | 81 | 28 |
| TON_1666 | TK1874 | 95 | 94 |
| TON_1672 | TK1857 | 91 | 91 |
| TON_1680 | TK1849 | 11 | 10 |
| TON_1689 | TK1836 | 11 | 11 |
| TON_1690 | TK1835 | 9 | 8 |
| TON_1691 | TK1833 | 77 | 65 |
| TON_1692 | TK1821 | 8 | 10 |
| TON_1693 | TK1824 | 10 | 10 |
| TON_1694 | TK1820 | 11 | 26 |
| TON_1709 | TK1817 | 8 | 104 |
| TON_1713 | TK1811 | 0 | 0 |
| TON_1718 | TK1557 | 9 | 8 |
| TON_1719 | TK1558 | 9 | 10 |
| TON_1721 | TK1561 | 0 | 0 |
| TON_1722 | TK1562 | 12 | 11 |
| TON_1731 | TK1571 | 11 | 11 |
| TON_1733 | TK1574 | 13 | 13 |
| TON_1736 | TK1580 | -24 | 0 |
| TON_1737 | TK1581 | 1 | 1 |
| TON_1742 | TK1592 | 12 | 11 |

| | | | |
|---|---|---|---|
| TON_1743 | TK1593 | 33 | 34 |
| TON_1758 | TK1551 | 0 | 11 |
| TON_1765 | TK1803 | 40 | 53 |
| TON_1772 | TK1796 | 41 | 41 |
| TON_1773 | TK1795 | 0 | 0 |
| TON_1775 | TK1793 | 11 | 11 |
| TON_1778 | TK1790 | 13 | 10 |
| TON_1779 | TK1789 | 50 | 52 |
| TON_1782 | TK1786 | 9 | 11 |
| TON_1784 | TK1784 | 0 | 0 |
| TON_1789 | TK1777 | 9 | 7 |
| TON_1790 | TK1776 | 0 | 0 |
| TON_1795 | TK1771 | 28 | 26 |
| TON_1796 | TK1770 | 26 | 26 |
| TON_1797 | TK1769 | 50 | 52 |
| TON_1800 | TK1766 | 22 | 14 |
| TON_1803 | TK1748 | 118 | 103 |
| TON_1804 | TK1746 | 12 | 13 |
| TON_1810 | TK1744 | 9 | 9 |
| TON_1813 | TK1738 | 10 | 9 |
| TON_1815 | TK1737 | 59 | 59 |
| TON_1818 | TK1734 | 13 | -30 |
| TON_1820 | TK1718 | 13 | 104 |
| TON_1821 | TK1733 | 17 | 3 |
| TON_1836 | TK1708 | 10 | 12 |
| TON_1837 | TK1711 | 2 | 26 |
| TON_1852 | TK1712 | 12 | 3 |
| TON_1865 | TK0111 | 36 | 28 |
| TON_1870 | TK2298 | 28 | 33 |
| TON_1884 | TK0194 | 21 | 22 |
| TON_1885 | TK0297 | 10 | 11 |
| TON_1887 | TK0296 | 10 | 13 |
| TON_1888 | TK1314 | 0 | 0 |
| TON_1892 | TK0209 | 10 | 9 |
| TON_1901 | TK1667 | 13 | 18 |
| TON_1904 | TK1670 | 11 | 12 |
| TON_1905 | TK1671 | 11 | 23 |
| TON_1906 | TK1673 | 0 | 0 |
| TON_1918 | TK1909 | 22 | 16 |
| TON_1920 | TK1913 | 34 | 29 |
| TON_1925 | TK1917 | 25 | 154 |
| TON_1933 | TK1926 | 27 | 26 |
| TON_1938 | TK1935 | 11 | 10 |
| TON_1940 | TK1941 | 0 | 0 |
| TON_1942 | TK1944 | 11 | 15 |
| TON_1944 | TK1946 | 14 | 12 |
| TON_1945 | TK1951 | 53 | 50 |
| TON_1946 | TK1952 | 14 | 34 |

| | | | |
|---|---|---|---|
| TON_1948 | TK1954 | 10 | 13 |
| TON_1949 | TK1956 | 0 | 0 |
| TON_1950 | TK1957 | 11 | 9 |
| TON_1954 | TK1961 | 12 | 12 |
| TON_1955 | TK1962 | 14 | 9 |
| TON_1956 | TK0107 | 173 | 11 |
| TON_1960 | TK0160 | 9 | 34 |
| TON_1966 | TK2304 | 0 | 0 |
| TON_1973 | TK0187 | 11 | 10 |
| TON_1976 | TK1955 | 112 | 61 |
